# Stereodivergent Total Syntheses of (+)‐Mycaperoxides C, D, G Methyl Ester and (−)‐Mycaperoxide B**

**DOI:** 10.1002/chem.202203004

**Published:** 2022-12-07

**Authors:** Mansour D. Kerim, Laurent Evanno, Laurent Ferrié

**Affiliations:** ^1^ BioCIS CNRS Université Paris-Saclay Bâtiment Henri-Moissan, 17 avenue des Sciences 91400 Orsay France

**Keywords:** endoperoxide, NMR analysis, peroxycarbenium ion ions, ring-expansion, total synthesis

## Abstract

Mycaperoxides are natural endoperoxides isolated from different Mycale genus sponges, showing significant antiviral or antibacterial activities. We report herein the first total syntheses of representative congeners of this family from sclareol using a stereodivergent approach. Thus, an innovative oxidative ring expansion of cyclobutanol was used to bring the 1,2‐dioxane subunit, and a Mukaiyama aldol reaction on peroxycarbenium species was utilized to install the propionic acid subunit. During the study toward (+)‐mycaperoxide D methyl ester (**2)**, the isolation of the eight possible diastereomers under their ethyl thioester form allowed to build a pertinent database for further NMR assignment studies. Thus, we completed the total syntheses of (+)‐mycaperoxides D, C, G methyl ester, and (−)‐mycaperoxide B in 11 to 15 steps, confirming their original assignment.

## Graphical abstract

**Totally expanded**. (+)‐Mycaperoxides D, C, G methyl ester, and (−)‐mycaperoxide B were synthesized in 11 to 15 steps for the first time. A stereodivergent approach from sclareol was adopted, highlighting ring expansion of strain cyclobutanol into 1,2‐dioxane, and Mukaiyama aldol reaction on peroxycarbenium species to install the propionic acid subunit.

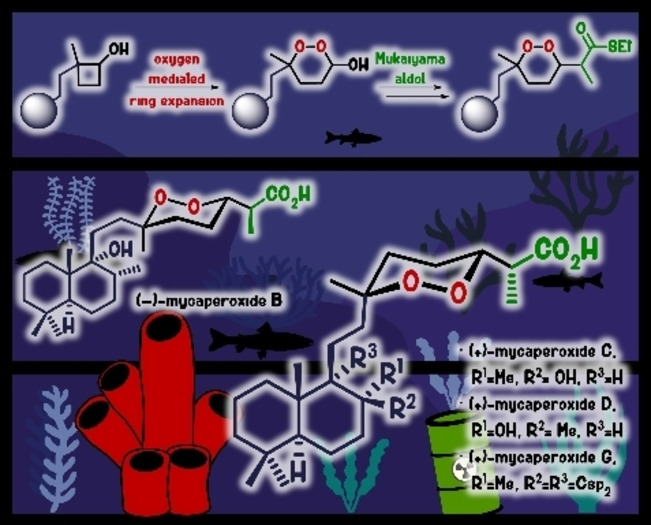

## Introduction

**Figure 1:**
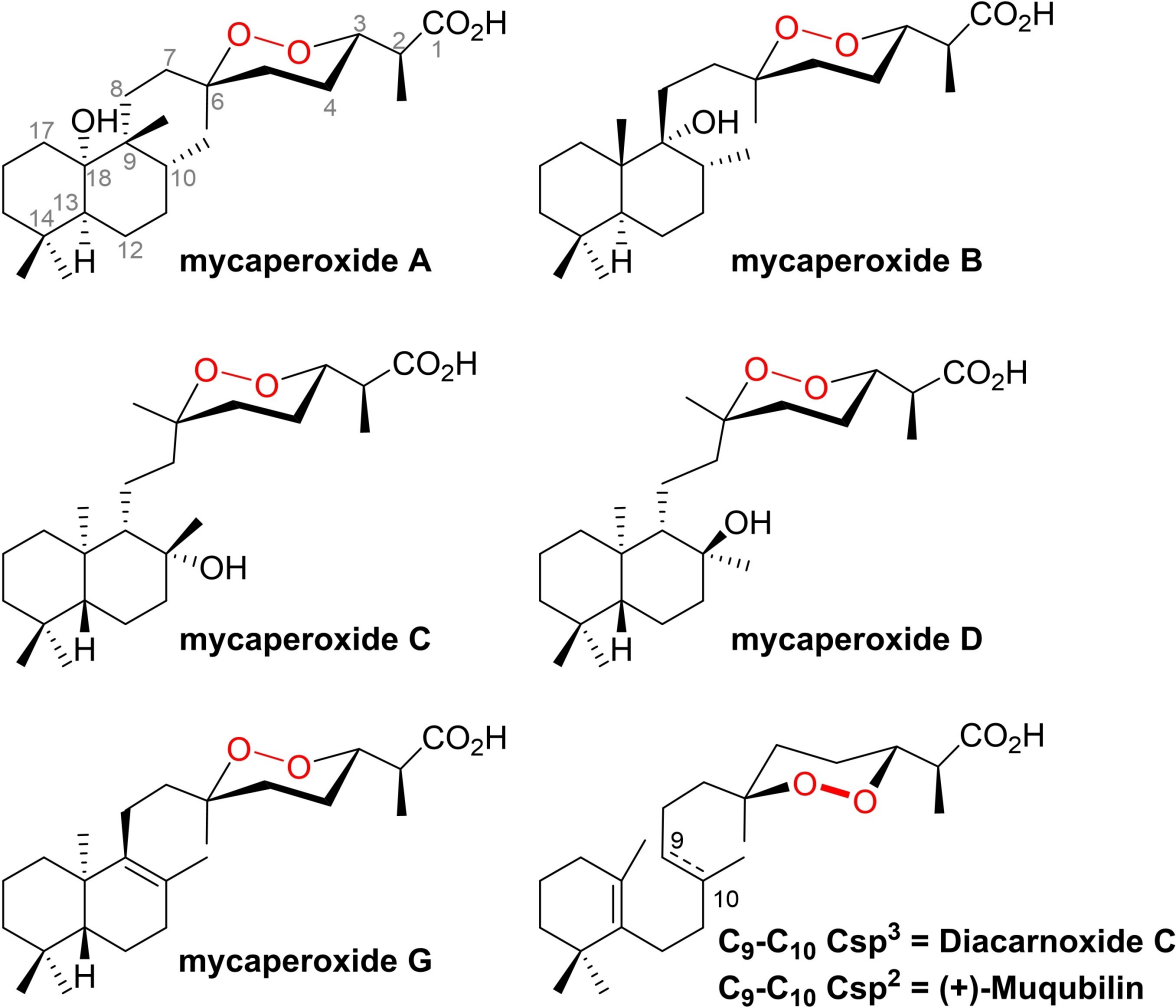
Mycaperoxides A–D and G, and diacarnoxide C.

The endoperoxide motif is an uncommon chemical function in natural products. However, a relatively large group of marine norsesterterpene metabolites represent a significant class of natural endoperoxides.[^1^, ^2^, ^3^] Indeed, more than 70 members have been isolated from various sponges of the class Demospongiae, including the genera *Prianos*, *Sigmosceptrella*, *Lacuntrulia*, *Diacarnus* or *Mycale*. Structurally, these endoperoxides have a common backbone consisting of a 1,2‐dioxane ring substituted by a propionic acid subunit on one side and a sesquiterpene subunit connected via a methylene bridge on the other side. The main difference between all members lies in the nature of the sesquiterpene side chain and, in particular, the degree of cyclization of the chain, the intervention of methyl transpositions, and the enantiomeric series of this chain. The absolute configuration of the stereogenic centers of this subclass of natural products is not identical from one product to another, depending mainly on their origin. Despite many members, this subclass has been barely studied at the synthetic level. Indeed, Harwood reported several synthetic studies towards the total synthesis of mycaperoxide B, leading, with difficulty, to the obtention of four inseparable diastereomers, in a total of 16 steps, sharing the gross structure of the target. However, none of them corresponded to the natural product.[^4^, ^5^, ^6^, ^7^] Later, Seifert reported the total synthesis of diacarnoxide C in about 20 steps but obtained as an inseparable mixture of *cis* and *trans*‐1,2‐dioxanes with epimerization at C_6_ (Figure 1).^[8]^ Lately, Wu and co‐workers reported the enantioselective total synthesis of (+)‐Muqubilin from seudenol, by adding hydrogen peroxide to a chiral epoxide. Still, a long synthetic sequence was necessary to reach this objective (29 linear steps).^[9]^

Recently, we have developed a synthetic method to construct 1,2‐dioxanes from the oxidative expansion of cyclobutanols,^[10]^ followed by the alkylation of the resulting peroxyacetals.[^10^, ^11^, ^12^] Thus, we felt it would be particularly well suited to the total synthesis of several members of this family of norsesterterpene endoperoxides. Mycaperoxides were targeted as some members have been reported to have significant antiviral activities (IC_50_=0.25–1.0 μg/mL against vesicular stomatitis virus and herpes simplex virus type‐1) and antibacterial properties (inhibition of the growth of gram‐positive bacteria *Bacillus subtilis* and *Staphylococcus aureus*).^[13]^

## Results and Discussion

**Scheme 1:**
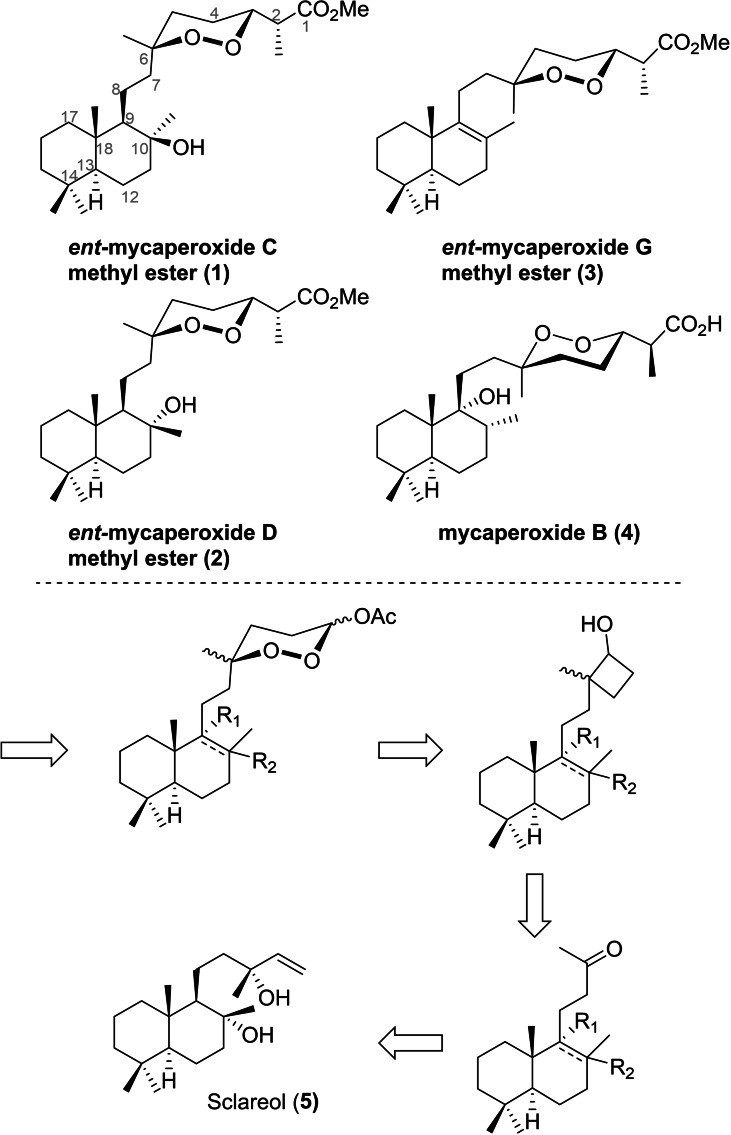
Stereodivergent strategy from sclareol to access *ent*‐mycaperoxides C, D, and G, and mycaperoxide B.

In terms of synthetic access, these molecules, mainly mycaperoxides B, C, D, and G, would be strategically accessible from sclareol (*Salvia sclarea*) used as starting synthon. Concerning the chirality control strategy, mycaperoxide B (**4**) would be directly accessible from sclareol as the latter corresponds to the proper enantiomeric series of the decalin moiety. For the mycaperoxides C, D, and G, their antipodes (*ent*‐mycaperoxides C, D, and G) would be accessible by total synthesis from sclareol. For these three compounds, methyl esters (**1**), (**2**), and (**3**) were targeted as the natural products were isolated and characterized in this form after derivatization with diazomethane.[^14^, ^15^] Thus, a stereodivergent total synthesis was collectively designed for all these products. They could be obtained by a Mukaiyama aldol reaction performed on an acetoxy‐endoperoxyketal.[^10^, ^11^, ^12^] The 1,2‐dioxane ring of the latter could be constructed from a cyclobutanol by ring expansion with molecular oxygen mediated by Co(acac)_2_.^[10]^ The cyclobutanol could be easily obtained from a methyl ketone derived from sclareol. The four required decalins would thus be prepared from the same starting synthon via divergent functional arrangements. (Scheme 1)

**Scheme 2:**
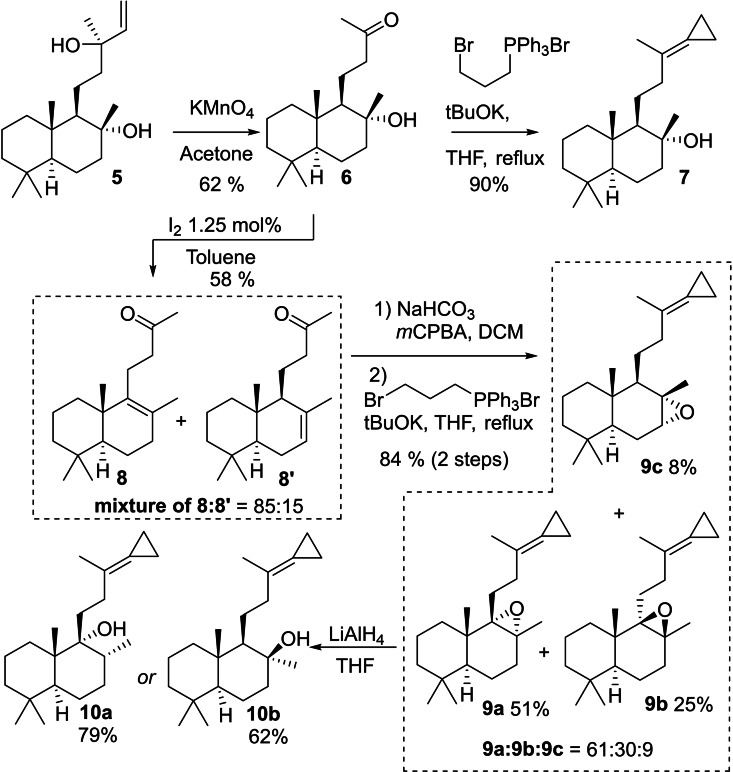
Divergent synthesis of sesquiterpene moiety of *ent‐*mycaperoxides C, D, G and mycaperoxide B from sclareol.

The synthesis of the different targeted mycaperoxides consisted first of the functionalization of sclareol (**5**). The latter underwent an oxidative cleavage with KMnO_4_ leading to methyl ketone **6**.^[16]^ A Wittig olefination then led to the formation of cyclopropylidene **7**,^[17]^ the precursor of mycaperoxide D. To obtain the other congeners, a hydroxyl elimination gave rise to a mixture of isomers **8** and **8’** in an 85 : 15 ratio and thus gave access to the other required decalins. Epoxidation of the **8**/**8’** mixture followed by Wittig olefination provided the three separable isomers **9 a**, **9 b**, and **9 c** in a 61 : 30 : 9 ratio. Compounds **9 a** and **9 b** were subjected to reduction with LiAlH_4_. From **9 a**, hydride attack led exclusively to **10 a**, the precursor of mycaperoxide B and *ent*‐mycaperoxide G. The same reaction performed on **9 b** gave only **10 b**, the precursor of *ent*‐mycaperoxide C. The regioselectivity of hydride attack is in agreement with the Fürst‐Plattner rule.[^18^, ^19^, ^20^] (Scheme 2)

**Scheme 3:**
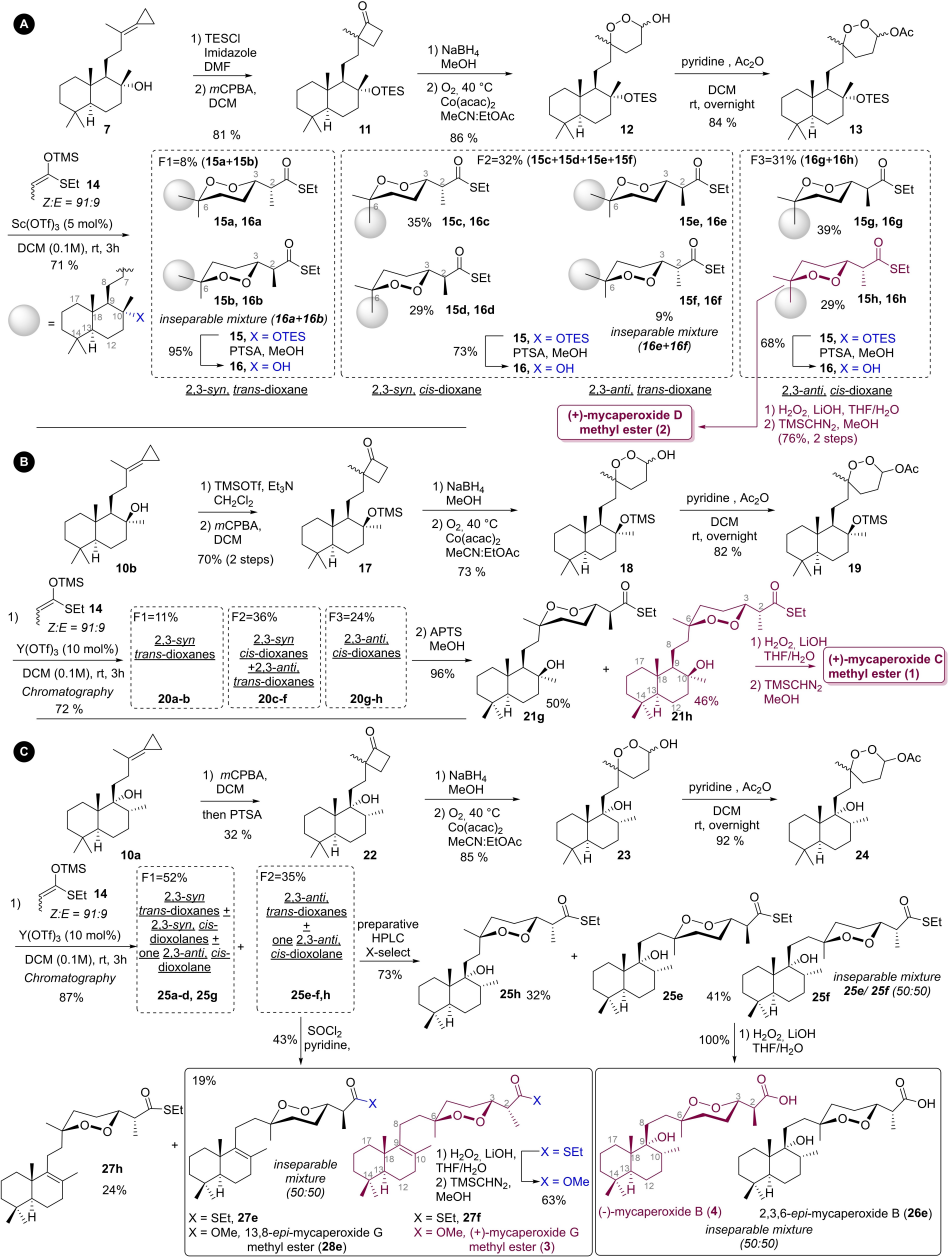
Stereodivergent total of mycaperoxides: A) (+)‐mycaperoxide D methyl ester; B) (+)‐mycaperoxide C methyl ester; C) (+)‐mycaperoxide G methyl ester and (−)‐mycaperoxide B.

The synthesis of *ent*‐mycaperoxide D was firstly studied for its better accessibility. Thus, after the protection of the hydroxyl in the form of a silyl ether (TES), the cyclopropylidene was epoxidized. *In situ* acid‐catalyzed rearrangement led then directly to cyclobutanone **11**. After reduction with NaBH_4_, the resulting cyclobutanol underwent a ring enlargement reaction in the presence of Co(acac)_2,_ affording 1,2‐dioxanol **12** in good yield.^[10]^ Although any initial stereochemistry at C_3_ and C_6_ was lost during the process due to hydroperoxyacetal equilibrium and radical mechanism of the reaction, we observed that the decalin slightly induced stereoselectivity during the cyclobutanol oxidative ring opening (see below for further discussion). The hydroxyl was acetylated in the presence of pyridine,^[21]^ and the resulting acetoxy‐endoperoxyacetal **13** reacted with silylketenethioketal **14** ^[22]^ as well as a catalytic amount of Sc(OTf)_3_.^[10]^ At this step, the first resolution of diastereomers [2,3‐*syn*/2,3‐*anti* and *cis*/*trans*‐1,2‐dioxanes] was thus performed by silica gel chromatography. Therefore, three fractions (F1, F2, and F3) were obtained in 8 %, 32 %, and 31 % yield, respectively; each containing a mixture of defined diastereomers among the eight possible (Scheme 3, A). The stereoselectivity of the reaction was estimated to be 4 : 1 in favor of *cis‐*1,2‐dioxanes, but no selectivity was observed for the *syn*/*anti* relationship at C_2_‐C_3_. After cleavage of the silyl ether by acid methanolysis performed independently on each fraction (F1, F2 and F3), a further separation of diastereomers was again done to deliver thioesters **16 a**–**h**. All *cis‐*1,2‐dioxanes could be isolated as single stereoisomers but, *trans‐*1,2‐dioxanes, do not allow a separation of their 2,3,6‐epimer and were thus obtained as a mixture of two diastereomers. For the *trans‐*1,2‐dioxanes mixtures, a selectivity about 55 : 45 in favor of one particular 2,3,6‐isomer, allowed the differentiation of carbon for attribution.

**Figure 2:**
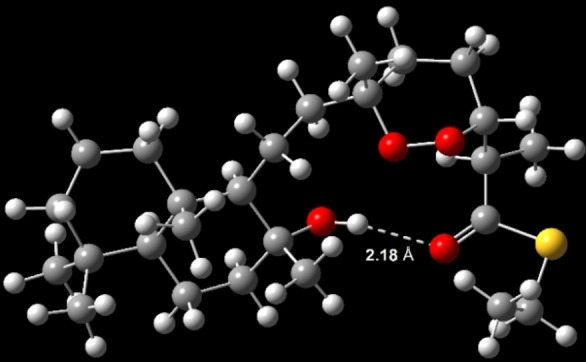
Evidence of a hydrogen bond on diastereomer **16 h** between the carbonyl group of thioester at C_1_ and hydroxy group at C_10_. DFT Energy minimization at the theoretical level B3LYP/6‐31G(d,p).

The structures of the different diastereomers were attributed as follow by using Capon's empirical rules^[23]^ based on the ^13^C NMR chemical shift. Firstly, *cis*/*trans*‐1,2‐dioxanes were assigned thanks to ^13^C NMR and the characteristic chemical shifts of the methyl groups at position C_6_ (20.0–21.0 ppm for *trans*‐dioxanes vs. 23.5–23.7 ppm for *cis*‐dioxanes) and of position C_7_ (43.0–43.9 ppm for *trans‐*dioxanes vs. 37.7–39.1 ppm for *cis*‐dioxanes). Secondly, the 2,3‐*syn or* 2,3‐*anti* configurations were then attributed based on the characteristic chemical shifts of the methyl groups at position C_2_ (13.2–13.3 ppm for 2,3‐*anti* vs. 14.4–14.9 ppm for 2,3‐*syn* products) and of position C_4_ (23.4–23.7 ppm for 2,3‐*anti* vs. 22.6–22.8 ppm for 2,3‐*syn* products). Thirdly, 2,3,6‐epimer pairs [2,3‐*syn*, *trans‐*dioxane **16 a** vs. **16 b**, 2,3‐*syn*, *cis‐*dioxane **16 c** vs. **16 d**, etc.] were the most difficult to discriminate due to the slight chemical shift differences due to the spacer between decalin and 1,2‐dioxane units. Structure of *cis*‐dioxanes **16 g** and **16 h** were deduced from the similarities observed on ^13^C NMR with natural (−)‐mycaperoxide D. Although tight chemical shift differences at C_7_ and C_8_, these positions are characteristic (±0.2–0.8 ppm difference for each 2,3,6‐epimer pairs) and *cis*‐dioxanes **16 c** and **16 d** were next assigned. A more significant difference can be observed at C_11_ for *cis*‐dioxanes **16 c** and **16 g** versus *trans*‐dioxanes **16 d** and **16 h** (+1.6 ppm), which is indicative of a hydrogen bond of the hydroxyl at C10. Indeed, DFT calculation at the B3LYP/6‐31G(d,p) showed that among the lowest energy conformers, an evident hydrogen bond exists between this hydroxyl group and carbonyl group at C1 with a distance of 2.18 Å (Figure 2). This particularity also explains the ability of *cis‐*dioxanes **6 c**–**d** and **6 g**–**h** to be isolatable as lone diastereomers compared to protected compounds **15 a**–**h** and *trans*‐dioxanes where this hydrogen bond is not possible. Thus, collectively taking into account the ^13^C NMR chemical shifts at positions C_7_, C_8_, and C_11_, 2,3,6‐epimers **16 a**, **16 b**, **16 e**, and **16 f** were assigned.

Among the eight diastereomers **16 a**–**h**, thioester **16 h** was spectroscopically the best match with mycaperoxide D methyl ester. Thus, in two steps, **16 h** was transformed into methyl ester **2** by transesterification. Spectral data of synthetic **2** were in agreement with the description of the natural product and the antipode sign of the optical rotation compared to those of natural mycaperoxides D methyl ester [+68 (*c* 0.25, CHCl_3_); lit:^[13]^ −52 (*c* 0.3, CHCl_3_)] confirmed the original assignment of mycaperoxide D (Scheme 3A).

Afterward, the synthesis of (+)*‐*mycaperoxide C methyl ester (**1**) was investigated. Thus, endoperoxyacetal **19** was obtained in five steps from **10 b**, using a sequence of ring‐expansion of cyclobutanone **17** producing endoperoxide **18**, similar to those described for the total synthesis of **2**. A notable difference between the two synthetic routes lies in using TMS as the protecting group for the hydroxy group at C10. The conformation of **10 b** did not allow the introduction of TES; consequently, only the use of TMS‐triflate with Et_3_N succeeded in the protection step. However, the use of TMS led to an unexpected consequence. Upon Mukaiyama‐type aldol reaction, in the presence of Sc(OTf)_3_ on **19**, the hydroxyl at C10 was eliminated. The use of less active Y(OTf)_3_ ^[11]^ allowed the aldolization to be carried out without significant side‐reaction (Scheme 3B). The selectivities were comparable for the stereocenters at C_6_ and C_2_ to that was obtained in the total synthesis of **2**. Again, during this synthesis, the formation of the *cis*‐dioxane rings (C_3_) was favored over the *trans* one, with a 2 : 1 *cis:trans* ratio. As described in the total synthesis of **2**, we obtained a mixture of diastereomers distributed over three fractions. The fraction F3 was also containing the isomer corresponding to the natural product according to ^13^C NMR data. After deprotection of the alcohol function, the compound of interest **21 h** was separated from its isomer **21 g**. The final transesterification performed on **21 h** led to the synthesis of the (−)*‐*mycaperoxide methyl ester C (**1**). Spectral data matched to the described natural product and, again, the optical rotation [+50 (*c* 0.198, CHCl_3_); lit:^[13]^ −71 (*c* 1.1, CHCl_3_)] confirmed the synthesis of *ent*‐mycaperoxide C methyl ester and the original assignment of mycaperoxide C (Scheme 3B).

Regarding (+)‐mycaperoxide methyl ester G (**3**) and (−)‐mycaperoxide B (**4**), their total synthesis was planned from compound **10 a**. The same reaction sequence used to prepare intermediates **13** and **19** (Scheme 3A and B) led to the formation of **24** in five steps. However, here again, a particular reactivity of substrate **10 a** led us to some adaptations. Hydroxyl in position C_9_ was reluctant to protection and systematically underwent an elimination reaction during all attempts to form a silyl ether. It was thus chosen to leave it free for the rest of the synthesis, its tertiary position inducing a steric hindrance making it less accessible and therefore less reactive. Indeed, the acetylation of **23** was carried out chemoselectively on the 1,2‐dioxane hydroxyl. To avoid further elimination reactions of the hydroxyl at the C_9_ position during the Mukaiyama aldol reaction, Y(OTf)_3_ was used again, which led to the obtention of two fractions, F1 and F2 in 87 % yield (1,2‐dioxane *cis*:*trans*=3 : 2; 2,3‐*syn*:2,3‐*anti*=1 : 1). The fraction of interest, F2, contained a mixture of three products, including isomers **25 e** and **25 f**, which corresponded to the relative stereochemistry of mycaperoxide B (**4**) and (+)*‐*mycaperoxide G methyl ester (**3**) respectively. The utilization of preparative HPLC for fraction F2 led to the separation of isomer **25 h** ^[24]^ from the mixture of isomers **25 e/25 f**. Thioesters **25 e** and **25 f** were saponified, providing mycaperoxide B (**4**) in an inseparable mixture with **26 e** isomer in about a 53 : 47 ratio. Assignment of isomers **25 e** and **25 f** (or **26 e** and **4**) was tricky due to tenuous differences in the ^13^C NMR chemical shifts for those compounds [*δ*=0.1 ppm only for three carbons (C_7_, C_10_‐Me, and C_18_)]. Data for synthetic **4** were slightly closer to the reported natural mycaperoxides B than isomer **26 e**, thus seeming to confirm the original statement. (Scheme 3C)

From the fraction F2 containing isomers **25 e**–**g**, elimination of hydroxyl group induced by the action of SOCl_2_ led selectively to the tetrasubstituted olefin. Compound **27 g** and a mixture of **27 e** and **27 f** were thus obtained. The combination of **27 e**/**27 f** was transesterified, finalizing the synthesis of the (+)‐mycaperoxide methyl ester G (**3**) as a mixture with **28 e**. As for **26 e** and **4**, the ^13^C NMR data for products **3** and **28 e** were very close. The most significant differences were observed for the ethylenic carbons at C_9_ and C_10_ [*δ*=0.2 ppm]. The synthetic product with NMR signals matching the most with the natural product was attributed to structure **3** (Scheme 3C).

## Conclusion

In summary, we were able to construct for the first time several members of the mycaperoxides, about thirty years after their discovery, employing an oxidative expansion of cyclobutanol to 1,2‐dioxane followed by a Mukaiyama aldol reaction on a peroxycarbenium species as key steps. Thus, the methyl esters of (+)‐mycaperoxides C (**1**) and D (**2**) were obtained in 15 and 11 steps, respectively. The methyl ester of (+)‐mycaperoxide G (**3**) and (−)‐mycaperoxide B (**4**) were obtained in 14 and 13 steps but combined with their respective 2,3,6‐epimer. Original assignments were thus confirmed. We highlighted the utility of yttrium compared to scandium in the Lewis acid‐catalyzed Mukaiyama aldol reaction, which allowed smoother conditions reducing elimination side reaction with tertiary alkoxides. Although our approach did not allow us to control all the stereogenic centers of the synthetic targets, the different mycaperoxides studied were obtained efficiently accordingly to literature precedents.[^7^, ^8^] In addition, obtaining several well‐identified diastereomers provides a valuable NMR database for the assignment of new natural endoperoxides of the same family. 2,3‐*syn*/2,3‐*anti* and *cis*/*trans*‐1,2‐dioxanes relationships are easily determined by this method. However, the assignment of the 2,3,6‐epimers is more challenging, leading to small differences in NMR spectral data. Moreover, the affected positions are also a function of the nature of the decalin unit, which makes a general rule impossible for these pairs of diastereomers. This project also underlines the lack of enantiomeric methods to build organic peroxides, with few reported examples.[^25^, ^26^, ^27^] Therefore, significant work must be undertaken to overcome the actual limitations in the field.^[28]^ Such effort will be reported from our laboratory in due course.

## Experimental Section

General information, detailed experimental procedures, characterization data, cartesian coordinates of **16 h**, tables of NMR data for each mycaperoxides and copies of ^1^H and ^13^C NMR spectra of all compounds are in the Supporting Information.

## Conflict of interest

The authors declare no conflict of interest.

1

## Supporting information

As a service to our authors and readers, this journal provides supporting information supplied by the authors. Such materials are peer reviewed and may be re‐organized for online delivery, but are not copy‐edited or typeset. Technical support issues arising from supporting information (other than missing files) should be addressed to the authors.

Supporting Information

## Data Availability

The data that support the findings of this study are available from the corresponding author upon reasonable request.
