# Supplementary material for: Stereodivergent Total Syntheses of (+)‐Mycaperoxides C, D, G Methyl Ester and (−)‐Mycaperoxide B
Source: Chemistry. 2022 Dec 7;29(6):e202203004. doi: 10.1002/chem.202203004 (PMC10107902; doi:10.1002/chem.202203004)
Supplement: Supplementary file 1 — Supporting Information [file CHEM-29-0-s001.pdf]

Chemistry–A European Journal

Supporting Information

Stereodivergent Total Syntheses of (+)-Mycaperoxides C, D, G Methyl Ester and (−)-Mycaperoxide B

Mansour D. Kerim, Laurent Evanno, and Laurent Ferrié*

TABLE OF CONTENTS

I-General 3

II-Experimental Protocols and Characterization of Synthesized Compounds 4

Compound **6** 4
Compound **7** 4
Compound **8** 5
Compounds **9a-c** 6
Compound **10a** 8
Compound **10b** 9
Compound **11** 10
Compound **12** 11
Compound **13** 12
Compounds **15a-h** 13
Compounds **16a-b** 15
Compounds **16d**, **16c**, and **16e-f** 16
Compounds **16g-h** 18
(+)-mycaperoxide D methyl ester **(2)** 20

Table S1 21

Compound **17** 22
Compound **18** 23
Compound **19** 24
Compounds **20a-h** 25
Compounds **21g-h** 27
(+)-mycaperoxide C methyl ester (**1**) 29

Table S2 30

Compound **22** 31
Compound **23** 32
Compound **24** 33
Compound **25** 34
Compounds **26e/4** : 36
Compounds **27e,f,h** 37
Compounds **28f/3** 38

Table S3 40

II-Computational methods 41
(+)-Mycaperoxide D thioethyl ester **16h** 41

III-References 43

IV-Copies of ^{1}H and ^{13}C NMR spectra 45

I-General

All the reactions were performed under an inert atmosphere (Ar) unless specified. THF was distilled over a sodium/benzophenone mixture. Et_2O and CH_2Cl_2 were purified by filtration over activated molecular sieves. DMF was purchased as the anhydrous grade from Acros Organics and used as received. Acetone, synthesis grade, was used directly as received. Analytical thin-layer chromatography (TLC) was performed on silica gel 60 F_{254} (0.25 mm) plates purchased from Merck. Compounds were visualized by exposure to a UV lamp (λ = 254 and 365 nm), aqueous $KMnO_4/K_2CO_3$, or an acidic vanillin solution in EtOH, followed by gentle heating. Flash chromatographies were performed with Merck (230-400 mesh) silica gel.

^1H and ^{13}C NMR spectra were recorded using a Brucker Advance 300 (300 MHz) or a Brucker Advance 400 (400 MHz) spectrometers in the indicated solvent. An optimized sequence for 1D ^{13}C spectra, called UDEFT, was used.[1] Chemical shifts (δ) are given in ppm and the coupling constants (J) in Hz. The solvent signals were used as reference [$CDCl_3$: δ_C = 77.16 ppm (77.00 ppm in tables S1-S3 for comparison with reported natural products), residual $CHCl_3$ in $CDCl_3$: δ_H = 7.26 ppm]. Multiplicities are described by the following abbreviations: s = singlet, d = doublet, t = triplet, q = quartet, p = pentuplet, h = hextuplet, m = multiplet, br = broad. Infrared spectra were recorded using a Brucker IRTF Vector 22 spectrometer or a Shimadzu IR-Affinity-1S, and wavenumbers (ν) were given in cm^{-1}. HigHresolution mass spectra were obtained on a Waters LCT Premier (ESI-TOF) or Agilent spectrometer QTOF 6500 series. Optical rotations were measured on a Optical-Activity-polAAR 32 polarimeter or an Anton-Paar-MCP100 polarimeter.

CAUTION*: Although 1,2-dioxanes seems relatively stable in our hand, peroxides always present an evident risk of explosion, and all precautions must be taken to avoid any hazard. In particular, the rotavapor concentrations must be conducted with gentle heating, not exceeding 30 °C. Reaction temperatures should not exceed 35-40 °C in case of exotherm.*

II-Experimental Protocols and Characterization of Synthesized Compounds

Compound **6**

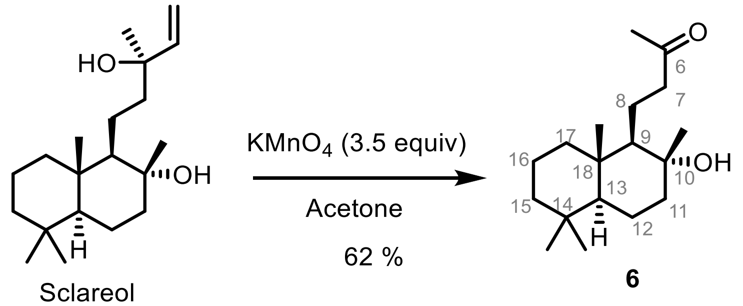

To a stirred solution of (-)-sclareol (10 g, 32.4 mmol, 1 equiv) in acetone (200 mL) was added portion wise $KMnO_4$ (16 g, 113.6 mmol, 3.5 equiv) and $MgSO_4$ (19.5 g, 162 mmol, 5 equiv) over 1 h and the reaction mixture was stirred at room temperature for 4 h. The slurry mixture was filtered through a pad of Celite, and the filter cake was washed with acetone (3x200 mL). The combined organic phase was concentrated under reduced pressure below 20 °C to afford **6** (5.59 g, 62 %) as white solid, which was directly used for the subsequent step reaction without further purification due to its tendency to undergo self-condensation.[2,3]

^{1}H NMR (300 MHz, Chloroform-*d*) δ 2.65 (dt, *J* = 17.8, 8.0 Hz, 1H, H_{7a}), 2.55 (ddd, *J* = 17.8, 7.7, 5.7 Hz, 1H, H_{7b}), 2.36 (brs, 1H), 2.11 (s, 3H, H_6-Me), 1.85 (dt, *J* = 12.0, 2.9 Hz, 1H), 1.75 (ddd, *J* = 14.9, 7.8, 4.0 Hz, 1H), 1.67 (ddd, *J* = 14.9, 8.0, 3.9 Hz, 1H), 1.65 – 1.56 (m, 3H), 1.57 – 1.47 (m, 1H), 1.47 – 1.30 (m, 2H), 1.30 – 1.19 (m, 1H), 1.19-1.11 (m, 1H), 1.13 (s, 3H, H_{10}-Me), 1.10 (dt, *J* = 16.2, 4.4 Hz, 1H), 0.97 – 0.86 (m, 2H), 0.85 (s, 3H, H_{14}-Meβ), 0.79 (s, 3H, H_{14}-Meα), 0.77 (s, 3H, H_{18}-Me).

^{13}C NMR (75 MHz, $CDCl_3$) δ 210.4 (C_6), 73.7 (C_{10}), 60.7 (C_9), 56.1 (C_{13}), 46.2 (C_7), 44.4 (C_{11}), 41.9 (C_{15}), 40.0 (C_{17}), 39.3 (C_{18}), 33.4 (C_{14}-Me), 33.2 (C_{14}), 29.9 (C_6-Me), 24.1 (C_{10}-Me), 21.5 (C_{14}-Me), 20.5 (C_{12}), 18.8 (C_{16}), 18.4 (C_8), 15.2 (C_{18}-Me).

Compound **7**

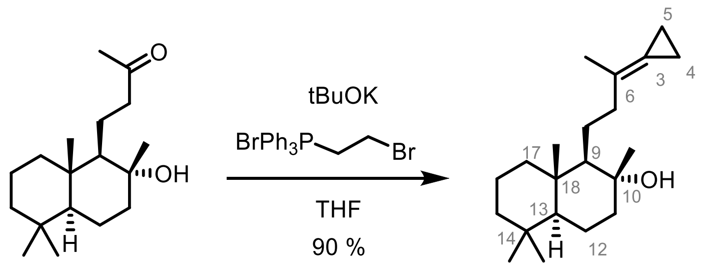

6 **7**

To a suspension of 1-bromopropyl-3-triphenylphosphonium bromide (23 g, 50 mmol, 2.5 equiv) in THF (200 mL) under argon atmosphere was added *t*-BuOK (5 equiv, 100 mmol, 11 g), and the reaction mixture turned to bright orange. The reaction mixture was then heated at reflux with an oil bath for 1 h. In parallel, **6** (5.6 g, 1 equiv, 20 mmol) was dissolved in THF (40 mL), and magnesium sulfate was added to dry the solution. The THF solution of **6** was filtered and added to the THF

solution of ylide. The reflux was continued for 4 h, and the reaction mixture was cooled down to room temperature. The reaction mixture was then poured into a large Erlenmeyer containing a large amount of pentane (400 mL), and the precipitates were filtered. The filtrate was concentrated. The crude was purified by flash chromatography over silica gel (PE/Et_2O, 100 :00 to 95 :05) to afford **7** as white solid (5.48 g, 90 %)

mp = 98 – 99 °C

[α]^{16}$_D$ = +2.1 (c 0.95, DCM)

IR (neat) : *v* = 3309, 2919, 1448, 1385, 1275, 1260 cm^{-1}.

^{1}H NMR (300 MHz, Chloroform-*d*) δ 2.26 (t, *J* = 8.0 Hz, 2H, H_7), 1.86 (dt, *J* = 12.0, 3.2, 2.7 Hz, 1H), 1.82 (p, *J* = 1.5 Hz, 3H, H_6-Me), 1.68 (dq, *J* = 12.5, 3.4, 1.6 Hz, 1H), 1.67 – 1.59 (m, 2H), 1.57 (ddd, *J* = 5.3, 3.6, 1.7 Hz, 1H), 1.54 – 1.42 (m, 2H), 1.41 – 1.38 (m, 1H), 1.37 – 1.33 (m, 1H), 1.33 – 1.26 (m, 1H), 1.25 – 1.17 (m, 1H), 1.17 – 1.13 (m, 1H), 1.13 (s, 3H, H_{10}-Me), 1.09 – 1.01 (m, 3H), 1.00 – 0.97 (m, 1H), 0.97 – 0.94 (m, 1H), 0.94 – 0.91 (m, 1H), 0.87 (s, 3H, H_{14}-Meβ), 0.80 (s, 3H, H_{14}-Meα), 0.79 (s, 3H, H_{18}-Me).

^{13}C NMR (75 MHz, $CDCl_3$) δ 125.5 (C_6), 115.4 (C_3), 74.2 (C_{10}), 61.8 (C_9), 56.4 (C_{13}), 44.7 (C_{11}), 42.2 (C_{15}), 40.3 (C_{17}), 39.8 (C_7), 39.4 (C_{18}), 33.6 (C_{14}-Me), 33.4 (C_{14}), 24.0 (C_{10}-Me), 23.6(C_8), 21.7 (C_{14}-Me), 20.9 (C_6-Me), 20.7 (C_{12}), 18.6 (C_{16}), 15.6 (C_{18}-Me), 3.2 ($C_{5/4}$), 1.8 ($C_{4/5}$).

HRMS (ESI) : calculated for $C_{21}H_{35}$ $[M-H_2O+H]^+$: 287.2739, found 287.2735

Compound **8**

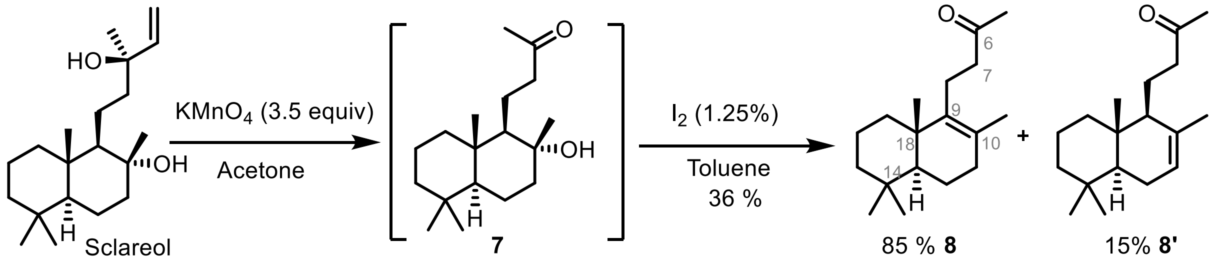

To a stirred solution of (-)-sclareol (10 g, 32.4 mmol, 1 equiv) in acetone (200 mL) was added portion-wise $KMnO_4$ (16 g, 3.5 equiv, 113.6 mmol) and $MgSO_4$ (19.5 g, 162 mmol, 5 equiv) for 1 h. The reaction mixture was stirred at ambient temperature for 4 h. The slurry mixture was filtered through a pad of Celite, and the filter cake was washed with acetone (3x200 mL). The combined organic phase was concentrated under reduced pressure and was added successively to toluene (500 mL) and I_2 (104 mg, 0.41 mmol, 1.25 mol%). The resulting mixture was refluxed with Dean-Stark equipment for 3 h. After cooling, an aqueous $Na_2S_2O_3$ solution was added to quench the reaction and extracted with ethyl acetate. The organic layer was dried over sodium sulfate, filtered, and concentrated, and the residue was purified by column chromatography to give compounds **8** and **8’** as an inseparable mixture (3.06 g, 36 %, **8**:**8’** = 85:15).[2,4]

^{1}H NMR (300 MHz, Chloroform-*d, major Isomer **8***) δ 2.42 (t, *J* = 8.1 Hz, 2H), 2.30 – 2.15 (m, 1H), 2.06 (s, 3H, H_6-Me), 2.14 – 1.99 (m, 1H), 1.98 – 1.83 (m, 2H), 1.76 – 1.66 (m, 1H), 1.63 – 1.49 (m, 2H), 1.47 (s, 3H, H_{10}-Me), 1.45 – 1.24 (m, 3H), 1.13 – 0.95 (m, 3H), 0.88 – 0.85 (m, 3H, H_{14}-Meα), 0.81 (s, 3H, H_{14}-Meα), 0.76 (s, 3H, H_{18}-Me).

^{13}C NMR (75 MHz, CDCl$_3$, *major isomer **8***) δ 209.0 (C$_3$), 139.5 (C$_9$), 126.7 (C$_{10}$), 52.2 (C$_{13}$), 44.8 (C$_7$), 42.0 (C$_{15}$), 39.3 (C$_{18}$), 37.2 (C$_{17}$), 33.8 (C$_{11}$), 33.5 (C$_{14}$+C$_{14}$-Me), 29.9 (C$_6$-Me), 21.8 (C$_{14}$-Me/C$_{18}$-Me), 21.8 (C$_{14}$-Me/C$_{18}$-Me), 20.1 (C$_8$), 19.6 (C$_{16}$), 19.2 (C$_{10}$-Me+C$_{12}$).

^{13}C NMR (75 MHz, CDCl$_3$, *minor isomer **8'***) δ 209.0, 134.6, 123.2, 54.6, 50.3, 46.1, 42.4, 39.5, 37.1, 33.3, 33.1, 30.1, 23.9, 22.3, 22.0, 21.1, 18.9.

<u>Compounds **9a-c**</u>

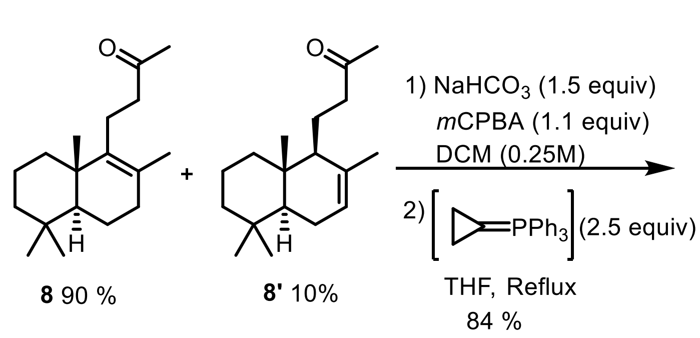

To a solution of **8** and **8'** (6.04 g, 23 mmol, 1 equiv) and NaHCO$_3$ (2.9 g, 34.5 mmol, 1.5 equiv) in DCM (92 mL) was added, *m*-CPBA (6.04 g, 25.3 mmol, 1.1 equiv, 70-77% in water) portion by portion during 5 minutes. The reaction mixture was stirred at rt for 15 min. Then 10 mL of sodium thiosulfate solution was added to the mixture, and the resulting mixture was stirred vigorously for an additional 15 min and poured into 100 mL of sodium bicarbonate solution. The aqueous layer was extracted twice with DCM (100 mL), and the combined organics layers were washed with 50 mL of sodium bicarbonate solution, dried over MgSO$_4$, filtered, and concentrated under vacuum to afford a mixture of epoxides as residue. Due to their sensitivity to silica gel, the crude mixture was subjected to the next step without further purification.

In parallel, to a suspension of 1-bromopropyl-3-triphenylphosphonium bromide (23 g, 57.5 mmol, 2.5 equiv) in THF (200 mL) under argon atmosphere was added *t*-BuOK (12.9 g, 115 mmol, 5 equiv), and the reaction mixture turned to bright orange. The reaction mixture was then heated at reflux with an oil bath for 1 h.

The crude mixture of the above epoxides was dissolved in THF (46 mL) and added to the above phosphorous ylide solution. The resulting mixture was refluxed for an additional 2 h, and the reaction mixture was cooled down to room temperature. Then the reaction mixture was poured into a large Erlenmeyer containing a large amount of pentane (400 mL), and the precipitates were filtered. The filtrate was concentrated, and the residue was purified by flash chromatography over silica gel (PE:Et$_2$O, 100:00 to 95:05) to afford **9a** (3.3 g, 30%, contaminated with about 4% PPh$_3$), **9b** (1.6 g, 30%), **9c** (458 mg, 24%, contaminated with about 2% PPh$_3$). The global yield was 84 %.

isomer ***9a***

$[\alpha]^{16}_D$ = +61.8 (*c* 2.30, DCM)

IR (neat) : *v* = 2943, 2922, 1458, 1446, 1207, 1180, 1093, 1066 cm^{-1}.

^{1}H NMR (300 MHz, Chloroform-*d*) δ 2.14 (t, *J* = 8.4 Hz, 2H), 1.97 – 1.82 (m, 2H), 1.80 (q, *J* = 1.7 Hz, 3H, H_6-Me), 1.78 (s, 1H), 1.76 – 1.66 (m, 2H), 1.56 – 1.47 (m, 1H), 1.48 (q, *J* = 3.1 Hz, 2H), 1.42 – 1.25 (m, 3H), 1.24 – 1.10 (m, 2H, H_4/H_5), 1.20 (s, 3H, H_{10}-Me), 1.07 – 0.97 (m, 2H, H_4/H_5), 1.01 (s, 3H, H_{14}-Meβ), 0.96 – 0.89 (m, 2H), 0.83 (s, 3H, H_{14}-Meα), 0.80 (s, 3H, H_{18}-Me).

^{13}C NMR (75 MHz, $CDCl_3$) δ 124.8 (C_3), 114.8 (C_6), 71.4 (C_9), 62.4 (C_{10}), 42.5 (C_{13}), 41.6 (C_{15}), 38.8 (C_{18}), 34.7 (C_{17}), 33.7 (C_{14}-Me), 33.6 (C_7), 33.0 (C_{14}), 29.3 (C_{11}), 24.9 (C_8), 22.1 (C_{14}-Me), 21.6 (C_{10}-Me), 21.0 (C_6-Me), 18.6 (C_{16}), 17.4 (C_{18}-Me), 17.4 (C_{12}), 3.0 ($C_{4/5}$), 1.5 ($C_{4/5}$).

HRMS (ESI) : calculated for $C_{21}H_{35}O$ $[M+H]^+$: 303.2688, found 303.2690

isomer ***9b***

$[\alpha]^{21}_D$ = +44 (*c* 1.5, $CHCl_3$)

IR (neat) : *v* = 2951, 2930, 1588, 1506, 1458, 1375 cm^{-1}.

^{1}H NMR (300 MHz, Chloroform-*d*) δ 2.44 – 2.20 (m, 2H), 2.05 (ddd, *J* = 12.4, 4.2, 1.7 Hz, 1H), 1.97 (td, *J* = 14.5, 12.4, 5.2 Hz, 1H), 1.85 – 1.75 (m, 1H), 1.78 (p, *J* = 1.7 Hz, 3H, H_6-Me), 1.75 – 1.31 (m, 7H), 1.30 (s, 3H, H_{10}-Me), 1.24 (dd, *J* = 11.3, 4.4 Hz, 1H), 1.16 (dd, *J* = 13.5, 4.2 Hz, 2H), 1.06 (s, 3H, H_{14}-Meβ), 1.04 – 0.99 (m, 2H), 0.96 – 0.88 (m, 2H, H_4/H_5), 0.83 (s, 3H, H_{14}-Meα), 0.87 – 0.81 (m, 2H, H_4/H_5), 0.78 (s, 3H, H_{18}-Me).

^{13}C NMR (75 MHz, $CDCl_3$) δ 124.5 (C_6), 115.0 (C_5), 72.8 (C_9), 64.8 (C_{10}), 54.1 (C_{13}), 41.7 (C_{15}), 38.9 (C_{18}), 37.3 (C_{17}), 36.0 (C_{11}), 34.1 (C_{14}), 34.0 (C_7), 33.4 (C_{14}-Me), 30.0 (C_8), 22.1 (C_{14}-Me), 21.4 (C_{10}-Me), 20.9 (C_6-Me), 20.0 (C_{16}), 17.1 (C_{12}), 16.9 (C_{18}-Me), 3.1 (C_3/C_4), 1.4 (C_3/C_4).

HRMS (ESI) : calculated for $C_{21}H_{35}O$ $[M+H]^+$: 303.2688, found 303.2689

isomer ***9c***

$[\alpha]^{20}_D$ = +15.1 (*c* 1.06, $CHCl_3$)

IR (neat) : *v* = 2950, 2928, 1589, 1506, 1456, 1374 cm^{-1}.

^{1}H NMR (300 MHz, $CDCl_3$) δ 2.95 (dd, *J* = 2.9, 1.4 Hz, 1H, H_{11}), 2.39 (tdp, *J* = 12.5, 4.5, 1.5 Hz, 1H), 2.18 (ddp, *J* = 10.1, 6.5, 1.5 Hz, 1H), 2.10 (dd, *J* = 15.3, 4.3 Hz, 1H), 1.82 (p, *J* = 1.5 Hz, 3H, H_6-Me), 1.81 – 1.60 (m, 3H), 1.52 – 1.30 (m, 4H), 1.36 (s, 3H, H_{10}-Me), 1.23 (d, *J* = 10.0 Hz, 1H), 1.16 – 0.92 (m, 6H), 0.86 (s, 3H, H_{14}-Meβ), 0.89 – 0.77 (m, 2H, H_4/H_5), 0.84 (s, 3H, H_{14}-Meα), 0.75 (s, 3H, H_{18}-Me).

^{13}C NMR (75 MHz, $CDCl_3$) δ δ 124.4 (C_3), 115.9 (C_6), 61.0 (C_{11}), 58.9 (C_{10}), 55.2 (C_9), 46.1 (C_{13}), 42.3 (C_{15}), 39.0 (C_{17}+C_7), 36.1 (C_{18}), 33.2 (C_{14}), 32.8 (C_{14}-Me), 24.2 (C_8), 23.1 (C_{10}-Me), 22.9 (C_{12}), 22.1 (C_{14}-Me), 20.8 (C_6-Me), 18.9 (C_{16}), 14.4 (C_{18}-Me), 3.0 (C_4/C_5), 1.9 (C_4/C_5).

HRMS (ESI) : calculated for $C_{21}H_{35}O$ $[M+H]^+$: 303.2688, found 303.2692

Compound **10a**

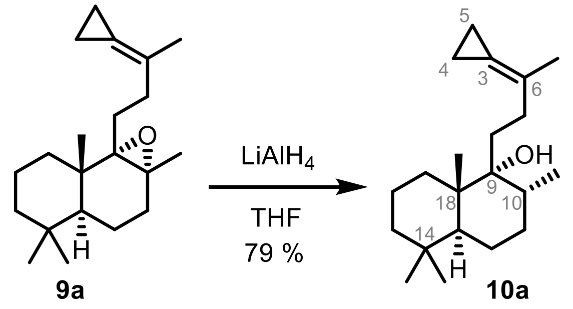

To a solution of **9a** (2.54 g, 8.4 mmol, 1 equiv) in dry THF (34 mL, 0.25 M), $LiAlH_4$ (1.12 g, 29.4 mmol, 3.5 equiv) was added portion-wise. After 3 h stirring at 60 °C, the reaction mixture was cooled down to 0 °C and was quenched carefully with water and an aqueous solution of NaOH (10 M, 100 mL). pH was adjusted to 7 with concentrated HCl. The mixture was extracted with DCM (3 x 200 mL). The combined organic extracts were dried over $MgSO_4$ and concentrated under reduced pressure. The purification of the residue by flash chromatography with silica gel (eluent: petroleum ether/ether, 90:10 to 80:20) afforded **10a** (2.02 g, 79 %) as a yellow oil.

$[\alpha]^{20}_{D}$ = +2.5 (*c* 0.55, $CHCl_3$)

IR (neat) : *v* = 2932, 2865, 1458, 1386, 1369, 910, 756 cm^{-1}.

^{1}H NMR (300 MHz, Chloroform-*d*) δ 2.27 – 2.12 (m, 2H, H_7), 1.83 (p, *J* = 1.7 Hz, 3H, H_6-Me), 1.86 – 1.73 (m, 2H), 1.63 (dd, *J* = 9.8, 7.0 Hz, 1H), 1.58 (dd, *J* = 10.8, 6.0 Hz, 1H), 1.56 – 1.41 (m, 6H), 1.40 – 1.25 (m, 3H), 1.15 (td, *J* = 12.6, 3.5 Hz, 1H), 1.08 – 0.99 (m, 2H, H_4/H_5), 0.99 – 0.93 (m, 2H, H_4/H_5), 0.94 (s, 3H, H_{18}-Me), 0.89 (d, *J* = 6.7 Hz, 3H, H_{10}-Me), 0.87 (s, 3H, H_{14}-Meβ), 0.84 (s, 3H, H_{14}-Meα).

^{13}C NMR (75 MHz, Chloroform-*d*) δ 125.1 (C_6), 115.3 (C_3), 77.3 (C_9), 46.4 (C_{13}), 43.4 (C_{18}), 42.0 (C_{15}), 36.7 (C_{10}), 33.9 (C_{14}-Meβ), 33.5 (2C, C_7, C_{14}), 32.7 (C_8), 32.1 (C_{17}), 31.5 (C_{11}), 22.2(C_{14}-Meα), 21.9 (C_{12}), 20.8 (C_6-Me), 18.9 (C_{16}), 16.5 (C_{10}-Me/C_{18}-Me), 16.4 (C_{10}-Me/C_{18}-Me), 3.0 (C_4/C_5), 1.8 (C_4/C_5).

HRMS (ESI) : calculated for $C_{21}H_{35}$ $[M-H_2O+H]^+$: 287.2739, found 287.2738.

Compound **10b**

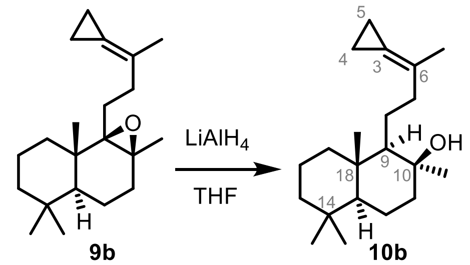

To a solution of **9b** (1.27 g, 4.2 mmol, 1 equiv) in dry THF (17 mL, 0.25 M), $LiAlH_4$ (0.56 g, 14.7 mmol, 3.5 equiv) was added portion-wise. After 3 h stirring at 60 °C, the reaction mixture was cooled down to 0 °C and was quenched carefully with water and an aqueous solution of NaOH (10 M, 100 mL). pH was adjusted to 7 with concentrated HCl. The mixture was extracted with DCM (3 x 200 mL). The combined organic extracts were dried over $MgSO_4$ and concentrated under reduced pressure. The purification of the residue by flash chromatography with silica gel (eluent: petroleum ether/ether, 90:10 to 80:20) afforded **10b** (0.86 g, 62%) as a yellow oil.

$[\alpha]^{20}_D$ = +25 (*c* 1.5, $CHCl_3$)

IR (neat) : *v* = 2922, 2866, 2843, 1458, 1387, 1369, 910, 756 cm^{-1}.

^{1}H NMR (300 MHz, Chloroform-*d*) δ 2.31 – 2.10 (m, 2H, H7), 1.85 (p, *J* = 1.7 Hz, 3H, H_6-Me), 1.81 – 1.69 (m, 2H, H_6-Me), 1.61 (tt, *J* = 12.7, 3.9 Hz, 1H, H11a), 1.61 – 1.49 (m, 3H, H12a, H12b, H17), 1.49 – 1.36 (m, 3H), 1.19 (s, 3H, H_{10}-Me), 1.22 – 1.10 (m, 2H), 1.09 – 1.02 (m, 2H, H_4/H_5), 1.01 – 0.95 (m, 2H, H_4/H_5), 0.98 (s, 3H, H_{18}-Me), 0.90 (s, 3H, H_{14}-Meβ), 0.73 – 0.83 (m, 2H), 0.85 (s, 3H, H_{14}-Meα).

^{13}C NMR (75 MHz, Chloroform-*d*) δ 125.1 (C_6), 115.2 (C_3), 73.4 (C_{10}), 59.2 (C_9), 56.2 (C_{13}), 42.4 (C_{11}), 42.3 (C_{15}), 40.7 (C_{17}), 39.3 (C_7), 39.1 (C_{18}), 33.6 (C_{14}-Meβ), 33.4 (C_{14}), 30.8 (C_{10}-Me), 23.9 (C_8), 21.8 (C_6-Me), 20.8 (C_{14}-Meα), 18.5 (C_{12} or C_{16}), 18.4 (C_{12} or C_{16}), 15.3 (C_{18}-Me), 2.9 ($C_{4/5}$), 1.8 ($C_{4/5}$).

HRMS (ESI) : calculated for $C_{21}H_{35}$ $[M-H_2O+H]^+$: 287.2739, found 287.2739

Compound **11**

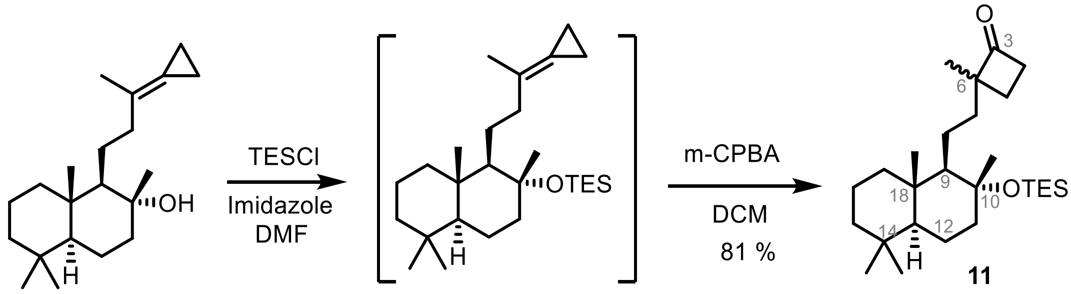

To a solution of **7** (5.5 g, 18 mmol, 1 equiv) and imidazole (3.68 g, 54 mmol, 3 equiv) in DMF (18 mL) was added triethylsilyl chloride (6.3 mL, 36 mmol, 2 equiv), and the mixture was stirred at room temperature overnight. After the completion of the reaction, the mixture was poured into 500 mL of diethyl ether and washed with 10% citric acid aqueous solution (50 mL) followed by water (50 mL). After evaporation of the organic layer, DCM (36 mL) was added. The new resulting mixture was cooled at 0 °C and was added *m*-CPBA (4.6 g, 19.8 mmol, 1.1 equiv, 70-77% in water) in 5 portions over 15 min. The mixture was warmed to rt and stirred for 1 h at this temperature. The reaction mixture was quenched with a $Na_2S_2O_3$ solution, and the mixture was stirred vigorously for 20 min. The organic layer was extracted with diethyl ether twice (200 mL), and the combined organic layers were washed with a saturated $NaHCO_3$ solution (2x50 mL), dried over $MgSO_4$, filtered, and concentrated on rotavapor. The residue was then purified on a silica gel chromatography (Petroleum ether: ether, 96:4) to afford **11** (6.38 g, 81%, 50:50 mixture of diastereomers) as a yellow oil

IR (neat) : *ν* = 2950, 2872, 1777, 1456, 1115, 1048, 741 cm^{-1}.

^{1}H NMR (300 MHz, $CDCl_3$, 50:50 mixture of diastereomers) δ 3.01 – 2.89 (m, 2H, H_4), 2.01 – 1.80 (m, 2H), 1.77 – 1.29 (m, 10H), 1.27 – 1.17 (m, 1H), 1.16 (s, 1.5H, H_6-Me, *1 diastereomer*), 1.16 (s, 1.5H, H_6-Me, *1 diastereomer*), 1.13 (s, 3H, H_{10}-Me), 1.13 – 1.04 (m, 1H), 1.04 – 1.00 (m, 1H), 0.93 (t, *J* = 7.9 Hz, 4.5H, C$\underline{H_3}$$CH_2$Si, *1 diastereomer*), 0.92 (t, *J* = 7.9 Hz, 4.5H, C$\underline{H_3}$$CH_2$Si, *1 diastereomer*), 0.87 – 0.85 (m, 1H), 0.84 (s, 3H, H_{14}-Meβ), 0.76 (s, 6H, H_{18}-Me & H_{14}-Meα), 0.55 (q, *J* = 7.9 Hz, 3H, $CH_3\underline{CH_2}$Si, *1 diastereomer*), 0.54 (q, *J* = 7.9 Hz, 3H, $CH_3\underline{CH_2}$Si, *1 diastereomer*).

^{13}C NMR (75 MHz, $CDCl_3$, 50:50 mixture of diastereomers) δ [216.3, 216.2] (C_3), [77.6, 77.5] (C_{10}), [65.1, 64.9] (C_6), [62.7, 62.7] (C_9), 56.2 (C_{13}), 44.4 (C_{11}), [42.4, 42.0] (C_4), 42.1 (C_{15}), [40.0, 39.8] (C_7), 39.9 (C_{17}), [39.3, 39.2] (C_{18}), 33.5 (C_{14}-Me), 33.3 (C_{14}), 25.0 (C_{10}-Me), [24.4, 23.8] (C_5), 21.6 (C_{14}-Me), [21.0, 20.5] (C_6-Me), 20.70 (C_{12}), [20.1, 19.7] (C_8), 18.6 (C_{16}), 15.8 (C_{18}-Me), 7.4 (C$\underline{H_3}$$CH_2$Si), 7.2($CH_3\underline{CH_2}$Si).

HRMS (ESI) : calculated for $C_{27}H_{50}O_2SiNa$ $[M+Na]^+$: 457.3478, found 457.3476

Compound **12**

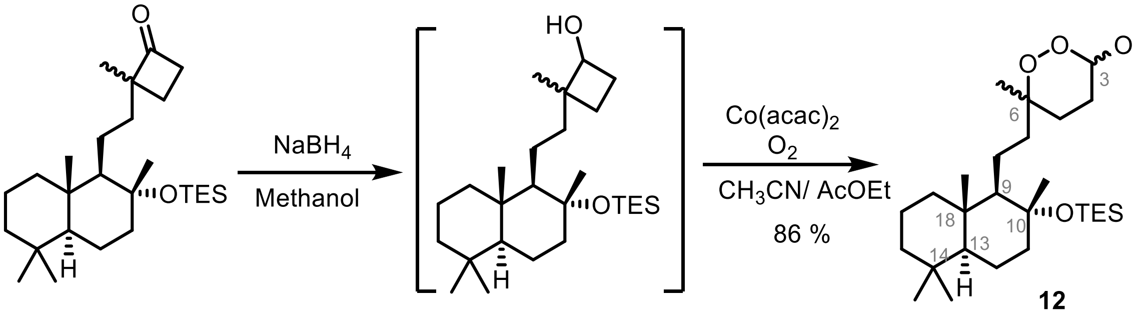

To a solution of **11** (6.09 g, 14 mmol, 1 equiv) in methanol (42 mL) at 0 °C was added $NaBH_4$ (530 mg, 14 mmol, 1 equiv) in 3 portions over 10 minutes. The reaction was then stirred at rt for 1 h and filtered on a pad of silica gel with (3x100 mL of ethyl acetate). The filtrate was concentrated under a vacuum. Acetonitrile (140 mL), ethylacetate (28 mL) were added to the crude followed by the addition of $Co(acac)_2$ (180 mg, 0.7 mmol, 0.05 equiv,). The brownish reaction mixture was then lightly bubbled with O_2 for 5 min, and the oxygen atmosphere was maintained with a rubber balloon. The reaction was then heated to 40 °C with an oil bath, and the color turned slowly to intense green. The reaction was monitored by TLC, and after 4 h of reaction under these conditions, the reaction mixture was concentrated on a rotavapor with gentle heating (no more than 30 °C). The residue was purified on a silica gel chromatography (Petroleum ether: ether, 8:2) to afford **12** (5.64 g, 86 %) as a colorless oil.

IR (neat) : *v* = 3400, 2934, 2874, 1777, 1456, 1083, 741 cm^{-1}.

^{1}H NMR (300 MHz, Chloroform-*d*) δ 5.23 (m, 1H, H_3), 3.28 – 3.15 (m, 1H, OH), 2.08 – 1.92 (m, 1H), 1.92 – 1.75 (m, 2H), 1.74 – 1.36 (m, 11H), 1.33 (s, 0.75H, *1 diastereomer,* H_6-Me), 1.32 (s, 0.75H, *1 diastereomer,* H_6-Me), 1.20 (s, 0.75H, *1 diastereomer,* H_6-Me), 1.18 (s, 0.75H, *1 diastereomer,* H_6-Me), 1.15 (s, 3H, H_{10}-Me), 1.15 – 1.11 (m, 1H), 1.05 (dt, *J* = 10.4, 2.9 Hz, 1H), 0.94 (t, *J* = 7.8 Hz, C<u>H</u>$_3$$CH_2$Si, 9H), 0.91 – 0.87 (m, 1H), 0.86 (s, 3H, H_{14}-Meβ), 0.77 (s, 6H, H_{18}-Me & H_{14}-Meα), 0.57 (q, *J* = 7.5 Hz, 3H, CH_3<u>CH</u>$_2$Si, *2 diastereomers*), 0.56 (q, *J* = 7.5 Hz, 3H, CH_3<u>CH</u>$_2$Si, *2 diastereomers*).

^{13}C NMR (75 MHz, $CDCl_3$) δ [96.7, 96.5, 96.3] (C_3), [81.15, 81.09] (C_6), [77.7, 77.7, 77.5, 77.5] (C_{10}), 62.7 (C_9), [56.3, 56.2] (C_{13}), [44.6, 44.5] (C_{11}), [42.6, 42.1, 41.1, 40.7] (C_7), [42.3, 42.2] (C_{15}), [40.1, 40.0, 39.8] (C_{17}), [39.4, 39.3] (C_{18}), 33.5 (C_{14}-Me), 33.3 (C_{14}), [30.2, 29.5, 29.4, 28.0] (C_5), [26.1, 25.8, 25.8, 25.6] (C_4), 25.0 (C_{10}-Me), [22.4, 21.9, 21.2, 20.8] (C_6-Me), 21.6 (C_{14}-Me), 20.8 (C_{12}), [19.8, 19.6, 19.1, 19.0] (C_8), [18.6, 18.6] (C_{16}), [15.8, 15.8] (C_{18}-Me), 7.4 (C<u>H</u>$_3$$CH_2$Si), [7.3, 7.2] ($CH_3$<u>CH</u>$_2$Si).

HRMS (ESI) : calculated for $C_{27}H_{52}O_4SiNa$ $[M+Na]^+$: 491.3533, found 491.3539

Compound **13**

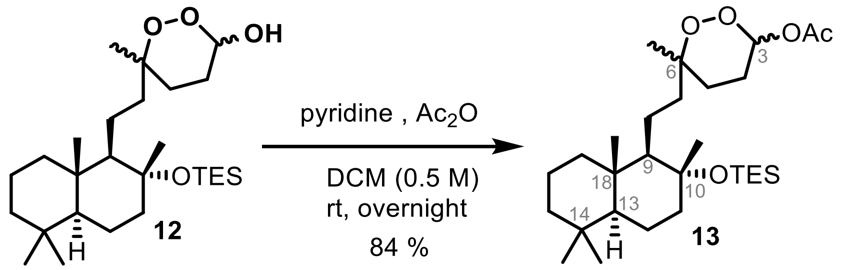

To a solution of **12** (5.63 g, 12 mmol, 1 equiv) in DCM (24 mL), were added AC_2O (6.8 mL, 72 mmol, 6 equiv) and pyridine (3.87 mL, 48 mmol, 4 equiv). The reaction mixture was stirred overnight at rt and was poured into an Erlenmeyer containing a saturated solution of $NaHCO_3$. The mixture was stirred vigorously for 30 min at rt, and the aqueous layer was extracted thrice with 100 mL of dichloromethane. The combined organic phases were washed with a citric aqueous solution (2x30 mL), dried over $MgSO_4$, filtered, and concentrated on a rotavapor under vacuum with gentle heating (no more than 30 °C). The crude was purified on a silica gel to afford **13** (5.13 g, 84%) as a colorless oil.

IR (neat) : *v* = 2972, 1752, 1377, 1248, 1066, 721 cm^{-1}.

^{1}H NMR (300 MHz, Chloroform-*d, about 0.25:0.25:0.25:0.25 mixture of diastereomers*) δ 6.13 (t, *J* = 5.0 Hz, 0.5H, H_3, *2 diastereomers*), 6.11 (t, *J* = 5.0 Hz, 1H, H_3, *2 diastereomers*), 2.04 (s, 3H, OAc), 2.03 – 1.90 (m, 1H), 1.86 – 1.72 (m, 2H), 1.72 – 1.29 (m, 12H), 1.28 (s, 1.5H, H_6-Me, 2 *diastereomers*), 1.23 – 1.10 (m, 2H), 1.09 (s, 3H, H_{10}-Me), 1.08 (s, 0.75H, H_6-Me, *1 diastereomer*), 1.06 (s, 0.75H, H_6-Me, *1 diastereomer*), 1.05 – 0.93 (m, 2H), 0.92 – 0.84 (4t, *J* = 7.9 Hz, C<u>H</u>$_3$CH$_2$Si, *4 diastereomers*), 0.85 – 0.78 (m, 1H), 0.79 (s, 3H, H_{14}-Meβ), 0.70 (s, 6H, H_{18}-Me & H_{14}-Meα), 0.57 – 0.43 (4q, *J* = 7.9 Hz, CH$_3$<u>CH$_2$</u>Si, *4 diastereomers*).

^{13}C NMR (75 MHz, $CDCl_3$) δ [170.1, 170.1] (O-C=O), [95.2, 95.1, 95.0, 94.9] (C_3), [81.8, 81.6, 81.6, 81.4] (C_6), [77.8, 77.7, 77.5, 77.4] (C_{10}), [62.8, 62.7, 62.7, 62.6] (C_9), [56.3, 56.3, 56.2] (C_{13}), [44.6, 44.5] (C_{11}), [42.5, 42.0, 41.1, 40.6,] (C_7), [42.3, 42.2], (C_{15}), [40.1, 40.0, 39.8] (C_{17}), [39.4, 39.3] (C_{18}), 33.5 (C_{14}-Me), 33.3 (C_{14}), [29.6, 28.6, 28.4, 28.1] (C_5), [25.1, 25.0, 25.0] (C_{10}-Me), [23.2, 23.0] (C_4), [22.2, 21.9, 21.8, 21.4] (C_6-Me), 21.6 (C_{14}-Me), 21.3 (OAc), 20.8 (C_{12}), [19.9, 19.5, 18.9, 18.9] (C_8), [18.7, 18.6] (C_{16}), 15.8 (C_{18}-Me), 7.4 (C<u>H$_3$</u>CH$_2$Si), [7.3, 7.2] (CH$_3$<u>CH$_2$</u>Si).

HRMS (ESI) : calculated for $C_{29}H_{54}O_5SiNa$ $[M+Na]^+$: 533.3638, found 533.3645

Compounds **15a-h**

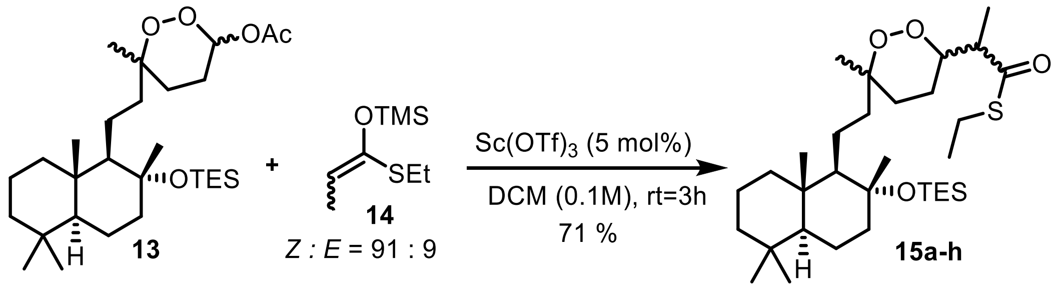

To a solution of **13** (1.02 g, 2 mmol, 1 equiv) and silylketenethioketal **14**[5] (1.14 g, 6 mmol, 3 equiv) in dry DCM (20 mL, 0.1 M) was added $Sc(OTf)_3$ (49 mg, 0.1 mmol, 0.05 equiv). The reaction mixture was stirred at rt for 3 h and was poured into a saturated aqueous solution of $NaHCO_3$. The aqueous phase was extracted with DCM (3x100 mL), and the combined organic layers were washed with brine. The organic layer was dried over $MgSO_4$ and concentrated on a rotavapor with gentle heating (no more than 30 °C). The residue was purified on a silica gel (Petroleum ether /toluene: from 100/00 to 50/50) to afford three fractions: F1 (90 mg, 8 %) as a colorless oil, F2 (360 mg, 32 %) as a colorless oil, and F3 (350 mg, 31 %) as a colorless oil (71% global yield). The description of each fraction is gathered below. The selectivity of the reaction was about 4:1 in favor of *cis*-1,2-dioxanes and 1:1 for the 2,3-*anti*/*syn* relationship. Rf are 0.58, 0.40, 0.21 for Fractions F1, F2, F3 respectively in Petroleum ether: toluene, 50:50.

Fraction F1= ***15a:15b*** *(45:55 mixture of diastereomers)*

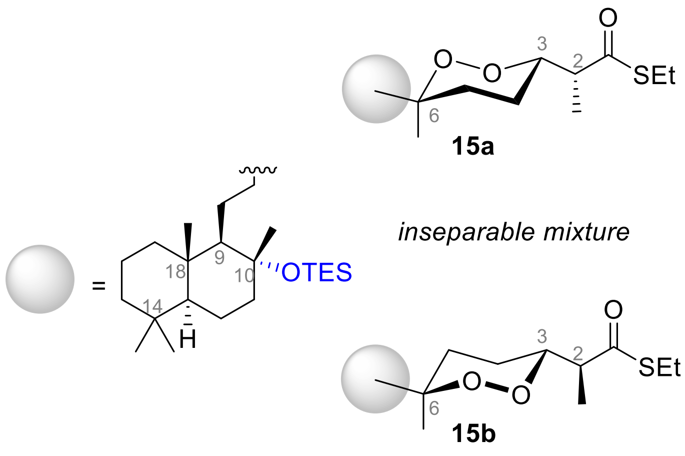

IR (neat) : v = 2933, 2875, 1683, 1456, 1376 cm^{-1}.

^{1}H NMR (300 MHz, $CDCl_3$, *about 1:1 mixture of diastereomers*) δ 4.10 (dt, J = 8.2, 5.9 Hz, 1H, H_3), 2.87 (q, J = 7.2 Hz, 2H, $CH_3\underline{CH_2}S$), 2.83 – 2.68 (m, 1H, H_2), 2.06 – 1.82 (m, 1H), 1.86 (dt, J = 12.0, 3.1 Hz, 1H), 1.79 – 1.52 (m, 6H), 1.53 – 1.29 (m, 4H), 1.30 – 1.15 (m, 9H), 1.25 (d, J = 8.9 Hz, 3H, H2-Me), 1.24 (t, J = 7.2 Hz, 3H, $C\underline{H_3}CH_2S$), 1.13 (s, 3H, H_{10}), 0.93 (t, J = 7.9 Hz, 9H, $C\underline{H_3}CH_2Si$), 0.92 – 0.81 (m, 2H), 0.84 (s, 3H H_{14}-Meβ), 0.75 (s, 6H, H_{18}-Me & H_{14}-Meα), 0.55 (q, J = 7.9 Hz, 6H, $CH_3C\underline{H_2}Si$).

^{13}C NMR (75 MHz, $CDCl_3$, ***15a:15b*** *= 55:45 mixture of diastereomers*) δ 201.0 (C_1), [81.8*, 81.6] (C_6), [80.9*,80.9] (C_3), [77.6, 77.8*] (C_{10}), [62.6*, 62.6] (C_9), [56.2, 56.2*] (C_{13}), [51.8*, 51.7] (C_2), 44.4

(C_{11}), 43.8 (br, C_7), [42.2, 42.1*] (C_{15}), [39.9*, 39.8] (C_{17}), 39.3 (C_{18}), 33.5 (C_{14}-Me), 33.3 (C_{14}), [32.6, 31.5*] (br, C_5), 29.8 (C_6-Me), 25.0 (C_{10}-Me], [24.0*, 23.8] (br, C_4), 23.4 ($CH_3\underline{CH_2}S$), 21.6 (C_{14}-Me), 20.7 (C_{12}), [19.9, 19.0] (C_8), 18.6 (C_{16}), 15.8 (C_{18}-Me), [14.9*, 14.8] (C_2-Me), 14.8 ($C\underline{H_3}CH_2S$), 7.4 ($C\underline{H_3}CH_2Si$), 7.2 ($CH_3\underline{CH_2}Si$). *= minor diastereomer = **15b**

HRMS (ESI) : calculated for $C_{32}H_{60}O_4SSiNa$ $[M+Na]^+$: 591.3879, found 591.3879

Fraction F2 = ***15c:15d:15e:15f*** *≈ 0.4:0.4:0.1:0.1*

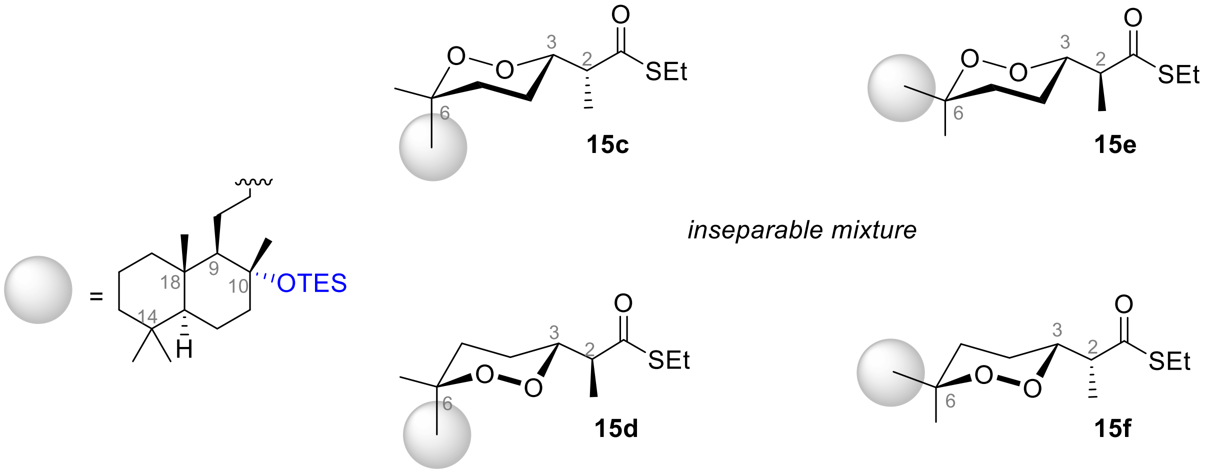

15c **15e**

inseparable mixture

15d **15f**

IR (neat) : *v* = 2934, 2873, 1684, 1455, 1377 cm^{-1}.

^{1}H NMR (300 MHz, Chloroform-*d, about 0.4:0.4:0.1:0.1 mixture of diatereomers*) δ 4.32 – 4.20 (m, 0.2H, H_3, *2 minor trans isomers*), 4.16 – 4.01 (m, 0.8H, H_3, *2 major cis isomers*), 2.86 (q, *J* = 7.4 Hz, 2H, $CH_3\underline{CH_2}S$), 2.97 – 2.65 (m, 1H, H_2), 1.92 – 1.32 (m, 14H), 1.32 – 1.17 (m, 8H), 1.16 – 1.13 (m, 1H), 1.14 (s, 4.8H), 1.12 (s, 1.2H), 1.06 (t, *J* = 3.5 Hz, 1H), 0.94 (t, *J* = 7.9 Hz, 7.2H, $CH_3C\underline{H_2}Si$, *3 diastereomers*), 0.92 (t, *J* = 7.9 Hz, 1.8H, $\underline{CH_3}CH_2Si$, *1 minor cis isomer*), 0.99 – 0.87 (m, 2H), 0.85 (s, 2.4H, H_{14}-Meβ, *2 major cis isomers*), 0.84 (s, 0.6H, H_{14}-Meβ, *2 minor trans isomers*), 0.77 (s, 2.4H, , H_{18}-Me & H_{14}-Meα, *1 major cis isomer*), 0.77 (s, 2.4H H_{18}-Me & H_{14}-Meα, *1 major cis isomer*), 0.75 (s, 0.6H, H_{18}-Me & H_{14}-Meα, *1 minor trans isomer*), 0.74 (s, 0.6H, H_{18}-Me & H_{14}-Meα, *1 minor trans isomer*), 0.57 (q, *J* = 7.9 Hz, 2.4H, $CH_3C\underline{H_2}Si$, *1 major cis isomer*), 0.56 (q, *J* = 7.9 Hz, 2.4H, $CH_3C\underline{H_2}Si$, *1 major cis isomers*), 0.51 (d, *J* = 6.2 Hz, 1.2H, $CH_3C\underline{H_2}Si$, *2 minor trans isomers*).

^{13}C NMR (75 MHz, $CDCl_3$, *about 0.4:0.4:0.1:0.1 mixture of diatereomers*) δ [201.3, 201.1, 201.1*, 201.0*] (C_1), [81.8*, 81.7*, 81.1] (C_6), [81.1*, 80.9, 80.7] (C_3), [77.6, 77.5*, 77.4] (C_{10}), [62.7, 62.6, 62.6*] (C_9), [56.3, 56.2] (C_{13}), [51.5, 51.2*, 51.2*, 51.1] (C_2], [44.5] (C_{11}), [44.1*, 44.0*, 40.5, 39.4] (br, C_7), [42.2, 42.1] (C_{15}), [40.0, 39.9*, 39.8*, 39.8] (C_{17}), 39.3 (C_{18}), 33.5 (C_{14}-Me), 33.3 (C_{14}), [32.1, 31.9*, 30.9, 30.4*] (br, C_5), 25.0 (C_{10}-Me), [23.7, 23.5, 23.1*, 23.0*] (C_4), [23.3, 23.1, 20.8*, 20.6*] (br, C_6-Me), 23.4 ($CH_3\underline{CH_2}S$), 21.6 (C_{14}-Me), 20.7 (C_{12}), [19.5, 19.2, 19.0*, 18.9*] (C_8), [18.6, 18.5*] (C_{16}), 15.8 (C_{18}-Me), [15.1, 14.7, 13.5* 13.5*] (C_2-Me), 14.8 ($C\underline{H_3}CH_2S$), 7.5 ($C\underline{H_3}CH_2Si$) , [7.3, 7.2] ($CH_3\underline{CH_2}Si$). *= minor diastereomers when identifiable.

HRMS (ESI) : calculated for $C_{32}H_{60}O_4SSiNa$ $[M+Na]^+$: 591.3879, found 591.3884

Fraction F3= ***15g:15h*** *(55:45 mixture of diastereomers)*

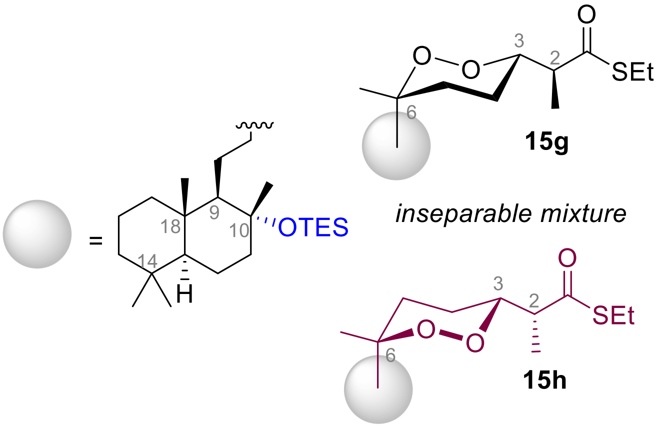

IR (neat) : *ν* = 2933, 2873, 1687, 1456,1376, 964 cm^{-1}.

^{1}H NMR (300 MHz, $CDCl_3$, *about 1:1 mixture of diastereomers*) δ 4.31 – 4.19 (m, 1H, H_3), 3.00 – 2.71 (m, 3H, $CH_3\underline{CH_2}S$ +H_2), 2.03 – 1.31 (m, 17H), 1.23 (t, *J* = 7.5 Hz, 1.5H, $C\underline{H_3}CH_2Si$, *1 diastereomer*), 1.23 (t, *J* = 7.5 Hz, 1.5H, $C\underline{H_3}CH_2Si$, *1 diastereomer*), 1.28 – 1.16 (m, 1H), 1.16 -1.02 (m, 2H), 1.13 (s, 3H, H_{10}-Me), 1.13 (s, 1.5H, H_6-Me, *1 diastereomer*), 1.12 (d, *J* = 5.6 Hz, 1.5H, H_2-Me, *1 diastereomer*), 1.12 (d, *J* = 5.6 Hz, 1.5H, H_2-Me, *1 diastereomer*), 1.09 (s, 1.5H, H_6-Me, *1 diastereomer*), 0.93 (t, *J* = 7.8, 9H, $\underline{CH_3}CH_2Si$), 0.92 – 0.87 (m, 2H), 0.85 (s, 3H, H_{14}-Meβ), 0.77 (s, 3H, H_{18}-Me and/or H_{14}-Meα), 0.76 (s, 3H, H_{18}-Me and/or H_{14}-Meα), 0.55 (q, *J* = 7.9 Hz, 6H, $CH_3C\underline{H_2}Si$).

^{13}C NMR (75 MHz, $CDCl_3$, ***15g:15h*** *= 45:55 mixture of diastereomers*) δ [201.2*, 201.0] (C_1), [81.3*, 81.1] (C_6), [81.0*, 80.3] (C_3), [77.6*, 77.3] (C_{10}), 62.6 (C_9), [56.3*, 56.1] (C_{13}), [51.0*, 50.5] (C_2), [44.5*, 44.5] (C_{11}), [42.3*, 42.1] (C_{15}), [40.0 , 39.7*] (C_{17}), 39.3 (C_{18}), [39.2, 38.5*] (br, C_7), [33.5*, 33.5] (C_{14}-Me), [33.3*, 33.3] (C_{14}), [33.0*, 31.0] (br, C_5), 25.0 (C_{10}-Me), [24.1*, 23.7] (C_6-Me), 23.4 ($CH_3\underline{CH_2}S$), 22.7 (C_4), 21.6 (C_{14}-Me), [20.8, 20.7*] (C_{12}), [20.0*, 19.1] (C_8), [18.7, 18.6*] (C_{16}), [15.8*, 15.8] (C_{18}), [14.7, 14.6*] ($C\underline{H_3}CH_2S$), [13.6*, 13.2] (C_2-Me), [7.5, 7.4] ($C\underline{H_3}CH_2Si$), [7.3, 7.2] ($CH_3\underline{CH_2}Si$). *= minor diastereomer = **15h**

HRMS (ESI) : calculated for $C_{32}H_{60}O_4SSiNa$ $[M+Na]^+$: 591.3879, found 591.3883

Compounds **16a-b**

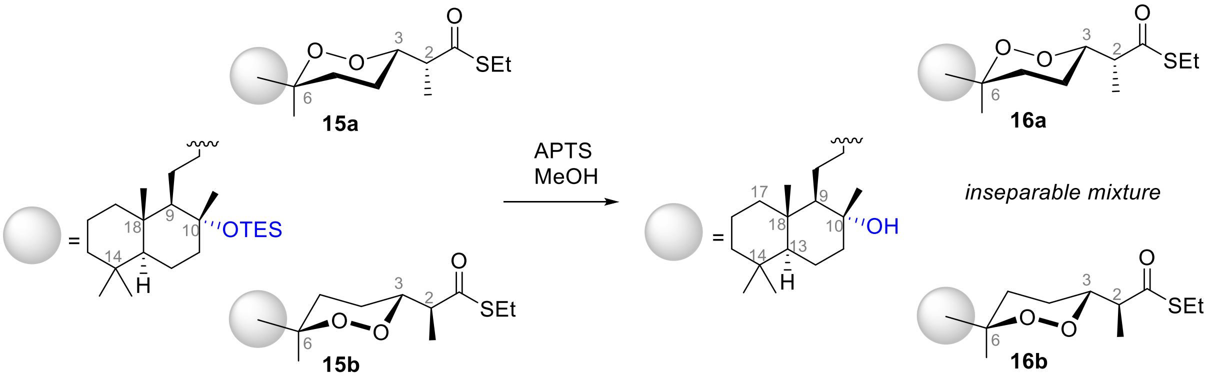

To a solution of the fraction F1 containing **15a-b** (75.7 mg, 0.131 mmol, 1 equiv) in methanol (5 mL) was added PTSA (22.6 mg, 0.131 mmol, 1 equiv). The reaction mixture was stirred at rt for 10 min and was poured into a saturated aqueous solution of $NaHCO_3$. The aqueous phase was extracted with DCM (3x20mL), and the combined organic layers were washed with brine, dried over $MgSO_4$, and concentrated on a rotavapor with gentle heating (no more than 30 °C). The residue was purified on a silica gel column (petroleum ether: Et_2O, 90:10) to afford **16a-b** as an inseparable mixture (56.6 mg, 95 %, **16a**:**16b** = 55:45) and as colorless oil. Rf = 0.37 in petroleum Ether: Et_2O, 2:1

IR (neat) : *v* = 3397, 2931, 2870, 1683, 1455, 1387, 966 cm^{-1}

^{1}H NMR (400 MHz, Chloroform-*d, about 1:1 mixture of diastereomers*) δ 4.16 – 4.05 (m, 1H, H_3), 2.87 (q, *J* = 7.4 Hz, 2H, $CH_3\underline{CH_2}S$), 2.79 (m, 1H, H_2), 1.88 – 1.79 (m, 1H), 1.79 – 1.50 (m, 8H), 1.50 – 1.32 (m, 5H), 1.31 – 1.19 (m, 3H), 1.29 (s, 1.5H, H_6-Me, *1 diastereomer*), 1.28 (s, 1.5H, H_6-Me, *1 diastereomer*), 1.27 (d, *J* = 5.9 Hz, 1.5H, H_2-Me, *1 diastereomer*), 1.25 (d, *J* = 5.9 Hz, 1.5H, H_2-Me, *1 diastereomer*), 1.24 (t, *J* = 7.4 Hz, 3H, $C\underline{H_3}CH_2S$), 1.14 (s, 1.5H, H_{10}-Me, *1 diastereomer*), 1.14 (s, 1.5H, H_{10}-Me, *1 diastereomer*), 1.18 – 1.08 (m, 1H), 0.99 (dt, *J* = 17.4, 4.0 Hz, 1H), 0.94 – 0.87 (m, 2H), 0.85 (s, 3H, H_{14}-Meβ), 0.77 (s, 6H, H_{18}-Me & H_{14}-Meα).

^{13}C NMR (101 MHz, $CDCl_3$, *55:45 mixture of diastereomers*) δ [201.0, 200.9*] (C_1), [81.6, 81.5*] (C_3), [80.8, 80.8*] (C_6), [74.6, 74.4*] (C_{10}), [62.3, 62.3*] (C_9), 56.3 (C_{13}), [51.7, 51.5*] (C_2), [44.9, 44.2*] (C_{11}), [43.4, 43.0*] (br, C_7), 42.2 (C_{15}), [39.9*, 39.8] (C_{17}), [39.4, 39.4*] (C_{18}), 33.5 (C_{14}-Me), 33.4 (C_{14}-Me), [32.1, 31.9*] (br, C_5), [24.3*, 24.2] (C_{10}-Me), [23.7, 23.6*] (br, C_4), 23.4 ($CH_3\underline{CH_2}S$), 21.6 (C_{14}-Me), [21.2*, 20.9] (br, C_6-Me), [20.7, 20.6*] (C_{12}), 18.6 (C_{16}), [18.6, 18.4*] (C_8), [15.6, 15.6*] (C_{18}-Me), [14.9*, 14.9] (C_2-Me), 14.8 ($C\underline{H_3}CH_2S$). *= minor diastereomer.

HRMS (ESI) : calculated for $C_{26}H_{46}O_4SNa$ $[M+Na]^+$: 477.3015, found 477.3014.

Compounds **16d**, **16c**, and **16e-f**

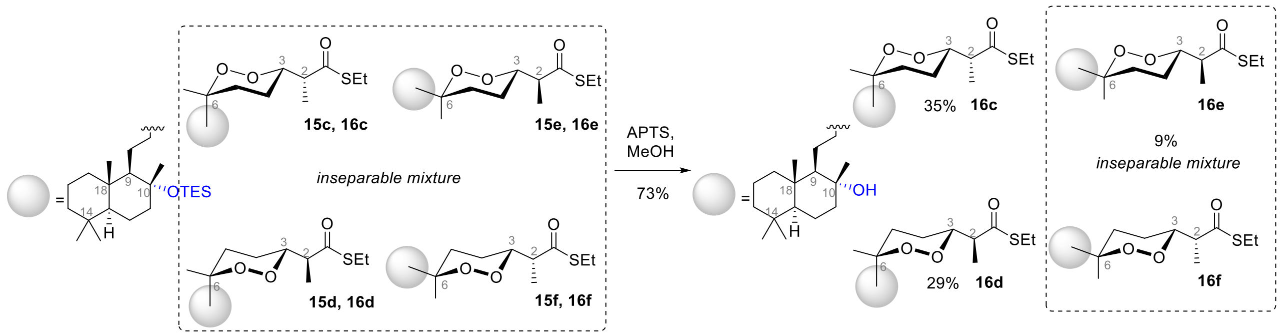

To a solution of the fraction F2 containing **15c-f** (170 mg, 0.3 mmol, 1 equiv) in methanol (3 mL) was added PTSA (52 mg, 0.3 mmol, 1 equiv). The reaction mixture was stirred at rt for 10 min and was poured into a saturated aqueous solution of $NaHCO_3$. The aqueous phase was extracted with DCM (3x20mL), and the combined organic layers were washed with brine, dried over $MgSO_4$, and concentrated on a rotavapor with gentle heating (no more than 30 °C). The residue was purified on a silica gel (petroleum ether: Et_2O, 80/20 to 60/40) to afford, by order of elution, **16d** (39 mg, 29 %) as a colorless oil, **16c** (48 mg, 35%) as white solid, and **16e-f** (12 mg, 9%) as an inseparable mixture and colorless oil. The global yield was 73%. Rf = 0.39, 0.29 and 0.21 in petroleum Ether: Et_2O, 2:1 for **16d, 16c** and **16e-f** mixture respectively.

Compound ***16d***

[α]$^{24}_{D}$ = +87 (c 1.2, $CHCl_3$)

IR (neat) : *v* = 3397, 2931, 2870, 1683, 1455, 1387, 966 cm^{-1}

^{1}H NMR (300 MHz, $CDCl_3$) δ 4.13 (td, *J* = 8.3, 3.8 Hz, 1H, H_3), 2.85 (q, *J* = 7.4 Hz, 2H, $CH_3\underline{CH_2}S$), 2.74 (p, *J* = 7.1 Hz, 1H, H_2), 2.05 (t, *J* = 10.9 Hz, 1H), 1.90 (brs, 1H, OH), 1.84 (dt, *J* = 12.1, 3.1 Hz, 1H), 1.75 – 1.52 (m, 8H), 1.48 – 1.33 (m, 5H), 1.32 – 1.24 (m, 1H), 1.24 (d, *J* = 6.8 Hz, 3H, H_2-Me), 1.23 (t, *J* = 7.4 Hz, 3H, $C\underline{H_3}CH_2S$), 1.18 (s, 3H, H_{10}-Me), 1.12 (s, 3H, H_6-Me), 1.16 – 1.07 (m, 1H), 0.93 (dd, *J* = 12.7, 4.5 Hz, 1H), 0.90 (dd, *J* = 11.9, 2.2 Hz, 1H), 0.86 (s, 3H, H_{14}-Meβ), 0.78 (s, 6H, H_{18}-Me & H_{14}-Meα).

^{13}C NMR (75 MHz, $CDCl_3$) δ 200.9 (C_1), 81.6 (C_3), 80.5 (C_6), 74.4 (C_{10}), 62.1 (C_9), 56.3 (C_{13}), 51.6 (C_2), 43.3 (C_{11}), 42.2 (C_{15}), 39.8 (C_{17}), 39.1 (C_{18}), 38.5 (br, C_7), 33.5 (C_{14}-Me), 33.4 (C_{14}), 32.7 (br, C_5), 24.5 (C_{10}-Me), 23.8 (br, C_6-Me), 23.5 ($CH_3\underline{CH_2}S$ + C_4), 21.6 (C_{14}-Me), 20.5 (C_{12}), 18.6 (C_{16}), 18.2 (C_8), 15.7 (C_{18}-Me), 14.9 (C_2-Me), 14.7 ($C\underline{H_3}CH_2S$).

HRMS (ESI): calculated for $C_{26}H_{46}O_4SNa$ $[M+Na]^+$: 477.3015, found 477.3014

Compound ***16c***

Mp = 86-87 °C

[α]$^{24}_{D}$ = –83 (c 0.95, $CHCl_3$)

IR (neat) : *v* = 3400, 2930, 2849, 1683, 1455, 966 cm^{-1}.

^{1}H NMR (300 MHz, $CDCl_3$) δ 4.11 (td, *J* = 7.9, 4.6 Hz, 1H, H_3), 2.85 (q, *J* = 7.4 Hz, 2H, $CH_3\underline{CH_2}S$), 2.75 (p, *J* = 7.5 Hz, 1H, H_2), 1.92 – 1.78 (m, 2H), 1.78 – 1.47 (m, 8H), 1.46 – 1.27 (m, 5H), 1.27 – 1.19 (m, 8H, contains H_2-Me and $C\underline{H_3}CH_2S$), 1.16 (s, 1H), 1.14 (s, 6H, H_6-Me & H_{10}-Me), 1.00 (t, *J* = 3.9 Hz, 1H), 0.94 – 0.86 (m, 2H), 0.85 (s, 3H, H_{14}-Meβ), 0.78 (s, 3H, H_{18}-Me or H_{14}-Meα), 0.77 (s, 3H, H_{18}-Me or H_{14}-Meα).

^{13}C NMR (75 MHz, $CDCl_3$) δ 201.0 (C_1), 81.2 (C_6), 80.6 (C_3), 74.6 (C_{10}), 62.4 (C_9), 56.3 (C_{13}), 51.5 (C_2), 44.9 (C_{11}), 42.2 (C_{15}), 39.7 (C_{17}), 39.3 (C_{18}), 39.2 (br, C_7), 33.6 (C_{14}), 33.4 (C_{14}), 32.1 (br, C_5), 24.1 (C_{10}-Me), 23.6 (br, C_6-Me), 23.5 (br, C_4), (23.4 ($CH_3\underline{CH_2}S$), 21.6 (C_{14}-Me), 20.7 (C_{12}), 18.9 (C_8), 18.6 (C_{16}), 15.6 (C_{18}-Me), 14.7 (C_2-Me), 14.6 ($C\underline{H_3}CH_2S$).

HRMS (ESI) : calculated for $C_{26}H_{46}O_4SNa$ $[M+Na]^+$: 477.3015, found 477.3016

*Compounds **16e-f***

IR (neat) : v = 3370, 2934, 1686, 1456, 966 cm^{-1}.

^{1}H NMR (300 MHz, $CDCl_3$) δ 4.35 – 4.22 (m, 1H, H_3), 2.97 – 2.81 (m, 2H, $CH_3\underline{CH_2}S$), 2.74 (p, J = 7.2 Hz, 1H, H_2), 1.84 (ddt, J = 11.6, 5.4, 3.2 Hz, 1H), 1.72 – 1.33 (m, 14H), 1.32 (s, 3H, H_6-Me), 1.30 – 1.20 (m, 2H), 1.24 (t, J = 7.4 Hz, 3H, $C\underline{H_3}CH_2S$), 1.14 (d, J = 7.0 Hz, 3H, H_2-Me), 1.13 (s, 3H, H_{10}-Me), 1.17 – 1.06 (m, 1H), 0.96 (dt, J = 11.8, 3.6 Hz, 1H), 0.93 – 0.86 (m, 2H), 0.85 (s, 3H, H_{14}-Meβ), 0.77 (s, 6H, H_{18}-Me & H_{14}-Meα).

^{13}C NMR (101 MHz, $CDCl_3$, *45:55 mixture of diastereomers*) δ 201.0 (C_1), [81.7, 81.7*] (C_3), [80.7, 80.7*] (C_6), [74.6, 74.4*] (C_{10}), [62.3, 62.3*] (C_9), [56.3, 56.3*] (C_{13}), [51.1, 51.0*] (C_2), [44.8, 44.4] (C_{11}), [44.0, 43.9*] (br, C_7), 42.2 (C_{15}), [39.9*, 39.8] (C_{17}), [39.4, 39.4*] (C_{18}), 33.5 (C_{14}-Me), 33.4 (C_{14}), [32.6, 32.4*] (C_5), [24.3*, 24.2] (C_{10}-Me), 23.5 ($CH_3\underline{CH_2}S$), [23.0, 22.9*] (C_4), 21.6 (C_{14}-Me), [20.7, 20.6] (C_{12}), [20.6, 20.4*] (C_6-Me), 18.6 (C_{16}), [18.6, 18.4] (C_8), [15.6*, 15.6] (C_{18}-Me), 14.7 ($C\underline{H_3}CH_2S$), [13.5*, 13.5] (C_2-Me). *= minor diastereomer.

HRMS (ESI) : calculated for $C_{26}H_{46}O_4SNa$ $[M+Na]^+$: 477.3015, found 477.3015

Compounds **16g-h**

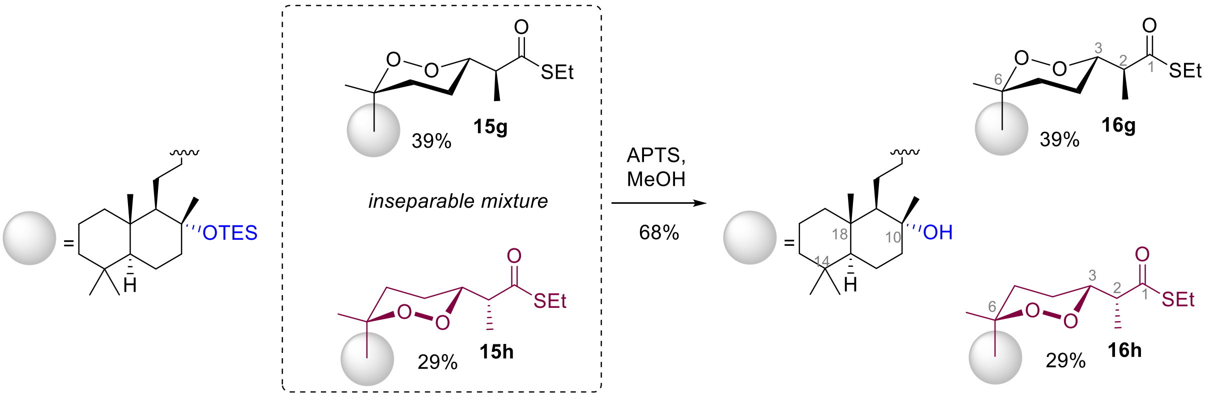

To a solution of the fraction F3 containing **15g-h** (85 mg, 0.15 mmol, 1 equiv) in MeOH (1.5 mL) was added PTSA (26 mg, 0.15 mmol, 1 equiv). The reaction mixture was stirred at rt for 10 min and was poured into a saturated aqueous solution of $NaHCO_3$. The aqueous phase was extracted with DCM thrice (20 mL), and the combined organic layers were washed with brine, dried over $MgSO_4$, and concentrated on a rotavapor under vacuum with gentle heating (no more than 30 °C). The crude was purified on a silica gel (petroleum ether: Et_2O, 80/20 to 60/40) to afford by order of elution **16h** (21 mg, 29%) and **16g** (28 mg, 39 %). The global yield was 68 %. Rf = 0.19 and 0.32 in petroleum Ether: Et_2O, 2:1 for **16h** and **16g**, respectively.

Compounds ***16h***

mp = 97 – 98 °C

[α]$^{25}_{D}$ = +42 (*c* 0.48, $CHCl_3$)

IR (neat) : *v* = 3368, 2926, 2854, 1687, 1457, 1387, 964 cm^{-1}.

^{1}H NMR (300 MHz, $CDCl_3$) δ 4.27 (td, J = 9.8, 8.2, 2.5 Hz, 1H, H_3), 2.87 (q, *J* = 7.4 Hz, 2H, $CH_3\underline{CH_2}S$), 2.70 (p, *J* = 7.3 Hz, 1H, H_2), 2.20 – 2.06 (m, 1H), 1.95 (brs, 1H, OH), 1.84 (dt, J = 12.2, 3.1 Hz, 1H), 1.74 – 1.50 (m, 7H), 1.50 – 1.30 (m, 5H), 1.29 – 1.20 (m, 2H), 1.23 (t, *J* = 7.5 Hz, 3H, $\underline{CH_3}CH_2S$), 1.18 (s, 3H, H_{10}-Me), 1.16 – 1.10 (m, 2H), 1.11 (d, *J* = 7.3 Hz, 3H, H_2-Me), 1.10 (s, 3H, H_6-Me), 0.95 (ddd, *J* = 12.1, 9.6, 2.8 Hz, 2H), 0.87 (s, 3H, H_{14}-Meβ), 0.78 (s, 6H H_{18}-Me & H_{14}-Meα).

^{13}C NMR (75 MHz, $CDCl_3$) δ 201.0 (C_1), 81.5 (C_3), 80.3 (C_6), 74.4 (C_{10}), 61.8 (C_9), 56.3 (C_{13}), 51.1 (C_2), 43.3 (C_{11}), 42.2 (C_{15}), 39.8 (C_{17}), 39.1 (C_{18}), 37.8 (br, C_7), 33.5 (C_{14}-Me), 33.4 (C_{14}), 33.2 (br, C_5), 24.5 (C_{10}-Me), 23.8 (C_6-Me), 23.5 ($CH_3\underline{CH_2}S$), 22.9 (C_4), 21.6 (C_{14}-Me), 20.5 (C_{12}), 18.7 (C_{16}), 18.2 (C_8), 15.6 (C_{18}-Me), 14.7 ($\underline{CH_3}CH_2S$), 13.4 (C_2-Me).

HRMS (ESI) : calculated for $C_{26}H_{46}O_4SNa$ $[M+Na]^+$: 477.3015, found 477.3013

Compound ***16g***

mp = 89 – 90 °C

[α]$^{25}_{D}$ = –33 (c 0.66, $CHCl_3$)

IR (neat) : *v* = 3368, 2932, 2868, 1687, 1456, 1387, 965 cm^{-1}.

^{1}H NMR (300 MHz, $CDCl_3$) δ 4.26 (td, *J* = 8.3, 4.2 Hz, 1H, H_3), 2.90 (dq, *J* = 13.1, 7.4 Hz, 1H, $CH_3\underline{CH_2}S$), 2.84 (dq, *J* = 13.1, 7.4 Hz, 1H, $CH_3\underline{CH_2}S$), 2.71 (p, *J* = 7.3 Hz, 1H, H_2), 2.01 – 1.68 (m, 4H), 1.72 – 1.46 (m, 6H), 1.46 – 1.28 (m, 6H), 1.28 – 1.17 (m, 5H), 1.30 – 1.14 (m, 2H), 1.14 (s, 3H, H_{10}-Me), 1.11 (s, 3H, H_6-Me), 1.11 (d, *J* = 7.1 Hz, 3H, H_2-Me), 1.02 (t, *J* = 3.8 Hz, 1H), 0.96 (dd, *J* = 12.9, 3.8 Hz, 1H), 0.91 (dd, *J* = 12.0, 2.1 Hz, 1H), 0.86 (s, 3H, H_{14}-Meβ), 0.79 (s, 3H, H_{18}-Me or H_{14}-Meα), 0.78 (s, 3H, H_{18}-Me or H_{14}-Meα).

^{13}C NMR (75 MHz, $CDCl_3$) δ ^{13}C NMR (75 MHz, $CDCl_3$) δ 201.0 (C_1), 81.3 (C_3), 80.4 (C_6), 74.6 (C_{10}), 62.4 (C_9), 56.4 (C_{13}), 51.1 (C_2), 45.0 (C_{11}), 42.3 (C_{15}), 39.7 (C_{17}), 39.4 (C_{18}), 38.6 (C_7), 33.6 (C_{14}-Me), 33.4 (C_{14}), 32.8 (C_5), 24.1 (C_{10}-Me), 23.8 (C_6-Me), 23.5 ($CH_3\underline{CH_2}S$), 22.8 (C_4), 21.7 (C_{14}-Me), 20.8 (C_{12}), 19.1 (C_8), 18.7 (C_{16}), 15.6 (C_{18}-Me), 14.7 ($\underline{CH_3}CH_2S$), 13.3 (C_2-Me).

HRMS (ESI) : calculated for $C_{26}H_{46}O_4SNa$ $[M+Na]^+$: 477.3015, found 477.3009

(+)-mycaperoxide D methyl ester **(2)**

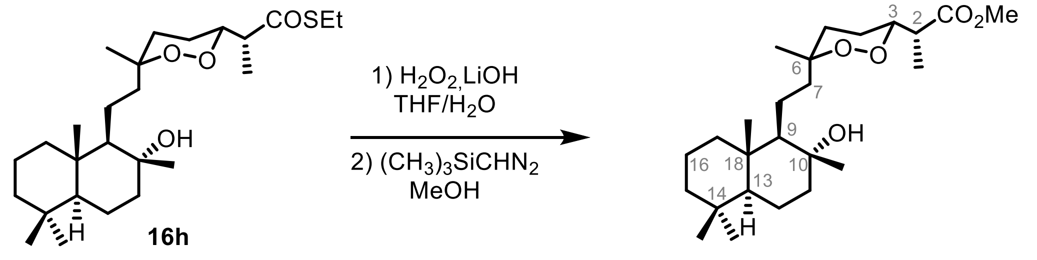

(+)-mycaperoxide D methyl ester **(2)**

To a solution of **16h** (23 mg, 0.05 mmol, 1 equiv) and H_2O_2 (34 mg, 0.3 mmol, 6 equiv, 30 % in water) in THF (2 mL) and water (1 mL) at 0 °C was added LiOH.H_2O (5 mg, 0.13 mmol, 2.5 equiv). The reaction mixture was then stirred at 0 °C for 1 h. After the disappearance of the starting material, the reaction mixture was poured into a 0.01 N aqueous solution of HCl (10 mL). The resulting mixture was extracted with DCM three times (about 10 mL). The combined organic layer was dried over $MgSO_4$ and was concentrated under a vacuum. Formic acid (1 mL) was added to ensure the protonation of the carboxylic acid. After evaporation to dryness under vacuum, anhydrous methanol (1 mL) was added to the residue, and the solution was cooled at 0 °C. TMSdiazomethane (2 M in hexanes) was added dropwise to the stirred solution at 0 °C until persistent yellow coloration was reached. The reaction mixture was then quenched with formic acid and was concentrated on a rotavapor with gentle heating (no more than 30 °C). The residue was purified on a silica gel (petroleum ether: Et_2O, 70/30 to 60/40 to afford **2** (16 mg, 76 %) as a white solid.

mp = 100 – 101 °C

$[\alpha]^{25}_D$ = +68 (*c* 0.25, $CHCl_3$) [litt: $[\alpha]_D$=–52° (*c* 0.3, $CHCl_3$) on (–)-mycaperoxide D methyl ester][6]

IR (neat) : *v* = 3368, 2922, 2854, 1742, 1458, 1387, 1199, 1161 cm^{-1}.

^{1}H NMR (300 MHz, $CDCl_3$) δ 4.28 – 4.18 (m, 1H, H_3), 3.69 (s, 3H, OMe), 2.53 (p, *J* = 7.3 Hz, 1H, H_2), 2.12 (t, *J* = 12.0, 10.0 Hz, 1H), 1.84 (dt, *J* = 12.1, 3.1 Hz, 1H), 1.97 – 1.68 (brs, 1H, OH), 1.76 – 1.55 (m, 8H), 1.49 – 1.33 (m, 4H), 1.33 – 1.21 (m, 2H), 1.18 (s, 3H, H_{10}-Me), 1.16 – 1.10 (m, 1H), 1.12 (d, *J* = 7.4 Hz, 3H, H_2-Me), 1.11 (s, 3H, H_6-Me), 0.96 (dd, *J* = 13.1, 3.3 Hz, 2H), 0.92 (dd, *J* = 12.0, 2.0 Hz, 1H), 0.87 (s, 3H, H_{14}-Meβ), 0.78 (s, 6H, H_{18}-Me & H_{14}-Meα).

^{13}C NMR (75 MHz, $CDCl_3$) δ 174.5 (C_1), 81.6 (C_3), 80.3 (C_6), 74.4 (C_{10}), 62.0 (C_9), 56.3 (C_{13}), 52.0 (COOMe), 43.3 (C_{11}), 42.8 (C_2), 42.2 (C_{15}), 39.8 (C_{17}), 39.1 (C_{18}), 37.9 (br, C_7), 33.5 (C_{14}-Me), 33.4 (C_{14}), 33.2 (br, C_5), 24.5 (C_{10}-Me), 23.8 (C_6-Me), 22.9 (C_4), 21.6 (C_{14}-Me), 20.5 (C_{12}), 18.7 (C_{16}), 18.3 (C_8), 15.7 (C_{18}-Me), 12.8 (C_2-Me).

HRMS (ESI) : calculated for $C_{25}H_{44}O_5Na$ $[M+Na]^+$: 447.3086, found 447.3090

Table S1: ^{13}C NMR data for the eight synthetic diastereisomers of (+)-mycaperoxides D ethyl thioester **16a-h**, synthetic (+)-mycaperoxide methyl ester **2** and natural (-)-mycaperoxide methyl ester.[6] Emphasis on the differences between the different stereoisomers.

Numbering	**16a**	**16b**	**16c**	**16d**	**16e**	**16f**	**16g**	**16h**	**2**	Mycaperoxide D Methyl ester (natural)	Δ **16h** – natural product	Δ **2** – natural product
C_1	201.0	200.9	200.9	200.7	200.8	200.8	200.9	200.9	174.4	174.4	-26.5	0.0
C_2	51.7	51.5	51.4	51.4	51.0	50.9	50.9	50.9	42.7	42.6	-8.3	-0.1
C_2-Me	14.9	14.9	14.4	14.8	13.3	13.3	13.2	13.2	12.7	12.7	-0.5	0.0
C_3	81.6	81.5	81.1	81.4	81.5	81.5	81.1	81.4	81.4	81.4	0.0	0.0
C_4	23.7	23.6	23.3	23.4	22.8	22.7	22.6	22.7	22.7	22.7	0.0	0.0
C_5	32.1	31.9	32.0	32.6	32.5	32.3	32.5	33.1	33.0	33.0	-0.1	0.0
C_6	80.8	80.8	80.5	80.4	80.5	80.5	80.2	80.1	80.1	80.1	0.0	0.0
C_6-Me	20.9	21.2	23.5	23.7	20.2	20.5	23.7	23.6	23.6	23.6	0.0	0.0
C_7	43.4	43.0	39.1	38.4	43.9	43.7	38.3	37.7	37.7	37.7	0.0	0.0
C_8	18.6	18.4	18.8	18.0	18.6	18.4	18.8	18.0	18.1	18.1	0.1	0.0
C_9	62.3	62.3	62.3	62.0	62.2	62.1	62.2	61.6	61.8	61.8	0.2	0.0
C_{10}	74.5	74.4	74.5	74.3	74.4	74.2	74.5	74.3	74.2	74.2	-0.1	0.0
C_{10}-Me	24.2	24.3	23.9	24.4	24.0	24.1	23.9	24.3	24.3	24.4	0.1	0.1
C_{11}	44.9	44.2	44.8	43.2	44.7	44.2	44.7	43.1	43.1	43.1	0.0	0.0
C_{12}	20.7	20.6	20.6	20.3	20.5	20.5	20.6	20.4	20.4	20.3	-0.1	-0.1
C_{13}	56.3	56.3	56.2	56.2	56.1	56.1	56.2	56.1	56.2	56.1	0.0	-0.1
C_{14}	33.4	33.4	33.2	33.2	33.2	33.2	33.2	33.2	33.2	33.2	0.0	0.0
C_{14}-Me α	33.5	33.5	33.4	33.4	33.4	33.4	33.4	33.4	33.4	33.4	0.0	0.0
C_{14}-Me β	21.6	21.6	21.5	21.4	21.5	21.5	21.5	21.4	21.4	21.4	0.0	0.0
C_{15}	42.2	42.2	42.0	42.0	42.0	42.0	42.1	42.0	42.0	42.0	0.0	0.0
C_{16}	18.6	18.6	18.5	18.5	18.6	18.6	18.5	18.5	18.5	18.5	0.0	0.0
C_{17}	39.8	39.9	39.5	39.6	39.7	39.6	39.4	39.6	39.6	39.6	0.0	0.0
C_{18}	39.4	39.3	39.2	38.9	39.3	39.2	39.2	39.0	39.0	38.9	-0.1	-0.1
C_{18}-Me	15.6	15.6	15.5	15.5	15.4	15.4	15.4	15.5	15.5	15.5	0.0	0.0
$\underline{CH_n}X(CO)$	23.4	23.4	23.4	23.3	23.3	23.3	23.3	23.3	51.9	51.9	28.6	0.0
$\underline{CH_3}CH_2S$	14.8	14.8	14.6	14.6	14.5	14.5	14.5	14.6	–	–	–	–
Relative configuration	*trans*-dioxane 2,3-*syn* 2,3,6-*epi*	*trans*-dioxane 2,3-syn –	*cis*-dioxane *2,3-syn* 2,3,6-*epi*	*cis*-dioxane 2,3-*syn* –	*trans*-dioxane 2,3-*anti* 2,3,6-*epi*	*trans*-dioxane 2,3-*anti* –	*cis*-dioxane 2,3-*anti* 2,3,6-*epi*	*cis*-dioxane 2,3-*anti* –	*cis*-dioxane 2,3-*anti* –	*cis*-dioxane 2,3-*anti* –		

Reported values in the table with calibration at 77.00 ppm for $CDCl_3$ solvant peak, for comparison with the data of the natural product. C_2, C_3 and C_4 data (blue colors) emphases on the 2,3- syn/anti relationship. C_6 and C_7 data (green colors) emphases on the cis/trans relationship of the dioxane. C_6- Me, C_7, C_8 and C_{11} (orange colors) emphases on the relative configuration at C_2, C_3, and C_6 compared to the decaline.

Compound **17**

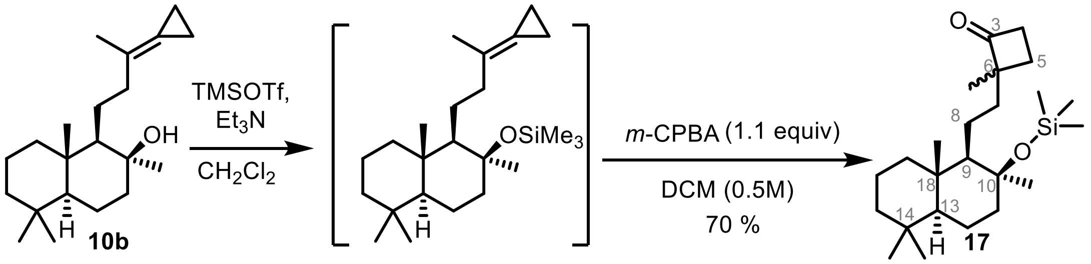

To a solution of **10b** (0.86 g, 2.82 mmol, 1 equiv) and triethylamine (1.15 mL, 8.46 mmol, 3 equiv) in anhydrous DCM (28 mL, 0.1M) cooled to –40 °C was added TMSOTf (1 mL, 5.64 mmol, 2 equiv). After 2 h of stirring at rt, the reaction mixture was poured into water, and the aqueous phase was extracted twice with DCM (50 mL). The combined organic layers were concentrated under a vacuum. The residue was diluted with DCM (5.6 mL), and the solution was cooled to 0 °C. *m*-CPBA (738 mg, 3.1 mmol, 1.1 equiv, 70-77%) was added in 5 portions over 15 min. After 1 h of stirring at rt, the reaction mixture was quenched with a 10% aqueous solution of $Na_2S_2O_3$, and the mixture was stirred vigorously for 20 min. The organic layer was extracted with diethyl ether (2 x 100 mL). The combined organic layers were washed with a saturated solution of $NaHCO_3$ (2 x 25 mL), dried over $MgSO_4$, and concentrated under a vacuum. The purification of the residue by flash chromatography on silica gel (petroleum ether: ether, 96:4) afforded **17** (775 mg, 70 %) as a yellow oil in about 1:1 mixture of diasteromers.

IR (neat) : *v* = 2953, 2922, 2866, 2843, 1776, 1458, 1248, 1182, 1092, 1053, 999, 756 cm^{-1}.

^{1}H NMR (300 MHz, Chloroform-*d, about 1:1 mixture of diastereomers*) δ 2.99 (tt, *J* = 7.5, 1.8 Hz, 2H, H_4), 2.00 – 1.87 (m, 1H), 1.82 – 1.68 (m, 2H), 1.67 – 1.41 (m, 6H), 1.41 – 1.24 (m, 4H), 1.20 (s, 3H, H_6-Me), 1.20 – 1.03 (m, 2H), 1.15 (s, 1.5H, H_{10}-Me, *1 diastereomer*), 1.13 (s, 1.5H, H_{10}-Me, *1 diastereomer*), 0.88 – 0.79 (m, 1H), 0.87 (s, 1.5H, H_{18}-Me, *1 diastereomer*), 0.86 (s, 1.5H, H_{18}-Me, *1 diastereomer*), 0.85 (s, 3H, C_{14}-Meβ), 0.82 (s, 3H, C_{14}-Meα), 0.80 – 0.71 (m, 1H), 0.55 (t, *J* = 3.4 Hz, 1H), 0.10 (s, 4.5H, SiC<u>H</u>$_3$, *1 diastereomer*), 0.09 (s, 4.5H, SiC<u>H</u>$_3$, *1 diastereomer*).

^{13}C NMR (75 MHz, Chloroform-*d*) δ [216.1, 216.0] (C_3), [76.6, 76.5] (C_{10}), 65.0 (C_6), 60.9 (C_9), 56.4 (C_{13}), [42.7, 42.7] (C_{11}), 42.4 (C_{15}), [39.7, 39.6] (C_4), 39.6 (C_{17}), 39.5 (C_7), [39.4, 39.3] (C_{18}), 33.7 (C_{14}-Meβ), 33.4 (C_{14}), [30.4, 30.3] (C_{10}-Me), 24.0 (C_8), 21.9 (C_6-Me), [20.7, 20.5] (C_{14}-Meα), [20.3, 20.1] (C_5), 18.6 (C_{16}), 18.4 (C_{12}), 15.3 (C_{18}-Me), 2.7 ($SiMe_3$).

HRMS (ESI) : calculated for $C_{24}H_{44}O_2SiNa$ $[M+Na]^+$: 415.3008, found 415.3007

Compound **18**

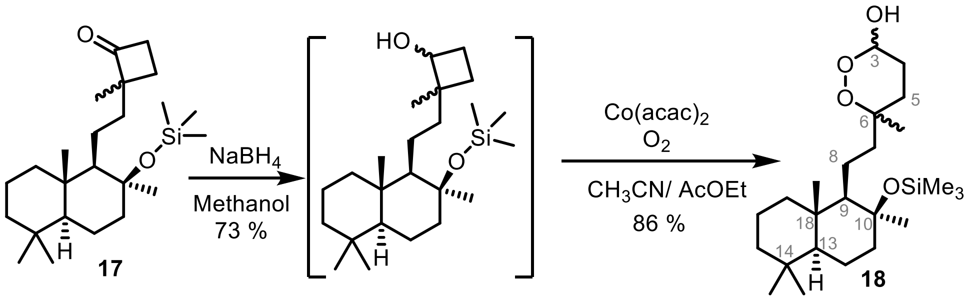

To a solution of **17** (393 mg, 1 mmol, 1 equiv) in methanol (4 mL, 0.25 M) at 0 °C was added $NaBH_4$ (38 mg, 1 mmol, 1 equiv) in 3 portions over 10 minutes. The reaction was then stirred at rt for 1h, filtered on a pad of silica gel with (3x30 mL of ethyl acetate), and the filtrate was concentrated under a vacuum. Acetonitrile (10 mL), ethylacetate (5 mL) were added to the residue followed by $Co(acac)_2$ (15 mg, 0.05 mmol, 0.05 equiv). The brownish reaction mixture was then lightly bubbled with O_2 for 5 min, and the oxygen atmosphere was maintained with a rubber balloon of O_2. The reaction was then heated to 40 °C with an oil bath, and its color turned slowly to intense green. The reaction was monitored by TLC, and after 4 h of reaction under these conditions, the reaction mixture was concentrated on a rotavapor with gentle heating (no more than 30 °C). The crude was purified on a silica gel to afford **18** (310 mg, 73 %) as colorless oil in about 1:1:1:1 mixture of diastereomers.

IR (neat) : *v* = 3400, 2947, 2924, 1458, 1248, 1090, 1055, 999, 752 cm^{-1}.

^{1}H NMR (300 MHz, $CDCl_3$, *about 1:1:1:1 mixture of diastereomers*) δ 5.21 – 5.08 (m, 1H, H_3), 3.12 – 2.97 (m, 1H, OH), 2.01 – 1.83 (m, 1H), 1.78 (dt, *J* = 13.4, 4.1 Hz, 0.5H, *2 diastereomers*), 1.24 (s, 1.5H, H_6-Me, *2 diastereomers*), 1.70 – 1.20 (m, 13.5H), 1.23 (s, 1.5H, H_6-Me, *2 diastereomers*), 1.11 (s, 1.5H, H_{10}-Me, *2 diastereomers*), 1.06 (s, 1.5H, H_{10}-Me, *2 diastereomers*), 1.06 – 0.95 (m, 2H), 0.79 – 0.70 (m, 1H), 0.78 (s, 3H, H_{18}-Me), 0.75 (s, 3H, H_{14}-Meβ), 0.72 (s, 3H, , H_{14}-Meα), 0.75 – 0.65 (m, 1H), 0.54 – 0.43 (m, 1H), 0.00 (s, 9H, SiC<u>H</u>$_3$).

^{13}C NMR (75 MHz, $CDCl_3$, *about 1:1:1:1 mixture of diastereomers*) δ [96.6, 96.5, 96.4, 96.3] (C_3), 81.1 (C_6), 76.6 (C_{10}), [60.8, 60.8] (C_9), [56.5, 56.4] (C_{13}), [42.8, 42.8] (C_{11}), [42.4, 42.3] (C_{15}), [42.0, 41.7, 39.5, 39.4] (C_7) [40.7, 40.6] (C_{17}), [39.6, 39.4] (C_{10}), 33.7 (C_{14}-Meβ), 33.4 (C_{14}), [30.4, 30.3, 30.2] (C_{10}-Me), [29.6, 29.2, 28.7, 28.5] (C_5), [25.9, 25.8, 25.7, 25.6] (C_4), [22.3, 22.1] (C_6-Me), 21.9 (C_{14}-Meα), [21.4, 21.2] (C_6-Me), [19.6, 19.6, 19.0, 18.9] (C_8), 18.6 (C_{16}), [18.5, 18.4] (C_{12}), [15.3, 15.2] (C_{18}-Me), 2.7 (SiMe).

HRMS (ESI) : calculated for $C_{24}H_{46}O_4SiNa$ $[M+Na]^+$: 449.3063, found 449.3065

Compound **19**

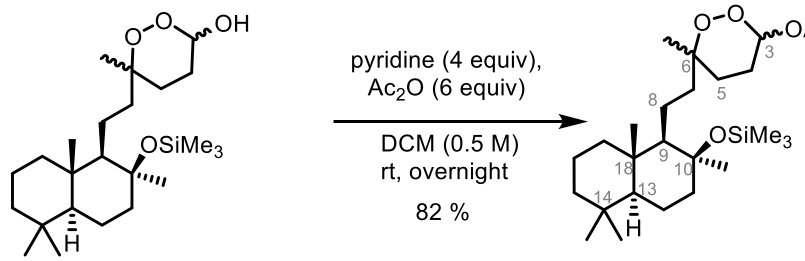

To a solution of **18** (299 mg, 1 equiv, 0.7 mmol) in DCM (1.4 mL), were added AC_2O (0.4 mL, 4.2 mmol, 6 equiv) and pyridine (0.11 mL, 1.4 mmol, 4 equiv). The reaction mixture was stirred overnight at rt and was poured into an Erlenmeyer containing a saturated $NaHCO_3$ solution. The mixture was stirred vigorously for 30 min at rt, and the aqueous layer was extracted 3 times with 20 mL of dichloromethane. The combined organic phases were washed with a 10% citric acid aqueous solution (2x10 mL), dried over $MgSO_4$, filtered, and concentrated on a rotavapor under vacuum with gentle heating (no more than 30 °C). The crude was purified on a silica gel to afford **19** (270 mg, 82 %) as colorless oil in about 1:1:1:1 mixture of diastereomers.

IR (neat) : v = 2955, 2947, 2924, 1749, 1458, 1373, 1246, 1229, 1182, 835, 752 cm^{-1}.

^{1}H NMR (300 MHz, $CDCl_3$, *about 1:1:1:1 mixture of diastereomers*) δ 6.29 – 6.14 (m, 1H, H_3), 2.13 (s, 3H, OAc), 2.12 – 1.98 (m, 1H), 1.87 (dt, J = 13.1, 4.3 Hz, 0.5H, *2 diastereomers*), 1.84 – 1.21 (m, 13.5H), 1.55 (s, 1.5H, H_6-Me, *2 diastereomers*), 1.37 (s, 1.5H, H_6-Me, *2 diastereomers*), 1.19 – 1.03 (m, 2H), 1.16 (s, 3H, H_{10}-Me), 0.89 – 0.80 (m, 1H), 0.88 (s, 1.5H, H_{18}-Me, *2 diastereomers*), 0.87 (s, 1.5H, H_{18}-Me, *2 diastereomers*), 0.85 (s, 3H, H_{14}-Meβ), 0.82 (s, 3H, H_{14}-Meα), 0.85 – 0.75 (m, 1H), 0.65 – 0.49 (m, 1H), 0.10 (s, 2.25H, $SiMe_3$, *1 diastereomer*), 0.10 (s, 2.25H $SiMe_3$, *1 diastereomer*), 0.09 (s, 2.25H $SiMe_3$, *1 diastereomer*), 0.09 (s, 2.25H $SiMe_3$, *1 diastereomer*).

^{13}C NMR (75 MHz, $CDCl_3$, *about 1:1:1:1 mixture of diastereomers*) δ [170.2, 170.1] (CH_3-$\underline{C}$=O), [95.2, 95.1, 94.9, 94.8] (C_3), [81.7, 81.7, 81.6, 81.5] (C_6), 76.6 (C_{10}), [60.8, 60.8] (C_9), [56.5, 56.4] (C_{13}), [42.7, 42.7] (C_{11}), [42.4, 41.9, 39.6, 39.5] (C_7) , [42.3, 42.3] (C_{15}), [40.7, 39.7] (C_{17}), (C_7), 39.4 (C_{18}), 33.7 (C_{14}-Meβ), 33.4 (C_{14}), [30.5, 30.4, 30.3] (C_{10}-Me), [29.2, 28.7, 27.9, 27.7] (C_5), [23.2, 23.2, 23.1, 23.1] (C_4), [22.0, 21.9] (C_6-Me), [21.9, 21.8] (C_{14}-Meα), 21.3 ($\underline{C}H_3$-C=O), [19.6, 19.5, 18.8] (C_8), [18.6, 18.6] (C_{16}), 18.5 (C_{12}), [15.3, 15.2, 15.2] (C_{18}-Me), 2.7 (SiMe).

HRMS (ESI) : calculated for $C_{26}H_{48}O_5SiNa$ $[M+Na]^+$: 491.3169, found 491.3168

Compounds **20a-h**

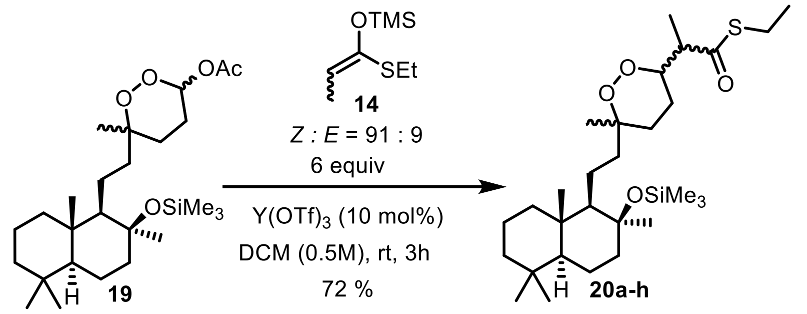

To a solution of **19** (1equiv, 0.5 mmol, 234 mg) and **14** (571 mg, 3 mmol, 6 equiv) in dry DCM (1 mL, 0.5 M) was added $Y(OTf)_3$ (27 mg, 0.05 mmol, 0.1 equiv). The reaction mixture was stirred at rt for 3 h and was poured into a saturated aqueous solution of $NaHCO_3$. The aqueous phase was extracted with DCM 3 times (20 mL), and the combined organic layers were washed with brine. Then the organic layer was dried over $MgSO_4$ and concentrated on a rotavapor under a vacuum with gentle heating (no more than 30 °C). The crude was purified on a silica gel to afford by order of elution three fractions: F1 (30 mg, 11 %) containing **20a-b**, F2 (95 mg, 36 %) containing **20c-e**, and F3 (63 mg, 24 %) containing **20g-h** as colorless oils. The global yield is 72 %.

Fraction F1 ***20a:20b*** = 55:45

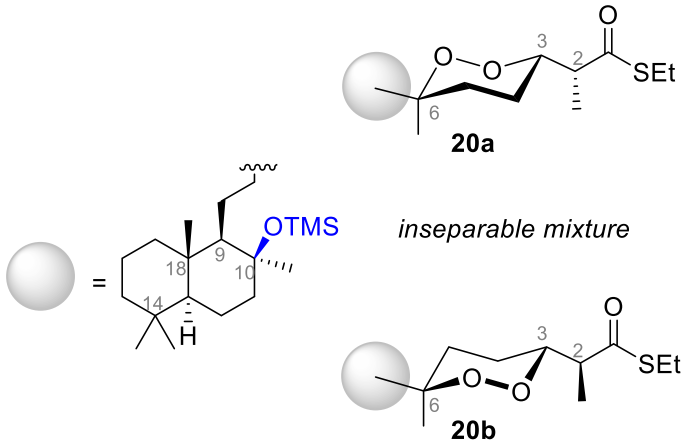

IR (neat) : *ν* = 2926, 2868, 1684, 1456, 1373, 1248, 1182, 1055, 835, 752 cm^{-1}.

^{1}H NMR (300 MHz, Chloroform-*d, about 1:1 mixture of diastereomers*) δ 4.18 – 4.01 (m, 1H, H_3), 2.87 (q, *J* = 7.1 Hz, 1H, S-C$\underline{H_2}$-CH_3, *1 diastereomer*), 2.86 (q, *J* = 7.1 Hz, 1H, S-$\underline{CH_2}$-CH_3, *1 diastereomer*), 2.89 – 2.76 (m, 1H, H_2), 1.80 – 1.21 (m, 15H), 1.31 – 1.26 (m, 6H, H_2-Me, S-CH_2-C$\underline{H}_3$), 1.26 (s, 1.5H, H_6-Me, *1 diastereomer*), 1.25 (s, 1.5H, H_6-C$\underline{H}_3$, *1 diastereomer*), 1.20 – 1.04 (m, 2H), 1.14 (s, 1,5H, H_{10}- Me, *1 diastereomer*), 1.13 (s, 1,5H, H_{10}-Me, *1 diastereomer*), 0.88 – 0.80 (m, 1H), 0.86 (s, 3H, H_{18}-Me), 0.84 (s, 3H, H_{14}-Meβ), 0.81 (s, 3H, H_{14}- Meα), 0.80 – 0.74 (m, 1H), 0.57 – 0.51 (m, 1H), 0.09 (s, 4.5H, $SiMe_3$, *1 diastereomer*), 0.08 (s, 4.5H $SiMe_3$, *1 diastereomer*).

^{13}C NMR (75 MHz, Chloroform-*d,* ***20a****:****20b*** *= 55:45 mixture of diastereomers*) δ [200.9*, 200.9] (C_1), [81.7, 81.7*] (C_3), [81.0*, 80.9] (C_6), [76.6*, 76.6] (C_{10}), [60.8*, 60.8] (C_9), 56.4 (C_{13}), [51.7] (C_2), [43.3, 43.2*] (C_7), [42.7, 42.7] (C_{11}), 42.4 (C_{15}), [39.6*, 39.4] (C_{17}), [39.4*, 39.3] (C_{18}), 33.7 (C_{14}-Meβ), 33.4 (C_{14}), [32.1, 31.5*] (C_5), [30.4, 30.3*] (C_{10}-Me), [23.9*, 23.8] (C_4), 23.5 (SC$\underline{H}_2$$CH_3$), 21.9 ($C_{14}$-Meα),

[21.0*, 20.7] (C_6-Me), [19.0*, 18.9] (C_8), 18.6 (C_{16}), 18.4 (C_{12}), [15.2, 15.2*] (C_{18}-Me), 14.9 (C_2-Me), 14.8 ($SCH_2C\underline{H}_3$), 2.7 (SiMe). * = minor isomer = **20b**

HRMS (ESI) : calculated for $C_{29}H_{54}O_4SSiNa$ $[M+Na]^+$: 549.3410, found 549.3409

Fraction F2 : ***20c:20d:20e:20f*** ≈ 0.17:0.17:0.33:0.33 (trans : cis =1:2)

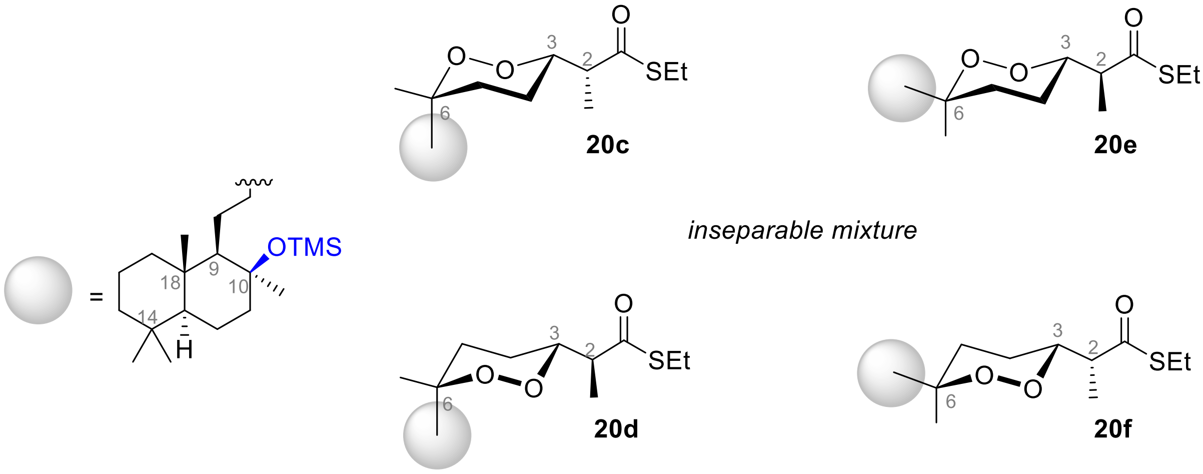

IR (neat) : *ν* = 2941, 2868, 1684, 1456, 1373, 1248, 1182, 1055, 835, 750 cm^{-1}.

^{1}H NMR (300 MHz, $CDCl_3$, ***20c:20d:20e:20f*** *≈ 0.17:0.17:0.33:0.33 mixture of diastereomers*) δ 4.28 (m, 0.33H, H_3, *2 trans diastereomers*), 4.19 – 4.04 (m, 0.67H, H_3, *2 cis diastereomers*), 2.87 (q, *J* = 7.0 Hz, 2H, S-C$\underline{H}_2$-CH_3), 2.82 – 2.65 (m, 1H, H_2), 1.82 – 1.19 (m, 15H), 1.33 (s, 0.5H, H_6-Me, *1 diastereomer*), 1.33 (s, 0.5H, H_6-Me, *1 diastereomer*), 1.25 (d, *J* = 6.0Hz, 2H, H_2-Me, 2 *trans diastereomer*), 1.24 (2t, *J* = 6.5 Hz, 3H, $SCH_2C\underline{H}_3$), 1.18 – 1.12 (m, 2H), 1.18 (s, 1H, H_6-Me, *1 cis diastereomer*), 1.16 (s, 3H, H_{10}-Me), 1.14 (d, *J* = 6.0Hz, 1H, H_2-Me, 2 *trans diastereomers*), 1.13 (s, 1H, H_6-Me, 1H, *1 cis diastereomer*), 0.89 – 0.79 (m, 1H), 0.87 (s, 1.5H, H_{18}-Me, *2 diastereomers*), 0.85 (s, 1.5H, H_{18}-Me*, 2 diastereomers*), 0.85 (s, 2H, H_{14}-Meβ, *2 cis diastereomers*), 0.84 (s, 1H, H_{14}-Meβ, *2 trans diastereomers*), 0.82 (s, 2H, H_{14}-Meα, 2 *cis* diastereomers), 0.81 (s, 1H, H_{14}-Meα, 2 *trans* diastereomers), 0.83 – 0.74 (m, 1H), 0.58 (m, 0.67H, *2 cis diastereomers*), 0.52 (m, 0.33H, *2 trans diastereomers*), 0.09 (s, 3H $SiMe_3$, *1 cis diastereomer*), 0.09 (s, 3H $SiMe_3$, *1 cis diastereomer*), 0.09 (s, 1.5H $SiMe_3$, *1 trans diastereomer*), 0.08 (s, 1.5H $SiMe_3$, *1 trans diastereomer*).

^{13}C NMR (75 MHz, $CDCl_3$) δ 200.9 (C_1), [81.8*, 81.2, 81.2] (C_3), [80.8*, 80.8*, 80.7] (C_6), [76.6*, 76.5*, 76.5] (C_{10}), [60.9, 60.8, 60.8* 60.8*] (C_9), [56.5, 56.4*] (C_{13}), [51.6, 51.5, 51.1*] (C_2), [43.9*, 43.8*, 39.5, 39.4] (C_7), [42.8, 42.8, 42.7*, 42.6*] (C_{11}), [42.4, 42.4] (C_{15}), [39.6, 39.4] (C_{17}), 39.3 (C_{18}), 33.7(C_{14}), 33.4 (C_{14}-Meβ), [32.6*, 32.0*, 31.6] (C_5), [30.4, 30.4*, 30.3*, 30.2](C_{10}-Me), [23.7, 23.6, 23.0, 22.9*] (C_4), 23.6 23.4 ($SC\underline{H}_2CH_3$), 21.9(C_{14}-Meα), [20.6, 20.2] (C_6-Me), [19.3, 19.5, 18.9*, 18.9*] (C_8), [18.6, 18.6*] (C_{12}), [18.5, 18.4*] (C_{16}), [15.3, 15.2] (C_{18}-Me), 14.7 ($SCH_2C\underline{H}_3$), [14.7, 14.6, 13.4*, 13.4*] (C_2-Me), 2.7(SiMe). *=minor isomers = **20e-f**

HRMS (ESI) : calculated for $C_{29}H_{54}O_4SSiNa$ $[M+Na]^+$: 549.3410, found 549.3406

Fraction F3 : ***20g:20h*** = 55:45

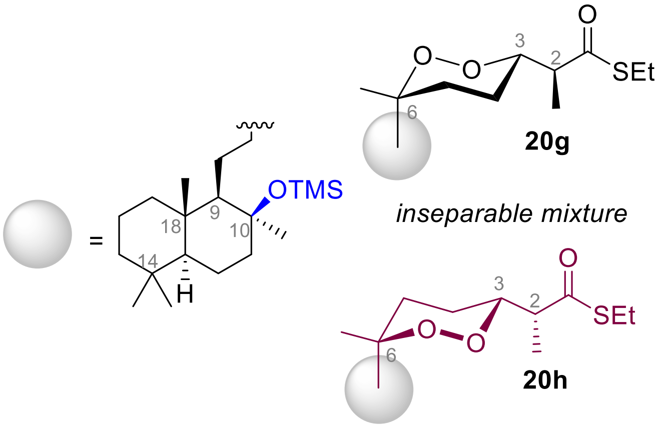

IR (neat) : v = 2941, 2868, 1686, 1456, 1373, 1248, 1184, 1055, 835, 750 cm^{-1}.

^{1}H NMR (300 MHz, $CDCl_3$, *about 1:1 mixture of diastereomers*) δ 4.44 – 4.14 (m, 1H, H_3), 2.93 –2.80 (m, 2H, S-C<u>H</u>$_2$-CH_3), 2.74 (2p, J = 7.5 Hz, 1H, H_2), 1.83 – 1.71 (m, 3H), 1.68 – 1.48 (m, 7H), 1.47 – 1.30 (m, 5H), 1.24 (t, J = 7.4 Hz, 3H, S-CH_2-C<u>H</u>$_3$), 1.20 – 1.07 (m, 2H), 1.19 (s, 1.5H, H_6-Me, *1 diastereomer*), 1.16 (s, H_6-Me, *1 diastereomer*), 1.12 (d, J = 7.0 Hz, 3H, H_2-Me), 1.12 (s, 3H, H_{10}-Me), 0.88 – 0.80 (m, 1H), 0.88 (s, 3H, H_{18}-Me), 0.85 (s, 3H, H_{14}-Meβ), 0.82 (s, 3H, H_{14}-Meα), 0.83 – 0.74 (m, 1H, H_{13}), 0.60 (brt, J = 3.8 Hz, 1H, H_9), 0.09 (s, 4.5H $SiMe_3$, *1 diastereomer*), 0.09 (s, 4.5H $SiMe_3$, *1 diastereomer*).

^{13}C NMR (75 MHz, $CDCl_3$, **20g**:**20h** = 55:45 mixture of diastereomers) δ [200.9, 200.8*] (C_1), [81.3, 81.1*] (C_3), [80.5, 80.4*] (C_6), 76.5 (C_6), [60.9*, 60.8] (C_9), 56.5 (C_{13}), 51.0 (C_2), [42.8*, 42.8] (C_{11}), [42.5*, 42.4] (C_{15}), [39.5*, 39.3] (C_{17}), 39.4, (C_{18}), 38.6 (C_7), 33.7 (C_{14}-Meβ), 33.4 (C_{14}), [32.4, 32.1*] (C_5), [30.4, 30.2*] (C_{10}-Me), 24.0 (C_6-Me), 23.4 (SC<u>H</u>$_2$$CH_3$), [22.8, 22.7*] ($C_4$), 21.9 ($C_{14}$-Meα), [19.4, 19.3*] ($C_8$), 18.6 ($C_{16}$), 18.5 ($C_{12}$), [15.3*, 15.2] ($C_{18}$-Me), 14.7 ($SCH_2$C<u>H</u>$_3$), [13.3, 13.1*] C_2-Me, 2.7 (SiMe).* = minor isomer = **20h**

HRMS (ESI) : calculated for $C_{29}H_{54}O_4SSiNa$ $[M+Na]^+$: 549.3410, found 549.3416

<u>Compounds **21g-h**</u>

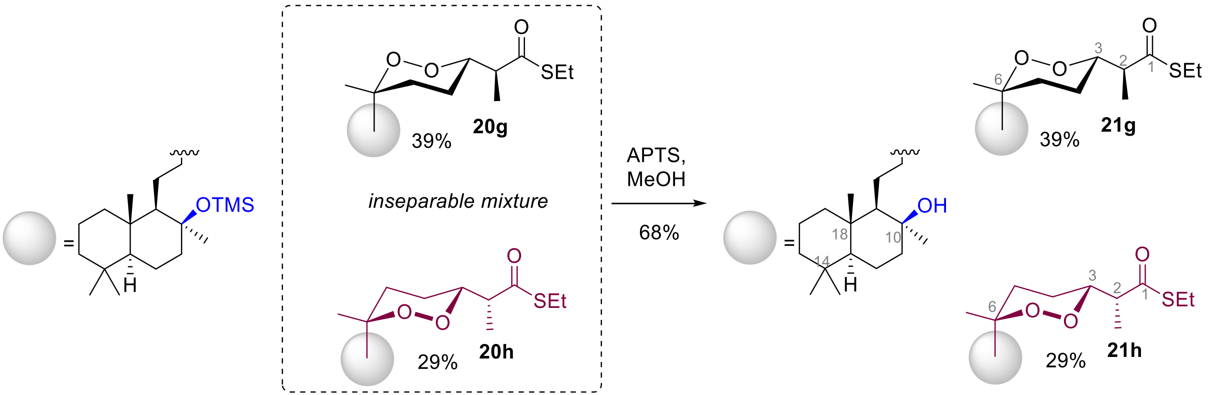

To a solution of **20g-h** (53 mg, 1 equiv, 0.1 mmol,) in methanol (2 mL, 0.05M) was added PTSA (10 mg, 0.05 mmol, 0.4 equiv). The reaction mixture was stirred at rt for 1.5 h and was poured into a saturated aqueous solution of $NaHCO_3$. The aqueous phase was extracted with DCM (3x20 mL), and

the combined organic layers were washed with brine, dried over $MgSO_4$, and concentrated on a rotavapor with gentle heating (no more than 30 °C). The residue was purified on a silica gel to afford by order of elution **21h** (21 mg, 46 %) as a yellow oil, then **21g** (23 mg, 50 %) as a white solid. The global yield was 96 %.

*Compound **21h***

$[\alpha]^{20}_D$ = +77.5 (*c* 0.124, $CHCl_3$)

IR (neat) : *ν* = 2922, 2866, 1684, 1458, 1373, 966 cm^{-1}.

^{1}H NMR (300 MHz, Chloroform-*d*) δ 4.33 – 4.19 (m, 1H, H_3), 2.90 (dq, *J* = 13.1, 7.4 Hz, 1H, S-C$\underline{H}_2$-CH$_3$), 2.84 (dq, *J* = 13.1, 7.4 Hz, 1H, S-C$\underline{H}_2$-CH$_3$), 2.73 (p, *J* = 7.2 Hz, 1H, H_2), 1.88 (td, *J* = 13.3, 4.1 Hz, 1H), 1.81 – 1.71 (m, 2H), 1.70 – 1.47 (m, 9H), 1.46 – 1.31 (m, 4H), 1.30 – 1.09 (m, 2H), 1.24 (t, *J* = 7.4 Hz, 3H, S-CH$_2$-C$\underline{H}_3$), 1.18 (s, 3H, H_{10}-Me), 1.13 (s, 3H, H_6-Me), 1.12 (d, *J* = 6.3 Hz, 3H, H_2-Me,), 0.96 – 0.73 (m, 3H), 0.97 (s, 3H, H_{18}-Me), 0.87 (s, 3H, H_{14}-Meβ), 0.83 (s, 3H, H_{14}-Meα),

^{13}C NMR (75 MHz, Chloroform-*d*) δ 200.8 (C_1), 81.3 (C_3), 80.3 (C_6), 73.3 (C_{10}), 59.3 (C_9), 56.2 (C_{13}), 51.0 (C_2), 42.3 (C_{11}), 42.2 (C_{15}), 39.4 (C_{17}), 39.3 (C_{18}), 38.7 (C_7), 33.6 (C_{14}-Meβ), 33.4 (C_{14}), 32.3 (C_5), 30.7 (C_{10}-Me), 23.9 (C_6-Me), 23.5 (SC$\underline{H}_2$CH$_3$), 22.8 (C_4), 21.8 (C_{14}-Meα), 19.0 (C_{12}), 18.5 (C_{16}), 18.4(C_8), 15.3 (SCH$_2$C$\underline{H}_3$), 14.7 (C_{18}-Me), 13.3 (C_2-Me).

HRMS (ESI) : calculated for $C_{26}H_{46}O_4SNa$ $[M+Na]^+$: 477.3015, found 477.3008

*Compound **21g***

mp = 137– 138 °C

$[\alpha]^{20}_D$ = –32.6 (*c* 0.062, $CHCl_3$)

IR (neat) : *ν* = 2928, 2868, 1684, 1558, 1506, 1456, 1373, 964 cm^{-1}.

^{1}H NMR (300 MHz, Chloroform-*d*) δ 4.36 – 4.20 (m, 1H, H_3), 2.90 (dq, *J* = 13.1, 7.4 Hz, 1H, S-C$\underline{H}_2$-CH$_3$), 2.84 (dq, *J* = 13.1, 7.4 Hz, 1H, S-C$\underline{H}_2$-CH$_3$), 2.73 (p, *J* = 7.2 Hz, 1H, H_2), 1.93 – 1.69 (m, 3H), 1.69 – 1.31 (m, 13H), 1.27 – 1.10 (m, 2H), 1.24 (t, *J* = 7.4 Hz, 3H, S-CH$_2$-C$\underline{H}_3$), 1.14 (s, 3H, H_{10}-Me), 1.13 (d, *J* = 6.5 Hz, 3H, H_2-Me,) 1.12 (s, 3H, H_6-Me), 0.96 (s, 3H, H_{18}-Me), 0.92 – 0.73 (m, 2H), 0.87 (s, 3H, H_{14}-Meβ), 0.83 (s, 3H, H_{14}-Meα).

^{13}C NMR (75 MHz, Chloroform-*d*) δ 200.8 (C_1), 81.2 (C_3), 80.3 (C_6), 73.4 (C_{10}), 59.4 (C_9), 56.2 (C_{13}), 51.0 (C_2), 42.4 (C_{11}), 42.3 (C_{15}), 39.3 (C_{18}), 39.1 (C_{17}), 38.7 (C_7), 33.6 (C_{14}-Meβ), 33.4 (C_{14}), 32.5 (C_5), 30.7 (C_{10}-Me), 24.0 (C_6-Me), 23.5 (SC$\underline{H}_2$CH$_3$), 22.8 (C_4), 21.8 (C_{14}-Meα), 19.0 (C_{12}), 18.5 (C_{16}), 18.4(C_8), 15.2 (SCH$_2$C$\underline{H}_3$), 14.7 (C_{18}-Me), 13.2 (C_2-Me).

HRMS (ESI) : calculated for $C_{26}H_{46}O_4SNa$ $[M+Na]^+$: 477.3015, found 477.3026

(+)-mycaperoxide C methyl ester (**1**)

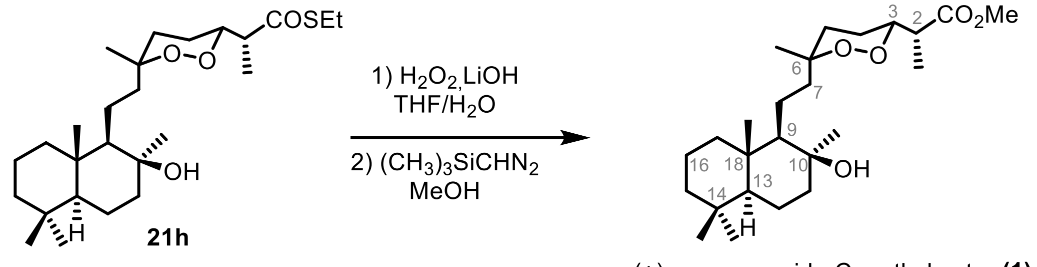

To a solution of **21h** (18 mg, 0.04 mmol, 1 equiv) and H_2O_2 (27 mg, 0.24 mmol, 6 equiv, 30 % in water) in THF (2 mL) and water (1 mL) at 0 °C was added LiOH.H_2O (5 mg, 0.1 mmol, 2.5 equiv). The reaction mixture was then stirred at 0 °C for 1 h. The reaction mixture was poured into a 0.01N aqueous solution of HCl (10 mL). The resulting mixture was extracted with DCM (3x10 mL), and the combined organic layer was concentrated under a vacuum. Formic acid (1 mL) was added to ensure the protonation of the carboxylic acid. After evaporation to dryness under vacuum, anhydrous methanol (1 mL) was added to the residue, and the solution was cooled at 0 °C. Trimethylsilyldiazomethane (2.0 M in hexanes) was added to the stirred solution at 0 °C until a persistent yellow coloration was reached. The reaction mixture was quenched with formic acid and was concentrated on a rotavapor with gentle heating (no more than 30 °C). The residue was purified on a silica gel chromatography to afford **1** (18 mg, 88 %) as a colorless oil.

$[\alpha]^{20}_D$ = +50.1 (*c* 0.198, $CHCl_3$) [litt: $[\alpha]_D$ = –71° (*c* 1.1, $CHCl_3$) on (–)-mycaperoxide C methyl ester][6]

IR (neat) : *v* = 2922, 2866, 1734, 1458, 1373, 754 cm^{-1}.

^{1}H NMR (300 MHz, Chloroform-*d*) δ 4.31 – 4.16 (m, 1H, H_3), 3.68 (s, 3H, OCH_3), 2.55 (p, *J* = 7.3 Hz, 1H, H_2), 1.86 (td, *J* = 13.4, 4.4 Hz, 1H), 1.81 – 1.71 (m, 2H), 1.71 – 1.54 (m, 8H), 1.54 – 1.31 (m, 4H), 1.30 – 1.08 (m, 3H), 1.16 (s, 3H, H_{10}-Me), 1.13 (s, 3H, H_6-Me), 1.13 (d, *J* = 7.0 Hz, 3H, H_2-Me), 0.96 (s, 3H, H_{18}-Me), 0.95 – 0.84 (m, 1H, H_{13}), 0.87 (s, 3H, C_{14}-Meβ), 0.83 (s, 3H, H_{14}-Meα), 0.78 (dd, *J* = 4.4, 2.6 Hz, 1H, H_9).

^{1}H NMR (300 MHz, Chloroform-*d*) δ 174.4 (C_1), 81.4 (C_3), 80.4 (C_6), 73.4 (C_{10}), 59.2 (C_9), 56.2 (C_{13}), 52.0 (O<u>C</u>H_3), 42.8 (C_2), 42.3 (C_{11}), 42.2 (C_{15}), 39.3 (C_{17}), 39.2 (C_{18}), 38.5 (C_7), 33.6 (C_{14}-Meβ), 33.4 (C_{14}), 32.3 (C_5), 30.6 (C_{10}-Me), 24.0 (C_6-Me), 22.8 (C_4), 21.8 (C_{14}-Meα), 19.1 (C_{12}), 18.5 (C_{16}), 18.4 (C_8), 15.3 (C_{18}-Me), 12.8 (C_2-Me).

HRMS (ESI) : calculated for $C_{26}H_{46}O_4SNa$ $[M+Na]^+$: 447.3086, found 477.3102

Table S2: ^{13}C NMR data of reported (-)-mycaperoxide C methyl ester.[6] Comparison with synthetic sample of (+)-mycaperoxide D methyl ester (**1**) and thioester derivatives **21g** and **21h**.

Numbering	21g	Δ 21g–natural product	21h	Δ 21h–natural product	1 (synthetic)	Δ 1–natural product	Mycaperoxide C Methyl ester (natural)
C_1	200.6	26.3	200.7	26.4	174.3	0.0	174.3
C_2	50.9	8.3	50.8	8.2	42.6	0.0	42.6
C_2-Me	13.0	0.4	13.2	0.6	12.6	0.0	12.6
C_3	81.0	-0.2	81.1	-0.1	81.2	0.0	81.2
C_4	22.6	0.0	22.7	0.0	22.6	0.0	22.6
C_5	32.3	0.2	32.2	0.1	32.1	0.0	32.1
C_6	80.1	-0.1	80.2	0.0	80.2	0.0	80.2
C_6-Me	23.8	-0.1	23.8	-0.1	23.9	0.0	23.9
C_7	38.5	0.1	38.5	0.1	38.4	0.0	38.4
C_8	18.2	0.0	18.3	0.1	18.2	0.0	18.2
C_9	59.3	0.3	59.1	0.1	59.1	0.1	59.0
C_{10}	73.3	0.0	73.2	0.0	73.2	0.0	73.2
C_{10}-Me	30.5	0.1	30.6	0.2	30.5	0.1	30.4
C_{11}	42.3	0.2	42.2	0.1	42.1	0.0	42.1
C_{12}	18.8	-0.2	18.9	-0.1	19.0	0.0	19.0
C_{13}	56.0	0.0	56.1	0.1	56.0	0.0	56.0
C_{14}	33.3	0.1	33.2	0.0	33.2	0.0	33.2
C_{14}-Me α	21.7	0.0	21.7	0.0	21.6	0.0	21.6
C_{14}-Me β	33.4	0.0	33.4	0.0	33.4	0.0	33.4
C_{15}	42.2	0.1	42.1	0.1	42.0	0.0	42.0
C_{16}	18.3	0.0	18.4	0.1	18.3	0.0	18.3
C_{17}	39.1	-0.1	39.3	0.0	39.2	0.0	39.2
C_{18}	39.0	0.0	39.1	0.1	39.1	0.0	39.0
C_{18}-Me	15.1	-0.1	15.2	0.0	15.2	0.0	15.2
$\underline{CH_n}$X(CO)	23.3	-28.6	23.3	-28.6	51.9	0.0	51.9
CH3CH2S	14.5	-	14.5	-	-	-	-

Reported values in the table with calibration at 77.00 ppm for $CDCl_3$ solvent peak, for comparison with the data of the natural products. Higher differences of chemical shift (ppm) with natural products are highlighted in from light yellow (Δ = 0.02 ppm), light orange (Δ = 0.03 ppm), orange (0.04 <Δ<1.0 ppm), light red (1.0 <Δ<10 ppm), scarlet (Δ ≥ 10 ppm).

Compound **22**

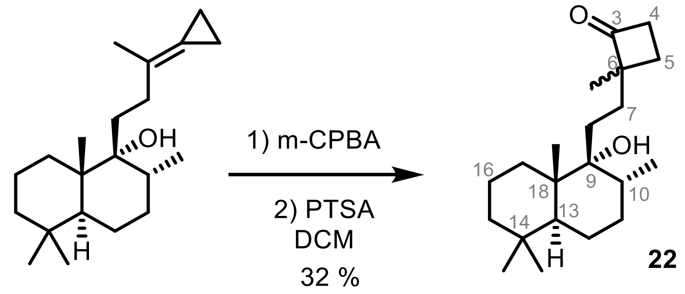

To a solution of **10a** (2 g, 6.6 mmol,1 equiv) in DCM (26.4 mL, 0.25 M) was added *m*-CPBA (1.73 g, 7.26 mmol, 1.1 equiv) by portions at 0 °C. After 30 min of stirring at r.t., the reaction mixture was quenched with a 10% aqueous solution of $Na_2S_2O_3$, and the mixture was stirred vigorously for 15 min. The biphasic mixture was poured into a saturated aqueous solution of Na_2CO_3 and extracted with DCM (2 x 200 mL). The combined organic extracts were dried over $MgSO_4$ and concentrated under reduced pressure. The residue was diluted with DCM (26 mL), and PTSA (114 mg, 0.1 equiv, 0.66 mmol) was added. After 2.5 h of stirring, the reaction mixture was quenched with a saturated aqueous solution of Na_2CO_3 and was extracted with DCM (2 x 200 mL). The combined organic layers were dried over $MgSO_4$ and concentrated under a vacuum. The residue was purified by flash chromatography on silica gel to afford **22** (680 mg, 32%) as yellow oil as a 2:1 mixture of diastereomers.

IR (neat) : *v* = 2930, 2866, 1458, 1386, 1369, 910, 756 cm^{-1}.

^{1}H NMR (300 MHz, $CDCl_3$*, 2:1 mixture of diastereomers*) δ 2.96 – 2.88 (m, 2H, H_4), 1.93 – 1.78 (m, 1H, H_{5a}), 1.76 – 1.65 (m, 3H), 1.65 – 1.15 (m, 14H), 1.12 (s, 3H, H_6-Me), 1.04 (dd, *J* = 13.2, 4.2 Hz, 1H, H_{13}), 0.86 (s, 2H, C_{18}-Me, *major diastereomer*), 0.85 (s, 1H, H_{18}-Me, *minor diastereomer*), 0.79 (s, 2H, H_{14}-Meβ, *major diastereomer*), 0.78 (s, 1H, C_{14}-CH_3β, *minor diastereomer*), 0.78 (d, *J* = 6.1 Hz, 2H, H_{10}-Me, *major diastereomer*), 0.76 (d, *J* = 6.1 Hz, 1H, H_{10}-Me, minor *diastereomer*), 0.76 (s, 3H, H_{14}-Meα).

^{13}C NMR (75 MHz, $CDCl_3$*, 2:1 mixture of diastereomers*) δ [216.2*, 216.1] (C_3), [76.9, 76.9] (C_9), 64.5 (C_6), 46.5 (C_{13}), 43.5 (C_{18}), [42.3, 42.3*] (C_4), 41.8 (C_{15}), [36.6*, 36.5] (C_{10}), 33.9 (C_{14}-Meβ), 33.4 (C_{14}), [32.3, 32.2*] (C_8), [32.1, 32.0*] (C_{17}), 31.4 (C_{11}), [29.1, 29.0*] (C_7), [24.2*, 24.2] (C_5), 22.2 (C_{14}-Meα), 21.7 (C_{12}), [20.7, 20.5*] (C_6-Me), 18.8 (C_{16}), 16.5 (C_{10}-Me), [16.5, 16.4*] (C_{18}-Me). * = minor isomer

HRMS (ESI) : calculated for $C_{21}H_{36}O_2Na$ $[M+Na]^+$: 343.2613, found 343.2627

Compound **23**

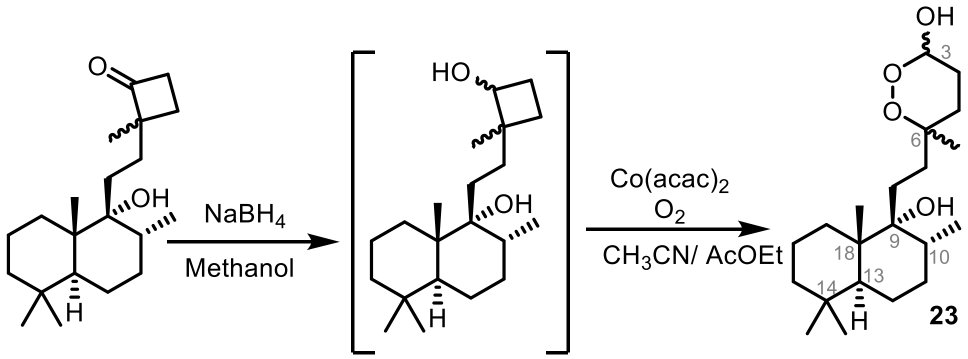

To a solution of **22** (545 mg, 1.7 mmol, 1 equiv) in methanol (6.8 mL) at 0 °C was added $NaBH_4$ (64 mg, 1.7 mmol, 1 equiv) in 3 portions over 10 minutes. The reaction was then stirred at rt for 1 h and was filtered on a pad of silica gel with ethyl acetate (3x100 mL). The filtrate was concentrated under a vacuum. Acetonitrile (17 mL) was added to the residue, followed by the addition of $Co(acac)_2$ (22 mg, 0.085 mmol, 0.05 equiv). The brownish reaction mixture was lightly bubbled with O_2 for 5 min, and the oxygen atmosphere was maintained with a rubber balloon. The reaction was then heated to 40 °C with an oil bath, and the color turned slowly to intense green. The reaction was monitored by TLC, and after 2 h of reaction under these conditions, the reaction mixture was concentrated on a rotavapor with gentle heating (no more than 30 °C). The residue was purified on a silica gel to afford **24** (511 mg, 85 %) as a white solid in 2:2:3:3 mixture of diastereomers. (*cis*:*trans* dioxane ratio = 2:3)

mp = 49–50 °C

IR (neat) : *v* = 3423, 2934, 2868, 1458, 1375, 1142, 991, 756 cm^{-1}.

^{1}H NMR (300 MHz, $CDCl_3$, 2:2:3:3 *mixture of diastereomers*) δ 5.30 – 5.17 (m, 1H, H_3), 3.66 (brs, 0.2H, OH, *1 cis diastereomer*), 3.49 (brs, 0.8H, OH, 3 *diastereomers*), 2.09 – 1.82 (m, 2H), 1.82 – 1.59 (m, 5H), 1.59 – 1.38 (m, 11H), 1.37 – 1.29 (m, 2H), 1.29 – 1.21 (m, 1H, OH), 1.26 (s, 0.9H, H_6-Me, *1 trans diastereomer*), 1.24 (s, 0.9H, H_6-Me, *1 trans diastereomer*), 1.20 –1.06 (m, 1H), 1.15 (s, 1.2H, H_6-Me, *2 cis diastereomers*), 0.93 (s, 0.9H, H_{18}-Me, *1 trans diastereomer*), 0.93 (s, 1.5H, H_{18}-Me, *1 trans+ 1 cis diastereomers*), 0.92 (s, 0.6H, H_{18}-Me, *1 cis diastereomer*), 0.90 – 0.83 (m, 6H, H_{10}-Me & H_{14}-Meβ), 0.82 (s, 3H, H_{14}-Meα).

^{13}C NMR (75 MHz, $CDCl_3$, *2:2:3:3 mixture of diastereomers*) δ [96.6*, 96.5, 96.5] (C_3), [81.0, 80.9*, 80.8*] (C_6), [77.2, 77.1] (C_9), 46.4 (C_{13}), [43.6*, 43.5] (C_{18}), 41.8 (C_{15}), [36.8*, 36.7, 36.6*, 36.5] (C_{10}), [34.3, 34.2, 32.9, 32.6] (br, C_7) 33.9 (C_{14}-Meβ), 33.4 (C_{14}), [32.2, 32.2, 32.1*, 32.0*] (C_{17}), [31.5*, 31.5] (C_{11}), [30.0, 29.5*, 29.2*] (br, C_5), [27.9, 27.3*] (C_8), [25.9*, 25.8, 25.5] (C_4), 22.1 (C_{14}-Meα), [22.1* 22.0, 21.6*, 21.5] (C_6-Me), [21.8*, 21.7] (C_{12}), 18.8 (C_{16}), [16.7, 16.6, 16.5, 16.5] (C_{10}-Me), [16.5, 16.4, 16.3] (C_{18}-Me). * = minor *cis* isomers when identifiable

HRMS (ESI) : calculated for $C_{21}H_{38}O_4Na$ $[M+Na]^+$: 377.2668, found 377.2663

Compound **24**

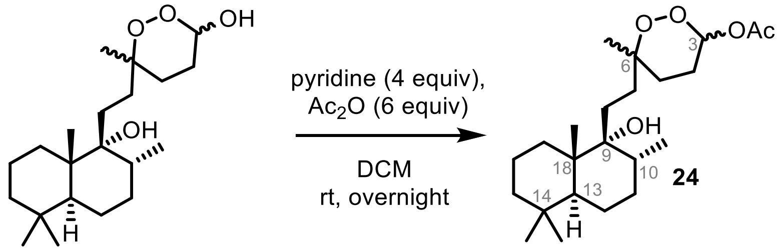

To a solution of **23** (496 mg, 1.4 mmol, 1 equiv) in DCM (2.8 mL), were added AC_2O (0.8 mL, 8.4 mmol, 6 equiv) and pyridine (0.46 mL, 5.6 mmol, 4 equiv). The reaction mixture was stirred overnight at rt and was poured into an Erlenmeyer containing a saturated $NaHCO_3$ solution to be quenched. The new resulting mixture was stirred vigorously for 30 min at rt, and the aqueous layer was extracted 3 times with 30 mL of dichloromethane. The combined organic phases were washed 2 times with 10% citric aqueous solution (30 mL), dried over $MgSO_4$, filtered, and concentrated on a rotavapor under vacuum with gentle heating (no more than 30 °C). The residue was purified on a silica gel column chromatography to afford **24** (512 mg, 92 %) as colorless oil in about 1:1:1:1 mixture of diastereomers.

IR (neat): v = 2934, 2868, 1458, 1373, 1229, 999, 968, 755 cm^{-1}.

^{1}H NMR (300 MHz, $CDCl_3$, about *1:1:1:1 mixture of diastereomers*) δ 6.20 (t, J = 4.5 Hz, 1H, H_3), 2.12 (s, 0.75H, OAc, *1 diastereomer*), 2.12 (s, 2.25H, OAc, 3 *diastereomers*), 2.11 – 1.80 (m, 2H), 1.80 – 1.67 (m, 2H), 1.67 – 1.35 (m, 13H), 1.34 (s, 1.5H, H_6-Me, *2 trans diastereomers*), 1.35 – 1.20 (m, 3H), 1.20 – 1.06 (m, 1H), 1.12 (s, 1.5H, H_6-Me, *2 cis diastereomers*), 0.93 (s, 1.5H, H_{18}-Me, *2 diastereomers*), 0.93 (s, 1.5H, H_{18}-Me, *2 diastereomers*), 0.88 (d, J = 6.7 Hz, 0.75H, H_{10}-Me, 1 *diastereomer*), 0.87 (s, 3H, H_{14}-Meβ), 0.86 (d, J = 6.7 Hz, H_{10}-Me, 2.25H, *3 diastereomers*), 0.83 (s, 3H, H_{14}-Meα).

^{13}C NMR (75 MHz, $CDCl_3$, about *1:1:1:1 mixture of diastereomers*) δ [170.1, 170.0] (OC=O), [95.0, 94.8] (C_3), [81.5, 81.5, 81.4, 81.3] (C_6), [77.1, 77.0, 76.9] (C_9), 46.5 (C_{13}), 43.6 (C_{18}), 41.9 (C_{15}), [36.8, 36.7, 36.5] (C_{10}), [34.9, 34.9, 33.0, 32.9] (C_7), 33.9 (C_{14}-Meβ), 33.5 (C_{14}), 32.2 (C_{17}), [31.6, 31.5] (C_{11}), [29.7, 29.6, 28.5, 28.4] (C_5), [28.1, 28.0, 27.3, 27.2] (C_8), [23.2, 22.9] (C_4), 22.2 (C_{14}-Meα), [21.9, 21.8, 21.6] (C_6-Me), 21.8 (C_{12}), 21.3 ($\underline{CH_3}$C=O), 18.8 (C_{16}), [16.6, 16.5, 16.4, 16.4] (C_{10}-Me and C_{18}-Me).

HRMS (ESI) : calculated for $C_{23}H_{40}O_5Na$ $[M+Na]^+$: 419.2773, found 419.2768

Compound **25**

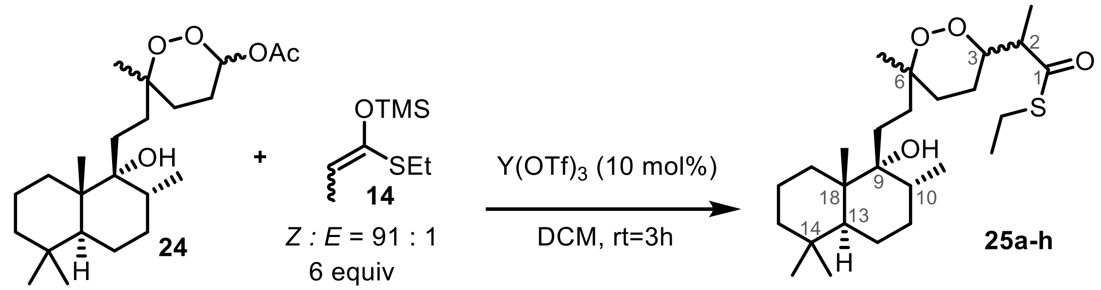

To a solution of **24** (238 mg, 0.6 mmol, 1 equiv) and thioketal **14**[5] (685 mg, 3.6 mmol, 6 equiv) in dry DCM (1.2 mL, 0.5 M) was added $Y(OTf)_3$ (32 mg, 0.06 mmol, 0.1 equiv). The reaction mixture was stirred at rt for 3 h and was poured into a saturated aqueous solution of $NaHCO_3$. The aqueous phase was extracted with DCM (3x20mL), and the combined organic layers were washed with brine, dried over $MgSO_4$, and concentrated on a rotavapor with gentle heating (no more than 30 °C). The residue was purified on a silica gel chromatography to afford two fractions: F1 (142 mg, 52 %) containing the five diastereomers **25a-d** and **25g** as a colorless oil, and F2 (96 mg, 35 %) containing the three diastereomers **25e-f and 25h** as a colorless oil. The global yield was 87 %. The selectivity of the reaction was about 60:40 in favor of *cis*-1,2-dioxanes and 1:1 for the 2,3-*anti*/*syn* relationship.

The fraction F2 was further purified by preparative HPLC [Column: XSELECT, gradient H_2O/MeOH 85:15 to 10:90 over 25 min]. From 73 mg of fraction F2, was obtained **25h** (23 mg) and a 53:47 mixture of **25e** and **25f** (30 mg) (73% yield for the preparative HPLC purification) as colorless oils.

Fraction F1 (mixture of five diastereomers) ***25a-d+25g***

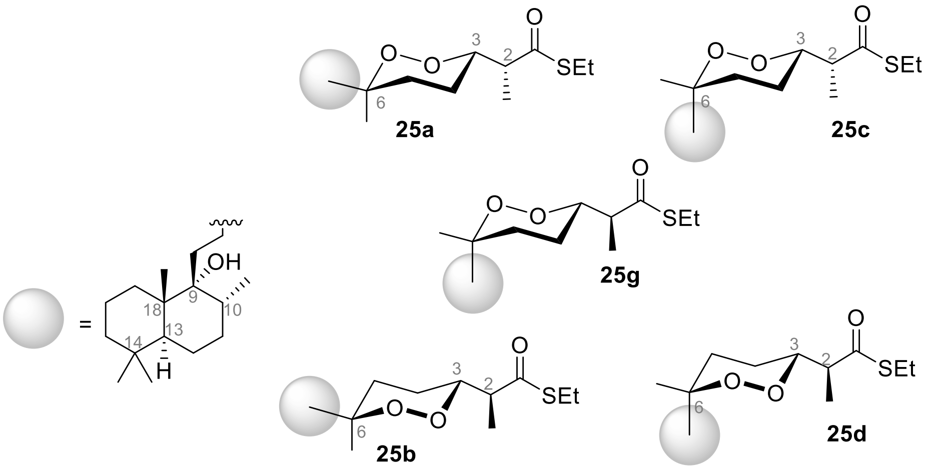

IR (neat) : *v* = 2931, 1683, 1456, 1373, 964, 754 cm^{-1}.

^{1}H NMR (300 MHz, $CDCl_3$, *about 1:1:1:1:1 mixture of diastereomers*) δ 4.14 – 3.91 (m, 1H, H_3), 2.85 (q, *J* = 7.4 Hz, 2H, $CH_3\underline{CH_2}S$), 2.83 – 2.63 (m, 1H, H_2), 2.09 – 1.85 (m, 1H), 1.83 – 1.55 (m, 6H), 1.55 – 1.33 (m, 9H), 1.32 – 1.18 (m, 10H), 1.18 – 1.02 (m, 4H), 0.92 (s, 1.8H, H_{18}-Me, *3 diastereomers*), 0.89

(s, 1.2H, H_{18}-Me, *2 diastereomers*), 0.86 (d, *J* = 6.6 Hz, 0.6H, H_{10}-Me, 1 *diastereomer*), 0.86 – 0.82 (m, 5.4H, 4 *diastereomers for* H_{10}-Me & H_{14}-Meβ), 0.81 (s, 1.8H, H_{14}-Meα, *3 diastereomers*), 0.80 (s, 1.2H, H_{14}-Meα, *2 diastereomers*).

^{13}C NMR (75 MHz, $CDCl_3$, *about 1:1:1:1:1 mixture of diastereomers*) δ [200.9, 200.8] (C_1), [81.5, 81.4, 81.3] (C_3), [80.7, 80.7, 80.5, 80.2] (C_6), [77.1, 77.1, 77.0, 76.8, 76.8] (C_9), [51.5, 51.4, 51.0] (C_2), [46.4, 46.3] (C_{13}), [43.5, 43.5] (C_{18}), [41.9, 41.9, 41.8] (C_{15}), [36.7, 36.6, 36.6] (C_{10}), [35.5, 33.1, 32.6] (br, C_7), 33.9 (C_{14}-Meβ), 33.4 (C_{14}), [32.1, 32.0, 31.9] (C_{17}), [31.5, 31.4] (C_{11}), [31.3, 31.0, 30.4, 30.4] (br, C_5), [27.6, 27.5, 27.3, 27.3] (C_8), [23.9, 21.0] (C_6-Me), [23.6, 22.9] (C_4), 23.4 ($CH_3\underline{CH_2}S$), 22.1 (C_{14}-Meα), [21.8, 21.7] (C_{12}), [18.8, 18.8, 18.8] (C_{16}), [16.6, 16.5, 16.4, 16.3] (C_{10}-Me and C_{18}-Me), [14.9, 14.7, 13.3] (C_2-Me), 14.7 ($\underline{CH_3}CH_2S$).

HRMS (ESI) : calculated for $C_{26}H_{46}O_4SNa$ $[M+Na]^+$: 477.3015, found 477.3012

*Compound **25h***

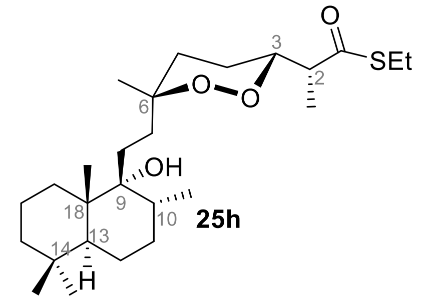

25h

$[\alpha]^{20}_D$ = +38.4 (*c* 0.5, $CHCl_3$)

IR (neat) : *ν* = 2932, 2868, 1684, 1456, 1375, 964, 752 cm^{-1}.

^{1}H NMR (300 MHz, $CDCl_3$) δ 4.27 (td, *J* = 8.1, 3.9 Hz, 1H, H_3), 2.89 (p, *J* = 10.3, 7.3 Hz, 1H, $CH_3\underline{CH_2}S$), 2.87 (dq, *J* = 10.3, 7.3 Hz, $CH_3\underline{CH_2}S$), 2.71 (p, *J* = 7.2 Hz, 1H, H_2), 2.03 (td, *J* = 12.5, 4.6 Hz, 1H), 2.03 – 1.24 (m, 21H), 1.24 (t, *J* = 7.3 Hz, 3H, $\underline{CH_3}CH_2S$), 1.13 (d, *J* = 7.1 Hz, 3H, H_2-Me), 1.09 (s, 3H, H_6-Me), 0.95 (s, 3H, H_{18}-Me), 0.90 (d, *J* = 6.7 Hz, 3H, H_{10}-Me), 0.87 (s, 3H, H_{14}-Meβ), 0.84 (s, 3H, H_{14}-Meα).

^{13}C NMR (75 MHz, $CDCl_3$) δ 200.9 (C_1), 81.3 (C_3), 80.3 (C_6), 77.2 (C_9), 51.1 (C_2), 46.5 (C_{13}), 43.6 (C_{18}), 41.9 (C_{15}), 36.7 (C_{10}), 33.9 (C_{14}-Meβ), 33.5 (C_{14}), 33.1 (C_7), 32.2 (C_{17}), 31.6 (C_{11}), 30.8 (C_5), 27.6 (C_8), 23.8 (C_6-Me), 23.5 ($CH_3\underline{CH_2}S$), 22.9 (C_4), 22.2 (C_{14}-Meα), 21.8 (C_{12}), 18.9 (C_{16}), 16.4 (C_{10}-Me and C_{18}-Me), 14.7 ($\underline{CH_3}CH_2S$), 13.4 (C_2-Me).

HRMS (ESI) : calculated for $C_{26}H_{46}O_4SNa$ $[M+Na]^+$: 477.3016, found 477.3009.

*Compound **25e-f***

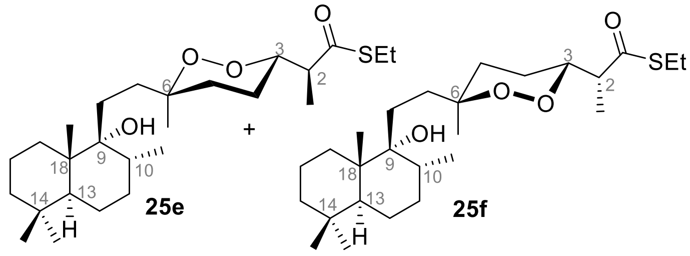

[α]$^{20}_{D}$ = +4.7 (*c* 1.265, $CHCl_3$)

IR (neat) : *v* = 2932, 2868, 1684, 1456, 1375, 964, 752 cm^{-1}.

^{1}H NMR (300 MHz, $CDCl_3$, *about 1:1 mixture of diastereomers*) δ 4.27 (q, *J* = 6.3 Hz, 1H, H_3), 2.92 (dq, *J* = 13.3, 7.3 Hz, 1H, $CH_3\underline{CH_2}S$), 2.87 (dq, *J* = 13.3, 7.3 Hz, 1H, $CH_3\underline{CH_2}S$), 2.76 (p, *J* = 7.7 Hz, 1H, H_2), 1.81 – 1.59 (m, 6H), 1.59 – 1.33 (m, 11H), 1.30 (s, 3H, H_6-Me), 1.37 – 1.20 (m, 2H), 1.24 (t, *J* = 7.4 Hz, 3H, $\underline{CH_3}CH_2S$), 1.14 (d, *J* = 7.1 Hz, 3H, H_2-Me), 1.19 – 1.04 (m, 2H), 0.91 (s, 3H, H_{18}-Me), 0.88 – 0.83 (m, 3H, H_{10}-Me), 0.86 (s, 3H, H_{14}-Meβ), 0.82 (s, 3H, H_{14}-Meα).

^{13}C NMR (75 MHz, $CDCl_3$, ***25e*:*25f*** *=53:47 mixture of diastereomers*) δ 201.0 (C_1), 81.7 (C_3), [80.6*, 80.6] (C_6), 76.9 (C_9), 51.1 (C_2), 46.5 (C_{13}), 43.6 (C_{18}), 41.9 (C_{15}), [36.7*, 36.6] (C_{10}), [36.5, 36.4*] (C_7), 33.9 (C_{14}-Meβ), 33.5 (C_{14}), [32.7, 32.6*] (C_5), 32.2 (C_{17}), 31.5 (C_{11}), [27.4, 27.3*] (C_8), 23.5 ($CH_3\underline{CH_2}S$), 22.9 (C_4), 22.2 (C_{14}-Meα), 21.8 (C_{12}), 20.5 (C_6-Me), 18.8 (C_{16}), [16.6*, 16.5] (C_{10}-Me), [16.4, 16.4*] (C_{18}-Me), 14.7 ($\underline{CH_3}CH_2S$), [13.5*, 13.5] (C_2-Me). * = minor isomer **25f**

HRMS (ESI) : calculated for $C_{26}H_{46}O_4SNa$ [M+Na]$^+$: 477.3019, found 477.3009.

Compounds **26e/4** :

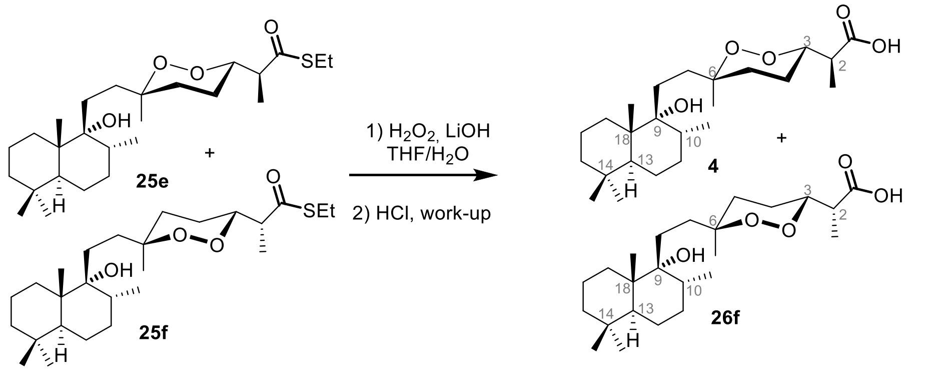

To a solution of the mixture of **25e-f** (46 mg, 0.1 mmol, 1 equiv) and H_2O_2 (68 mg, 6 equiv, 0.6 mmol, 30 % in water) in THF (4 mL) and water (2 mL) at 0 °C was added LiOH (6 mg, 0.25 mmol, 2.5 equiv). The reaction mixture was then stirred at 0 °C for 1 h. After the disappearance of the starting material, the reaction mixture was poured in 0.01 N aqueous solution of HCl (10 mL). The medium was extracted with DCM (about 3x10 mL), and the combined organic layers were concentrated under a vacuum to afford the inseparable mixture of **26f** and **4** (44 mg, 100%) as a yellow liquid. No further purification was performed.

IR (neat) : *v* = 2927, 2866, 1709, 1458, 1375, 1375, 754 cm^{-1}.

^{1}H NMR (300 MHz, $CDCl_3$) δ 11.52 – 6.85 (brm, 1H, COOH), 4.32 – 4.08 (m, 1H, H_3), 2.72 – 2.42 (m, 1H, H_2), 1.96 – 1.59 (m, 6H), 1.57 – 1.33 (m, 11H), 1.25 (s, 3H, H_6-Me), 1.34 – 1.08 (m, 4H), 1.18 (d, *J* = 5.9 Hz, 3H, H_2-Me), 0.91 (s, 3H, H_{18}-Me), 0.86 (s, 3H, H_{14}-Meβ), 0.84 (d, *J* = 7.5 Hz, 3H, H_{10}-Me), 0.82 (s, 3H, H_{14}-Meα).

^{13}C NMR (75 MHz, $CDCl_3$, **26e**:**25f** =57:43 *mixture of diastereomers*) δ [179.7, 179.6*] (C_1), 81.5 (C_3), [80.7*, 80.6] (C_6), 77.1 (C_9), 46.5 (C_{13}), [43.5*, 43.5] (C_{18}), 42.8 (C_2), 41.9 (C_{15}), 36.6 (C_{10}), [36.4, 36.3*] (C_7), 33.9 (C_{14}-Meβ), 33.4 (C_{14}), [32.6, 32.5] (C_5), 32.1 (C_{17}), 31.5 (C_{11}), [27.3, 27.2] (C_8), 22.8 (C_4), 22.1 (C_{14}-Meα), 21.7 (C_{12}), [20.5*,20.5] (C_6-Me), 18.8 (C_{16}), [16.5*, 16.5] (C_{10}-Me), [16.4, 16.4] (C_{18}-Me), 12.8 (C_2-Me).* = minor isomer **4** when a differentiation can apply

HRMS (ESI) : calculated for $C_{24}H_{42}O_5Na$ $[M+Na]^+$: 433.2930, found 433.2936

<u>Compounds **27e,f,h**</u>

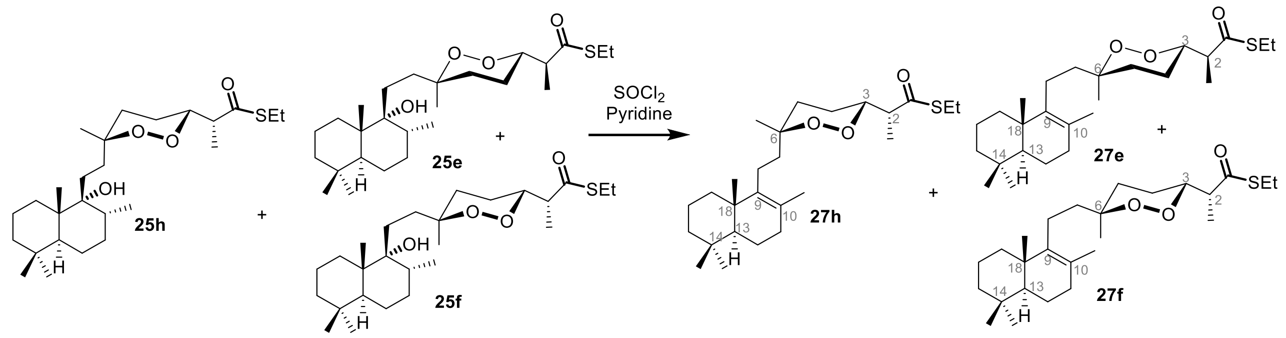

To a stirred solution of fraction F2 containing the three diastereomers **25e-f and 25h** (136 mg, 0.3 mmol, 1 equiv) in pyridine (3 mL) at 0 °C was added $SOCl_2$ (0.3 mL, 4.5 mmol, 15 equiv) in three portions. The reaction mixture was warmed to rt and stirred at this temperature for 1 h. The mixture was poured into a saturated $NaHCO_3$ solution (10 mL), and the aqueous phase was extracted with DCM (3x20 mL). The combined organic layers were washed with 10% citric acid solution (10 mL), dried over $MgSO_4$, and concentrated under a vacuum. The crude product was purified by flash chromatography on silica gel (petroleum ether: toluene, from 80:20 to 20:80) to afford by order of elution **27e-f,** as a light-yellow oil (32 mg, 24 %, ***27e:27f*** = 53:47) followed by **27h,** as light yellow oil (26 mg, 19 %). The global yield was 43 %.

*Compounds **27e-f***

$[\alpha]^{20}_D$ = +25.3 (*c* 0.91, $CHCl_3$)

IR (neat) : *v* = 2930, 2866, 1686, 1456, 1375, 1373, 962, 752 cm^{-1}.

^{1}H NMR (300 MHz, $CDCl_3$, *about 1:1 mixture of diastereomers*) δ 4.37 – 4.20 (m, 1H, H_3), 2.92 (dq, *J* = 13.4, 7.5 Hz, 0.5H, CH_3<u>CH_2</u>S, 1 diastereomer), 2.85 (dq, *J* = 13.4, 7.5 Hz, 1H, CH_3<u>CH_2</u>S, 1 diastereomer), 2.76 (2p, *J* = 7.3 Hz, 1H, CH_3<u>CH_2</u>S, *2 diastereomers*), 2.14 – 1.73 (m, 5H), 1.70 – 1.20 (m, 12H), 1.54 (s, 3H, H_{10}-Me), 1.34 (s, 3H, H_6-Me), 1.24 (t, *J* = 7.4 Hz, 3H, <u>CH_3</u>CH_2S), 1.15 (d, *J* = 7.4 Hz, 3H, H_2-Me), 1.15 – 1.01 (m, 2H), 0.92 (s, 1.5H, *1 diastereomer*, H_{18}-Me), 0.92 (s, 1.5H, *1 diastereomer*, H_{18}-Me), 0.87 (s, 3H, H_{14}-Meβ), 0.82 (s, 3H, H_{14}-Meα).

^{13}C NMR (75 MHz, $CDCl_3$, ***27e:27f*** = 53:47 *mixture of diastereomers*) δ 200.9 (C_1), [140.0, 139.9*] (C_9), [126.3, 126.2*] (C_{10}), 81.7 (C_3), 80.5 (C_6), 52.1 (C_{13}), 51.1 (C_2), 42.0 (C_{15}), 40.6 (C_7), 39.3 (C_{18}), [37.3*, 37.2] (C_{17}), 33.8 (C_{11}), 33.5 (C_{14}+C_{14}-Meβ), [32.4, 32.3*] (C_5), 23.5 (CH_3<u>CH_2</u>S), 23.0 (C_4), 21.9

(C_{14}-Meα), 21.2 (C_8-Me), 20.3 (C_6-Me+C_{18}-Me), 19.6 (C_{10}-Me), 19.2 (C_{12}+C_{16}), 14.7 ($\underline{CH_3}CH_2S$), 13.4 (C_2-Me). *= minor isomer **27f**

HRMS (ESI) : calculated for $C_{26}H_{44}O_3SNa$ $[M+Na]^+$: 459.2909, found 459.2906

*Compound **27h***

[α]$^{20}_D$ = +62.6 (*c* 1.48, $CHCl_3$)

IR (neat) : *v* = 2931, 2866, 1686, 1456, 1373, 962, 752 cm^{-1}.

^{1}H NMR (300 MHz, $CDCl_3$) δ 4.28 (m, 1H, H_3), 2.90 (dq, *J* = 13.3, 7.5 Hz, 1H, $CH_3\underline{CH_2}S$), 2.85 (dq, *J* = 13.3, 7.5 Hz, 1H, $CH_3\underline{CH_2}S$), 2.74 (p, *J* = 7.3 Hz, 1H, H_2), 2.21 – 2.07 (m, 1H), 2.07 – 1.32 (m, 16H), 1.61 (s, 3H, H_{10}-Me), 1.24 (t, *J* = 7.5 Hz, 1H, $\underline{CH_3}CH_2S$), 1.23 – 1.05 (m, 4H), 1.13 (d, *J* = 7.1 Hz, 3H, H_2-Me), 1.13 (s, 3H, H_6-Me), 0.96 (s, 3H, H_{18}-Me), 0.89 (s, 3H, H_{14}-Meβ), 0.84 (s, 3H, H_{14}-Meα).

^{13}C NMR (75 MHz, $CDCl_3$) δ 200.9 (C_1), 140.1 (C_9), 126.3 (C_{10}), 81.3 (C_3), 80.2 (C_6), 52.1 (C_{13}), 51.1 (C_2), 42.0 (C_{15}), 39.3 (C_{18}), 37.3 (C_{17}), 35.3 (C_7), 33.9 (C_{11}), 33.5 (C_{14}+C_{14}-Meβ), 32.9 (C_5), 23.7 (C_6-Me), 23.5 ($CH_3\underline{CH_2}S$), 22.8 (C_4), 21.9 (C_{14}-Meα), 21.6 (C_8-Me), 20.3 (C_{18}-Me), 19.7 (C_{10}-Me), 19.3 (C_{12}+C_{16}), 14.7 ($\underline{CH_3}CH_2S$), 13.2 (C_2-Me).

HRMS (ESI) : calculated for $C_{26}H_{44}O_3SNa$ $[M+Na]^+$: 459.2909, found 459.2910

<u>Compounds **28f/3**</u>

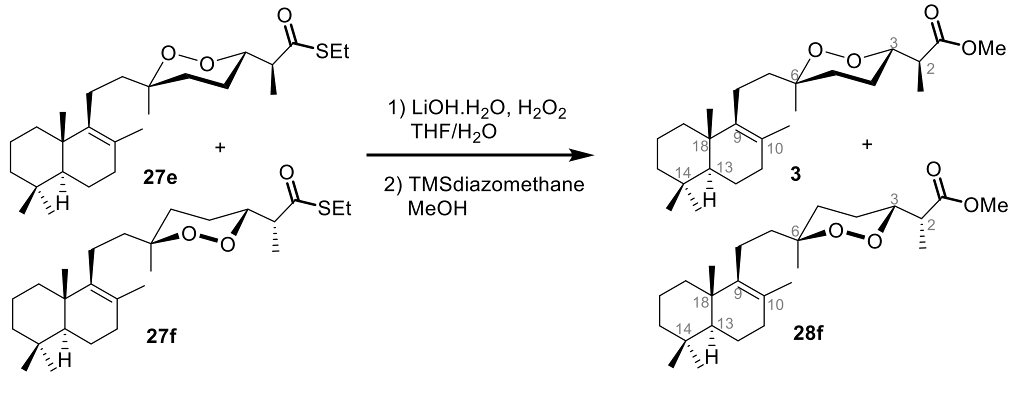

To a solution of the mixture of **27e/27f** (21 mg, 0.05 mmol, 1 equiv) and H_2O_2 (34 mg, 0.3 mmol, 30 % in water, 6 equiv) in THF (2 mL) and water (1 mL) at 0 °C was added $LiOH.H_2O$ (5 mg, 0.13 mmol, 2.5 equiv). The reaction mixture was then stirred at 0 °C for 1 h. After the disappearance of the starting material, the reaction mixture was poured into a 0.1 N aqueous solution of HCl (10 mL) and was extracted with DCM (3x10 mL). The combined organic layers were concentrated under a vacuum. Formic acid (1 mL) was added to ensure the protonation of the carboxylic acid. After evaporation to dryness under vacuum, anhydrous methanol (1 mL) was added to the residue, and the solution was cooled at 0 °C. Trimethylsilyl diazomethane (2.0 M in hexane) was added to the stirred solution at 0 °C until getting persistent yellow coloring. The reaction mixture was quenched with formic acid and was concentrated on a rotavapor with gentle heating (no more than 30 °C). The crude was purified on a silica gel column to afford mycaperoxide G (**3)** in a mixture with isomer **28f** (12 mg, 60 %, ***28e:3*** = 53:47) as a colorless oil.

IR (neat) : *v* = 2931, 2866, 1740, 1458, 1375, 752 cm^{-1}.

^{1}H NMR (400 MHz, $CDCl_3$, *about 1:1 mixture of diastereomers*) δ 4.29 – 4.19 (m, 1H, H_3), 3.70 (s, 3H, OMe), 2.58 (2p, *J* = 7.4 Hz, 1H, $CH_3\underline{CH_2}S$, *2 diastereomers*), 2.13 – 1.84 (m, 4H), 1.84 – 1.74 (m, 1H), 1.73 – 1.19 (m, 11H), 1.54 (s, 3H, H_{10}-Me), 1.34 (s, 3H, H_6-Me), 1.15 (d, *J* = 7.4 Hz, 3H, H_2-Me), 1.19 – 1.04 (m, 3H), 0.93 (s, 1.5H, H_{18}-Me, *1 diastereomer*), 0.92 (s, 1.5H, H_{18}-Me, *1 diastereomer*), 0.87 (s, 3H, H_{14}-Meβ), 0.82 (s, 3H, H_{14}-Meα).

^{13}C NMR (75 MHz, $CDCl_3$, ***28e:3*** = 53:47 *mixture of diastereomers*) δ 174.59 (C_1), [140.0, 140.0*] (C_9), [126.35, 126.19*] (C_{10}), 81.8 (C_3), 80.5 (C_6), 52.1 (C_{13}) 52.0 (OMe), 42.9(C_2), 42.0 (C_{15}), 40.6 (C_7), 39.3 (C_{18}), [37.3*, 37.2] (C_{17}), 33.8 (C_{11}), 33.5 (C_{14}+C_{14}-Meβ), [32.4, 32.3*] (C_5), 22.9 (C_4), 21.9 (C_{14}-Meα), 21.3 (C_8-Me), 20.3 (C_6-Me+C_{18}-Me), 19.6 (C_{10}-Me), 19.2 (C_{12}+C_{16}), 12.9 (C_2-Me). * = minor isomer **27f**

HRMS (ESI) : calculated for $C_{25}H_{42}O_4Na$ $[M+Na]^+$: 429.2981, found 459.2981

Table S3: ^{13}C NMR data of reported mycaperoxides B,[7] and mycaperoxide G methyl ester.[8] Comparison with synthetic samples containing **26e/f** and **28e/f**.

Numbering	Mycaperoxide B (natural)	26e = 4	26f	Δ 4 – Myca. B	Δ 26f – Myca. B	Mycaperoxide G methyl ester (natural)	28e	28e = 3	Δ 28e – Myca. G Me ester	Δ 3 – Myca. G Me ester
C_1	178.7	179.5	179.5	0.8	0.8	174.5	174.4	174.4	-0.1	-0.1
C_2	42.6	42.6	42.6	0.0	0.0	42.7	42.8	42.8	0.0	0.0
C_2-Me	12.5	12.6	12.6	0.1	0.1	12.8	12.8	12.8	-0.1	-0.1
C_3	81.3	81.4	81.4	0.1	0.1	81.7	81.7	81.7	0.0	0.0
C_4	22.5	22.6	22.6	0.1	0.1	22.8	22.8	22.8	0.0	0.0
C_5	32.4	32.4	32.4	0.0	0.0	32.2	32.2	32.1	0.0	-0.1
C_6	80.4	80.5	80.5	0.1	0.1	80.4	80.4	80.4	-0.1	-0.1
C_6-Me	20.1	20.3	20.3	0.2	0.2	20.1 [a]	20.1	20.1	0.0	0.0
C_7	36.2	36.2	36.1	0.0	-0.1	40.4	40.4	40.4	0.0	0.0
C_8	27	27.1	27.1	0.1	0.1	21.1[a]	21.1	21.1	0.0	0.0
C_9	77	76.9	76.9	-0.1	-0.1	139.7	139.9	139.7	0.2	0.0
C_{10}	36.4	36.4	36.4	0.0	0.0	126	126.2	126.0	0.2	0.0
C_{10}-Me	16.2	16.3	16.4	0.1	0.2	19.4	19.4	19.4	0.0	0.0
C_{11}	31.2	31.3	31.3	0.1	0.1	33.6	33.7	33.7	0.1	0.1
C_{12}	21.6	21.6	21.6	0.0	0.0	19	19.1	19.1	0.1	0.1
C_{13}	46.2	46.3	46.3	0.1	0.1	51.9	51.9	51.9	0.0	0.0
C_{14}	33.2	33.3	33.3	0.0	0.0	33	33.3	33.3	0.3	0.3
C_{14}-Meα	21.9	22.0	22.0	0.1	0.1	21.7	21.7	21.7	0.0	0.0
C_{14}-Meβ	33.7	33.7	33.7	0.0	0.0	33	33.3	33.3	0.3	0.3
C_{15}	41.7	41.7	41.7	0.0	0.0	41.7	41.8	41.8	0.1	0.1
C_{16}	18.5	18.6	18.6	0.1	0.1	19	19.1	19.1	0.1	0.1
C_{17}	31.8	32.0	32.0	0.2	0.2	36.9	37.0	37.1	0.1	0.2
C_{18}	43.2	43.3	43.4	0.1	0.2	39.1	39.2	39.2	0.0	0.0
C_{18}-Me	16.1	16.2	16.3	0.1	0.1	20.1	20.1	20.1	0.0	0.0
$CH_3O(CO)$	-	-	-	-	-	51.9	51.9	51.9	0.0	0.0

Reported values in the table with calibration at 77.00 ppm for $CDCl_3$ solvent peak, for comparison with the data of the natural products. Higher differences of chemical shift (ppm) with natural products are highlighted in light yellow (Δ = 0.01 ppm) or light orange (Δ ≥ 0.02 ppm) for the farthest data of the two pairs of synthesized diastereomers.

[a] Reported data for C_8 and C_6-Me of natural mycaperoxide G methyl ester were considered misreported, probably due to the overlap of C_6-Me and C_{18}-Me. Original data values were 53.4 ppm for C_8 and 21.1 ppm for C_6-Me.

II-Computational methods

Geometry was fully optimized with chloroform as solvent (scrf=chloroform) and without constraint, in the framework of the density functional theory[9,10] as implemented in the Gaussian 16, revision B.01 software package[11] using the hybrid Becke, 3-parameter, Lee-Yang-Parr exchange-correlation functional,[12] the 6-31G(d,p) basis set[13,14] for organo-elements. Vibrational analysis at the same level of theory was conducted upon geometrical optimization convergence, and local minima and first-order saddle points were characterized by their respective number of imaginary frequencies.

(+)-Mycaperoxide D thioethyl ester **16h**

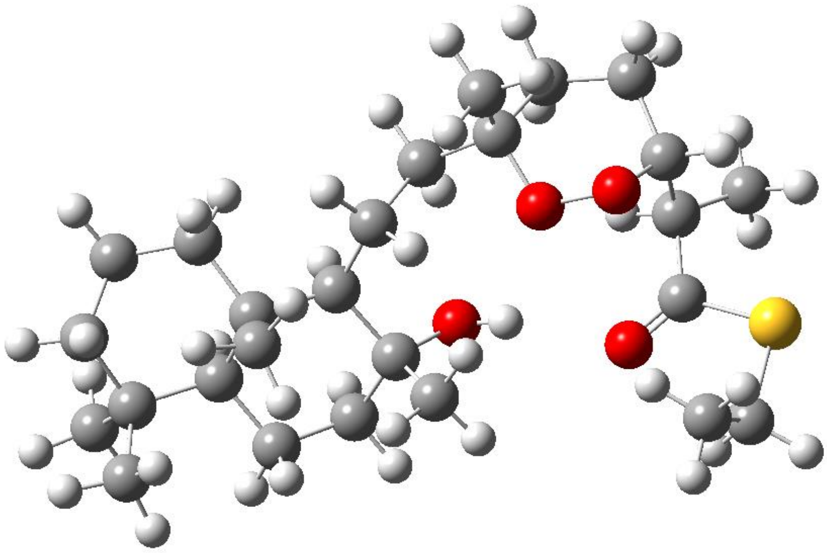

Lowest frequency = 19.242 cm^{-1}

E(RB3LYP) = -1717.551640 Ha

Sum of electronic and thermal Free Energies= -1716.923520 Ha = -4507783.045145 kJ/mol

= -1077386.005054 kcal/mol

Cartesian coordinates:

C	-5.39575600	1.82592000	-0.05377300
C	-6.17725800	0.61370500	0.45247800
C	-5.66569100	-0.71117800	-0.18208000
C	-4.13388300	-0.58723500	-0.53573000
C	-3.28312600	0.33661200	0.40147000
C	-3.91715600	1.77657600	0.38148800
C	-3.44960200	-1.94289000	-0.77779700
C	-2.06853400	-1.75933500	-1.40906300
C	-1.11040600	-0.90462400	-0.56130100
C	-1.82486100	0.44835300	-0.19650100
C	-5.99415600	-1.88157200	0.76819400
C	-6.43946300	-0.94554400	-1.50007800
C	-3.26363400	-0.16042000	1.86771000
C	-0.91825900	1.40843700	0.62183500
C	-0.56192000	-1.72082800	0.61962200
O	-0.00740100	-0.59507000	-1.43831300
H	-4.11003100	-0.07382600	-1.50913800
C	2.41212900	3.09496300	-0.66267100
C	1.47393800	2.42179200	0.36480900
C	4.16517100	1.38815100	0.01514200
C	3.90031500	2.85524500	-0.35602600
C	4.07705000	0.38629000	-1.16837000
C	0.07601000	2.21439800	-0.24945800
O	1.92235800	1.05159800	0.55132700
O	3.27995700	1.02932800	1.08458800
C	5.28415800	0.51027100	-2.11271800
C	3.88350500	-1.03055900	-0.62221100
C	1.43924900	3.15868200	1.70667100
O	2.81241100	-1.60271500	-0.64037900
S	5.36226000	-1.80080400	0.06673500
C	3.96852000	-3.09102800	2.14514400
C	4.60474300	-3.31853200	0.77670500
H	-5.85110800	2.75567500	0.30716900
H	-5.46332500	1.86686900	-1.14847100
H	-7.24725500	0.72639900	0.23648700
H	-6.09773400	0.56676500	1.54568900
H	-3.83195800	2.21670800	1.38223100
H	-3.34059800	2.42953400	-0.28376300
H	-3.36534700	-2.50726400	0.15851000
H	-4.06635300	-2.56006700	-1.44139300
H	-1.59344400	-2.72710400	-1.60850500
H	-2.17387500	-1.26026400	-2.38069400
H	-1.97265400	0.91464300	-1.18136300
H	-5.78029700	-2.85287500	0.31170600
H	-7.06246100	-1.86689600	1.01443200
H	-5.44072100	-1.82339200	1.70880200
H	-6.36085000	-0.08175000	-2.16962300
H	-6.06365200	-1.81895100	-2.04314200
H	-7.50326200	-1.11530500	-1.29864700
H	-4.22536700	0.00886700	2.35691700
H	-3.03824800	-1.22454300	1.95837100
H	-2.51383800	0.38341100	2.45054700
H	-1.53816600	2.10735600	1.18986700
H	-0.36184000	0.83726400	1.36828700
H	-0.01652300	-2.58181600	0.21963200
H	-1.34511400	-2.09825700	1.27845300
H	0.14096400	-1.13960400	1.22254000
H	0.83245800	-0.75433400	-0.97922300
H	2.20863100	4.17104900	-0.70053000
H	2.17236400	2.69743900	-1.65518600
H	5.15004900	1.28780400	0.48803000
H	4.21533400	3.46752600	0.49591100
H	4.51672000	3.16369800	-1.20718900
H	3.15836700	0.57912600	-1.72776100
H	-0.33267900	3.20694200	-0.48021800
H	0.21398700	1.69240800	-1.19787600

H	5.31352000	1.50428700	-2.56797300
H	6.23217100	0.35409300	-1.58860100
H	5.22289700	-0.22228300	-2.92198300
H	1.05286200	4.17402400	1.57045000
H	0.79194600	2.63556200	2.41456600
H	2.43514300	3.22272900	2.14983500
H	3.53091100	-4.02676000	2.51126600
H	4.70580000	-2.74879700	2.87668800
H	3.17438900	-2.34385700	2.08032000
H	3.86745500	-3.66214700	0.04826300
H	5.41946000	-4.04625400	0.81981300

III-References

(1) Piotto, M.; Bourdonneau, M.; Elbayed, K.; Wieruszeski, J.-M.; Lippens, G. New DEFT Sequences for the Acquisition of One-Dimensional Carbon NMR Spectra of Small Unlabelled Molecules. *Magn. Reson. Chem.* **2006**, *44* (10), 943–947. https://doi.org/10.1002/mrc.1884.

(2) Hua, S.-K.; Wang, J.; Chen, X.-B.; Xu, Z.-Y.; Zeng, B.-B. Scalable Synthesis of Methyl Ent-Isocopalate and Its Derivatives. *Tetrahedron* **2011**, *67* (6), 1142–1144. https://doi.org/10.1016/j.tet.2010.12.008.

(3) Barrero, A. F.; Alvarez-Manzaneda, E. J.; Altarejos, J.; Salido, S.; Ramos, J. M. Synthesis of Ambrox® from (−)-Sclareol and (+)-Cis-Abienol. *Tetrahedron* **1993**, *49* (45), 10405–10412. https://doi.org/10.1016/S0040-4020(01)80567-6.

(4) Wang, J.; Wang, P.; Li, J.; Wu, P.; Ren, J.; Zeng, B. An Efficient Synthesis of (+)-Subersic Acid. *Chinese Journal of Chemistry* **2015**, *33* (6), 679–682. https://doi.org/10.1002/cjoc.201500219.

(5) DeRoy, P. L.; Charette, A. B. Total Synthesis of (+)-Cystothiazole A. *Org. Lett.* **2003**, *5* (22), 4163–4165. https://doi.org/10.1021/ol035600s.

(6) Capon, R. J.; Rochfort, S. J.; Ovenden, S. P. B. Cyclic Peroxides and Related Norterpenes from a Southern Australian Marine Sponge, Mycale Sp. *J. Nat. Prod.* **1997**, *60* (12), 1261–1264. https://doi.org/10.1021/np970313g.

(7) Tanaka, J.; Higa, T.; Suwanborirux, K.; Kokpol, U.; Bernardinelli, G.; Jefford, C. W. Bioactive Norsesterterpene 1,2-Dioxanes from a Thai Sponge, Mycale Sp. *J. Org. Chem.* **1993**, *58* (11), 2999–3002. https://doi.org/10.1021/jo00063a016.

(8) Capon, R. J.; Rochfort, S. J.; Ovenden, S. P. B.; Metzger, R. P. Mycaperoxides F and G and a Related Norterpene Ketone from Southern Australian Marine Sponges, Mycale Species. *J. Nat. Prod.* **1998**, *61* (4), 525–528. https://doi.org/10.1021/np970484l.

(9) Hohenberg, P.; Kohn, W. Inhomogeneous Electron Gas. *Phys. Rev.* **1964**, *136* (3B), B864–B871. https://doi.org/10.1103/PhysRev.136.B864.

(10) Kohn, W.; Sham, L. J. Self-Consistent Equations Including Exchange and Correlation Effects. *Phys. Rev.* **1965**, *140* (4A), A1133–A1138. https://doi.org/10.1103/PhysRev.140.A1133.

(11) Frisch, M. J.; Trucks, G. W.; Schlegel, H. B.; Scuseria, G. E.; Robb, M. A.; Cheeseman, J. R.; Scalmani, G.; Barone, V.; Petersson, G. A.; Nakatsuji, H.; Li, X.; Caricato, M.; Marenich, A. V.; Bloino, J.; Janesko, B. G.; Gomperts, R.; Mennucci, B.; Hratchian, H. P.; Ortiz, J. V.; Izmaylov, A. F.;

Sonnenberg, J. L.; Williams; Ding, F.; Lipparini, F.; Egidi, F.; Goings, J.; Peng, B.; Petrone, A.; Henderson, T.; Ranasinghe, D.; Zakrzewski, V. G.; Gao, J.; Rega, N.; Zheng, G.; Liang, W.; Hada, M.; Ehara, M.; Toyota, K.; Fukuda, R.; Hasegawa, J.; Ishida, M.; Nakajima, T.; Honda, Y.; Kitao, O.; Nakai, H.; Vreven, T.; Throssell, K.; Montgomery Jr., J. A.; Peralta, J. E.; Ogliaro, F.; Bearpark, M. J.; Heyd, J. J.; Brothers, E. N.; Kudin, K. N.; Staroverov, V. N.; Keith, T. A.; Kobayashi, R.; Normand, J.; Raghavachari, K.; Rendell, A. P.; Burant, J. C.; Iyengar, S. S.; Tomasi, J.; Cossi, M.; Millam, J. M.; Klene, M.; Adamo, C.; Cammi, R.; Ochterski, J. W.; Martin, R. L.; Morokuma, K.; Farkas, O.; Foresman, J. B.; Fox, D. J. *Gaussian 16 Rev. C.01*; Wallingford, CT, 2016.

(12) Hehre, W. J.; Radom, L.; Schleyer, P. v. R.; Pople, J. A. */Ab Initio Molecular Orbital Theory/*, Wiley.; New York, 1986.

(13) Becke, A. D. Density-functional Thermochemistry. III. The Role of Exact Exchange. *J. Chem. Phys.* **1993**, *98* (7), 5648–5652. https://doi.org/10.1063/1.464913.

(14) Lee, C.; Yang, W.; Parr, R. G. Development of the Colle-Salvetti Correlation-Energy Formula into a Functional of the Electron Density. *Phys. Rev. B* **1988**, *37* (2), 785–789. https://doi.org/10.1103/PhysRevB.37.785.

IV-Copies of ^{1}H and ^{13}C NMR spectra

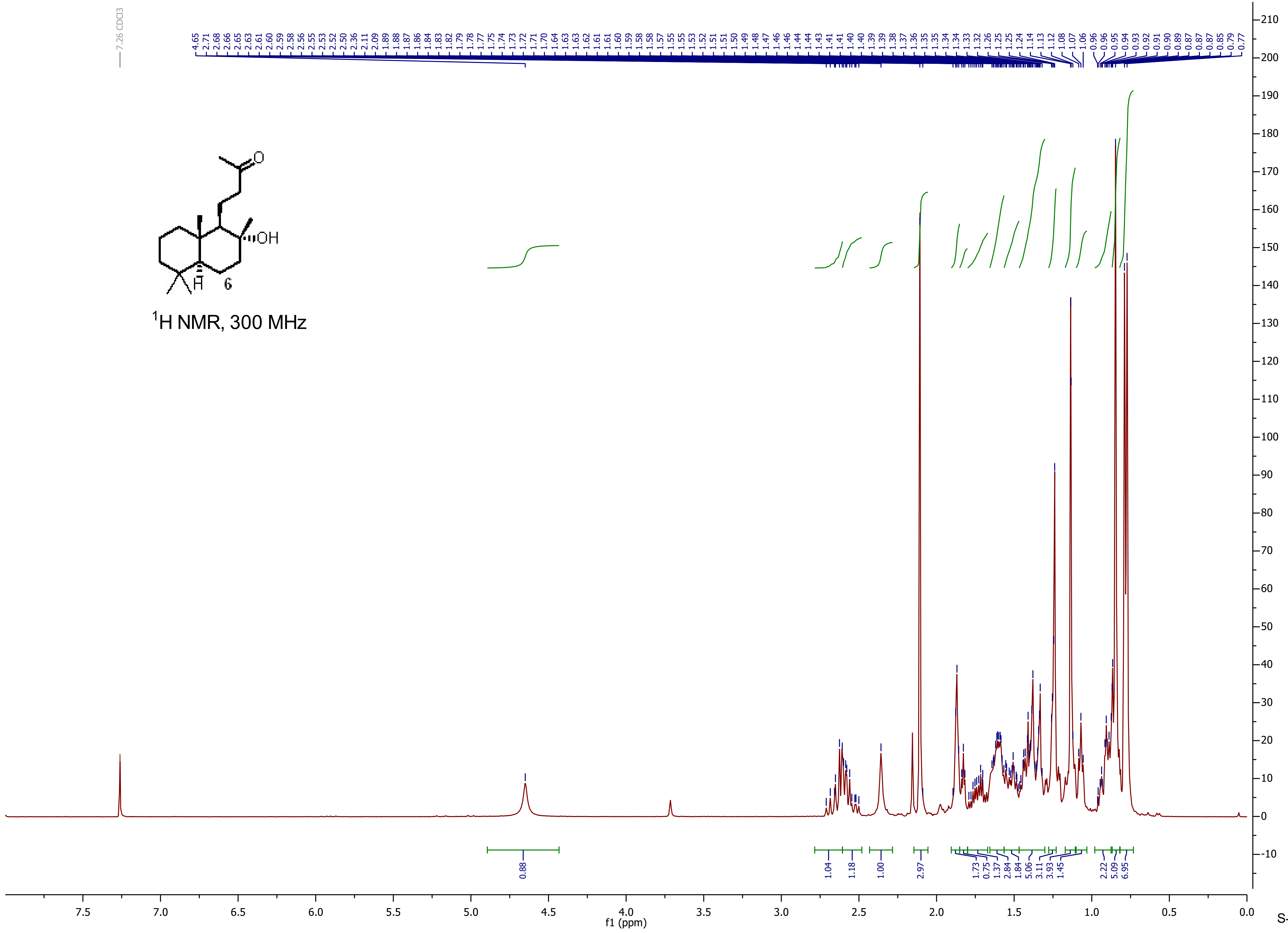

OH
O
H
6
1H NMR, 300 MHz
7.26 CDCl3
4.65 2.71 2.68 2.66 2.65 2.63 2.61 2.60 2.59 2.58 2.56 2.55 2.53 2.52 2.50 2.36 2.11 2.09 1.89 1.88 1.87 1.86 1.84 1.83 1.82 1.79 1.78 1.77 1.75 1.74 1.73 1.72 1.71 1.70 1.64 1.63 1.63 1.62 1.61 1.61 1.60 1.59 1.58 1.58 1.57 1.55 1.55 1.53 1.52 1.51 1.51 1.50 1.49 1.48 1.47 1.46 1.44 1.44 1.43 1.41 1.41 1.40 1.40 1.39 1.39 1.38 1.37 1.36 1.35 1.35 1.34 1.34 1.33 1.32 1.26 1.25 1.25 1.24 1.14 1.13 1.12 1.08 1.07 1.06 0.96 0.96 0.95 0.94 0.93 0.92 0.91 0.90 0.89 0.87 0.87 0.85 0.79 0.77
0.88 1.04 1.18 1.00 2.97 1.73 0.75 1.37 2.84 1.84 5.06 3.11 3.93 1.45 2.22 5.09 6.95
f1 (ppm)

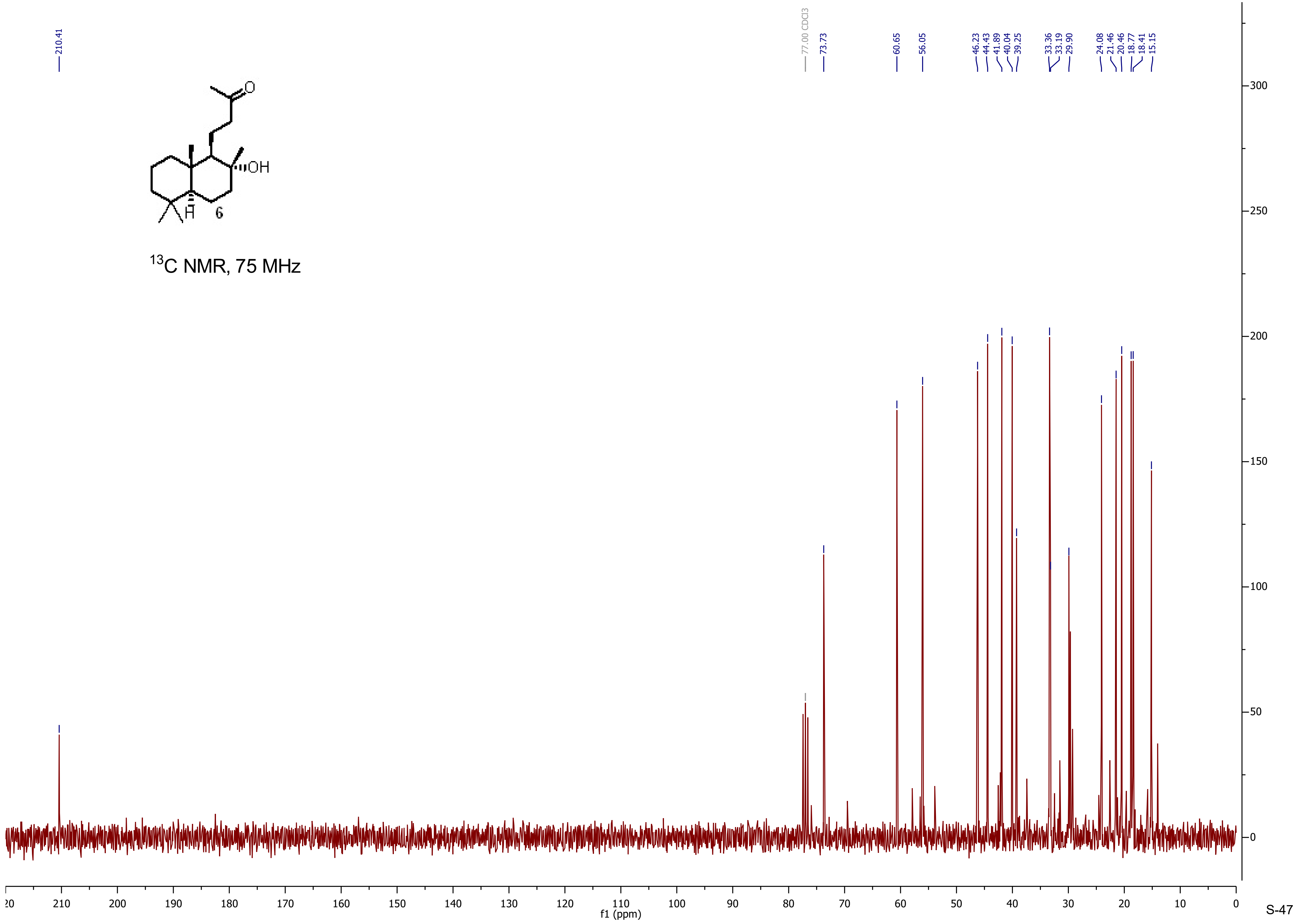
210.41
77.00 CDCl3
73.73
60.65
56.05
46.23
44.43
41.89
40.04
39.25
33.36
33.19
29.90
24.08
21.46
20.46
18.77
18.41
15.15
OH
H
6
^{13}C NMR, 75 MHz
f1 (ppm)

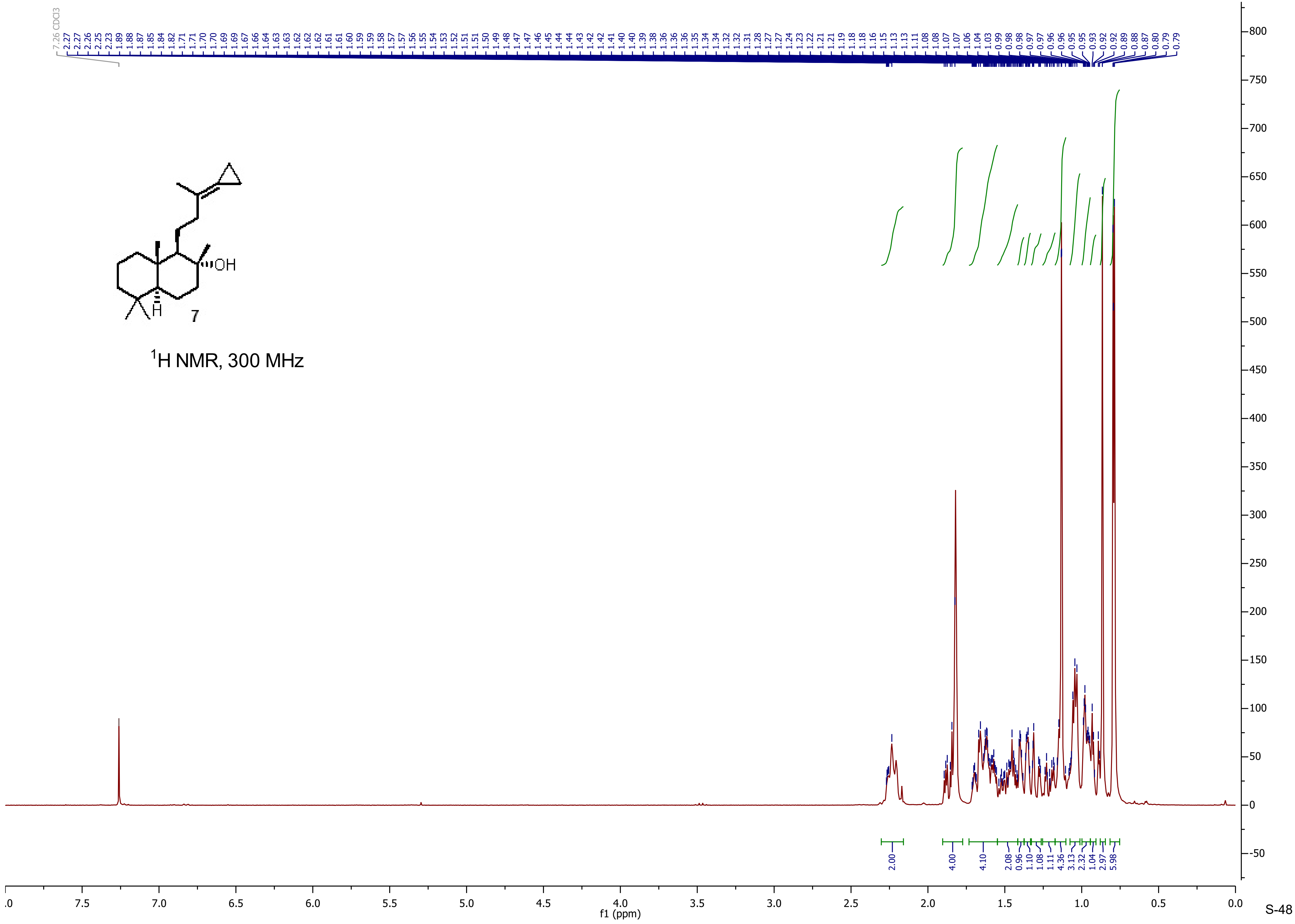
OH
H
7
1H NMR, 300 MHz
f1 (ppm)

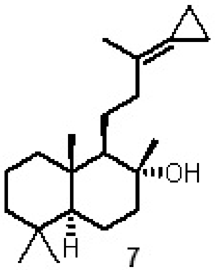

7

^{13}C NMR, 75 MHz

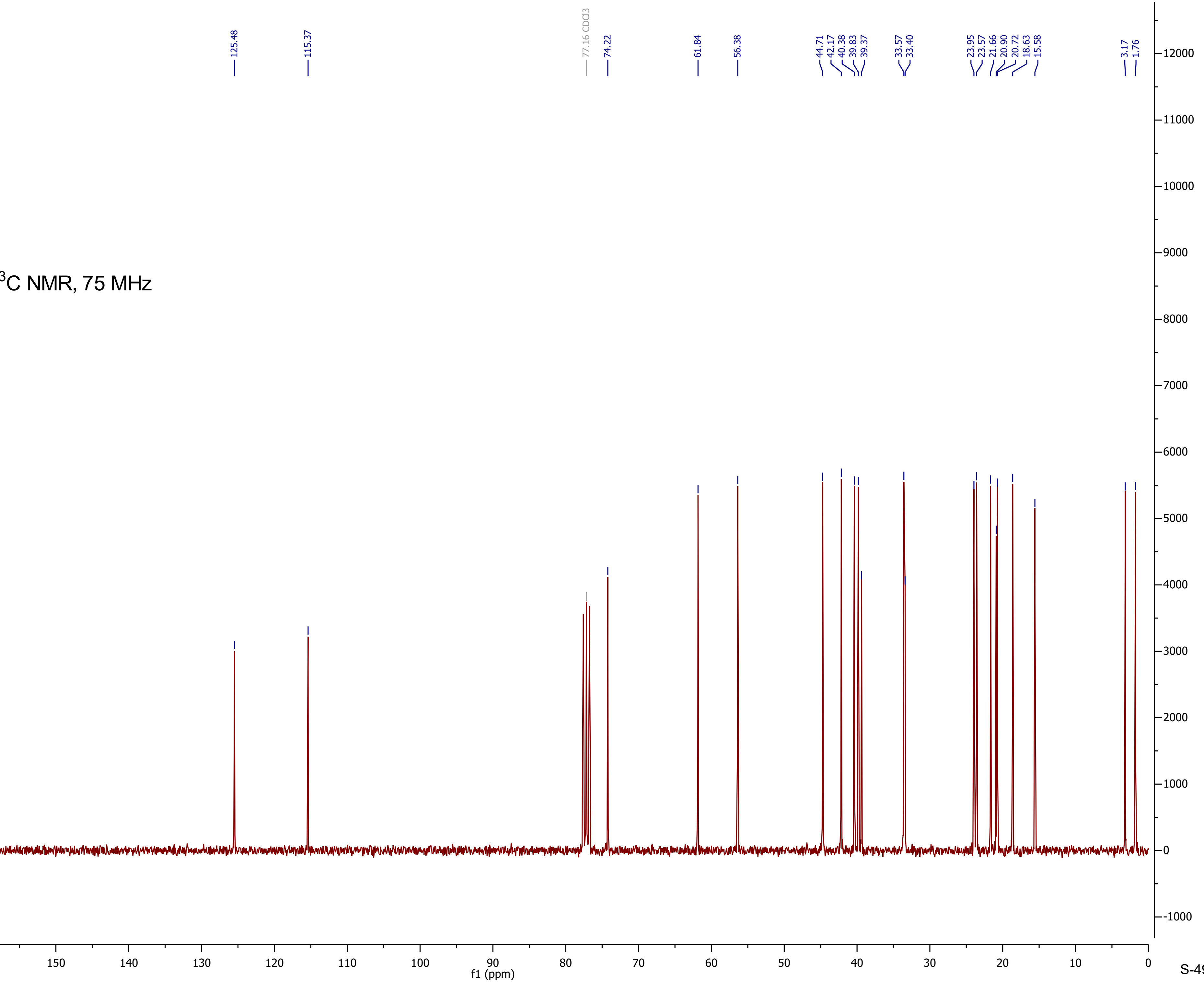

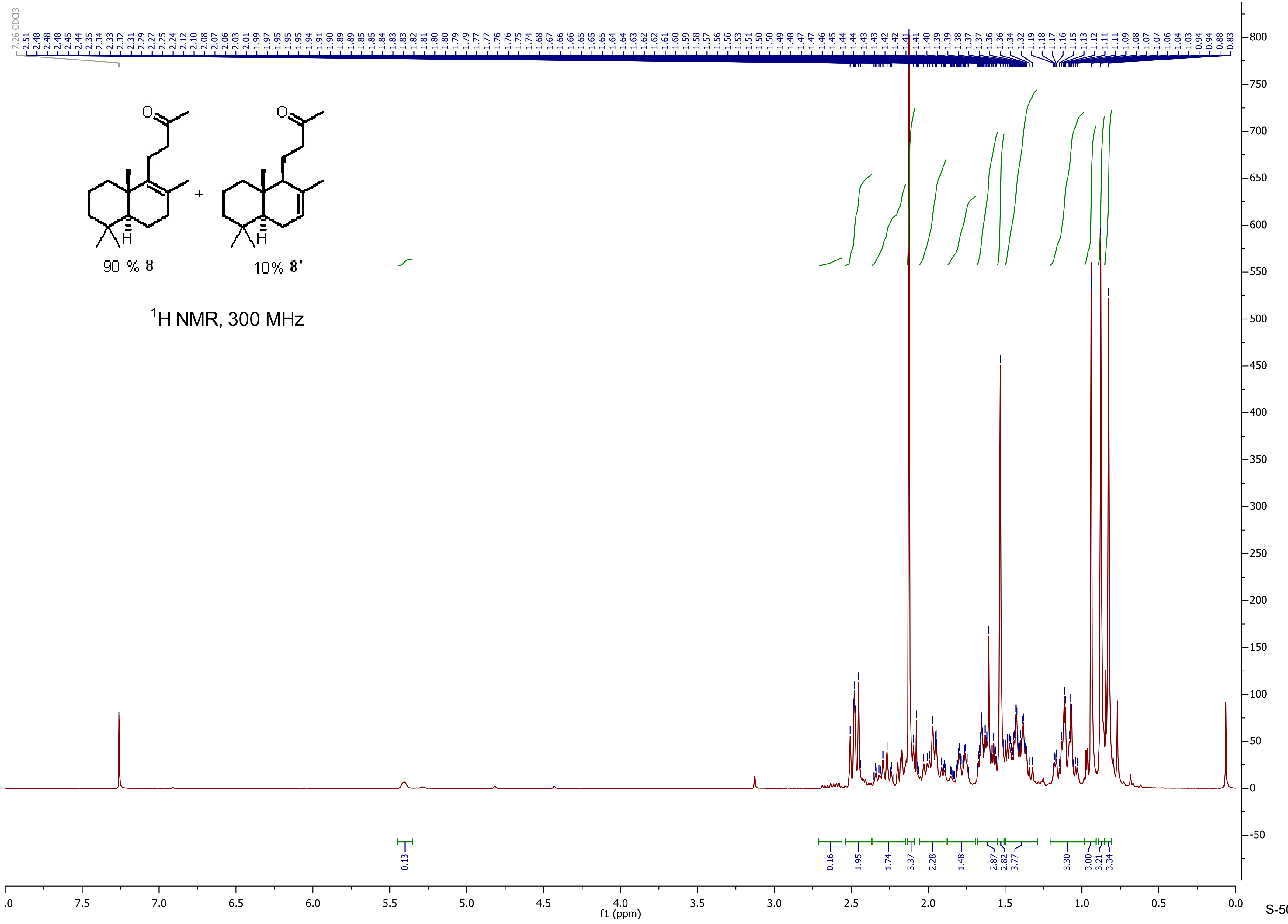
90 % 8
10% 8'
1H NMR, 300 MHz
f1 (ppm)

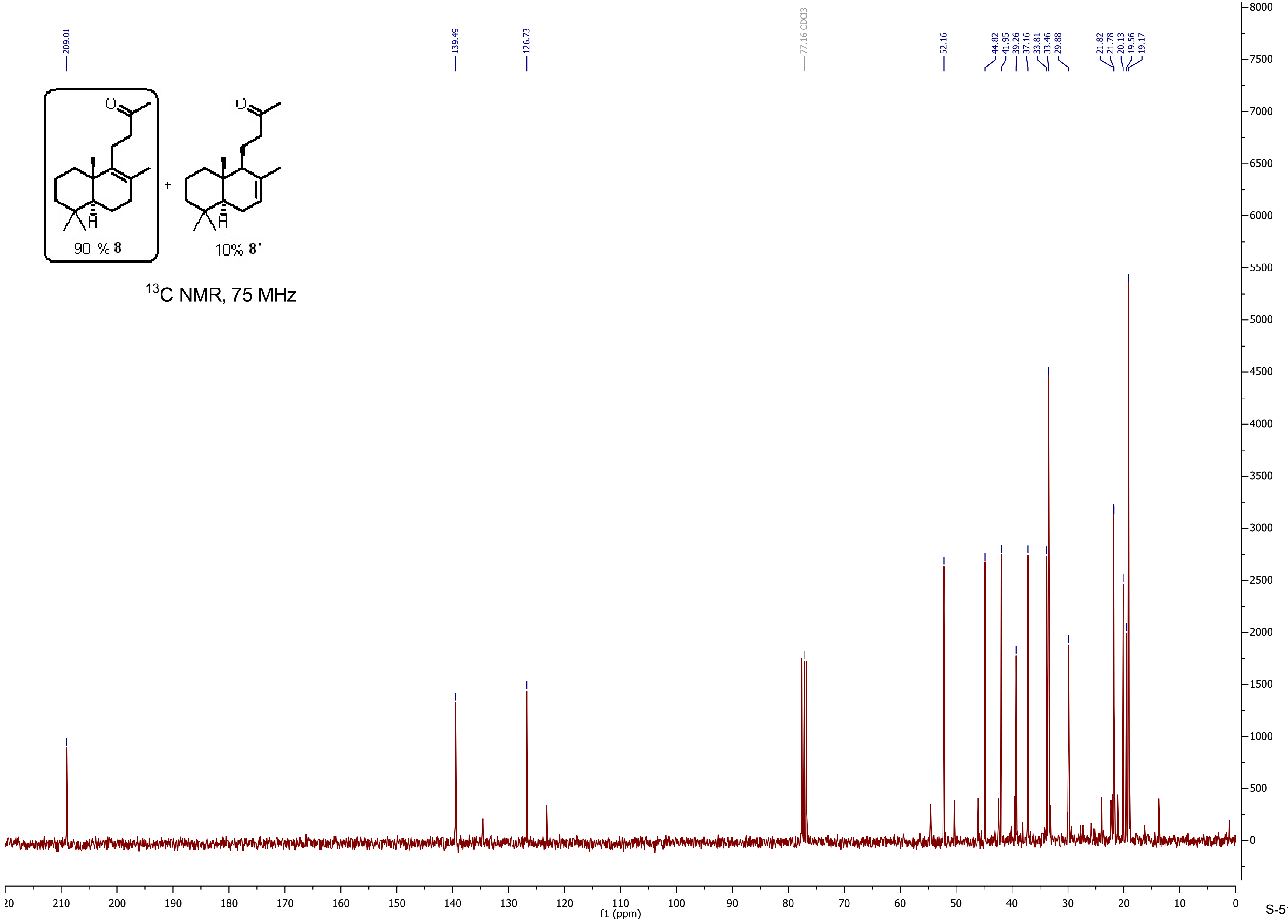
209.01
139.49
126.73
77.16 CDCl3
52.16
44.82
41.95
39.26
37.16
33.81
33.46
29.88
21.82
21.78
20.13
19.56
19.17
O
O
H
H
+
90 % 8
10% 8'
13C NMR, 75 MHz
f1 (ppm)

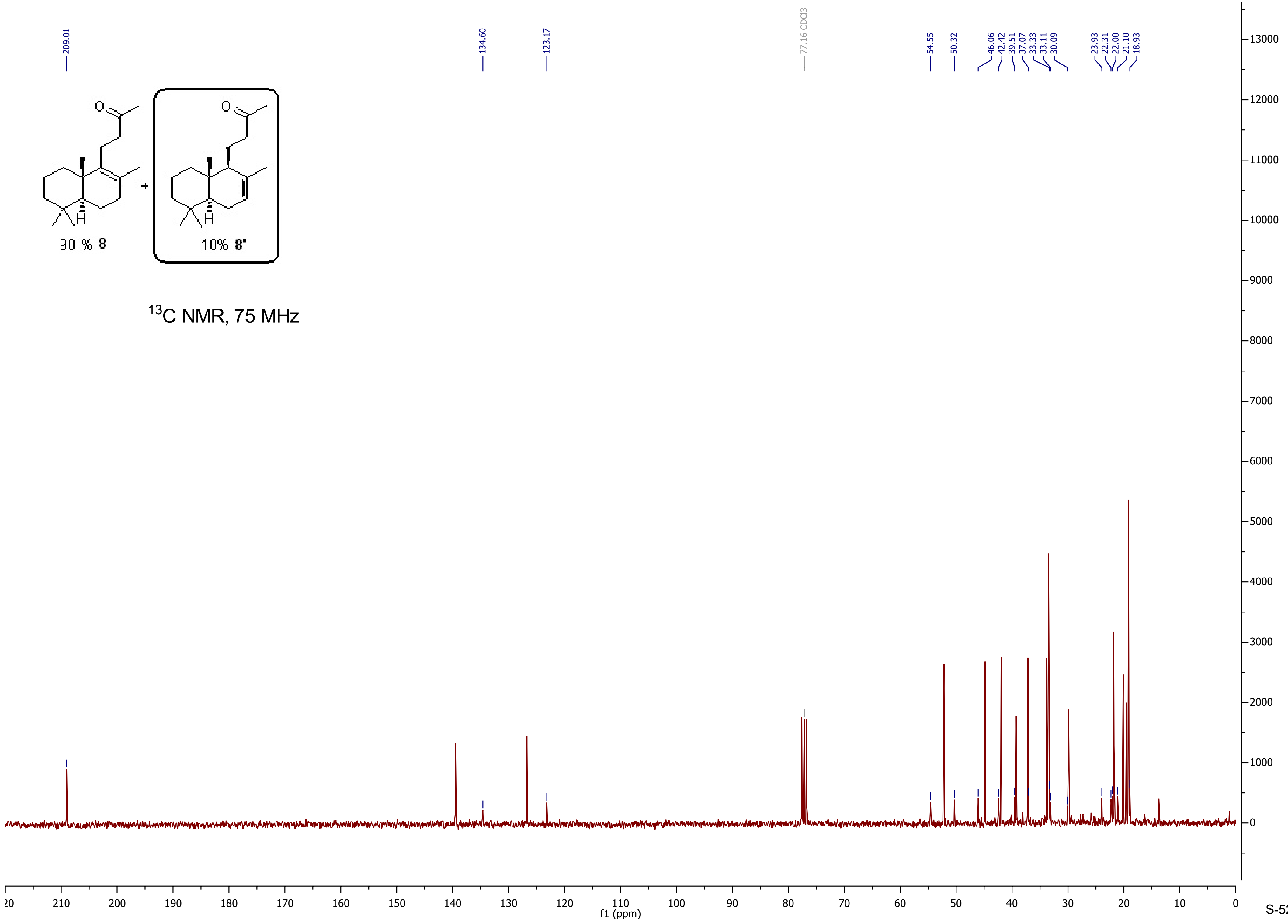
209.01
134.60
123.17
77.16 CDCl3
54.55
50.32
46.06
42.42
39.51
37.07
33.33
33.11
30.09
23.93
22.31
22.00
21.10
18.93
O
H
90 % 8
10% 8'
^{13}C NMR, 75 MHz
f1 (ppm)

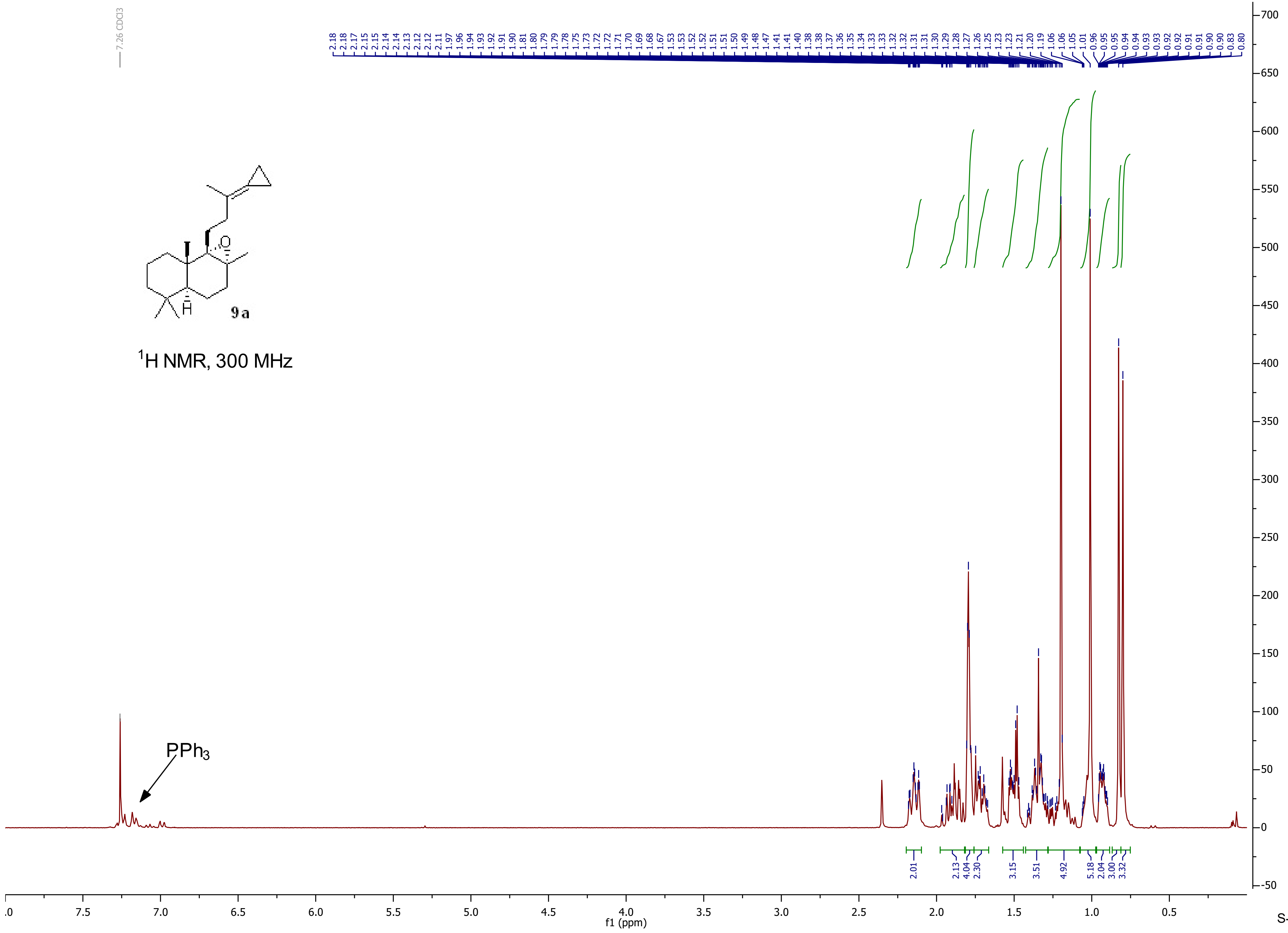

9a
^{1}H NMR, 300 MHz
PPh$_3$
f1 (ppm)

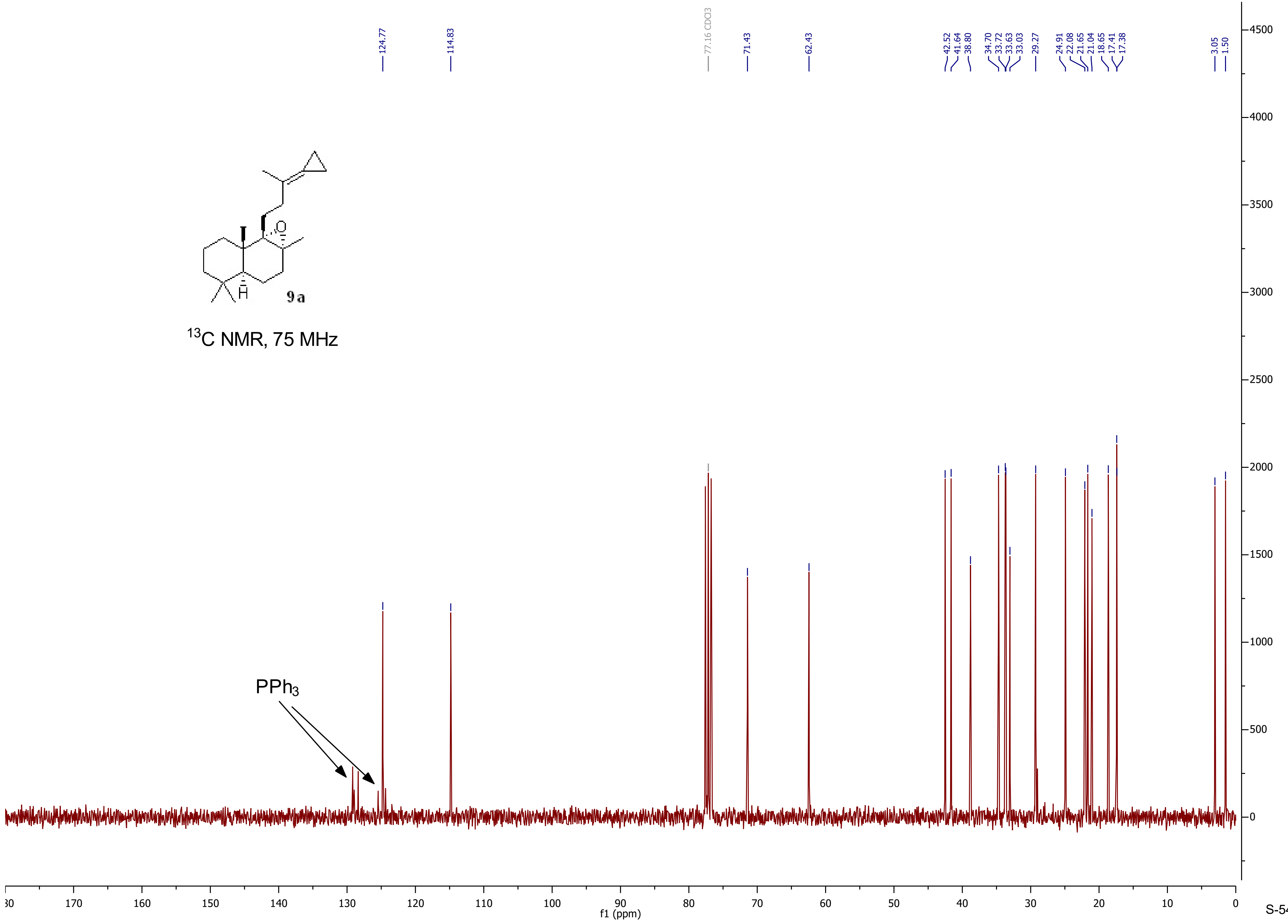
124.77
114.83
77.16 CDCl3
71.43
62.43
42.52
41.64
38.80
34.70
33.72
33.63
33.03
29.27
24.91
22.08
21.65
21.04
18.65
17.41
17.38
3.05
1.50
O
H
9a
^{13}C NMR, 75 MHz
PPh_3
f1 (ppm)

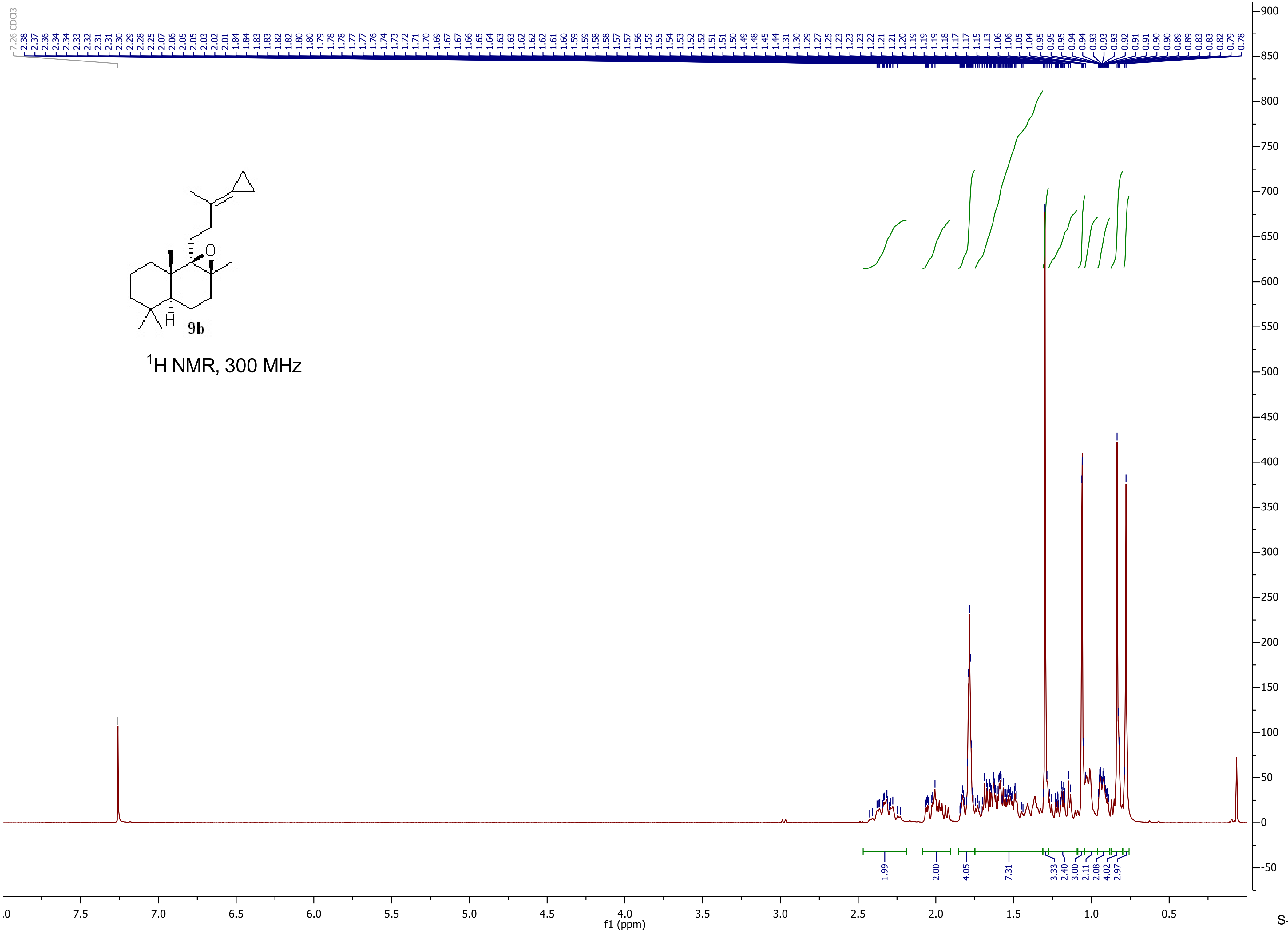

9b

^1H NMR, 300 MHz

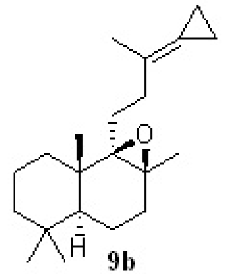

9b

^{13}C NMR, 75 MHz

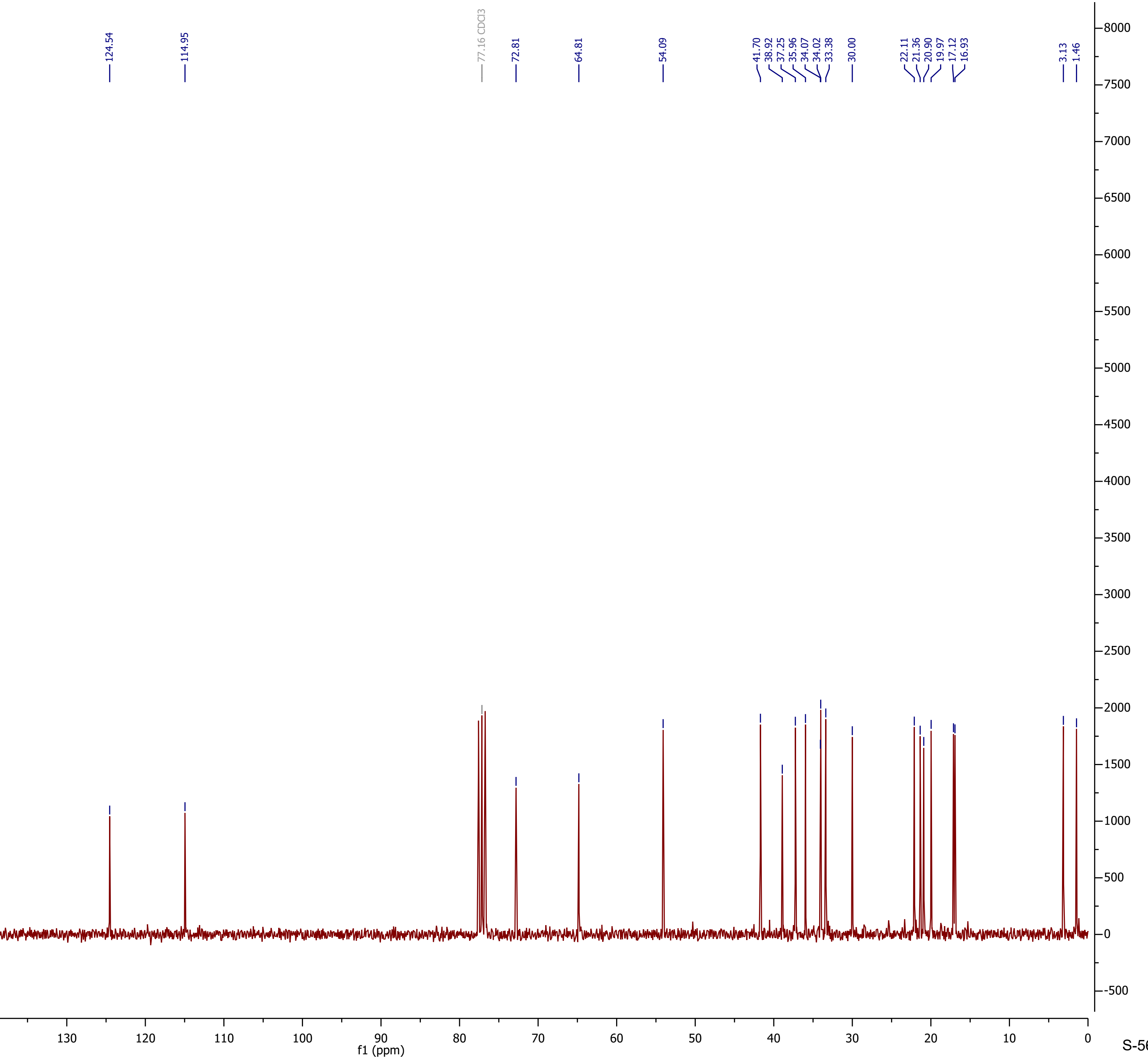

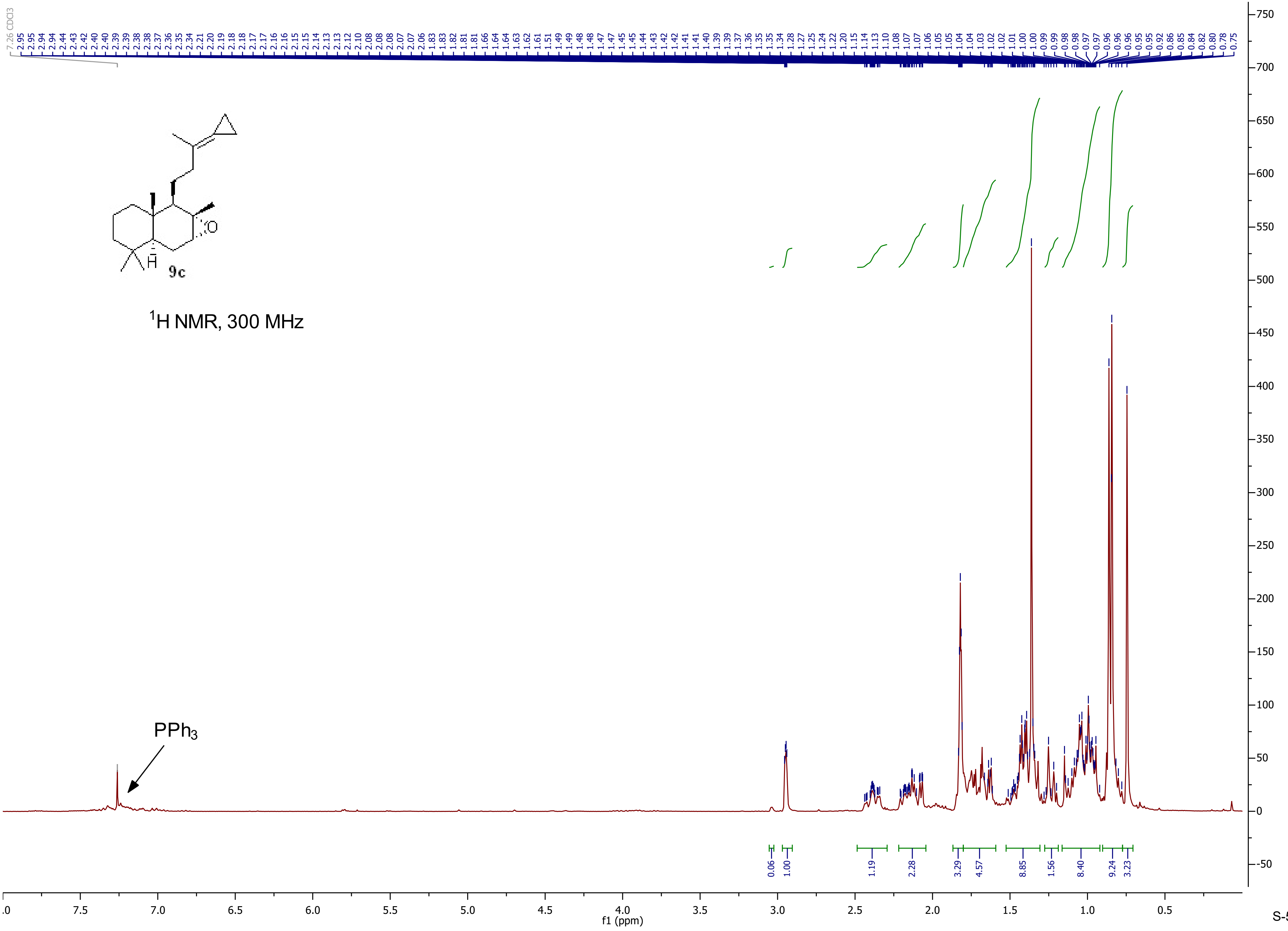
9c
^{1}H NMR, 300 MHz
PPh$_3$
7.26 CDCl3
2.95 2.95 2.94 2.94 2.44 2.43 2.42 2.40 2.40 2.39 2.39 2.38 2.38 2.37 2.36 2.35 2.34 2.21 2.20 2.19 2.18 2.18 2.17 2.17 2.16 2.16 2.15 2.15 2.14 2.13 2.13 2.12 2.10 2.08 2.08 2.07 2.07 2.06 1.83 1.83 1.82 1.81 1.81 1.66 1.64 1.64 1.63 1.62 1.61 1.51 1.49 1.49 1.48 1.48 1.47 1.47 1.45 1.45 1.44 1.43 1.42 1.42 1.41 1.41 1.40 1.39 1.39 1.37 1.36 1.35 1.35 1.34 1.28 1.27 1.25 1.24 1.22 1.20 1.15 1.14 1.13 1.10 1.08 1.07 1.07 1.06 1.05 1.05 1.04 1.04 1.03 1.02 1.02 1.01 1.00 1.00 0.99 0.99 0.98 0.98 0.97 0.97 0.96 0.96 0.96 0.95 0.95 0.92 0.86 0.85 0.84 0.82 0.80 0.78 0.75
0.06 1.00 1.19 2.28 3.29 4.57 8.85 1.56 8.40 9.24 3.23
f1 (ppm)
.0 7.5 7.0 6.5 6.0 5.5 5.0 4.5 4.0 3.5 3.0 2.5 2.0 1.5 1.0 0.5
750 700 650 600 550 500 450 400 350 300 250 200 150 100 50 0 -50

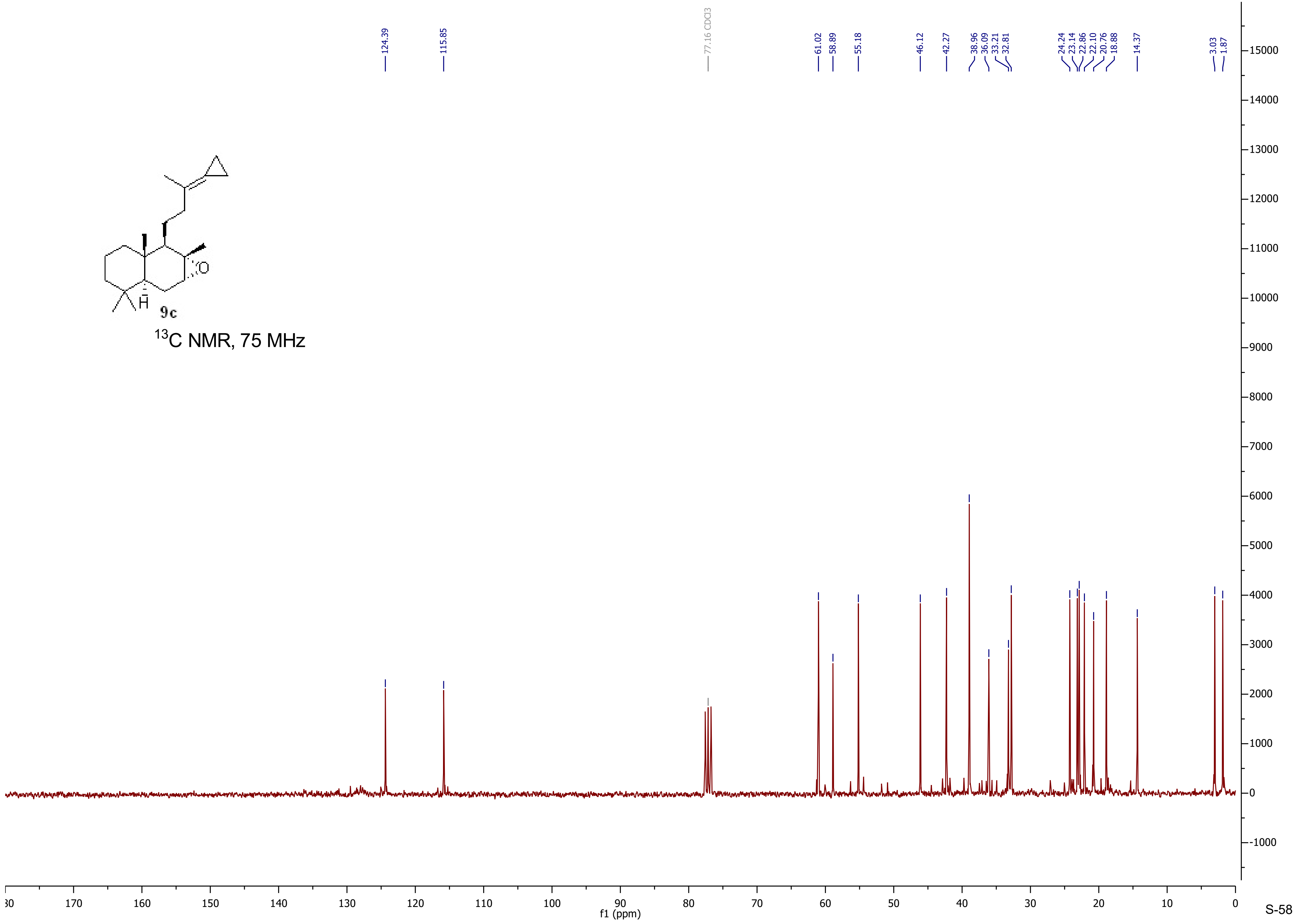
124.39
115.85
77.16 CDCl3
61.02
58.89
55.18
46.12
42.27
38.96
36.09
33.21
32.81
24.24
23.14
22.86
22.10
20.76
18.88
14.37
3.03
1.87
9c
^{13}C NMR, 75 MHz
f1 (ppm)

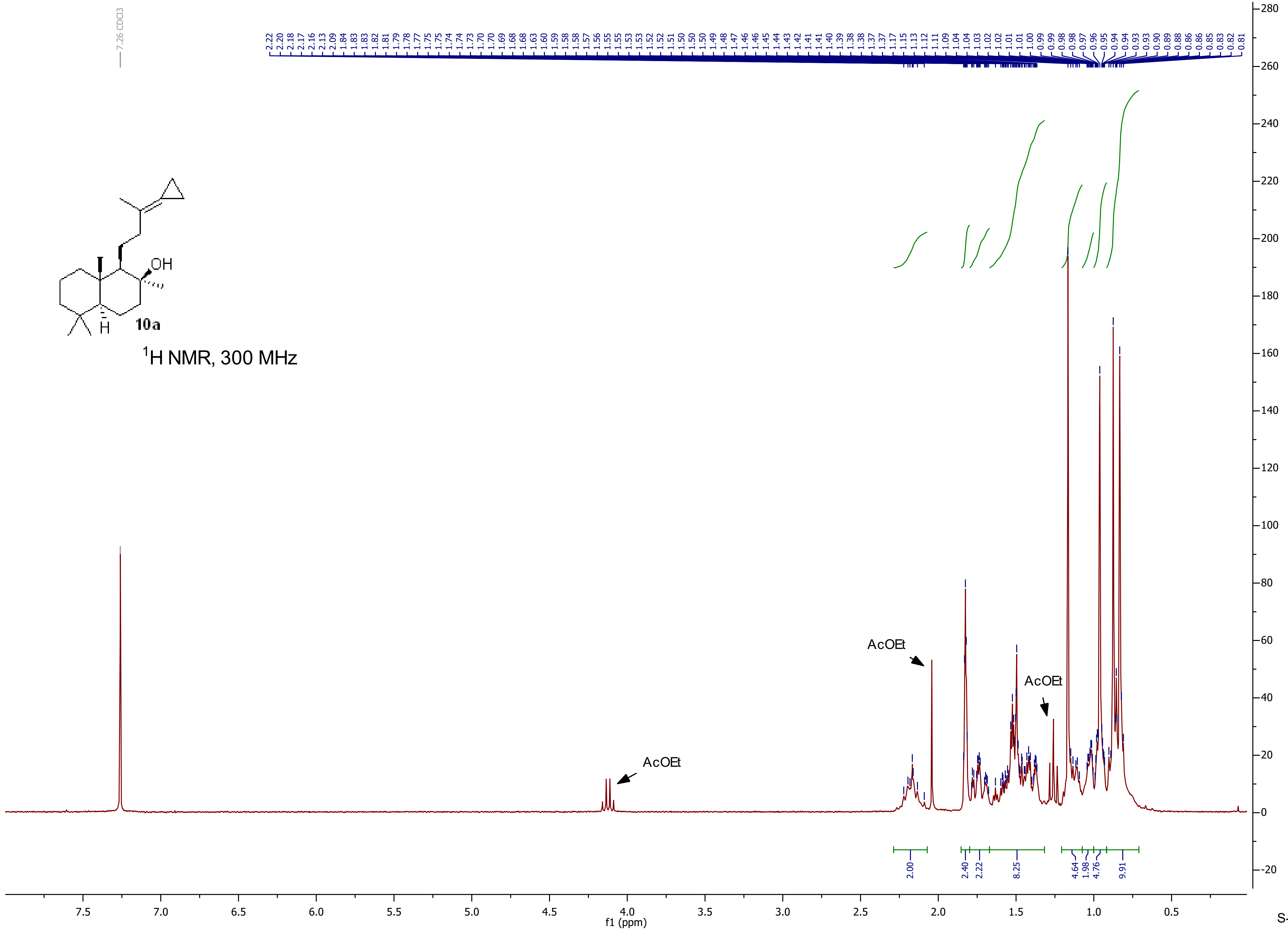

7.26 CDCl3
2.22 2.20 2.18 2.17 2.16 2.13 2.09 1.84 1.83 1.83 1.82 1.81 1.79 1.78 1.77 1.75 1.75 1.74 1.74 1.73 1.70 1.70 1.69 1.68 1.63 1.60 1.59 1.58 1.58 1.57 1.56 1.55 1.55 1.53 1.53 1.52 1.52 1.51 1.50 1.50 1.50 1.49 1.48 1.47 1.46 1.46 1.45 1.44 1.43 1.42 1.41 1.41 1.40 1.39 1.38 1.38 1.37 1.37 1.17 1.15 1.13 1.12 1.11 1.09 1.04 1.04 1.03 1.02 1.02 1.01 1.01 1.00 0.99 0.99 0.98 0.98 0.97 0.96 0.95 0.94 0.94 0.93 0.93 0.90 0.89 0.88 0.86 0.86 0.85 0.83 0.82 0.81
OH
H
10a
^{1}H NMR, 300 MHz
AcOEt
AcOEt
AcOEt
2.00
2.40
2.22
8.25
4.64
1.98
4.76
9.91
7.5 7.0 6.5 6.0 5.5 5.0 4.5 4.0 3.5 3.0 2.5 2.0 1.5 1.0 0.5
f1 (ppm)
280 260 240 220 200 180 160 140 120 100 80 60 40 20 0 -20

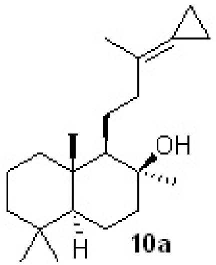

10a

^{13}C NMR, 75 MHz

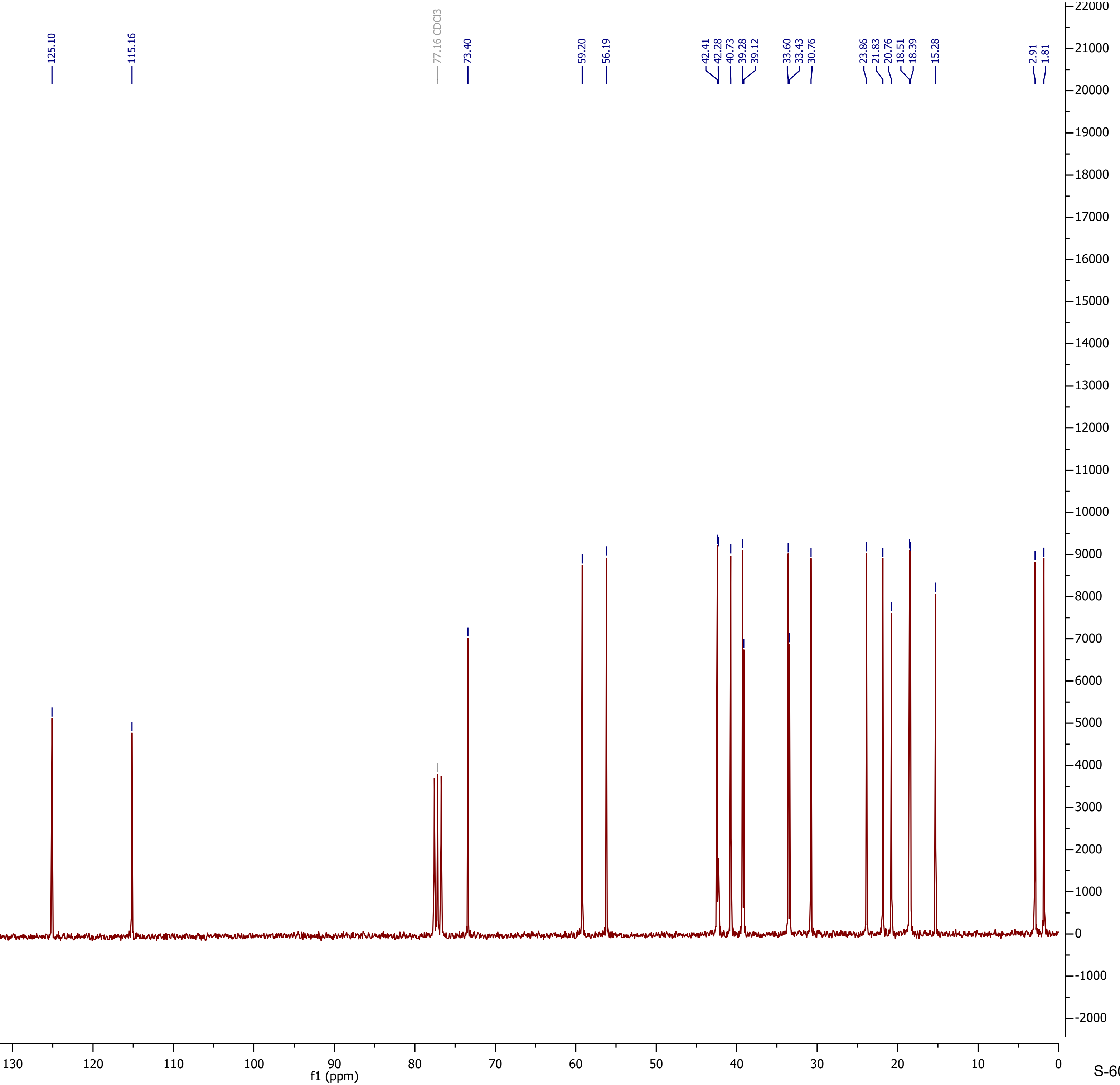

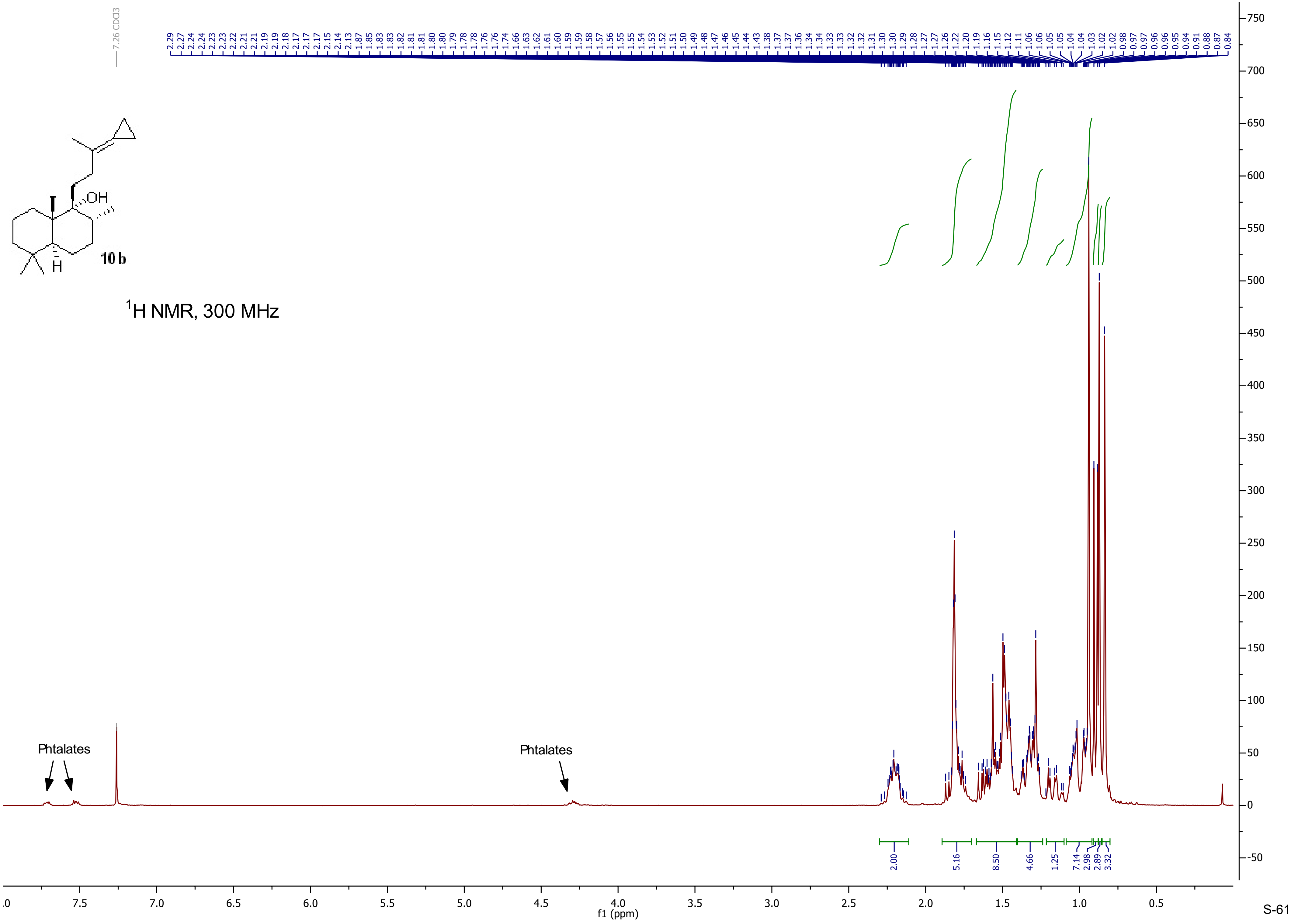
OH
H
10b
1H NMR, 300 MHz
7.26 CDCl3
Phtalates
Phtalates
2.00
5.16
8.50
4.66
1.25
7.14
2.98
2.89
3.32
f1 (ppm)

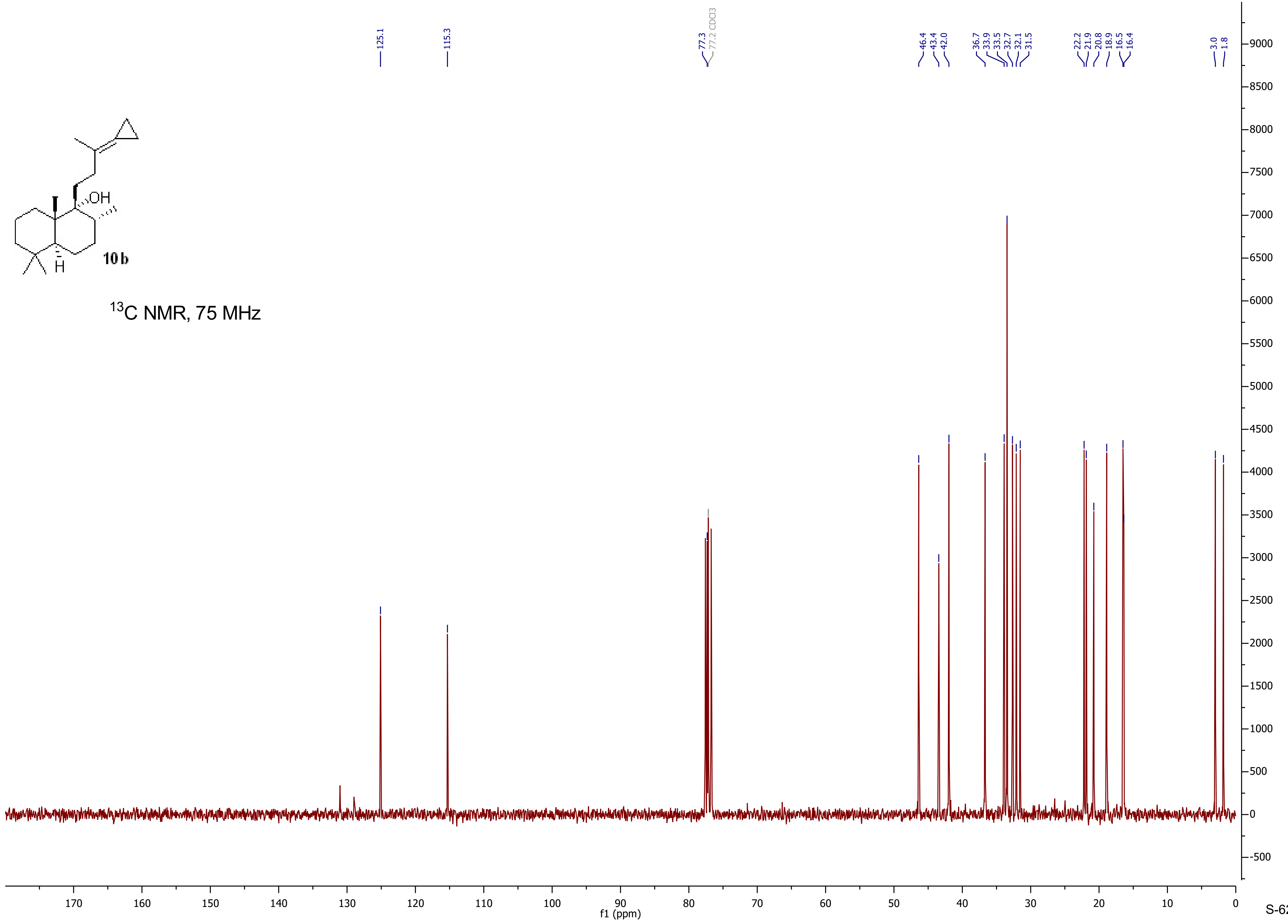
125.1
115.3
77.3
77.2 CDCl3
46.4
43.4
42.0
36.7
33.9
33.5
32.7
32.1
31.5
22.2
21.9
20.8
18.9
16.5
16.4
3.0
1.8
OH
H
10b
13C NMR, 75 MHz
f1 (ppm)

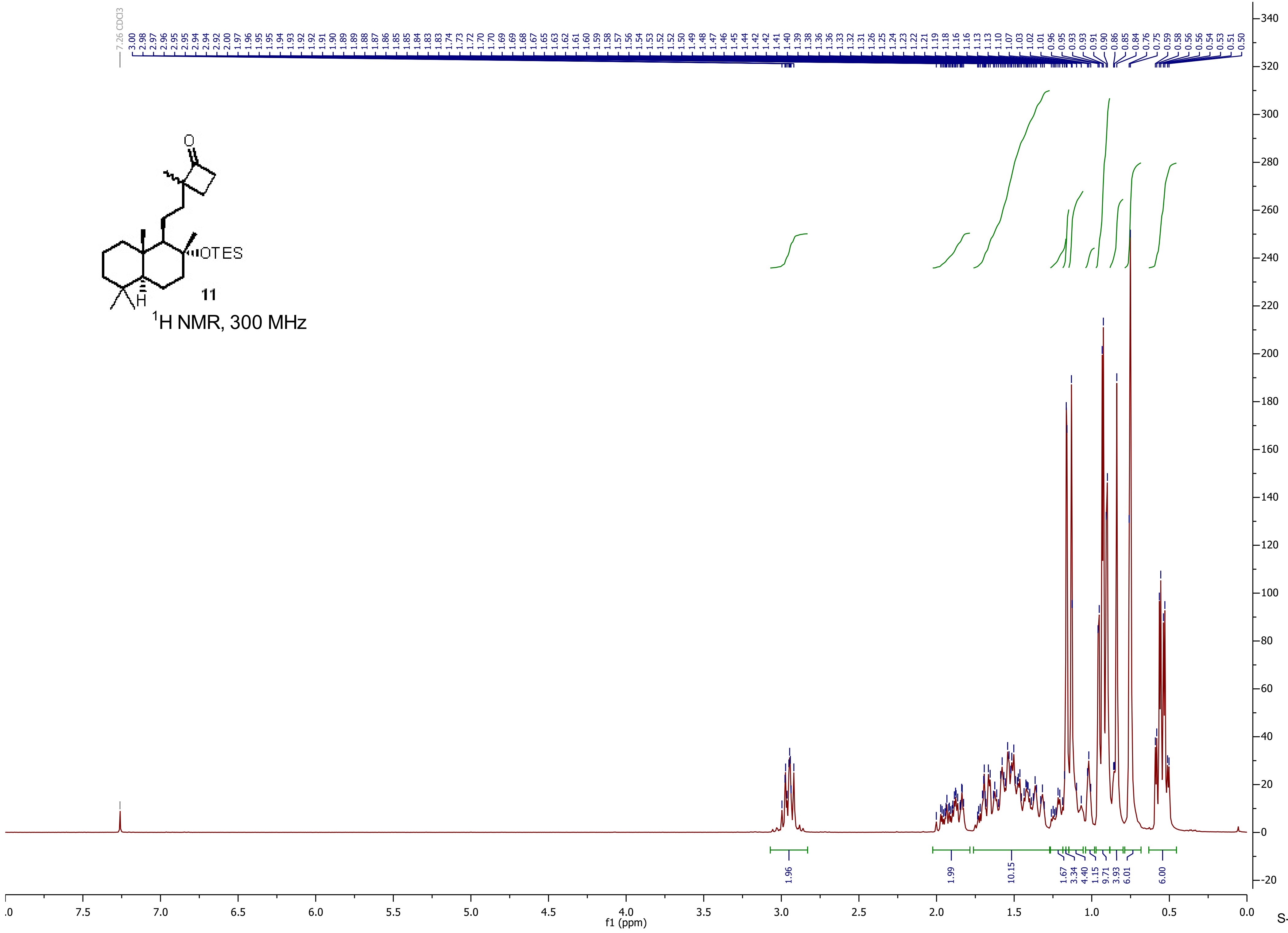
OTES
11
^{1}H NMR, 300 MHz
7.26 CDCl3
1.96
1.99
10.15
1.67
3.34
4.40
1.15
9.71
3.93
6.01
6.00
f1 (ppm)

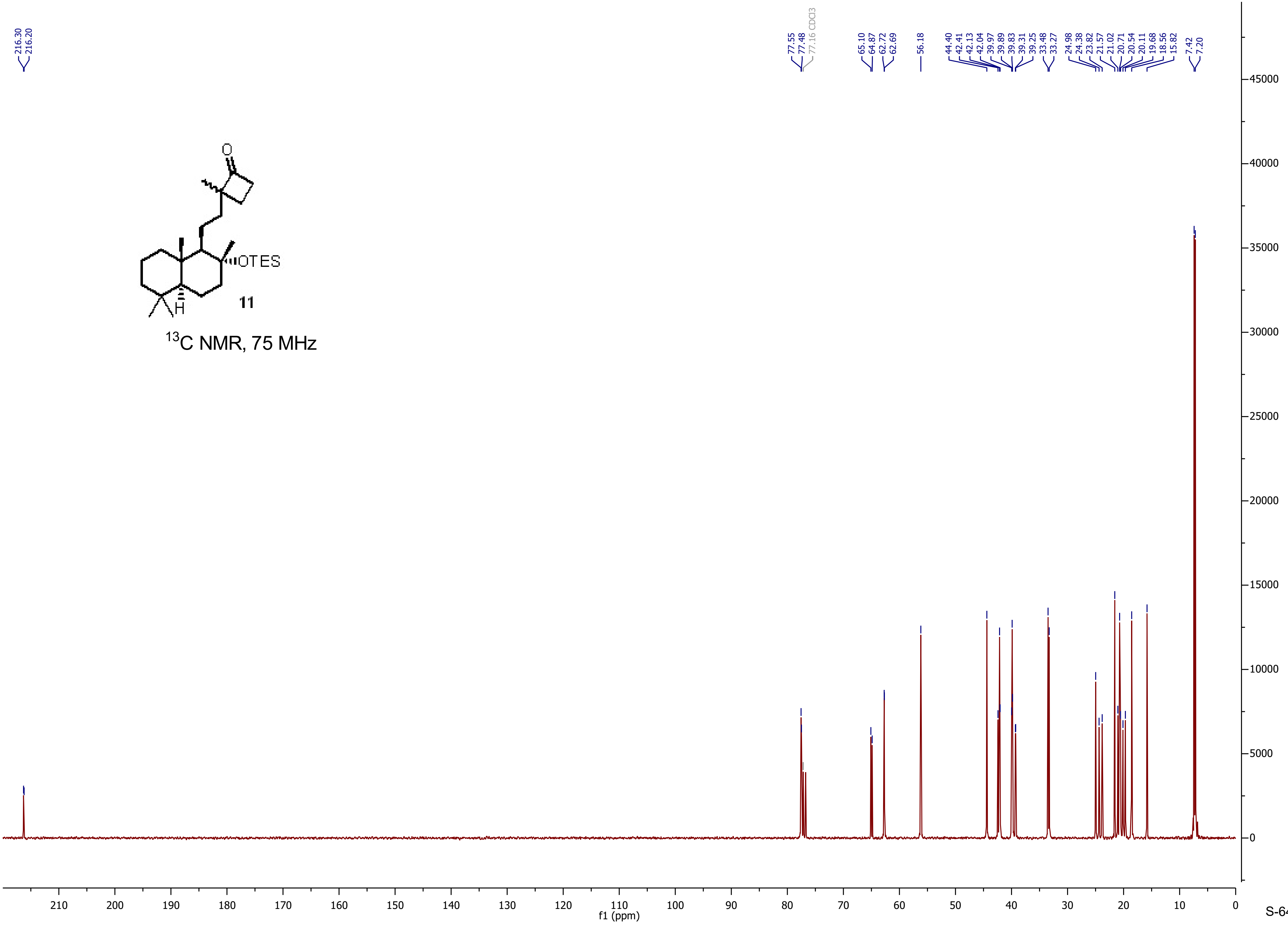
216.30
216.20
77.55
77.48
77.16 CDCl3
65.10
64.87
62.72
62.69
56.18
44.40
42.41
42.13
42.04
39.97
39.89
39.83
39.31
39.25
33.48
33.27
24.98
24.38
23.82
21.57
21.02
20.71
20.54
20.11
19.68
18.56
15.82
7.42
7.20
O
OTES
H
11
13C NMR, 75 MHz
f1 (ppm)

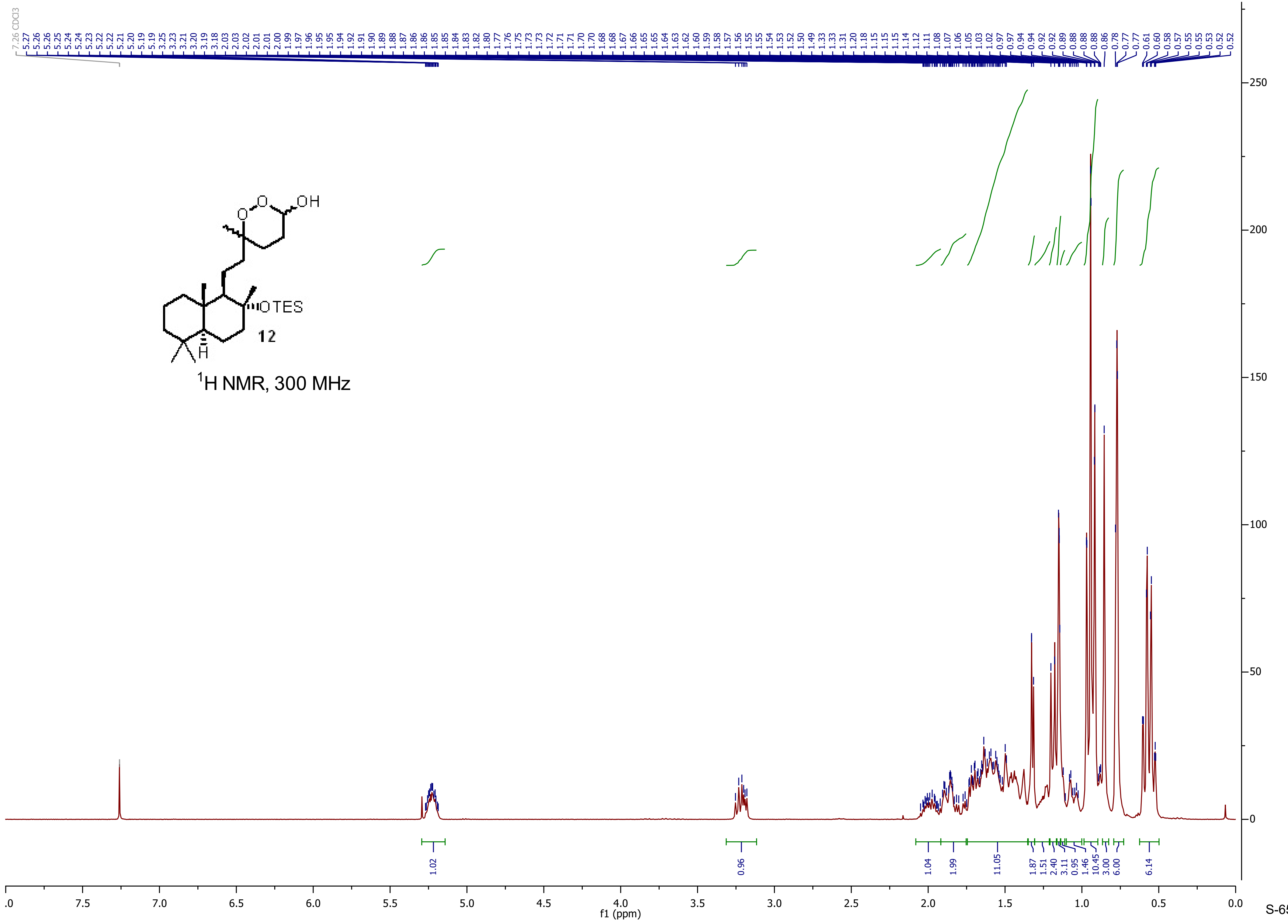
OH
O
O
OTES
12
H
1H NMR, 300 MHz
f1 (ppm)

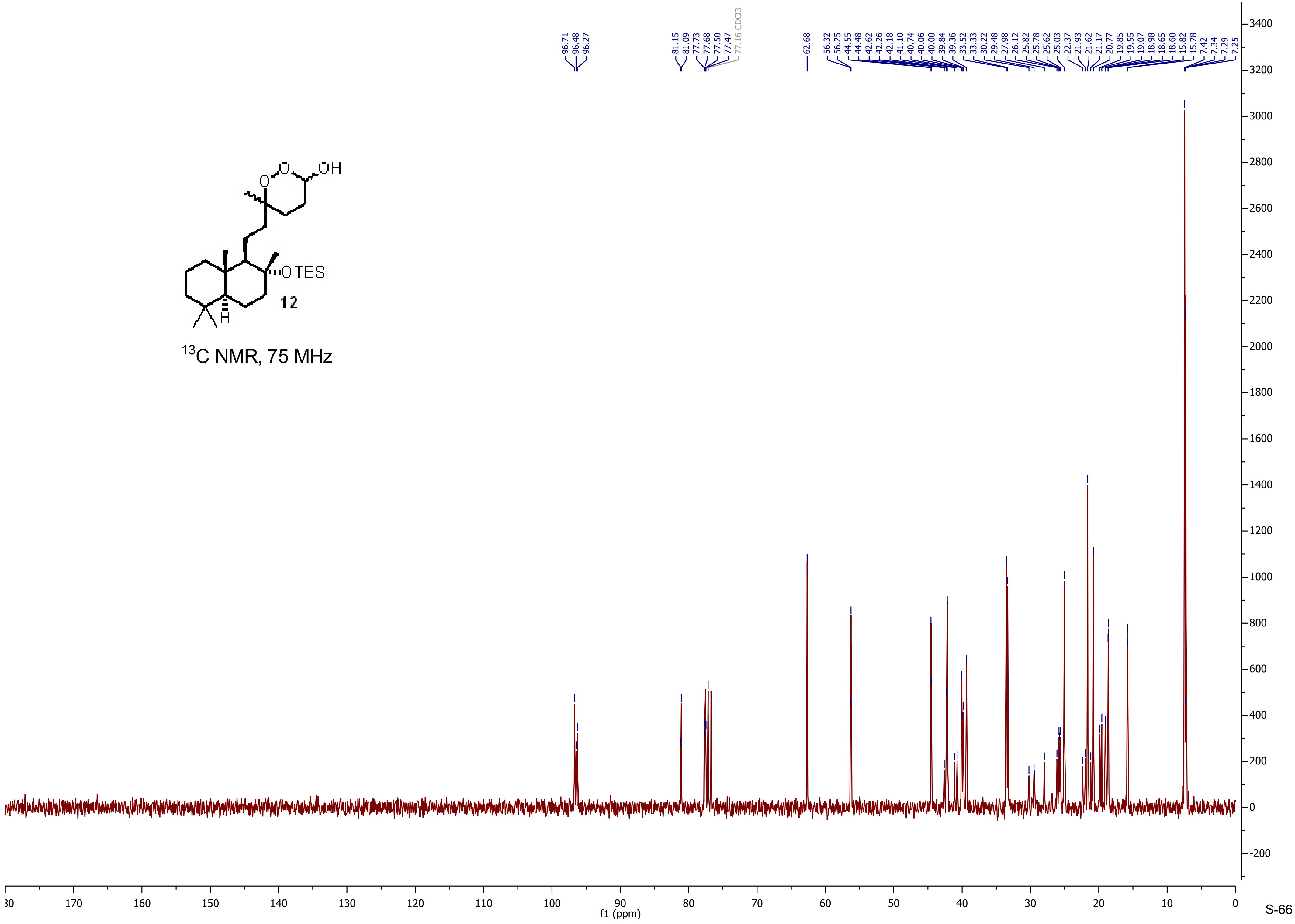
OH
O
O
OTES
H
12
13C NMR, 75 MHz
96.71
96.48
96.27
81.15
81.09
77.73
77.68
77.50
77.47
77.16 CDCl3
62.68
56.32
56.25
44.55
44.48
42.62
42.26
42.18
41.10
40.74
40.06
40.00
39.84
39.36
33.52
33.33
30.22
29.48
27.98
26.12
25.82
25.78
25.62
25.03
22.37
21.93
21.62
21.17
20.77
19.85
19.55
19.07
18.98
18.65
18.60
15.82
15.78
7.42
7.34
7.29
7.25
f1 (ppm)

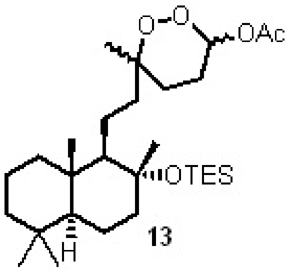

^{1}H NMR, 300 MHz

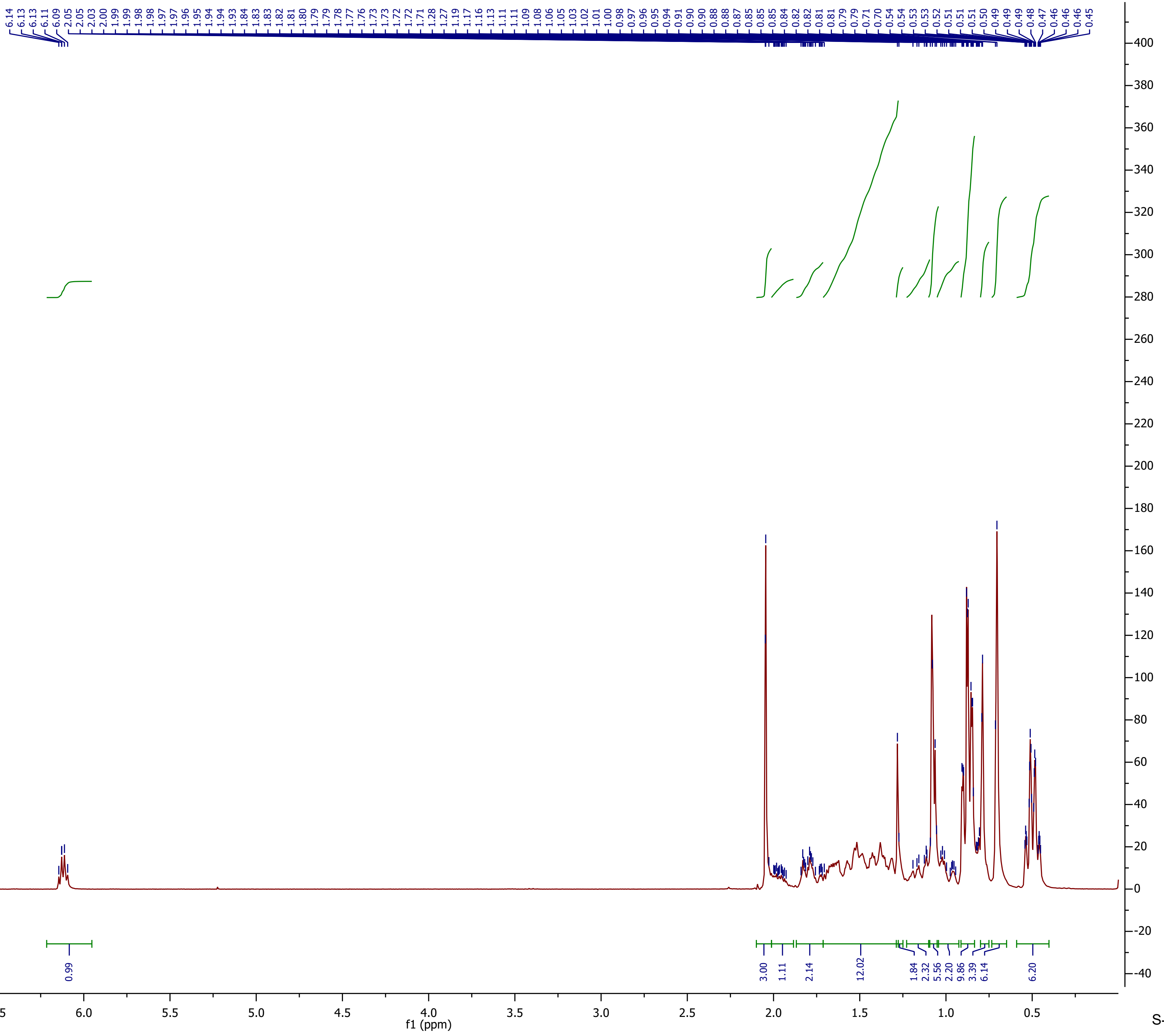

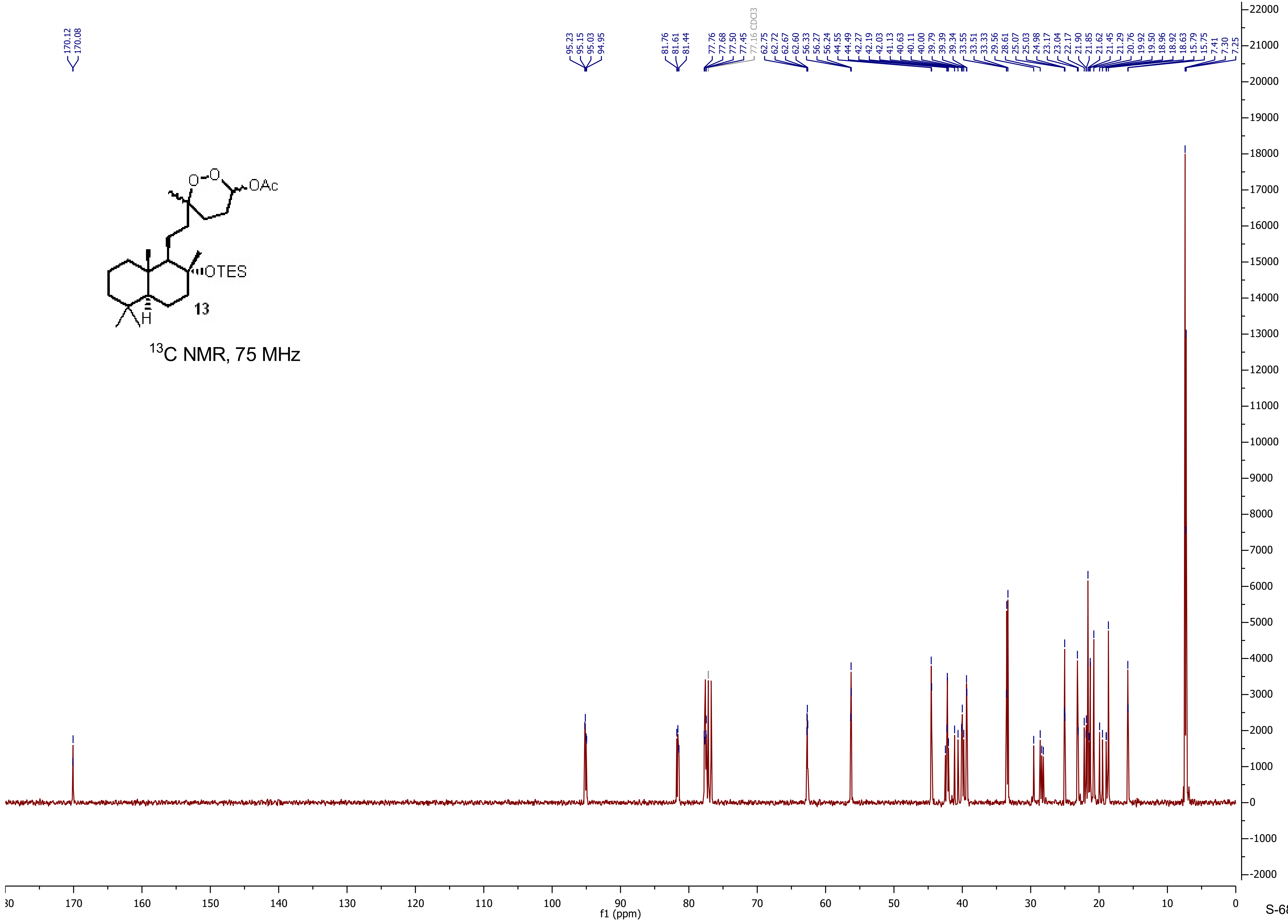
170.12
170.08
95.23
95.15
95.03
94.95
81.76
81.61
81.44
77.76
77.68
77.50
77.45
77.16 CDCl3
62.75
62.72
62.67
62.60
56.33
56.27
56.24
44.55
44.49
42.27
42.19
42.03
41.13
40.63
40.11
40.00
39.79
39.39
39.34
33.55
33.51
33.33
29.56
28.61
25.07
25.03
24.98
23.17
23.04
22.17
21.90
21.85
21.62
21.45
21.29
20.76
19.92
19.50
18.96
18.92
18.63
15.79
15.75
7.41
7.30
7.25
O
O
OAc
OTES
H
13
^{13}C NMR, 75 MHz
f1 (ppm)

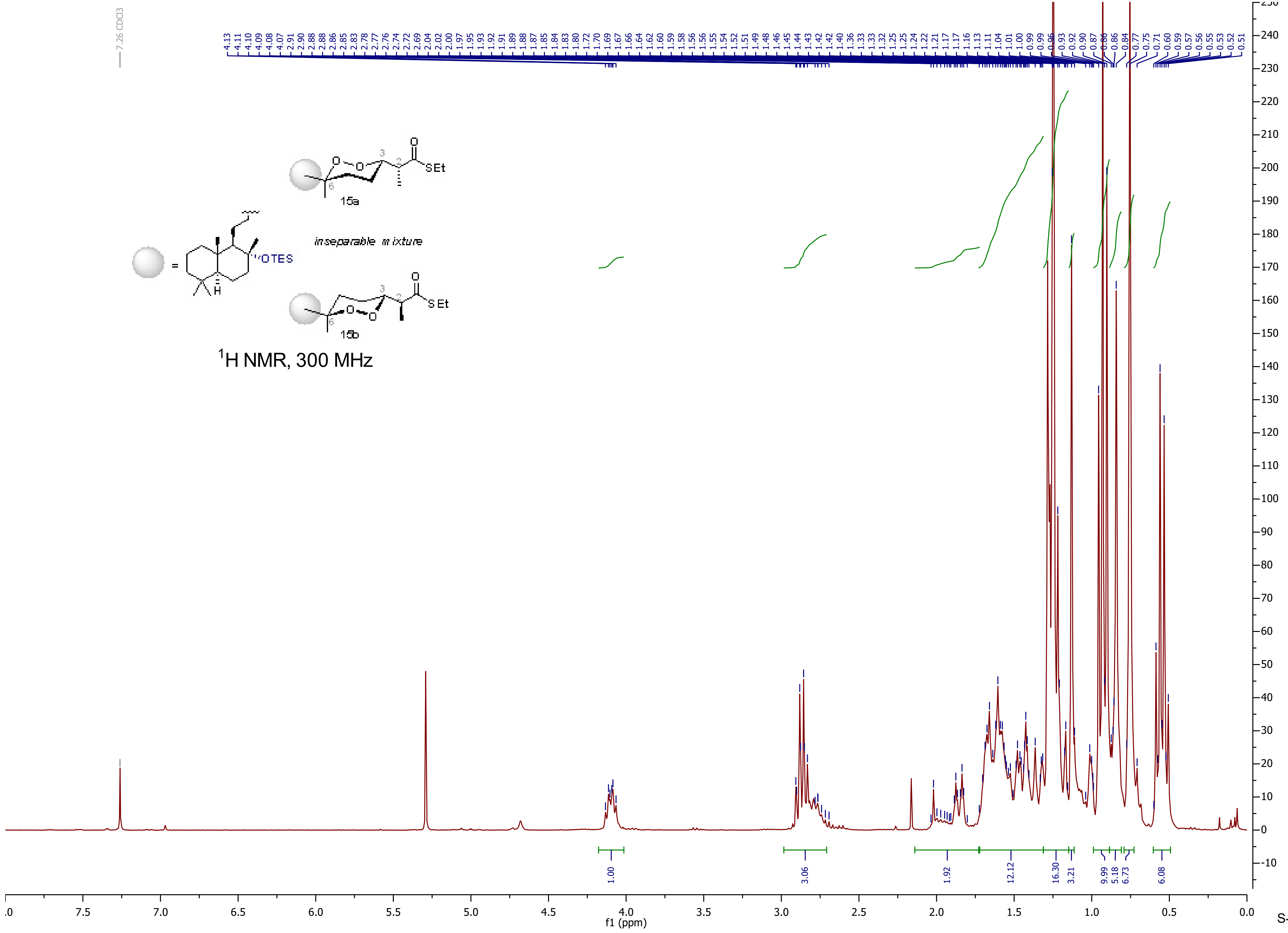

^1H NMR, 300 MHz

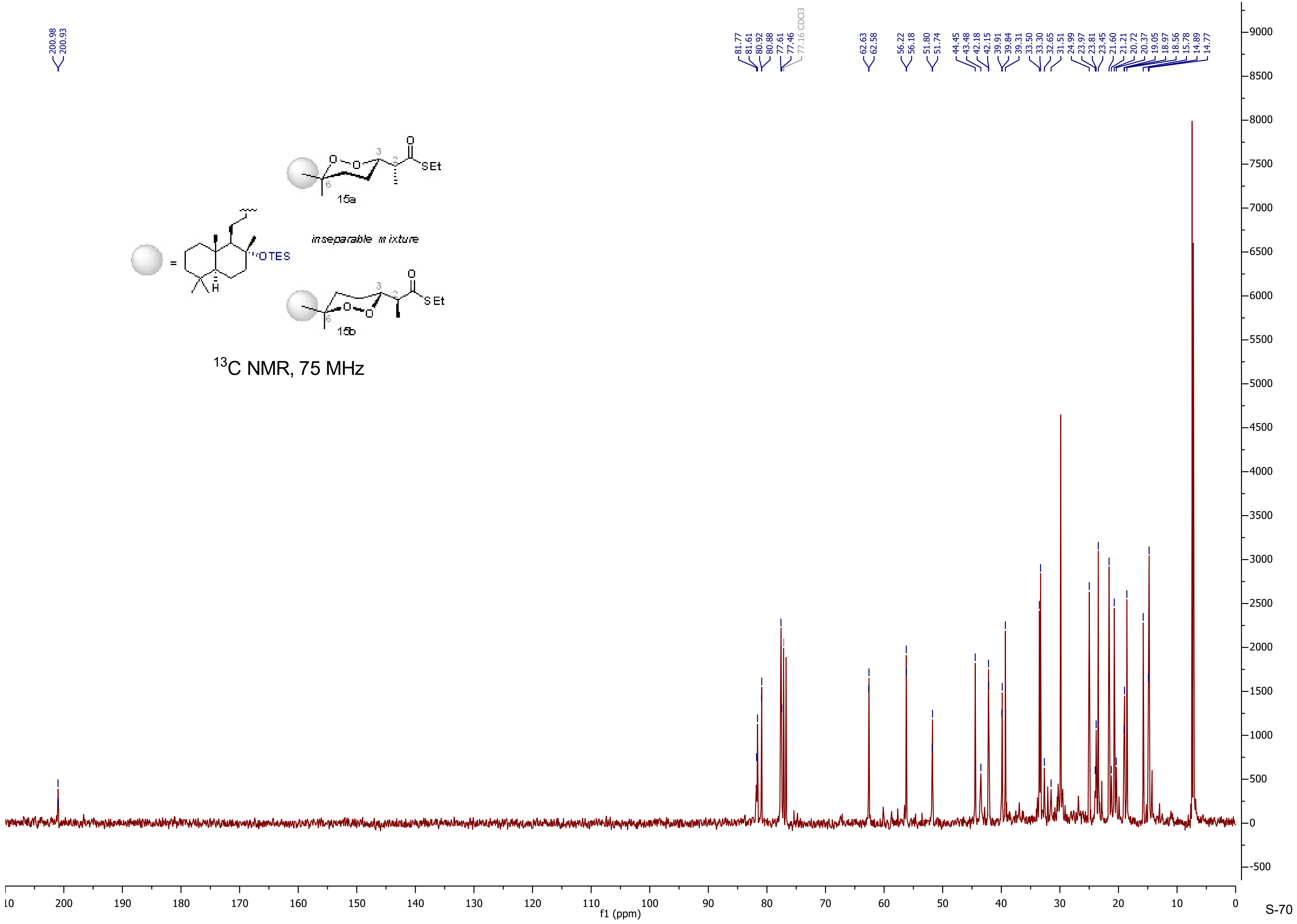
200.98
200.93
81.77
81.61
80.92
80.88
77.61
77.46
77.16 CDCl3
62.63
62.58
56.22
56.18
51.80
51.74
44.45
43.48
42.18
42.15
39.91
39.84
39.31
33.50
33.30
32.65
31.51
24.99
23.97
23.81
23.45
21.60
21.21
20.72
20.37
19.05
18.97
18.56
15.78
14.89
14.77
SEt
15a
OTES
inseparable mixture
15b
13C NMR, 75 MHz
f1 (ppm)

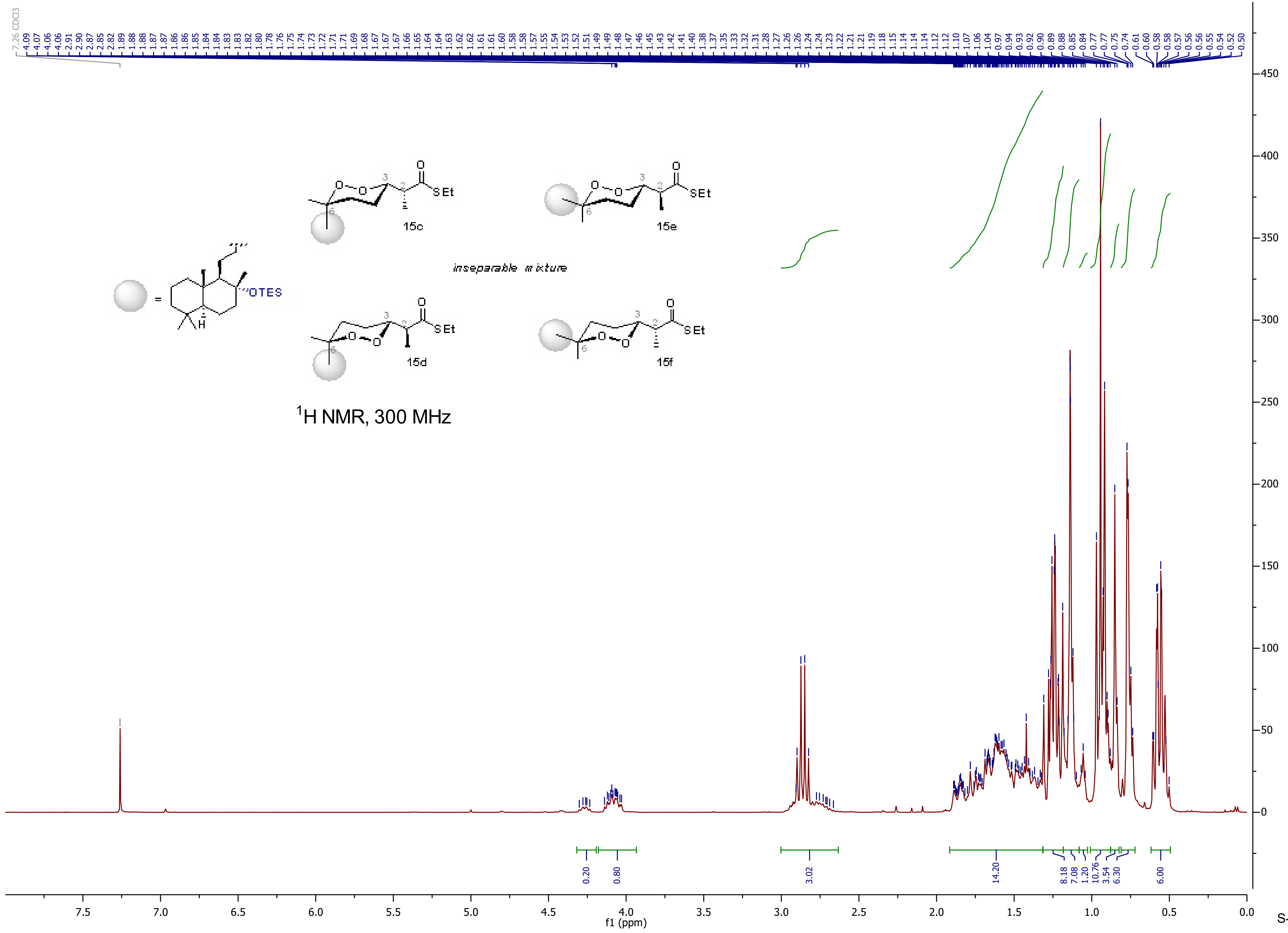
15c
15e
15d
15f
SEt
OTES
inseparable mixture
¹H NMR, 300 MHz
0.20
0.80
3.02
14.20
8.18
7.08
1.20
10.76
3.54
6.30
6.00
f1 (ppm)

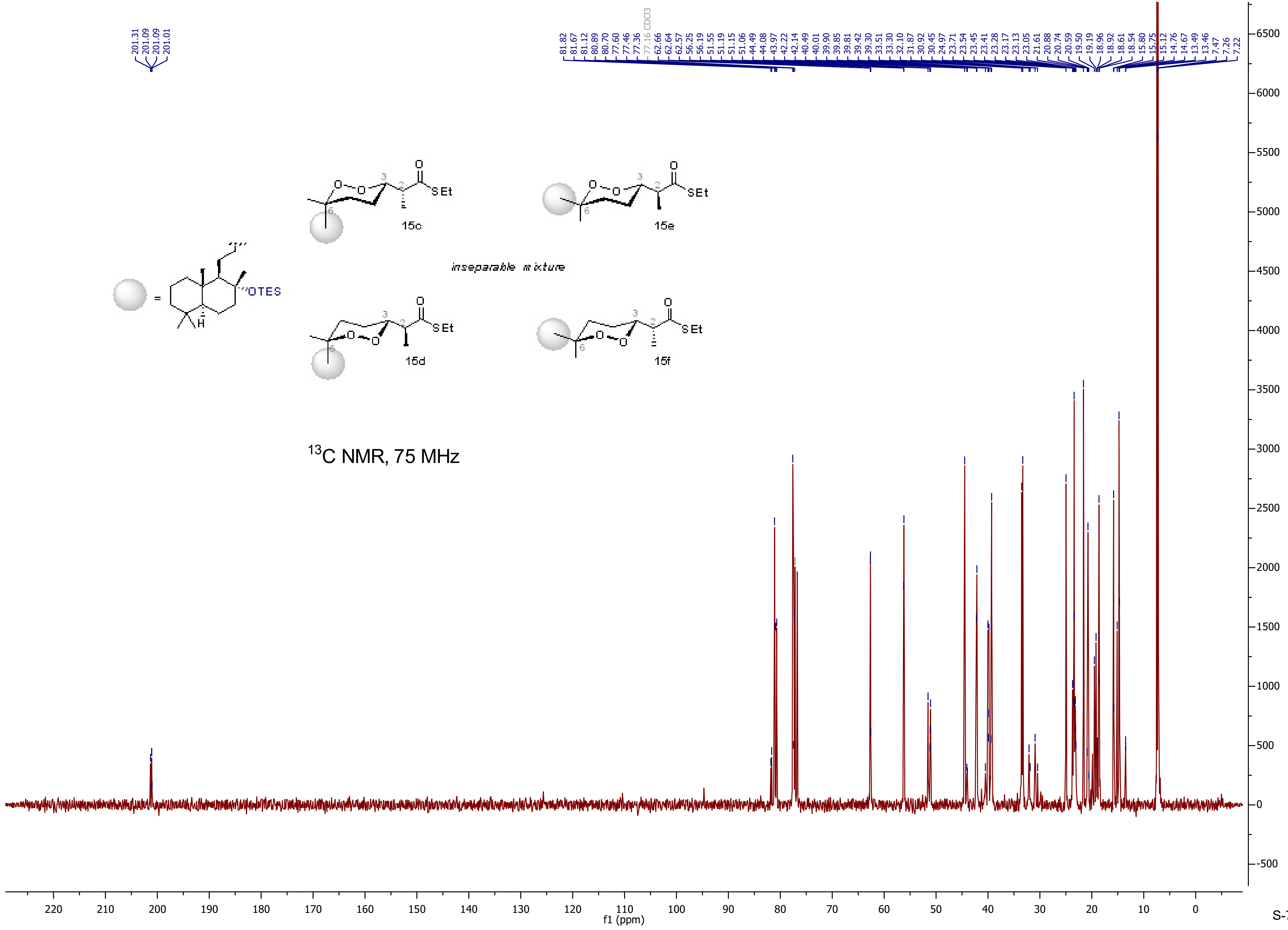
201.31
201.09
201.09
201.01
81.82 81.67 81.12 80.89 80.70 77.60 77.46 77.36 77.16 CDCl3 62.66 62.64 62.57 56.25 56.19 51.55 51.19 51.15 51.06 44.49 44.08 43.97 42.22 42.14 40.49 40.01 39.90 39.85 39.81 39.42 39.30 33.51 33.30 32.10 31.87 30.92 30.45 24.97 23.71 23.54 23.45 23.41 23.28 23.17 23.13 23.05 21.61 20.88 20.74 20.59 19.50 19.19 18.96 18.92 18.61 18.54 15.80 15.75 15.12 14.76 14.67 13.49 13.46 7.47 7.26 7.22
OTES
SEt
15c
15e
15d
15f
inseparable mixture
^{13}C NMR, 75 MHz
f1 (ppm)

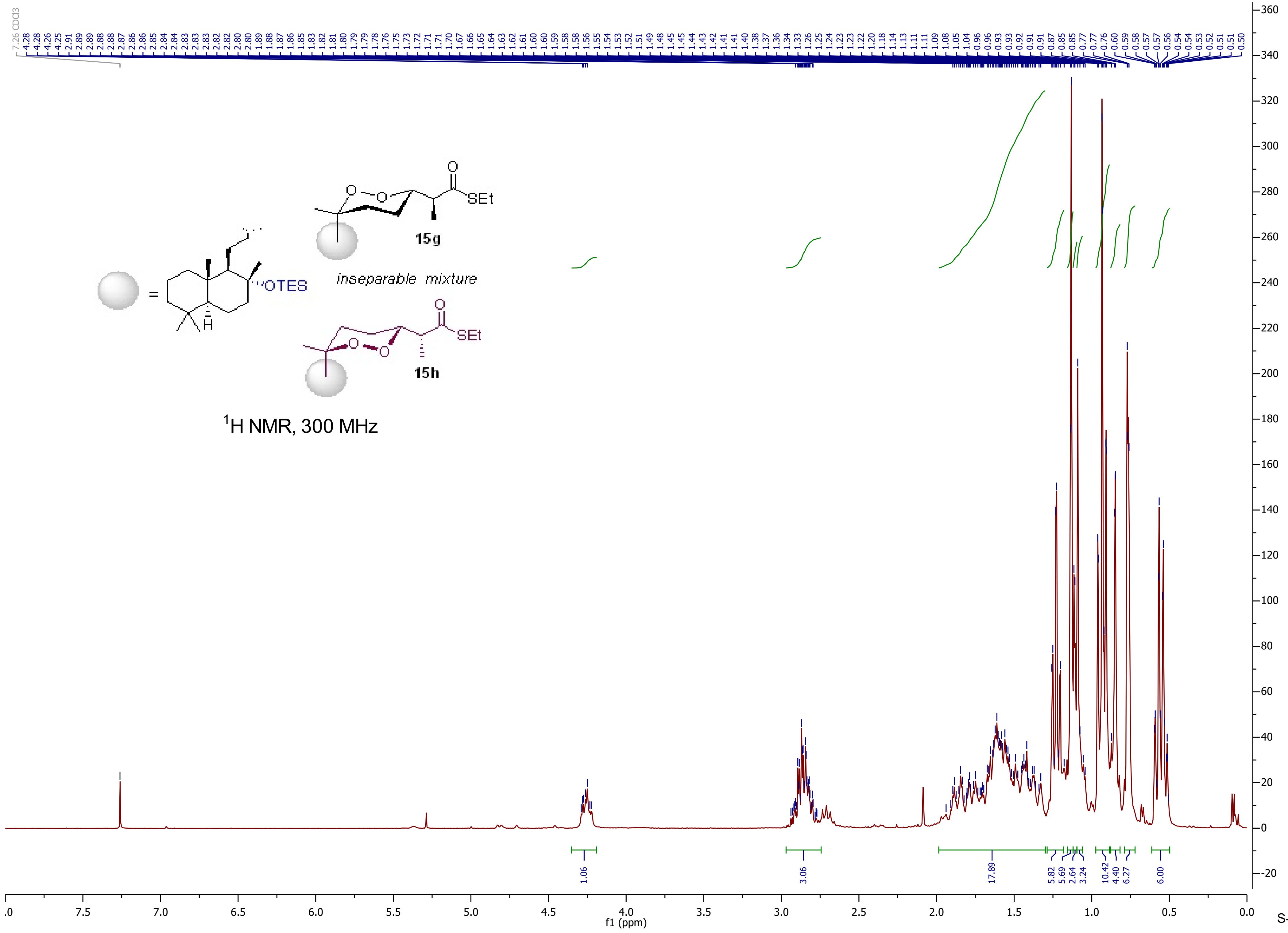
15g
inseparable mixture
15h
OTES
SEt
^{1}H NMR, 300 MHz
f1 (ppm)

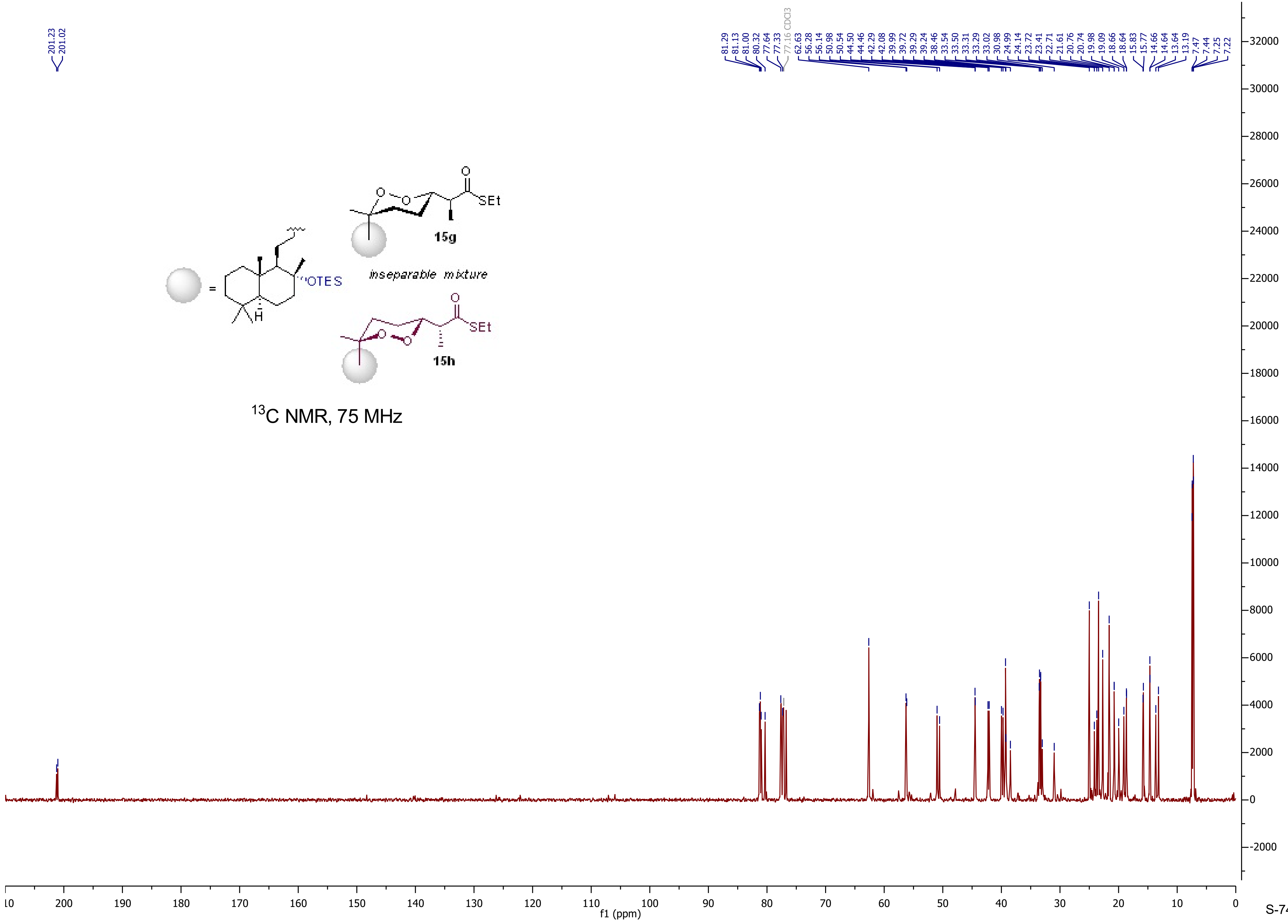
201.23
201.02
81.29
81.13
81.00
80.32
77.64
77.33
77.16 CDCl3
62.63
56.28
56.14
50.98
50.54
44.50
44.46
42.29
42.08
39.99
39.72
39.29
39.24
38.46
33.54
33.50
33.31
33.29
33.02
30.98
24.99
24.14
23.72
23.41
22.71
21.61
20.76
20.74
19.98
19.09
18.66
18.64
15.83
15.77
14.66
14.64
13.64
13.19
7.47
7.44
7.25
7.22
SEt
15g
OTES
inseparable mixture
15h
¹³C NMR, 75 MHz
f1 (ppm)

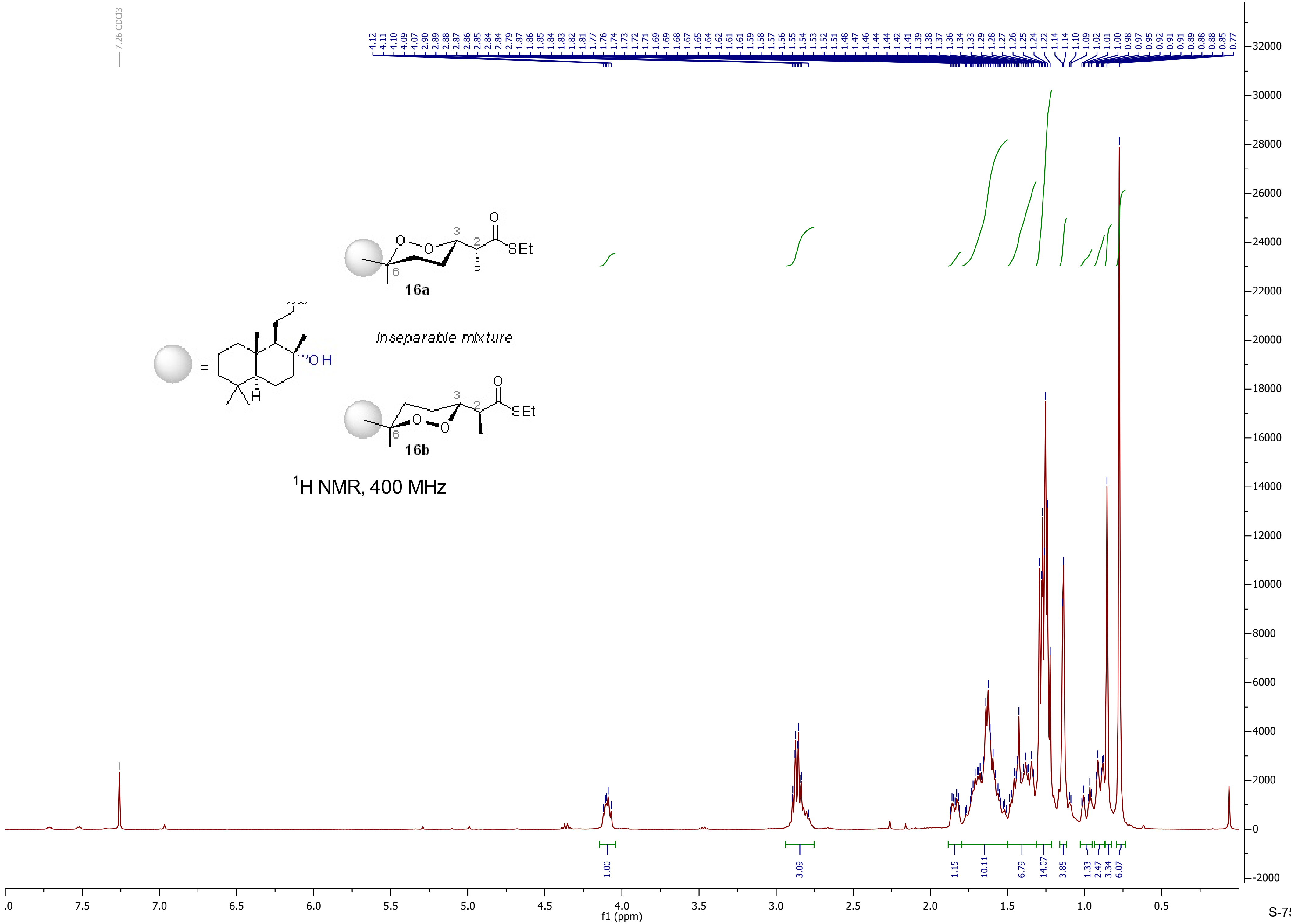
7.26 CDCl3
16a
16b
inseparable mixture
SEt
OH
1H NMR, 400 MHz
f1 (ppm)

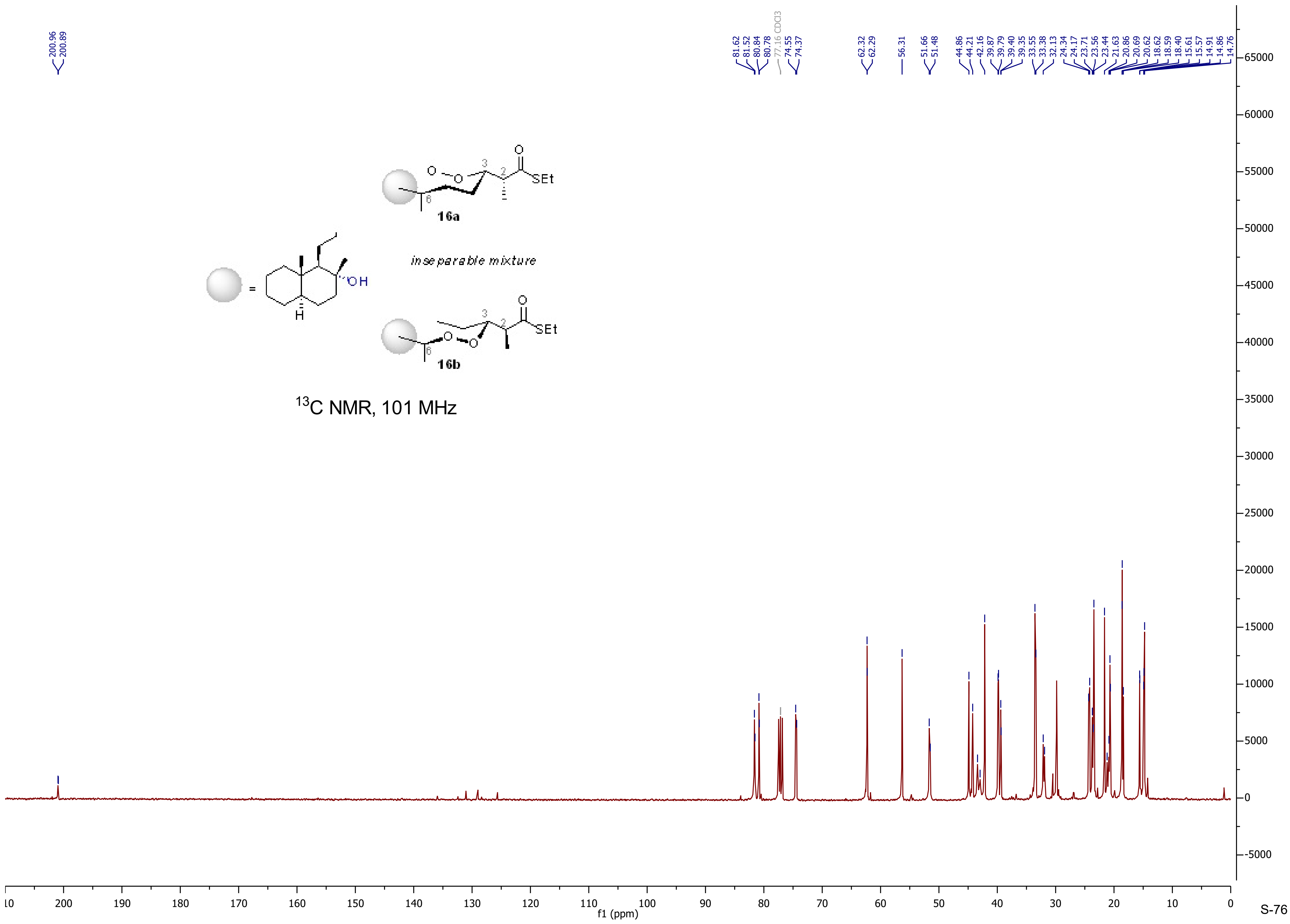

^{13}C NMR, 101 MHz

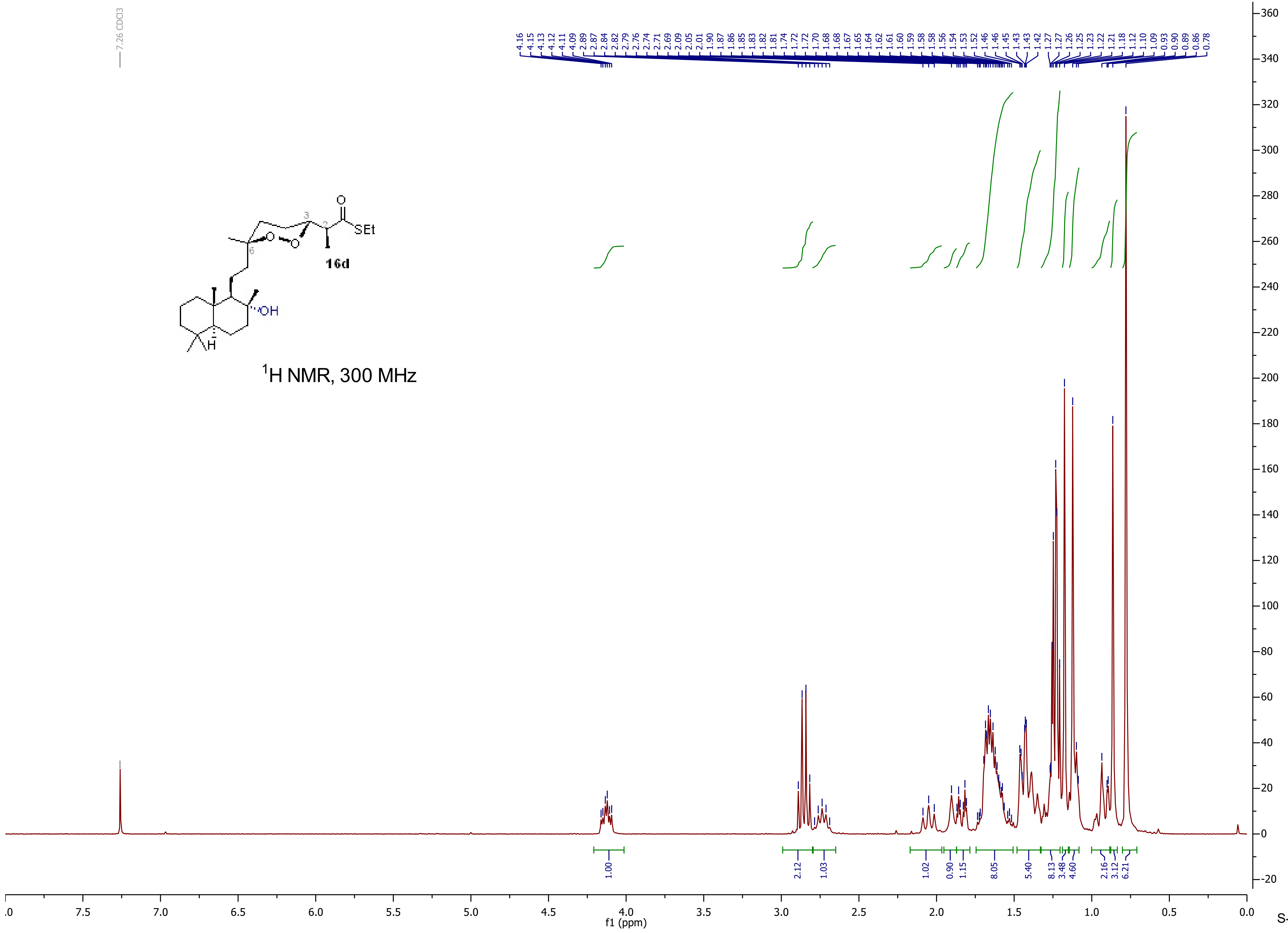

7.26 CDCl3
4.16 4.15 4.13 4.12 4.11 4.09 2.89 2.87 2.84 2.82 2.79 2.76 2.74 2.71 2.69 2.09 2.05 2.01 1.90 1.87 1.86 1.85 1.83 1.82 1.81 1.74 1.72 1.72 1.70 1.68 1.67 1.65 1.64 1.62 1.61 1.60 1.59 1.58 1.58 1.56 1.54 1.53 1.52 1.46 1.46 1.45 1.43 1.43 1.42 1.27 1.27 1.26 1.25 1.23 1.22 1.21 1.18 1.12 1.10 1.09 0.93 0.90 0.89 0.86 0.78
SEt
OH
H
16d
1H NMR, 300 MHz
1.00
2.12
1.03
1.02
0.90
1.15
8.05
5.40
8.13
3.48
4.60
2.16
3.12
6.21
f1 (ppm)

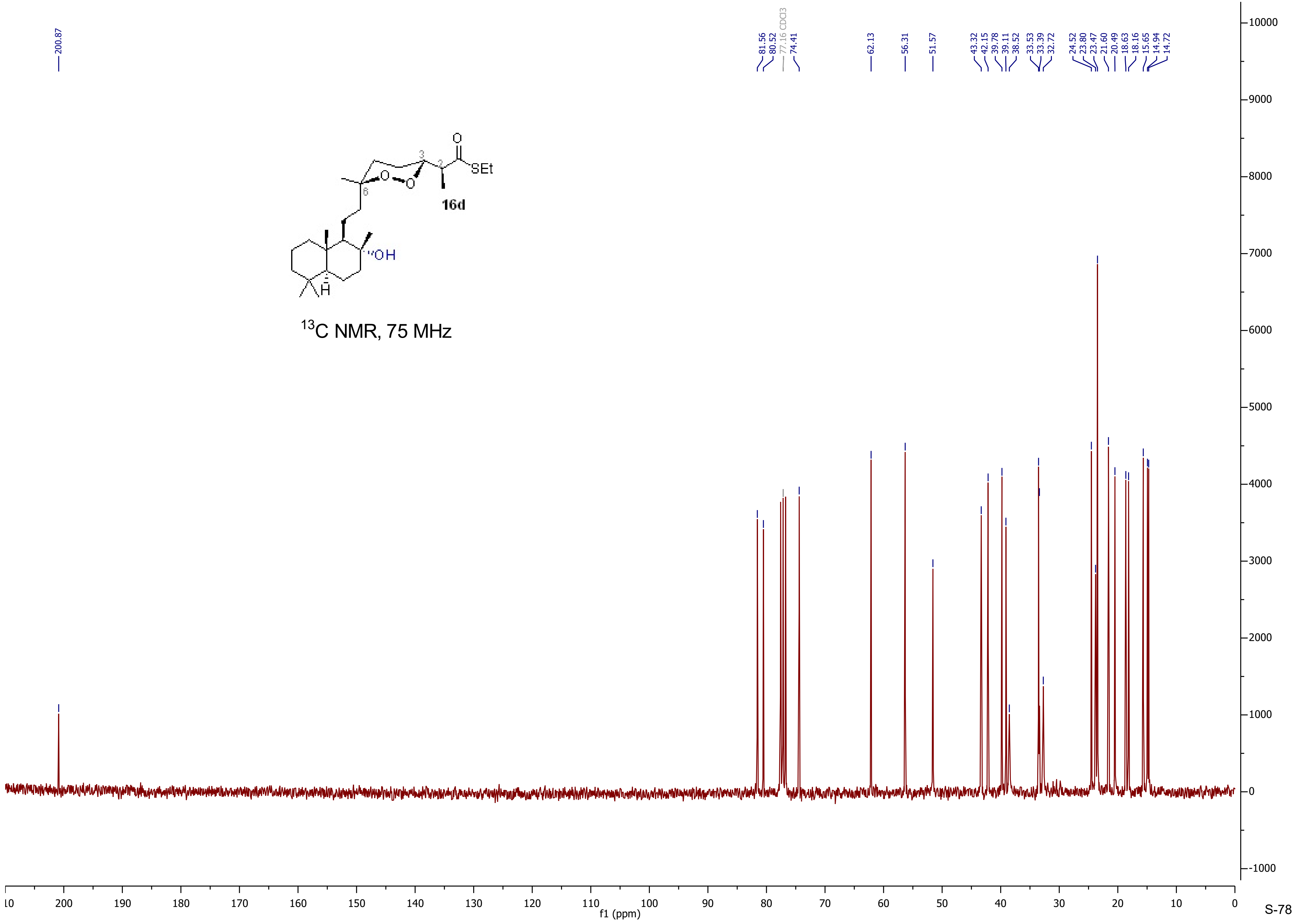
200.87
81.56
80.52
77.16 CDCl3
74.41
62.13
56.31
51.57
43.32
42.15
39.78
39.11
38.52
33.53
33.39
32.72
24.52
23.80
23.47
21.60
20.49
18.63
18.16
15.65
14.94
14.72
16d
^{13}C NMR, 75 MHz
f1 (ppm)

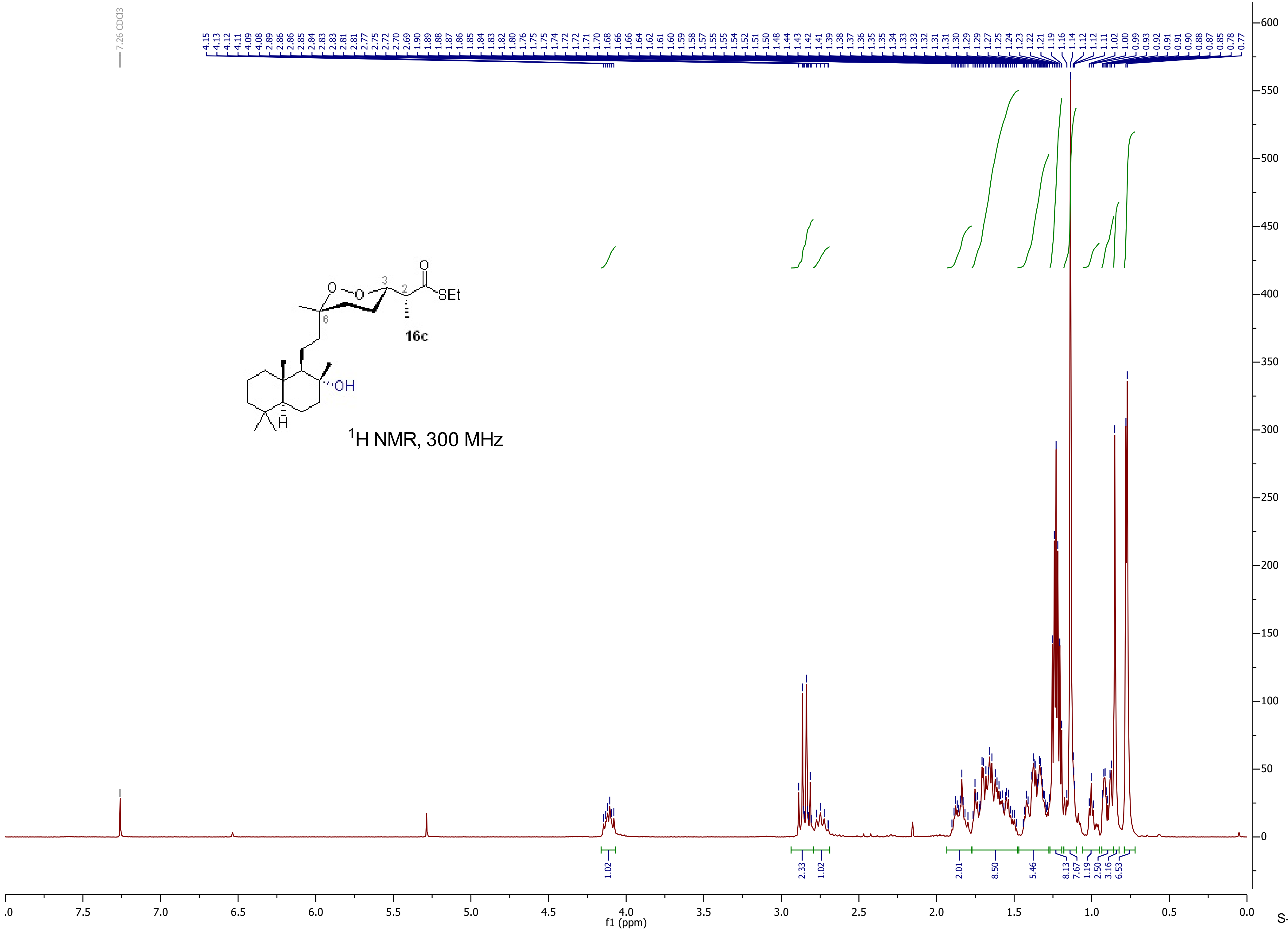
7.26 CDCl3
O
SEt
3
2
6
16c
OH
H
^{1}H NMR, 300 MHz
1.02
2.33
1.02
2.01
8.50
5.46
8.13
7.67
1.19
2.50
3.16
6.53
f1 (ppm)

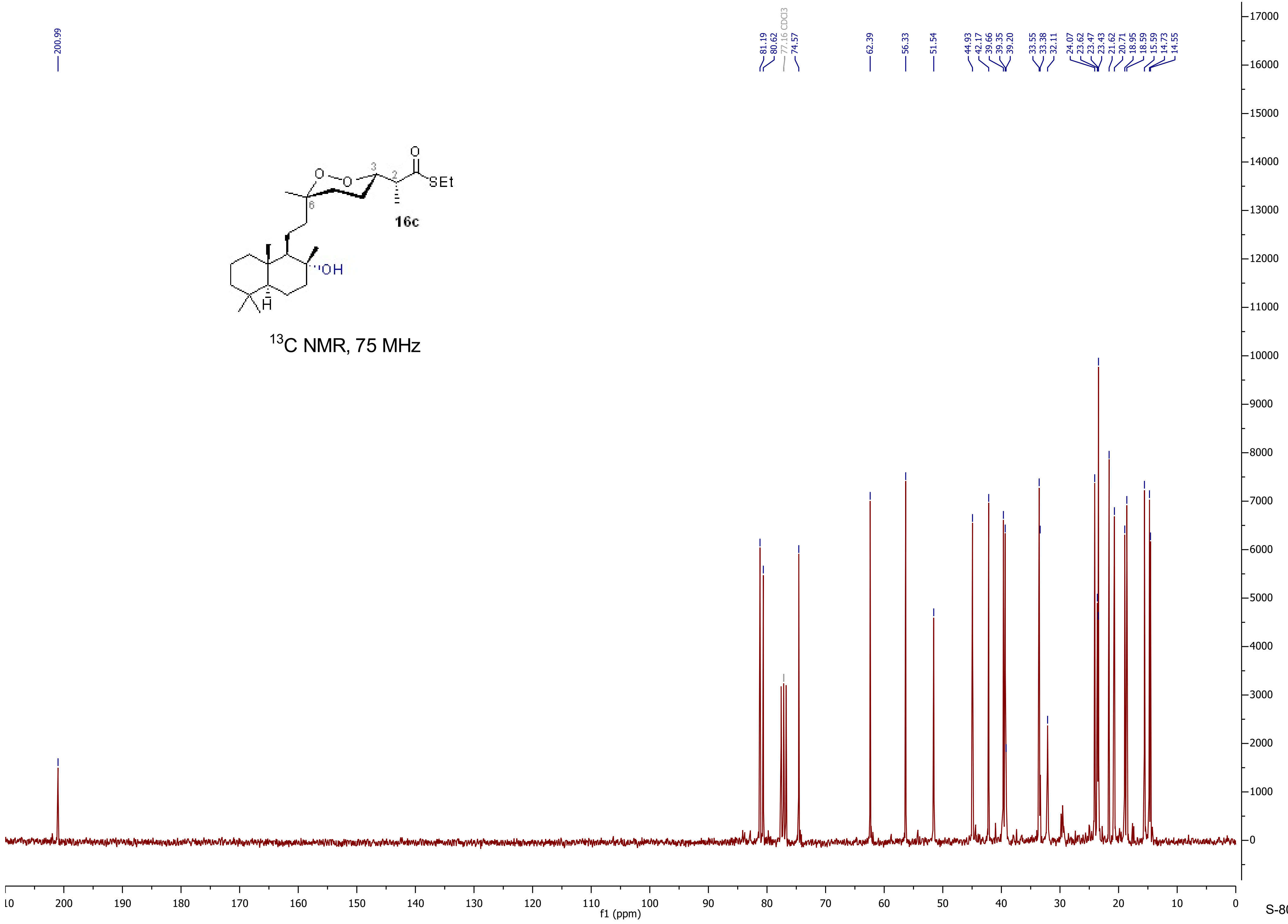
200.99
81.19
80.62
77.16 CDCl3
74.57
62.39
56.33
51.54
44.93
42.17
39.66
39.35
39.20
33.55
33.38
32.11
24.07
23.62
23.47
23.43
21.62
20.71
18.95
18.59
15.59
14.73
14.55
SEt
OH
16c
13C NMR, 75 MHz
f1 (ppm)

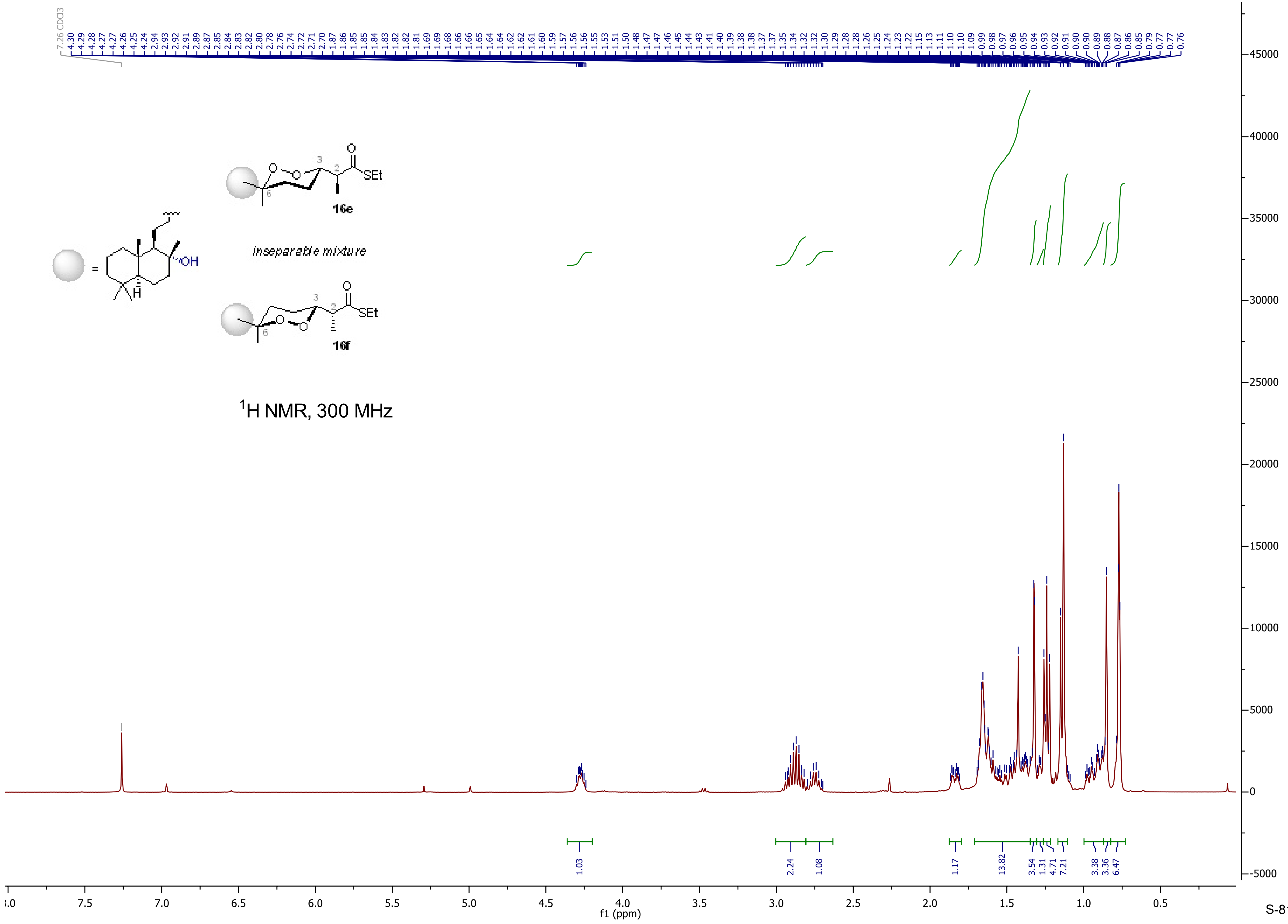
16e
Inseparable mixture
16f
1H NMR, 300 MHz
f1 (ppm)

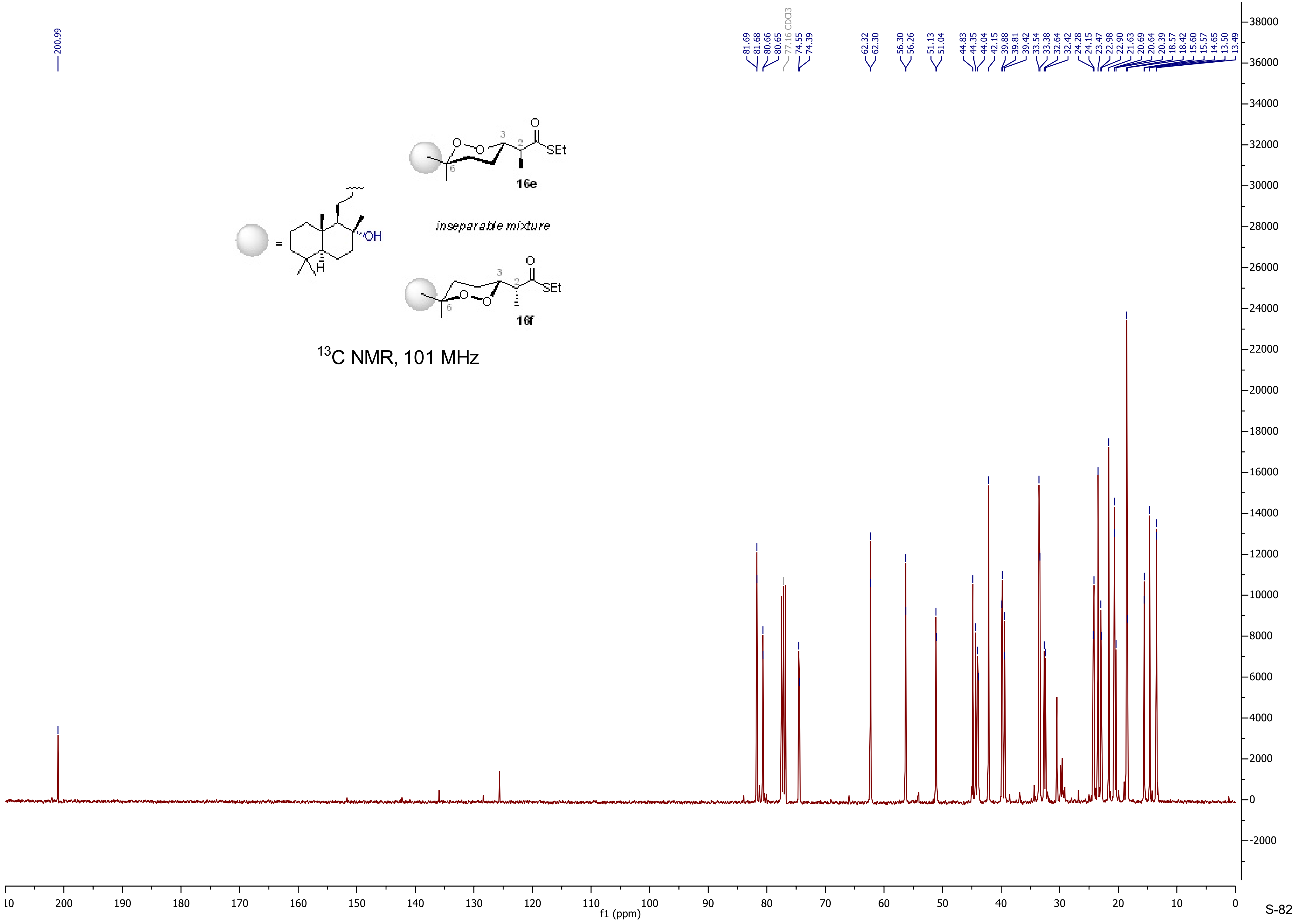

200.99
81.69
81.68
80.66
80.65
77.16 CDCl3
74.55
74.39
62.32
62.30
56.30
56.26
51.13
51.04
44.83
44.35
44.04
42.15
39.88
39.81
39.42
33.54
33.38
32.64
32.42
24.28
24.15
23.47
22.98
22.90
21.63
20.69
20.64
20.39
18.57
18.42
15.60
15.57
14.65
13.50
13.49
OH
H
SEt
16e
inseparable mixture
16f
13C NMR, 101 MHz
f1 (ppm)

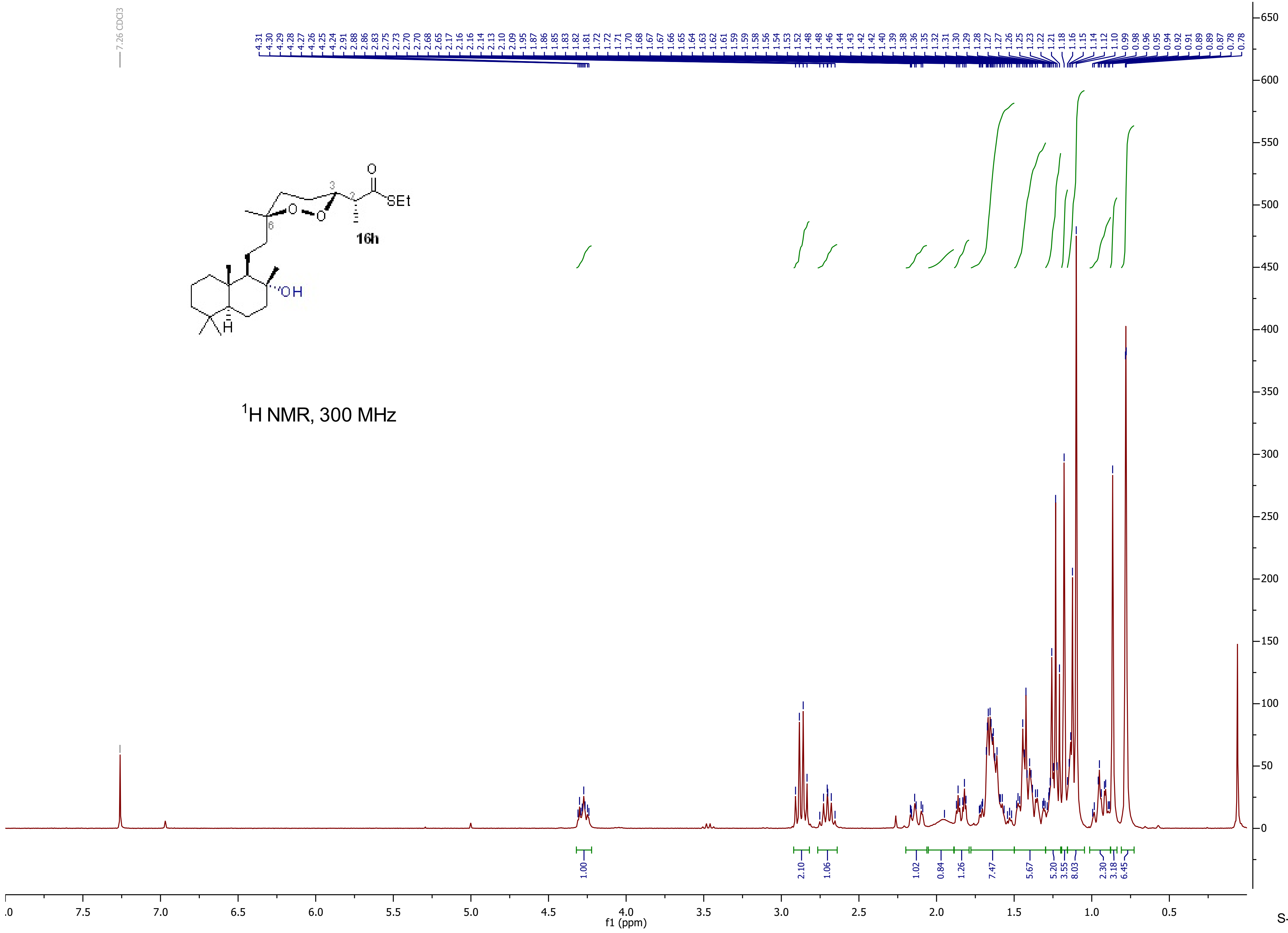

O
SEt
3
2
6
O
O
OH
H
16h
¹H NMR, 300 MHz
7.26 CDCl3
f1 (ppm)

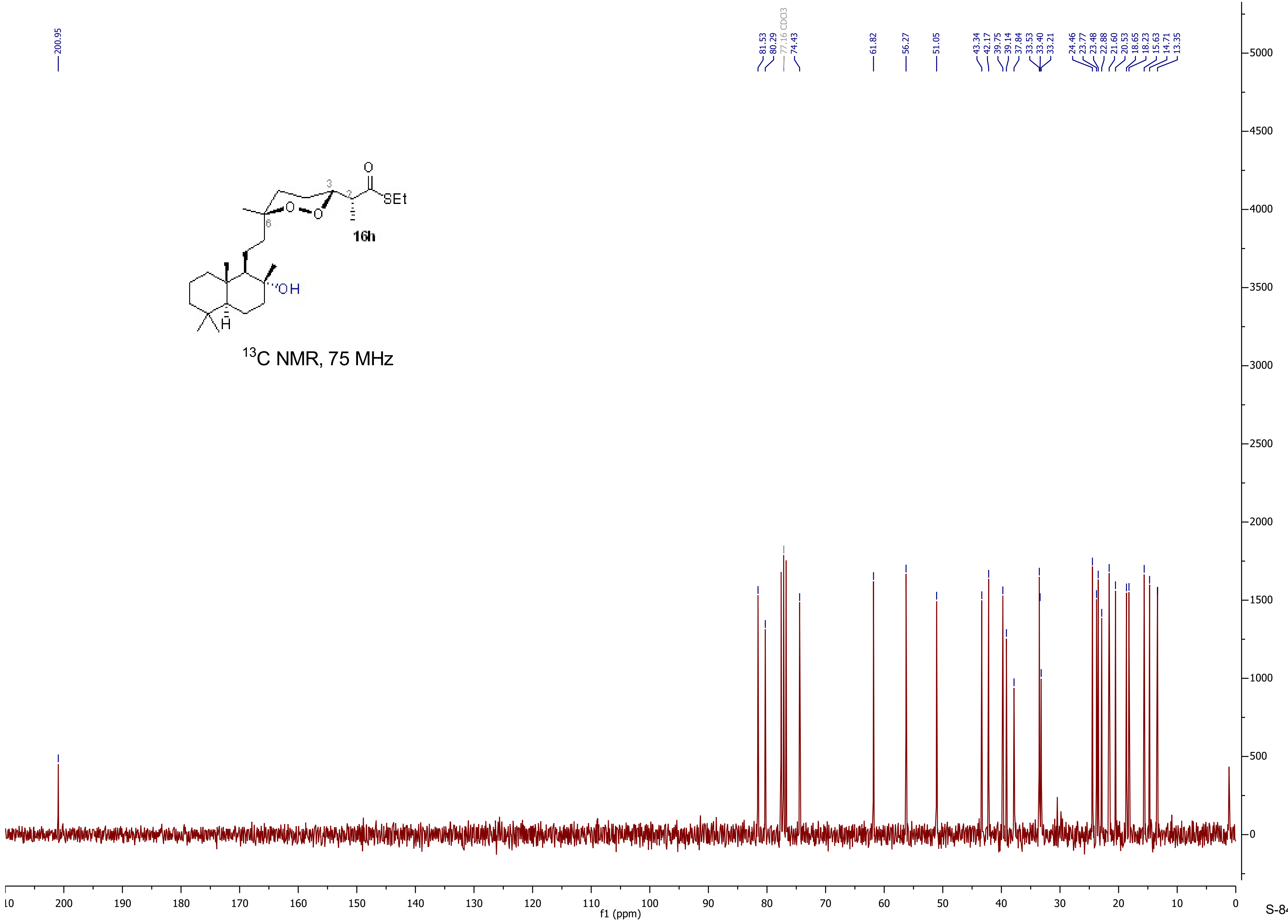
200.95
81.53
80.29
77.16 CDCl3
74.43
61.82
56.27
51.05
43.34
42.17
39.75
39.14
37.84
33.53
33.40
33.21
24.46
23.77
23.48
22.88
21.60
20.53
18.65
18.23
15.63
14.71
13.35
O
SEt
OH
H
O
O
3
2
6
16h
13C NMR, 75 MHz
f1 (ppm)

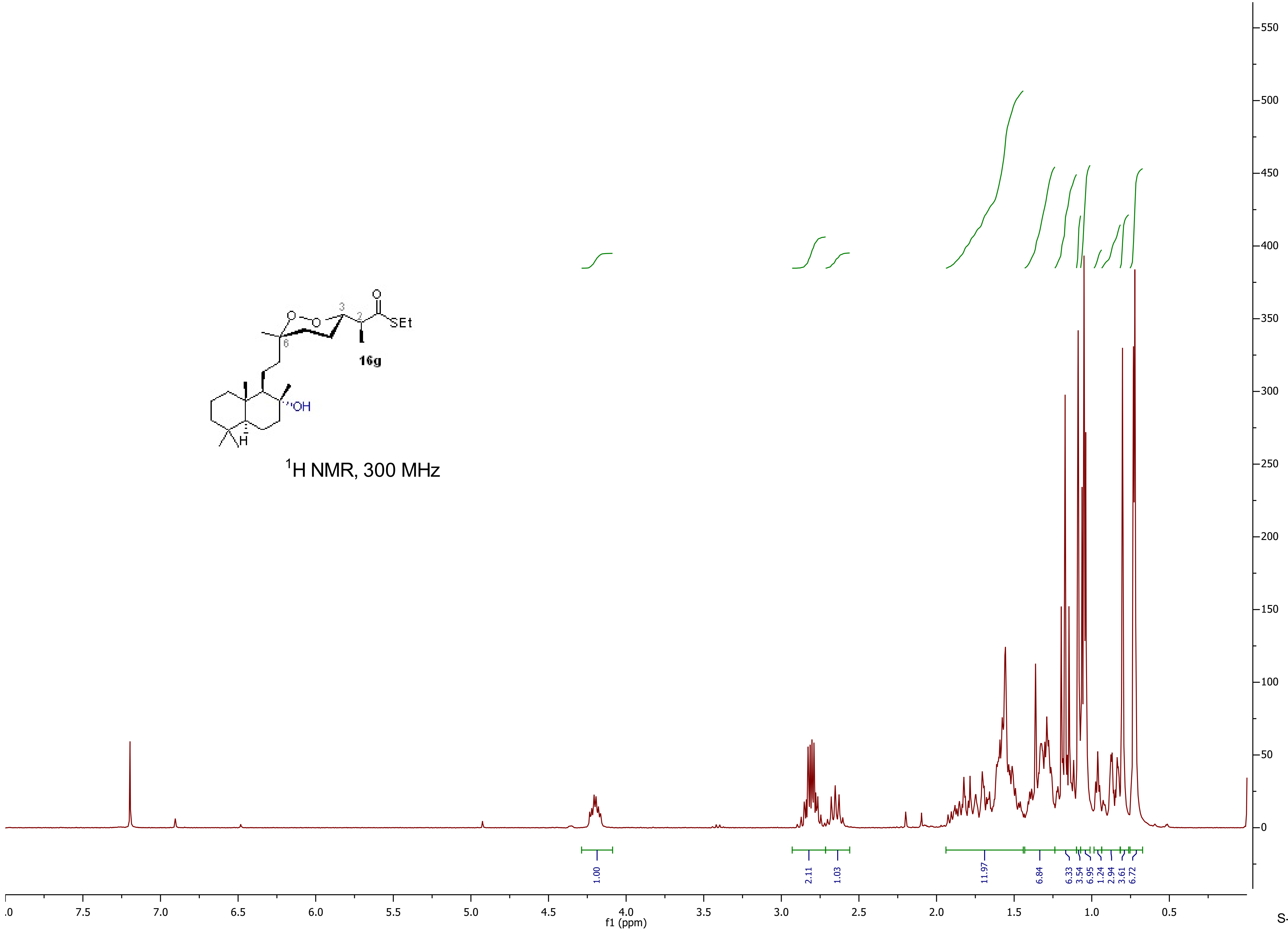

16g
SEt
OH
H
1H NMR, 300 MHz
1.00
2.11
1.03
11.97
6.84
6.33
3.54
6.95
1.24
2.94
3.61
6.72
f1 (ppm)

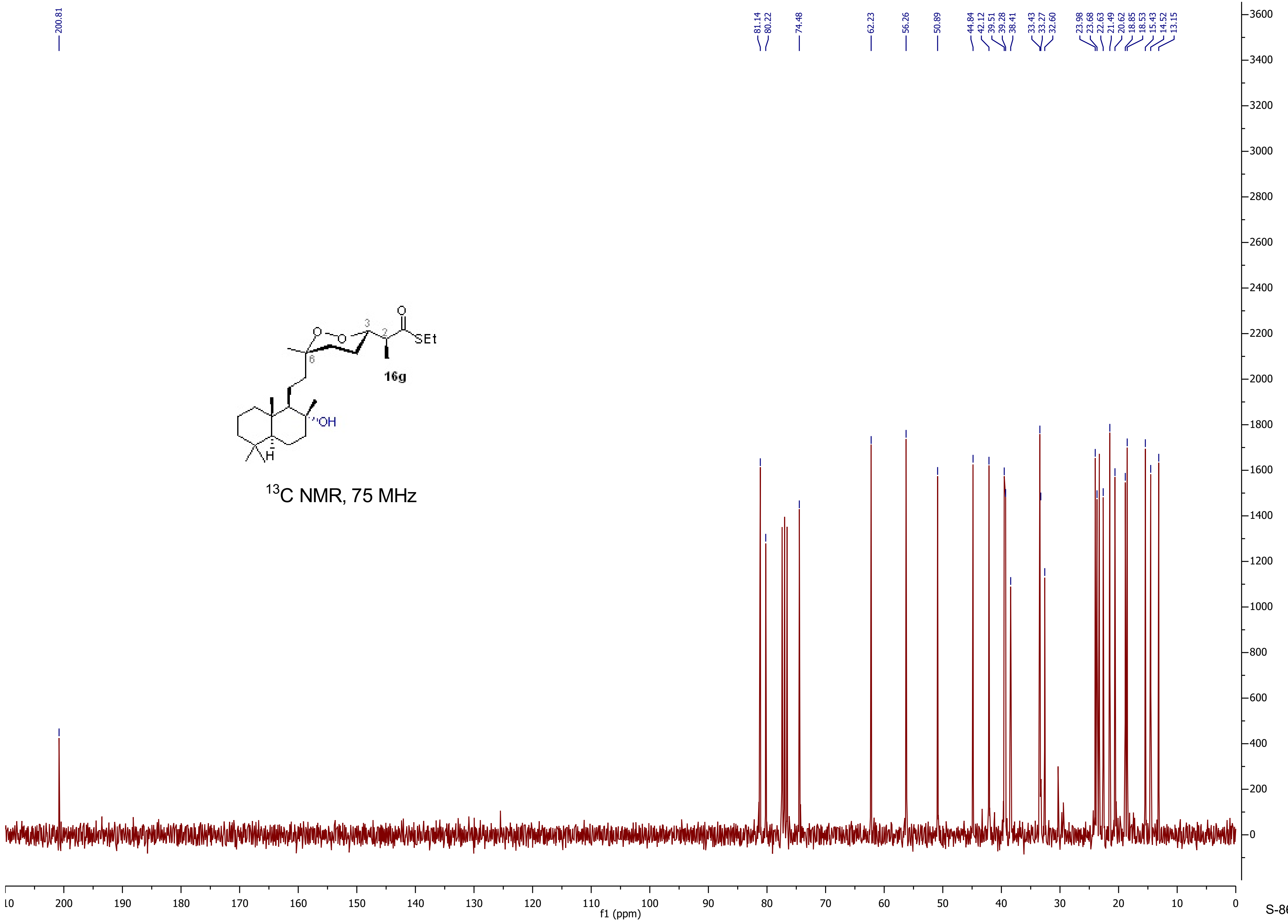
200.81
81.14
80.22
74.48
62.23
56.26
50.89
44.84
42.12
39.51
39.28
38.41
33.43
33.27
32.60
23.98
23.68
22.63
21.49
20.62
18.85
18.53
15.43
14.52
13.15
O
SEt
OH
H
16g
^{13}C NMR, 75 MHz
f1 (ppm)

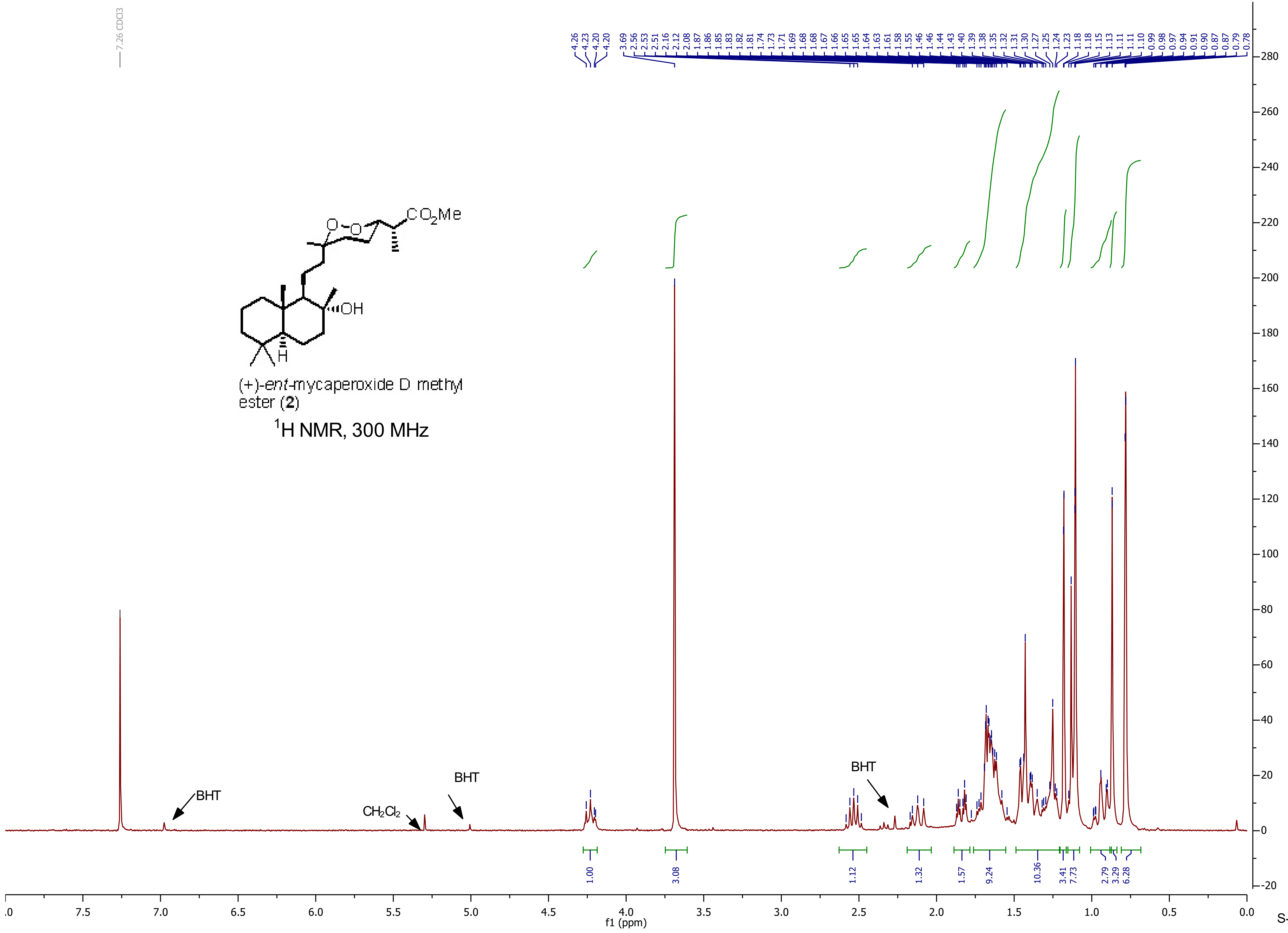

(+)-ent-mycaperoxide D methyl ester (2)
1H NMR, 300 MHz
CO2Me
OH
H
BHT
CH2Cl2
BHT
BHT
7.26 CDCl3
f1 (ppm)

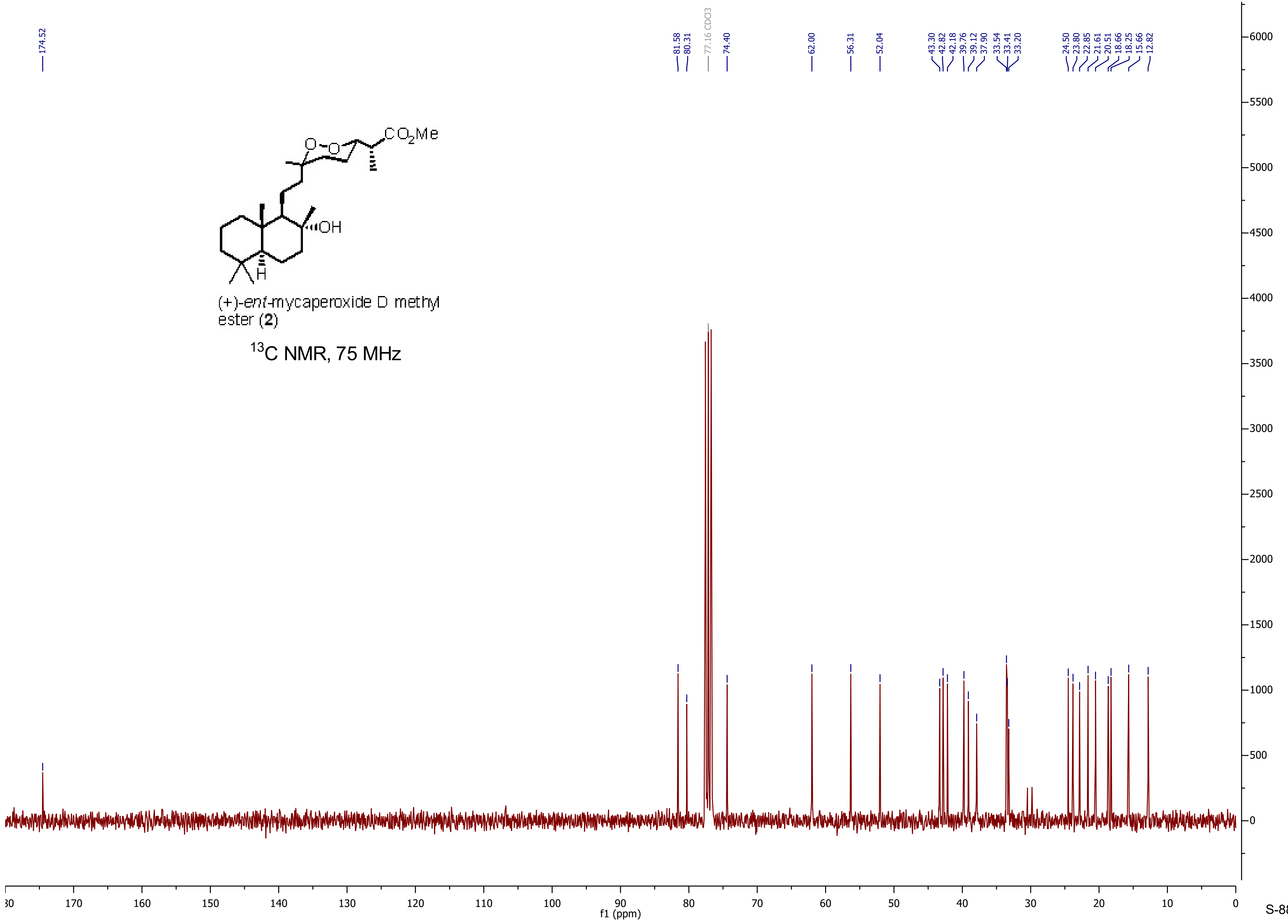

174.52
81.58
80.31
77.16 CDCl3
74.40
62.00
56.31
52.04
43.30
42.82
42.18
39.76
39.12
37.90
33.54
33.41
33.20
24.50
23.80
22.85
21.61
20.51
18.66
18.25
15.66
12.82
O-O
CO2Me
OH
H
(+)-ent-mycaperoxide D methyl ester (2)
13C NMR, 75 MHz
f1 (ppm)

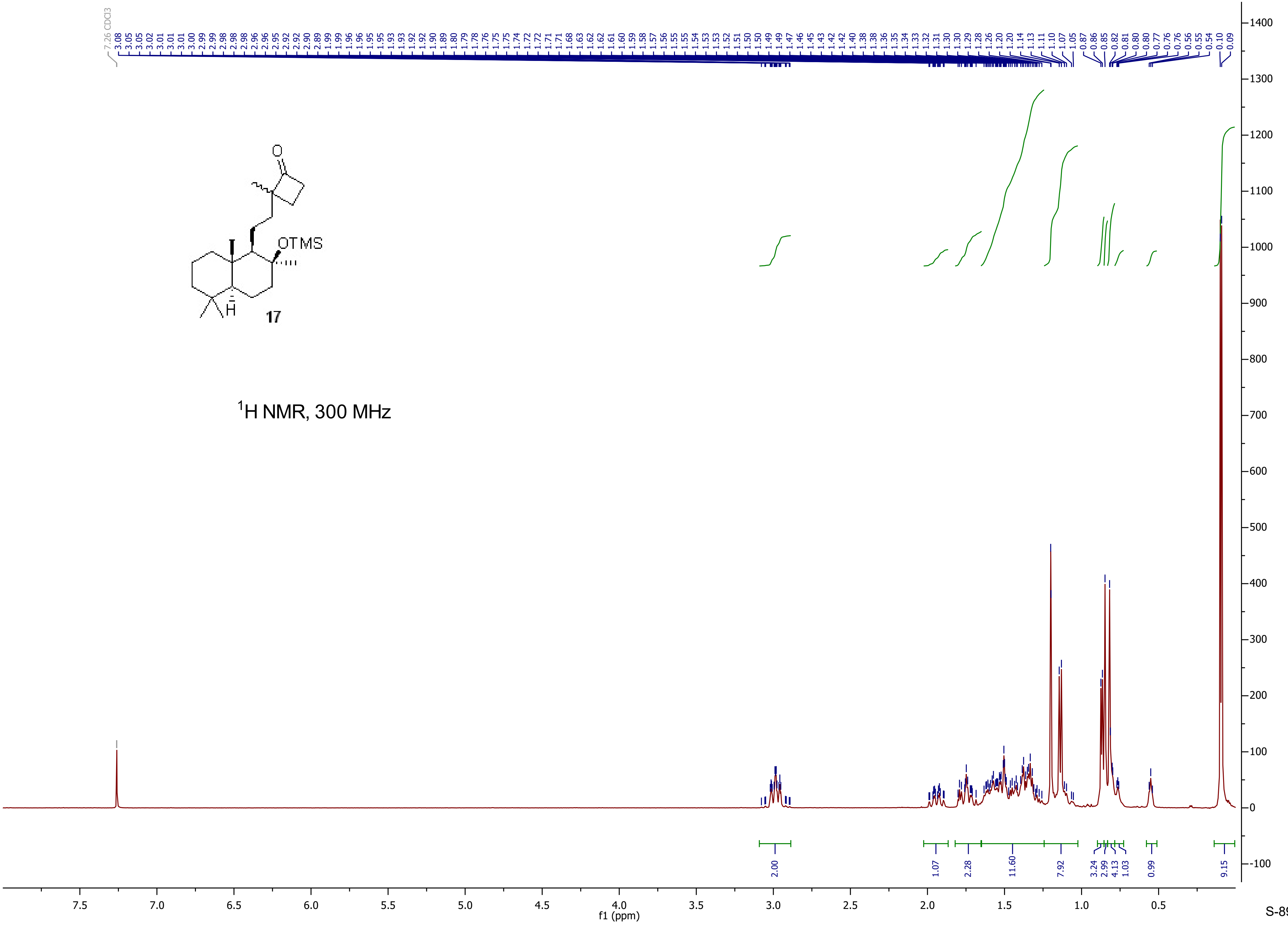
7.26 CDCl3
3.08 3.05 3.05 3.02 3.01 3.01 3.01 3.00 2.99 2.99 2.98 2.98 2.96 2.96 2.95 2.92 2.92 2.90 2.89 1.99 1.99 1.96 1.96 1.95 1.95 1.93 1.93 1.93 1.92 1.92 1.90 1.89 1.80 1.79 1.78 1.76 1.75 1.75 1.74 1.72 1.72 1.71 1.71 1.68 1.63 1.62 1.62 1.61 1.60 1.59 1.58 1.57 1.56 1.55 1.55 1.54 1.53 1.53 1.52 1.51 1.50 1.50 1.49 1.49 1.47 1.46 1.45 1.43 1.42 1.42 1.40 1.38 1.38 1.36 1.35 1.34 1.33 1.32 1.31 1.30 1.30 1.29 1.28 1.26 1.20 1.20 1.14 1.13 1.11 1.10 1.07 1.05 0.87 0.86 0.85 0.82 0.81 0.80 0.80 0.77 0.76 0.56 0.55 0.54 0.10 0.09
O
OTMS
H
17
1H NMR, 300 MHz
2.00 1.07 2.28 11.60 7.92 3.24 2.99 4.13 1.03 0.99 9.15
f1 (ppm)

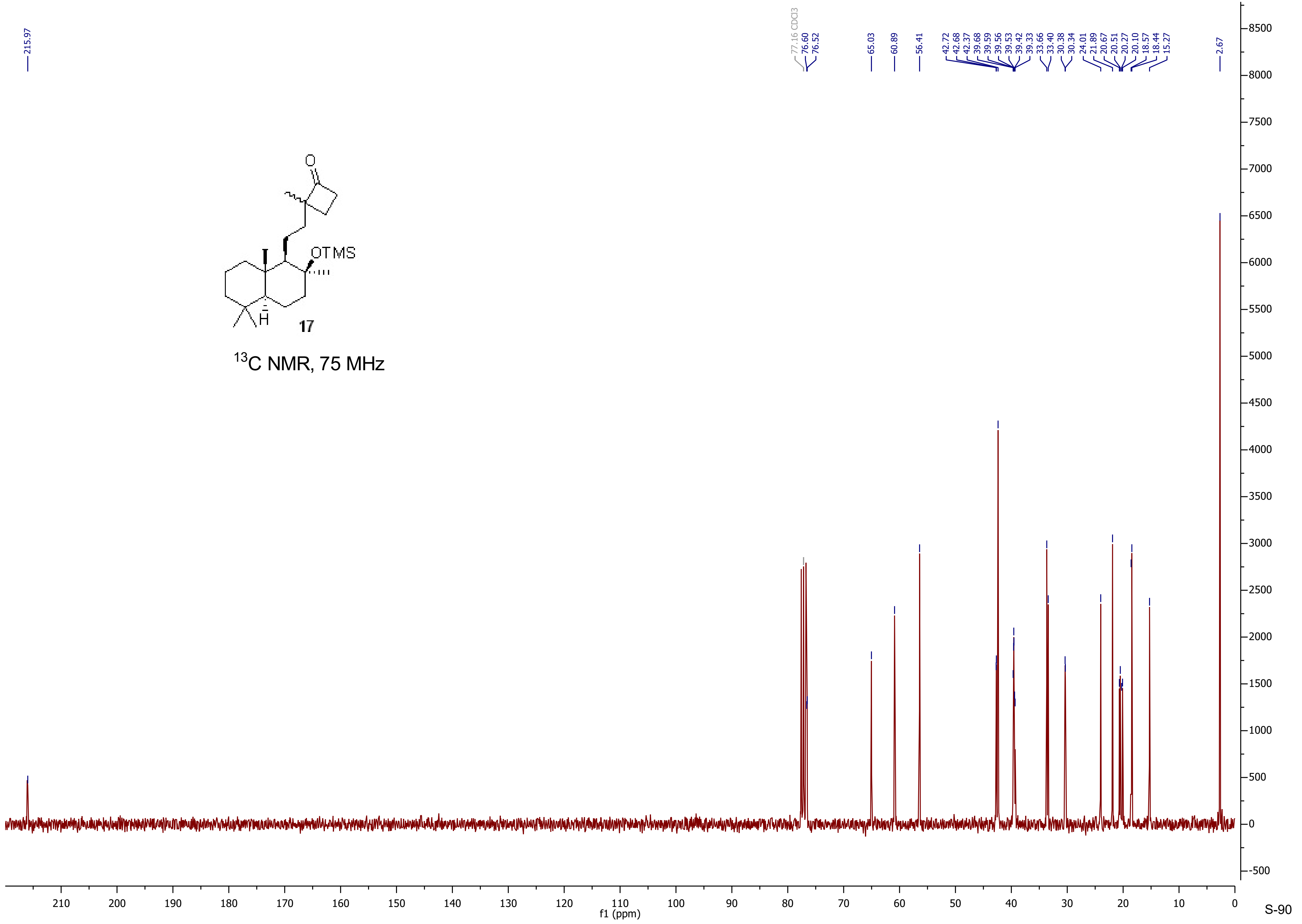
215.97
77.16 CDCl3
76.60
76.52
65.03
60.89
56.41
42.72
42.68
42.37
39.68
39.59
39.56
39.53
39.42
39.33
33.66
33.40
30.38
30.34
24.01
21.89
20.67
20.51
20.27
20.10
18.57
18.44
15.27
2.67
O
OTMS
H
17
^{13}C NMR, 75 MHz
f1 (ppm)

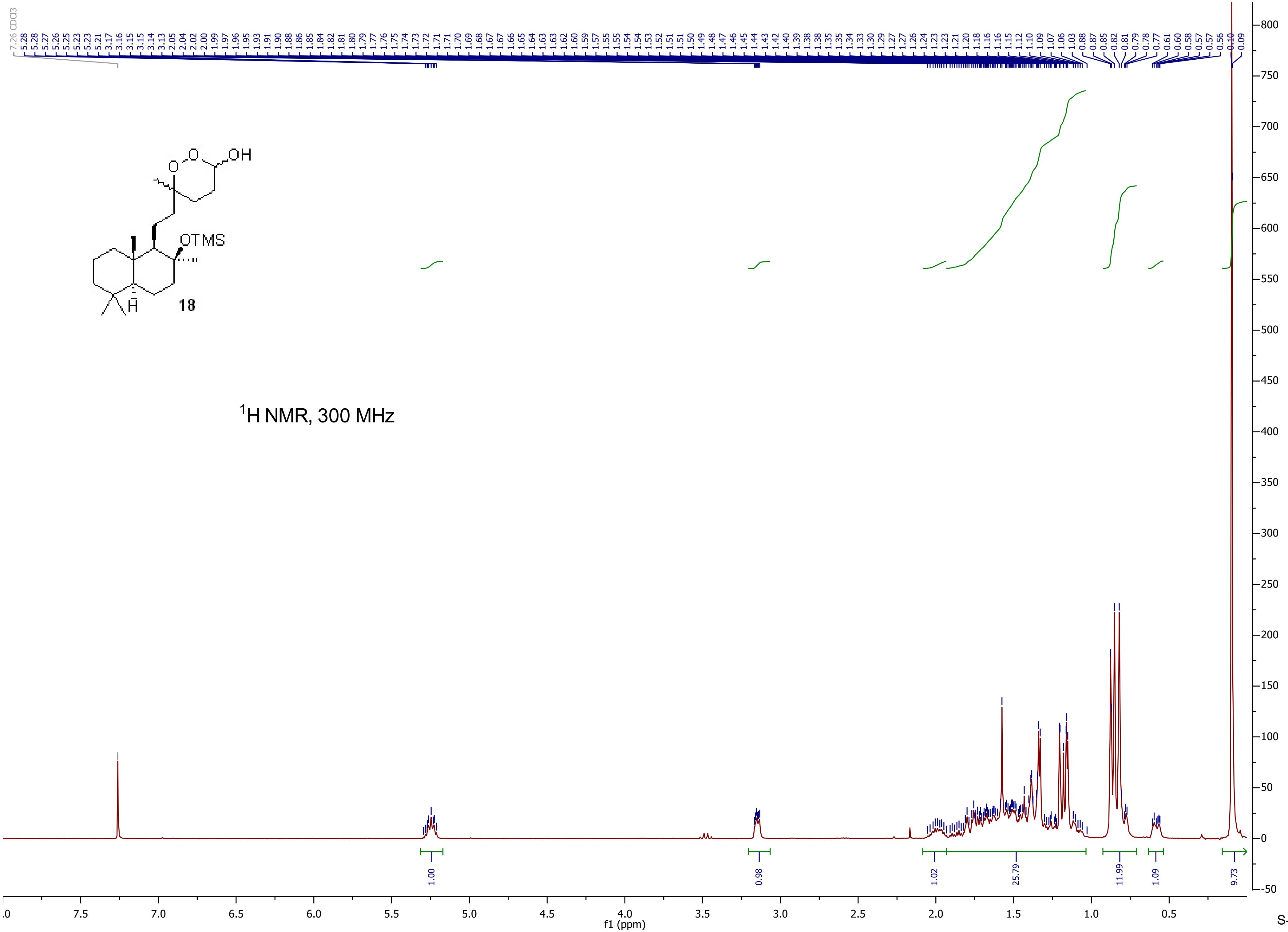

OH
OTMS
18
^{1}H NMR, 300 MHz
7.26 CDCl3
f1 (ppm)
1.00
0.98
1.02
25.79
11.99
1.09
9.73

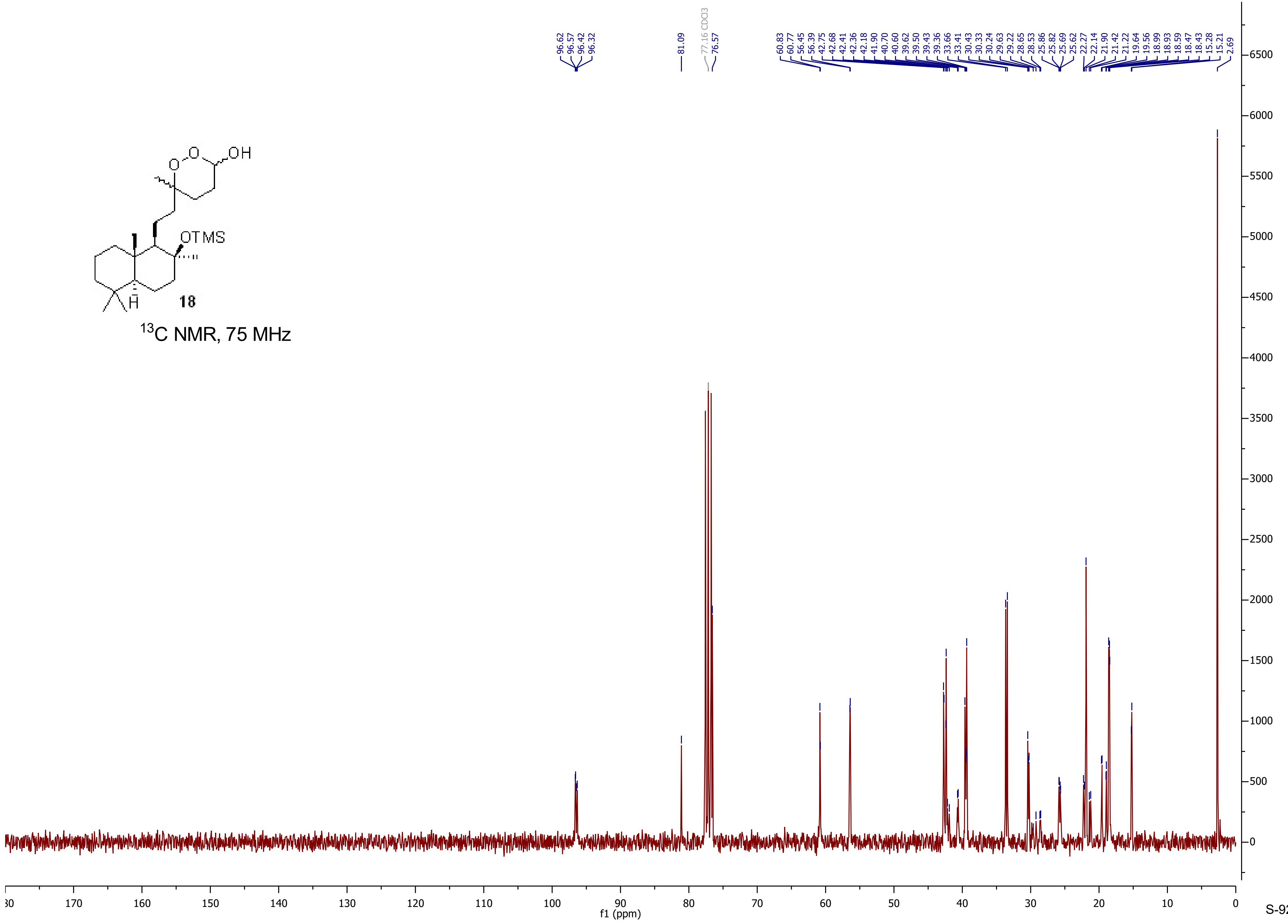
96.62
96.57
96.42
96.32
81.09
77.16 CDCl3
76.57
60.83
60.77
56.45
56.39
42.75
42.68
42.41
42.36
42.18
41.90
40.70
40.60
39.62
39.50
39.43
39.36
33.66
33.41
30.43
30.33
30.24
29.63
29.22
28.65
28.53
25.86
25.82
25.69
25.62
22.27
22.14
21.90
21.42
21.22
19.64
19.56
18.99
18.93
18.59
18.47
18.43
15.28
15.21
2.69
OH
O
O
OTMS
H
18
^{13}C NMR, 75 MHz
f1 (ppm)

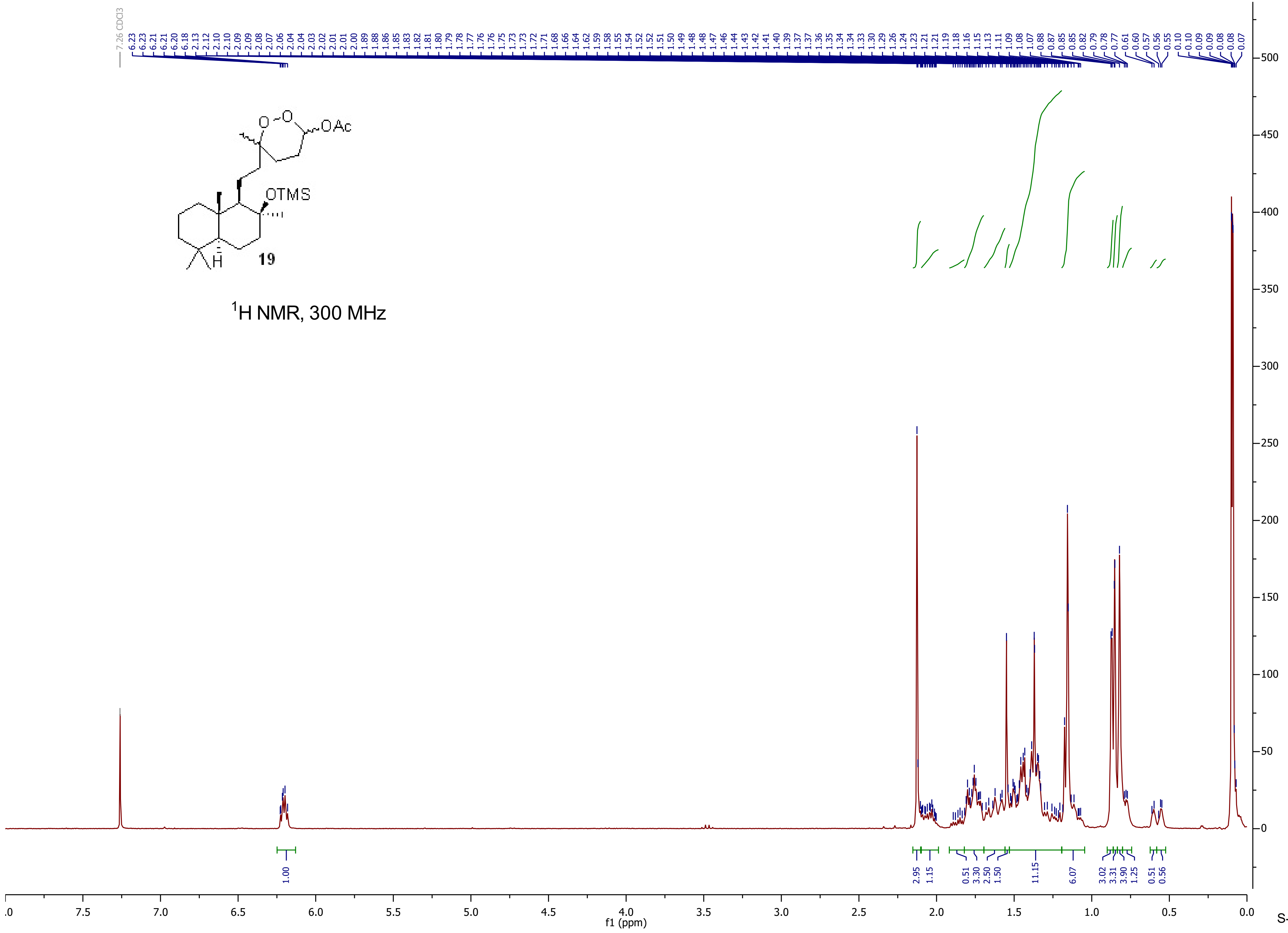

O
O
OAc
OTMS
H
19
1H NMR, 300 MHz
7.26 CDCl3
6.23 6.23 6.21 6.21 6.20 6.18 2.13 2.12 2.10 2.10 2.09 2.08 2.07 2.06 2.04 2.04 2.03 2.02 2.01 2.01 2.00 1.89 1.88 1.86 1.85 1.83 1.82 1.81 1.80 1.79 1.78 1.77 1.76 1.75 1.73 1.73 1.72 1.71 1.68 1.66 1.64 1.62 1.59 1.58 1.55 1.54 1.52 1.52 1.51 1.50 1.49 1.48 1.47 1.46 1.44 1.43 1.42 1.41 1.40 1.39 1.37 1.37 1.36 1.35 1.34 1.34 1.33 1.30 1.29 1.26 1.24 1.23 1.21 1.21 1.19 1.18 1.16 1.15 1.13 1.11 1.09 1.08 1.07 0.88 0.87 0.85 0.85 0.82 0.79 0.78 0.77 0.61 0.60 0.57 0.56 0.55 0.10 0.10 0.09 0.09 0.08 0.08 0.07
1.00
2.95 1.15
0.51 3.30 2.50 1.50
11.15
6.07
3.02 3.31 3.90 1.25
0.51 0.56
f1 (ppm)

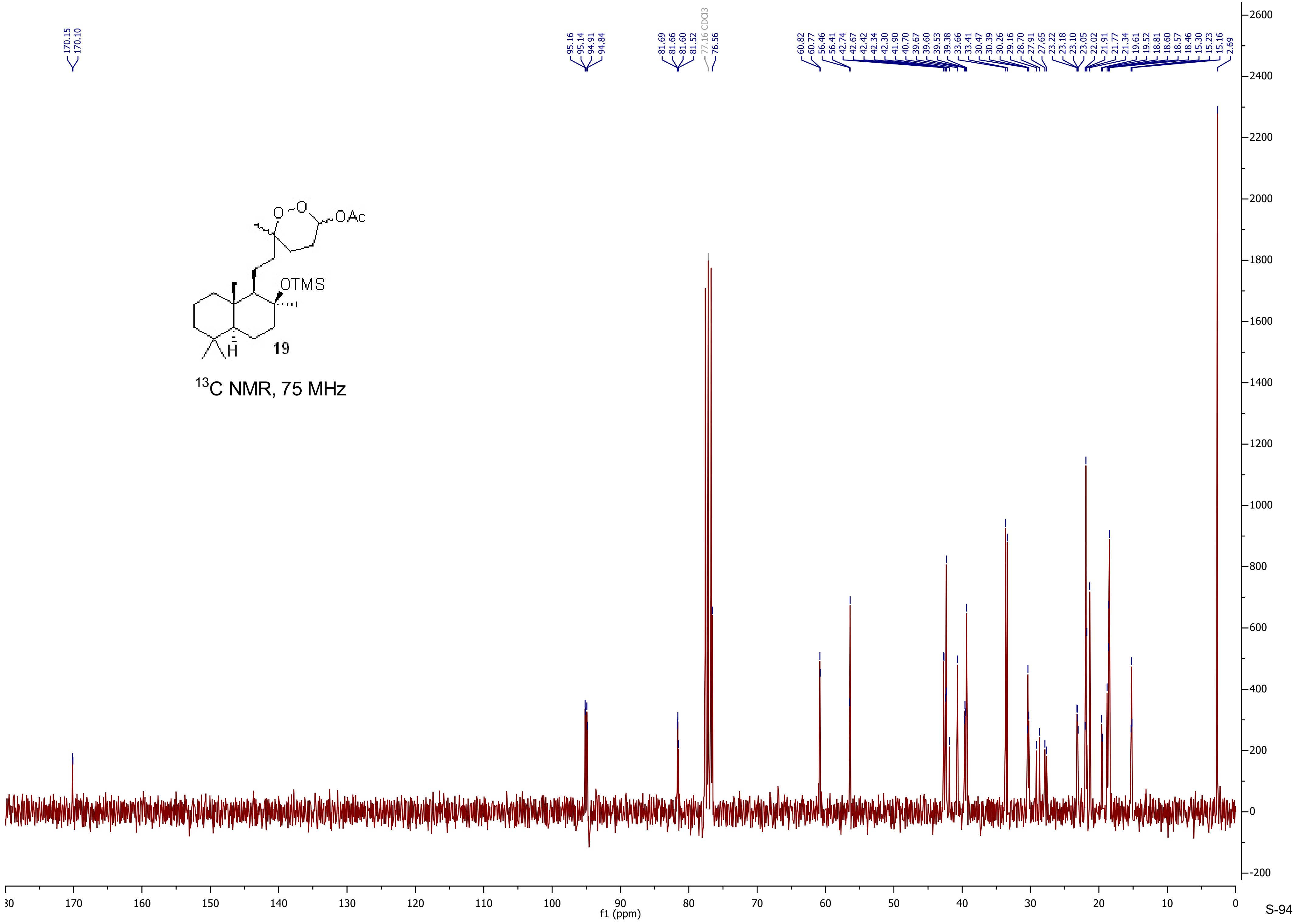
170.15
170.10
95.16
95.14
94.91
94.84
81.69
81.66
81.60
81.52
77.16 CDCl3
76.56
60.82
60.77
56.46
56.41
42.74
42.67
42.42
42.34
42.30
41.90
40.70
39.67
39.60
39.53
39.38
33.66
33.41
30.47
30.39
30.26
29.16
28.70
27.91
27.65
23.22
23.18
23.10
23.05
22.02
21.91
21.77
21.34
19.61
19.52
18.81
18.60
18.57
18.46
15.30
15.23
15.16
2.69
O
O
OAc
OTMS
H
19
^{13}C NMR, 75 MHz
f1 (ppm)

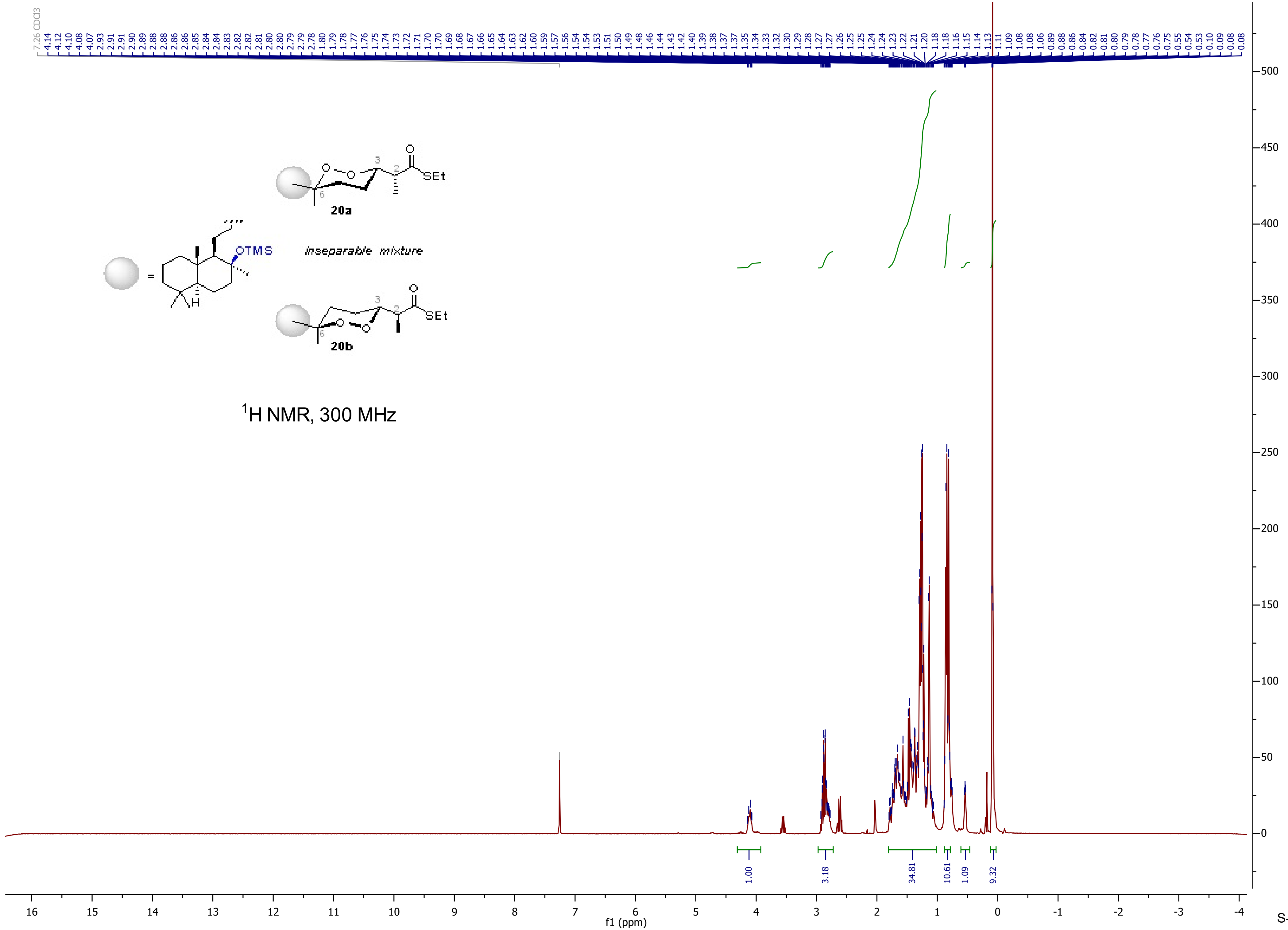
20a
inseparable mixture
20b
OTMS
SEt
^{1}H NMR, 300 MHz
f1 (ppm)

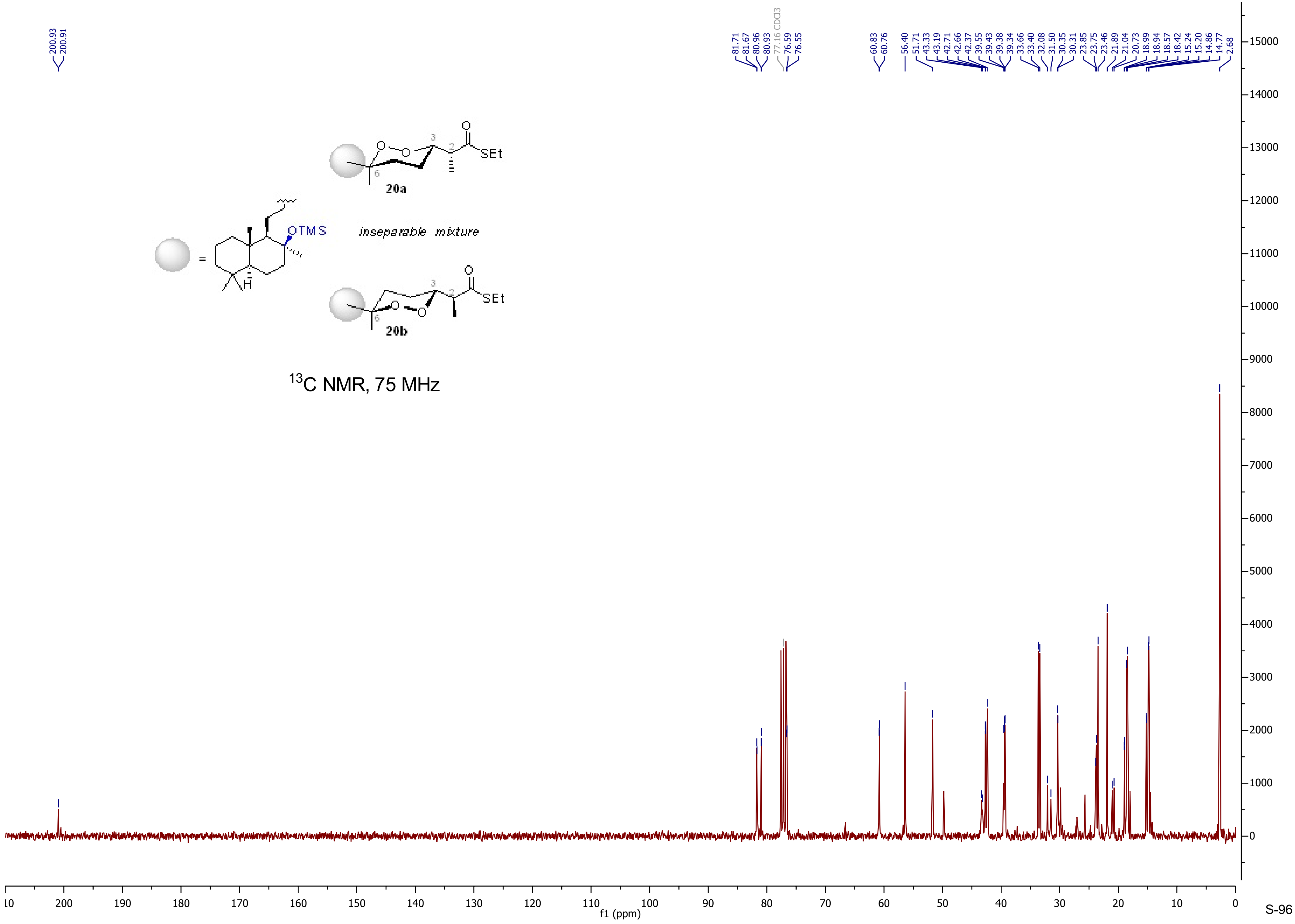
200.93
200.91
81.71
81.67
80.96
80.93
77.16 CDCl3
76.59
76.55
60.83
60.76
56.40
51.71
43.33
43.19
42.71
42.66
42.37
39.55
39.43
39.38
39.34
33.66
33.40
32.08
31.50
30.35
30.31
23.85
23.75
23.46
21.89
21.04
20.73
18.99
18.94
18.57
18.42
15.24
15.20
14.86
14.77
2.68
SEt
20a
OTMS
inseparable mixture
20b
13C NMR, 75 MHz
f1 (ppm)

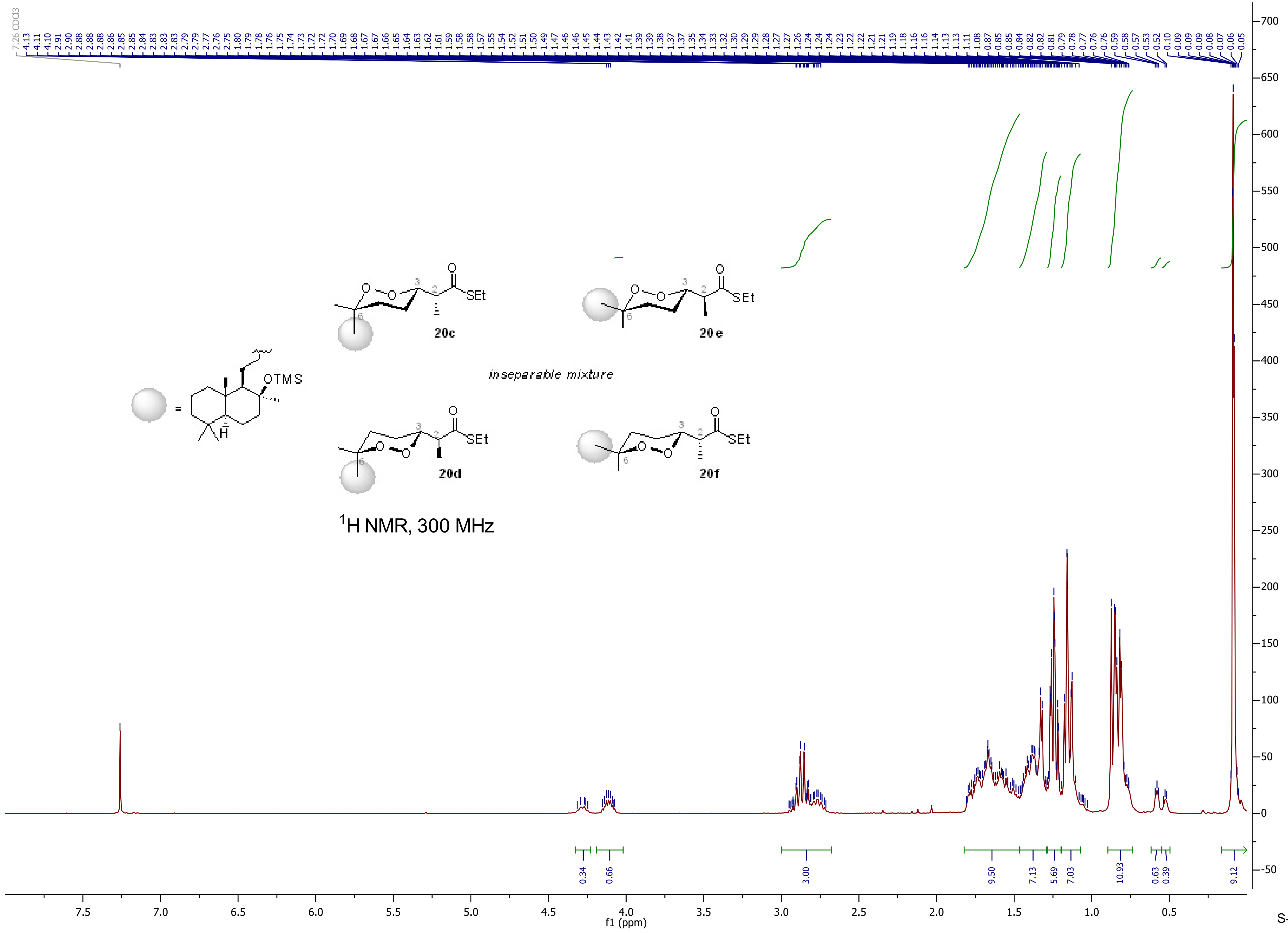
20c
20e
20d
20f
OTMS
SEt
inseparable mixture
1H NMR, 300 MHz
f1 (ppm)

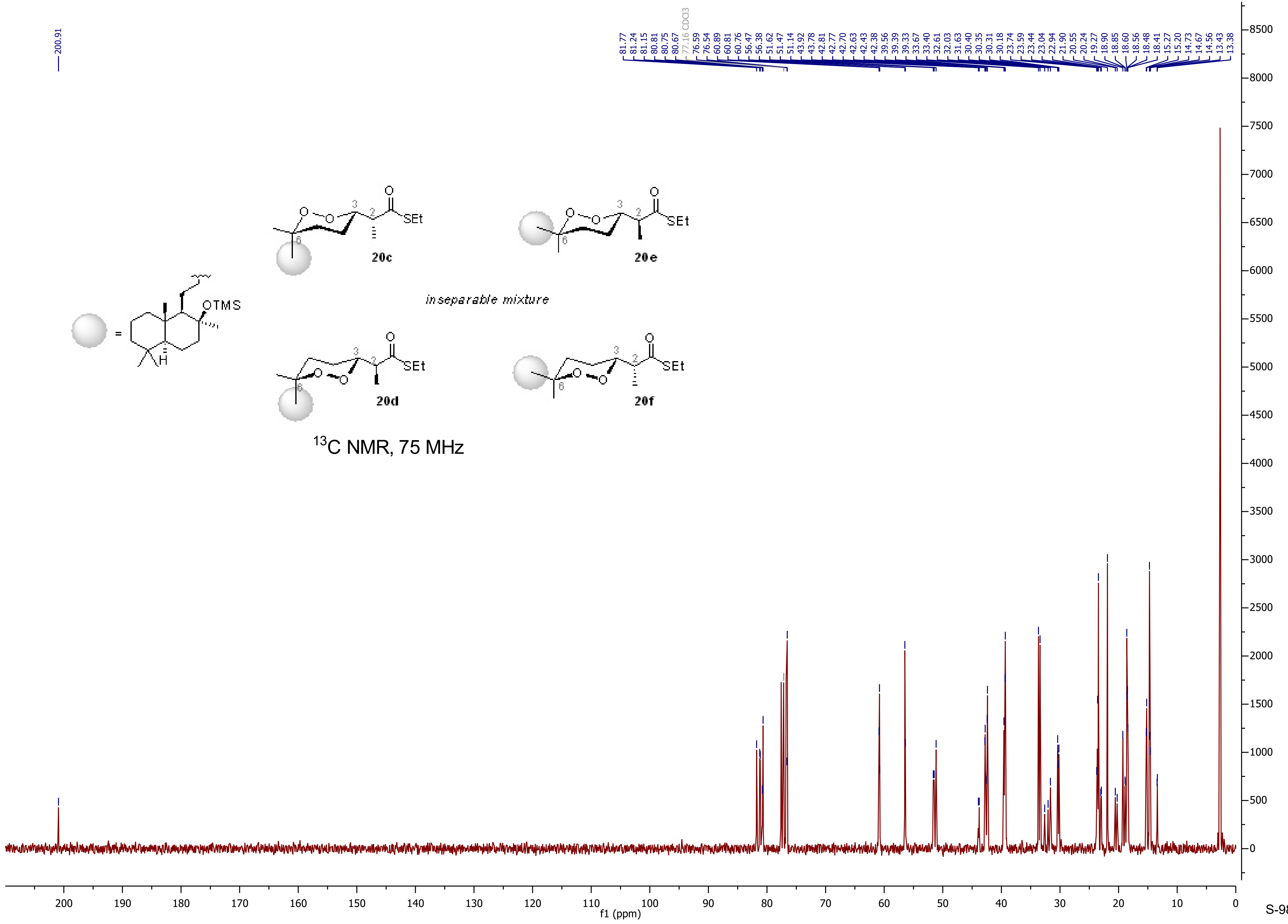

200.91
81.77 81.24 81.15 80.81 80.75 80.67 77.16 CDCl3 76.59 76.54 60.89 60.81 60.76 56.47 56.38 51.62 51.47 51.14 43.92 43.78 42.81 42.77 42.70 42.63 42.43 42.38 39.56 39.39 39.33 33.67 33.40 32.61 32.03 31.63 30.40 30.35 30.31 30.18 23.74 23.59 23.44 23.04 22.94 21.90 20.55 20.24 19.27 18.90 18.85 18.60 18.56 18.48 18.41 15.27 15.20 14.73 14.67 14.56 13.43 13.38
OTMS
H
SEt
20c
20e
inseparable mixture
20d
20f
^{13}C NMR, 75 MHz
f1 (ppm)

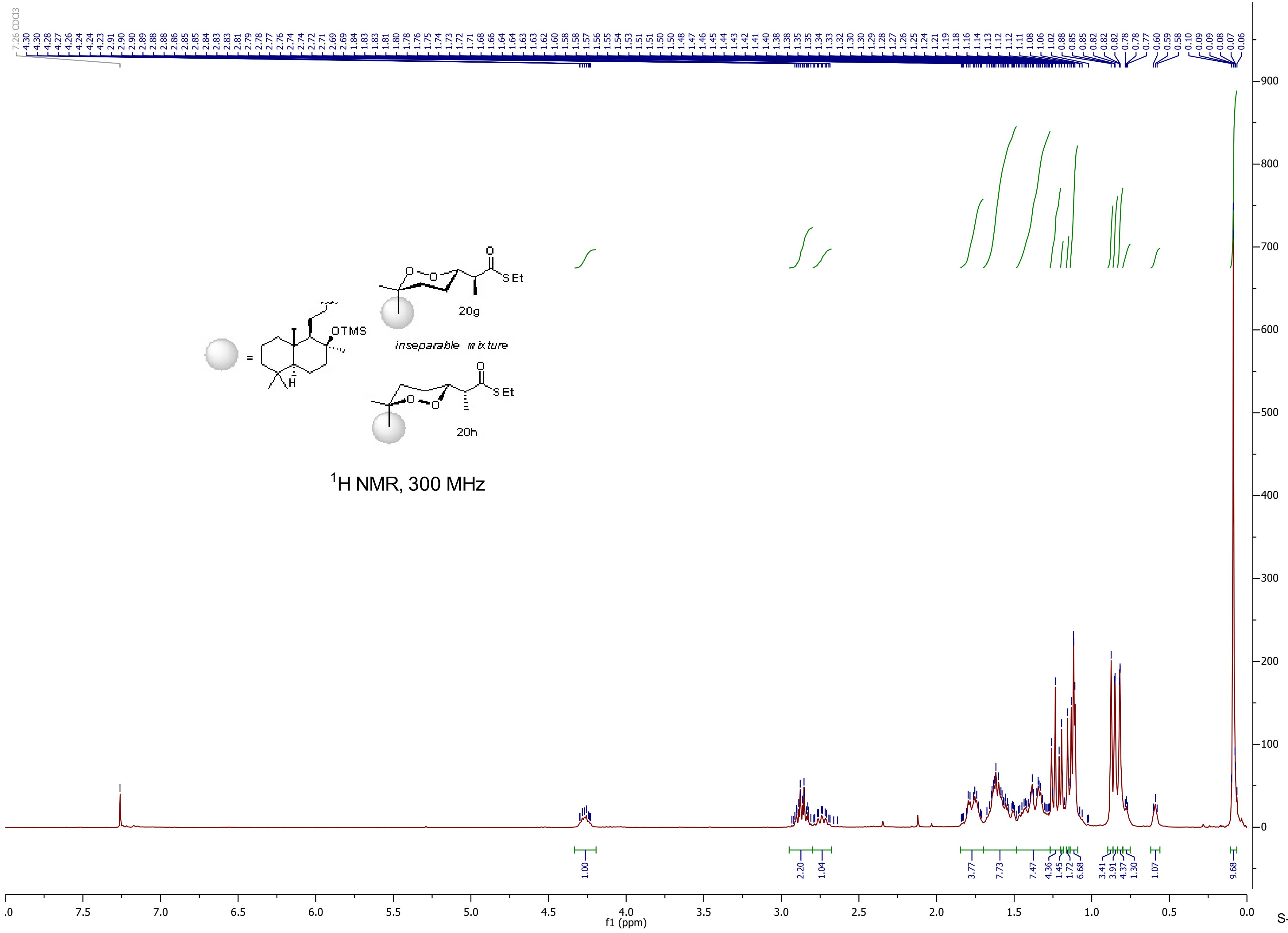
20g
20h
inseparable mixture
OTMS
SEt
=
H
O
¹H NMR, 300 MHz
f1 (ppm)
7.26 CDCl3
1.00
2.20
1.04
3.77
7.73
7.47
4.36
1.45
1.72
6.68
3.41
3.91
4.37
1.30
1.07
9.68

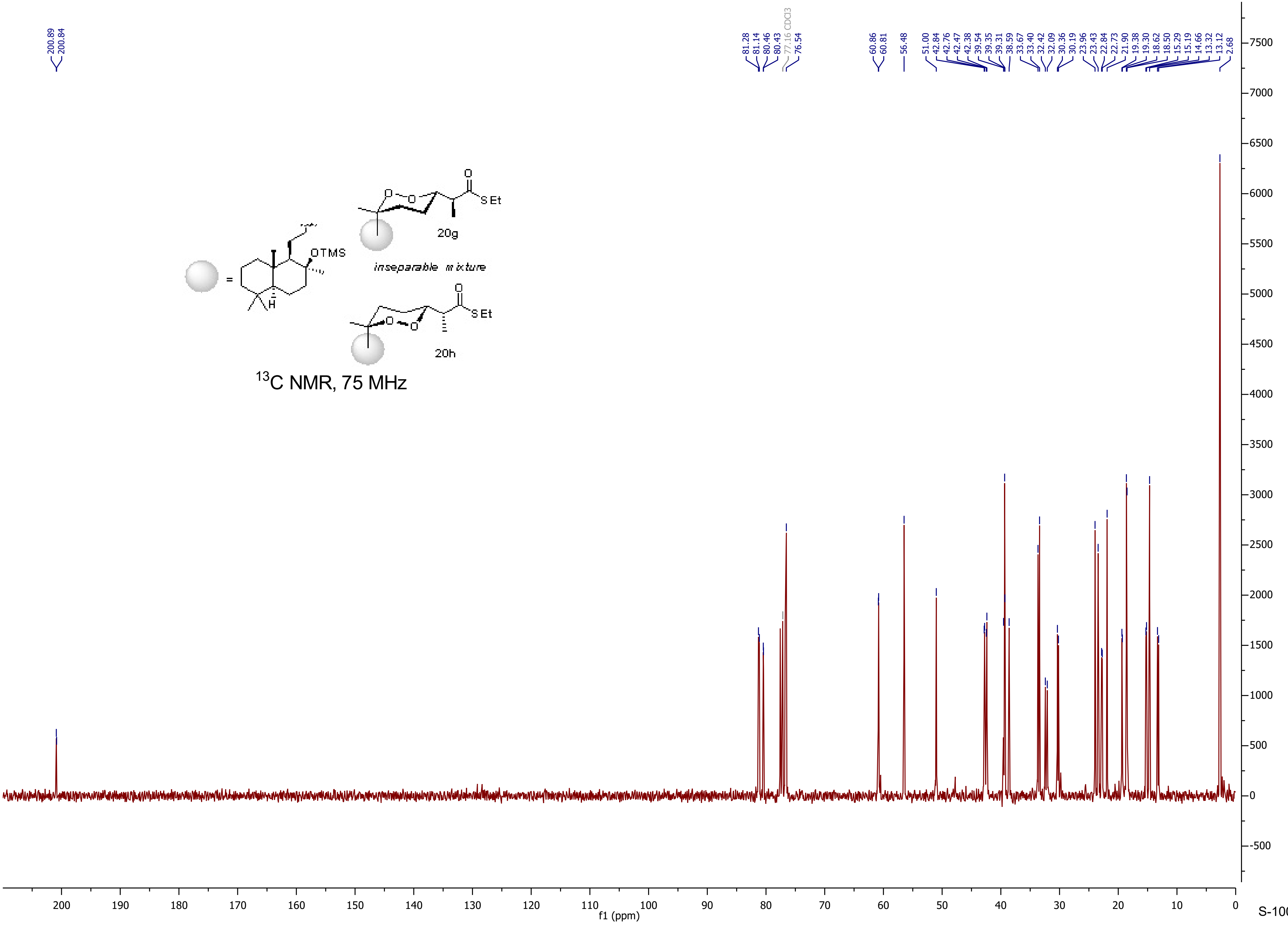
200.89
200.84
81.28
81.14
80.46
80.43
77.16 CDCl3
76.54
60.86
60.81
56.48
51.00
42.84
42.76
42.47
42.38
39.54
39.35
39.31
38.59
33.67
33.40
32.42
32.09
30.36
30.19
23.96
23.43
22.84
22.73
21.90
19.38
19.30
18.62
18.50
15.29
15.19
14.66
13.32
13.12
2.68
OTMS
SEt
20g
inseparable mixture
20h
13C NMR, 75 MHz
f1 (ppm)

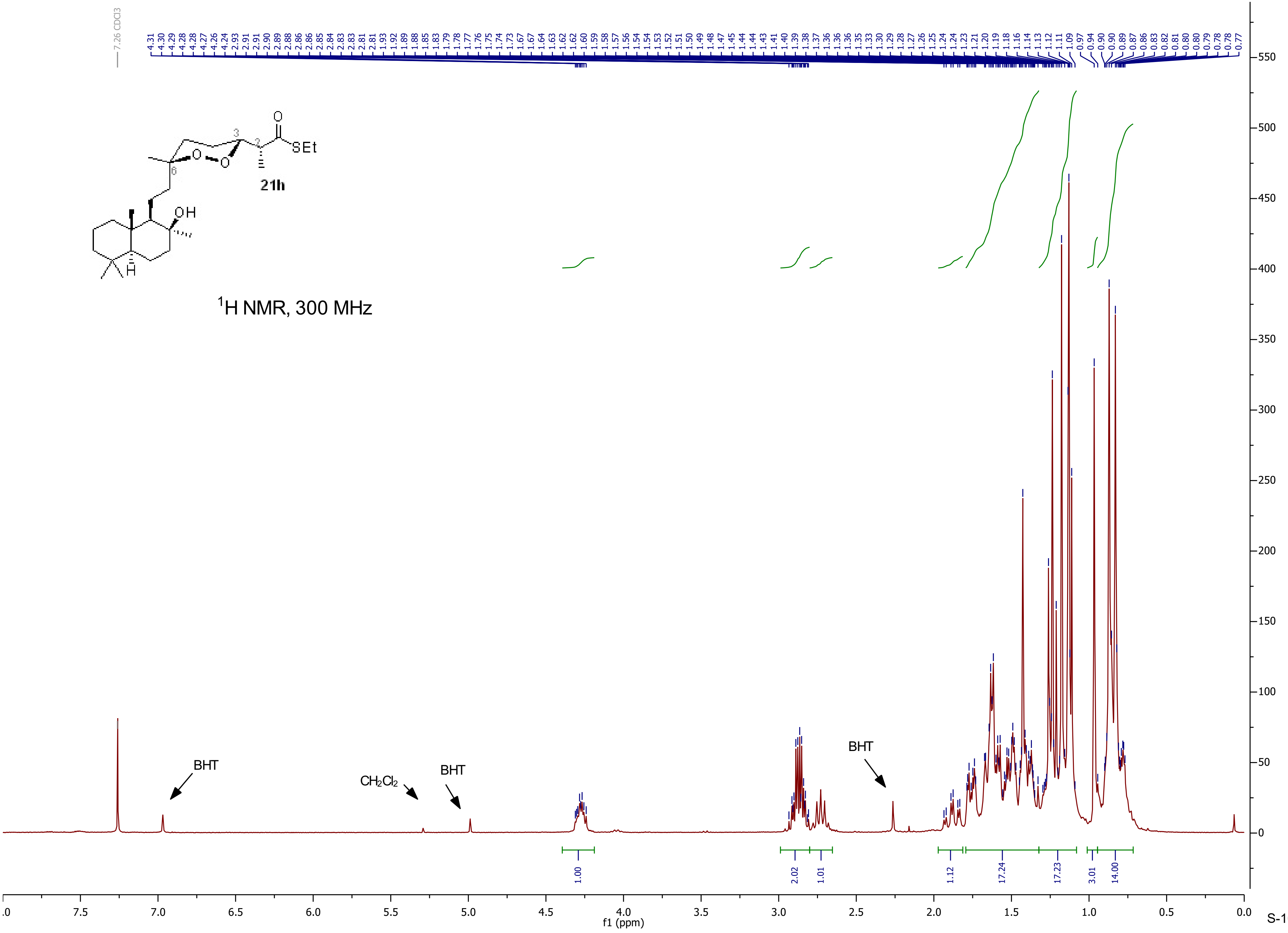
7.26 CDCl3
4.31 4.30 4.29 4.28 4.28 4.27 4.26 4.24 2.93 2.91 2.91 2.90 2.89 2.88 2.86 2.86 2.85 2.84 2.83 2.83 2.81 2.81 1.93 1.92 1.89 1.88 1.85 1.83 1.79 1.78 1.77 1.76 1.75 1.74 1.73 1.67 1.64 1.63 1.62 1.62 1.60 1.59 1.58 1.57 1.56 1.54 1.54 1.53 1.52 1.51 1.50 1.49 1.48 1.47 1.45 1.44 1.43 1.41 1.40 1.39 1.38 1.37 1.36 1.36 1.35 1.33 1.30 1.29 1.28 1.27 1.26 1.25 1.24 1.24 1.23 1.21 1.20 1.19 1.18 1.16 1.14 1.13 1.12 1.11 1.09 0.97 0.94 0.90 0.90 0.89 0.87 0.86 0.83 0.82 0.81 0.80 0.80 0.79 0.78 0.78 0.77
SEt
OH
21h
1H NMR, 300 MHz
BHT
CH2Cl2
BHT
BHT
1.00
2.02
1.01
1.12
17.24
17.23
3.01
14.00
f1 (ppm)

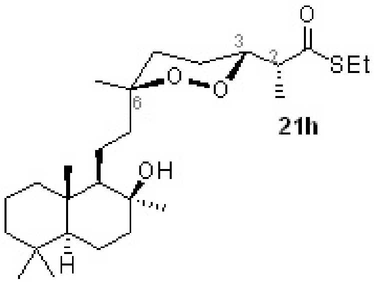

21h

^{13}C NMR, 75 MHz

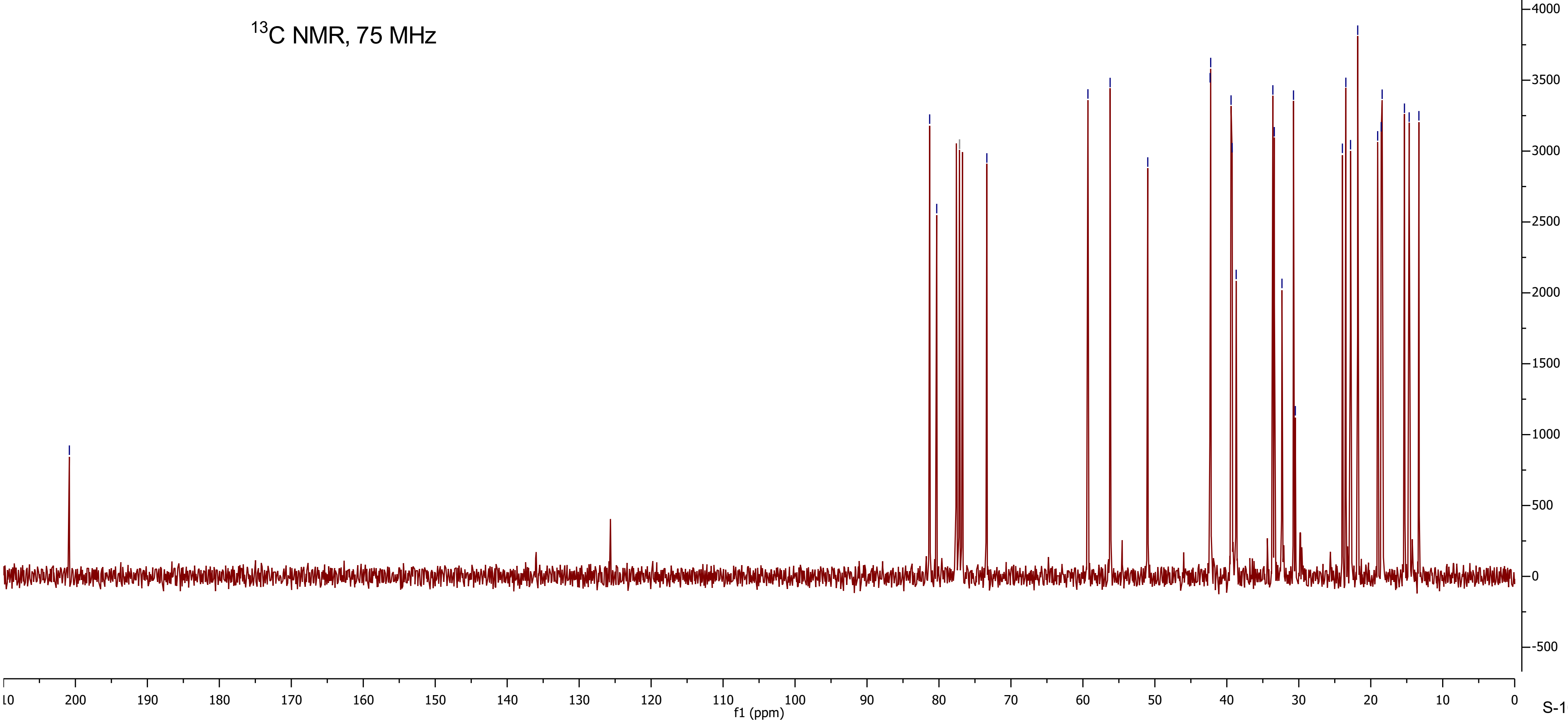

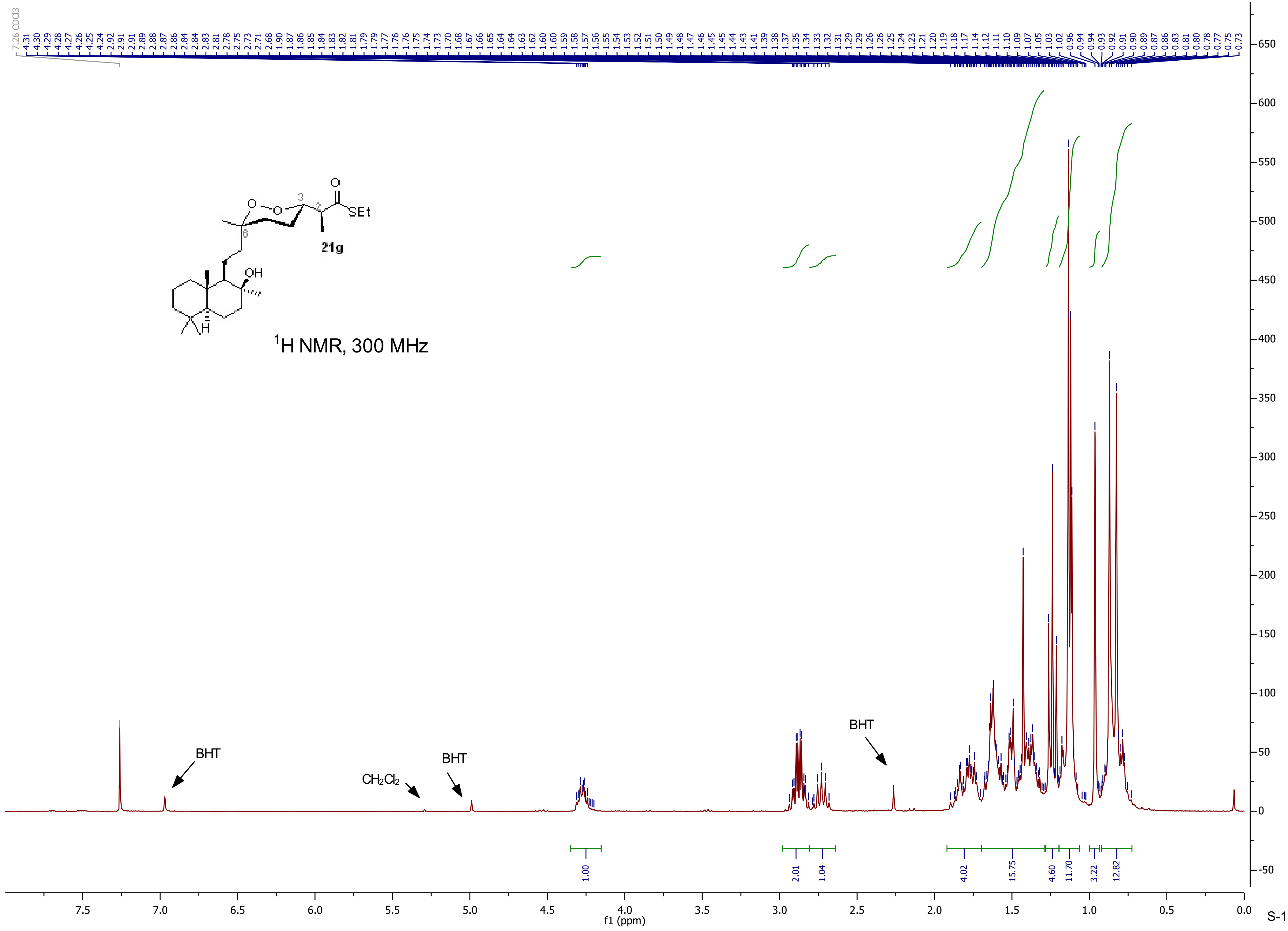
21g
SEt
OH
1H NMR, 300 MHz
BHT
CH2Cl2
BHT
BHT
f1 (ppm)

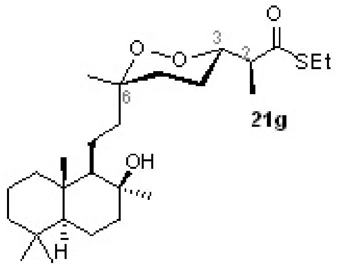

21g

^{13}C NMR, 75 MHz

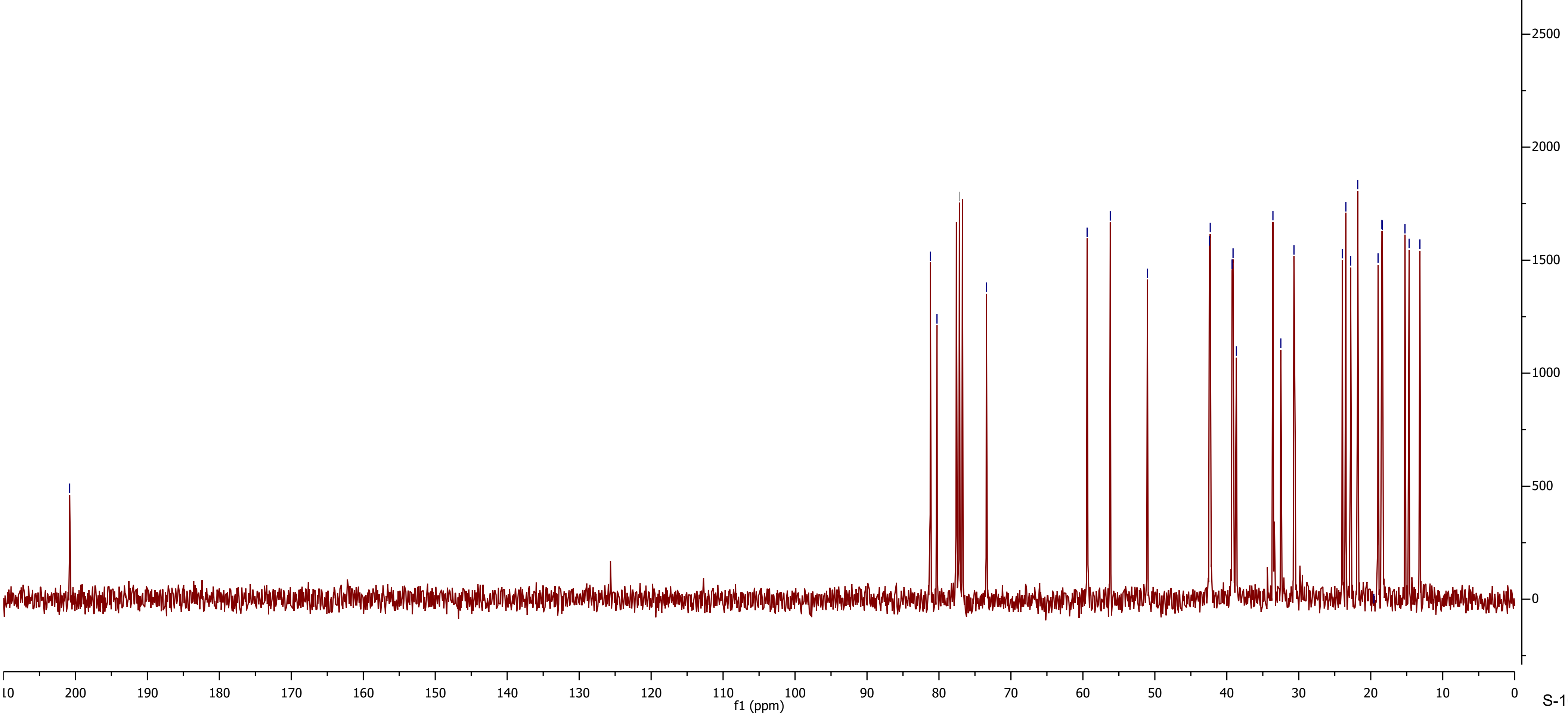

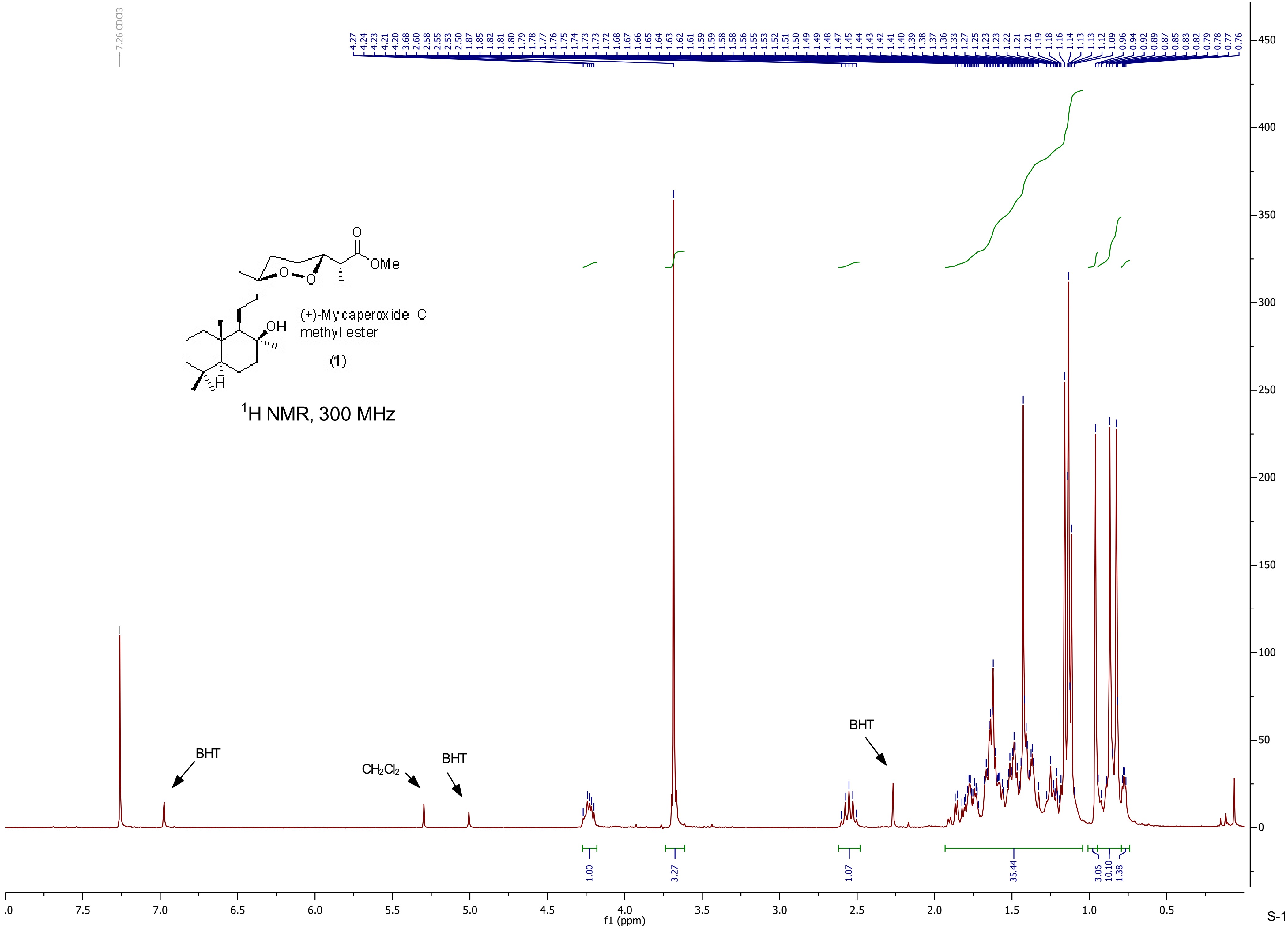
7.26 CDCl3
OMe
O
OH
H
(+)-Mycaperoxide C methyl ester
(1)
1H NMR, 300 MHz
BHT
CH2Cl2
BHT
BHT
1.00
3.27
1.07
35.44
3.06
10.10
1.38
f1 (ppm)

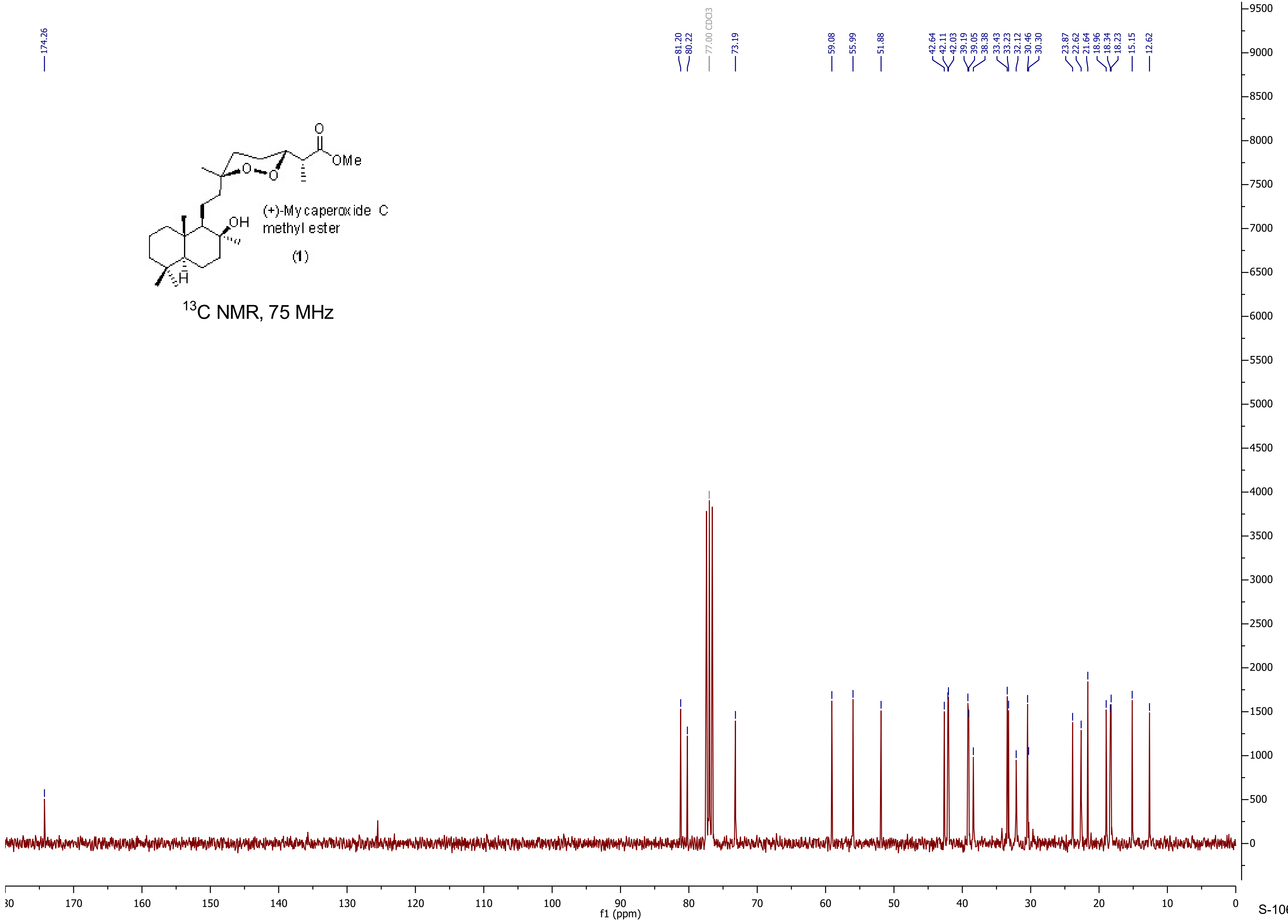
174.26
81.20
80.22
77.00 CDCl3
73.19
59.08
55.99
51.88
42.64
42.11
42.03
39.19
39.05
38.38
33.43
33.23
32.12
30.46
30.30
23.87
22.62
21.64
18.96
18.34
18.23
15.15
12.62
O
OMe
O
O
OH
H
(+)-Mycaperoxide C methyl ester
(1)
^{13}C NMR, 75 MHz
f1 (ppm)
9500
9000
8500
8000
7500
7000
6500
6000
5500
5000
4500
4000
3500
3000
2500
2000
1500
1000
500
0
170
160
150
140
130
120
110
100
90
80
70
60
50
40
30
20
10
0

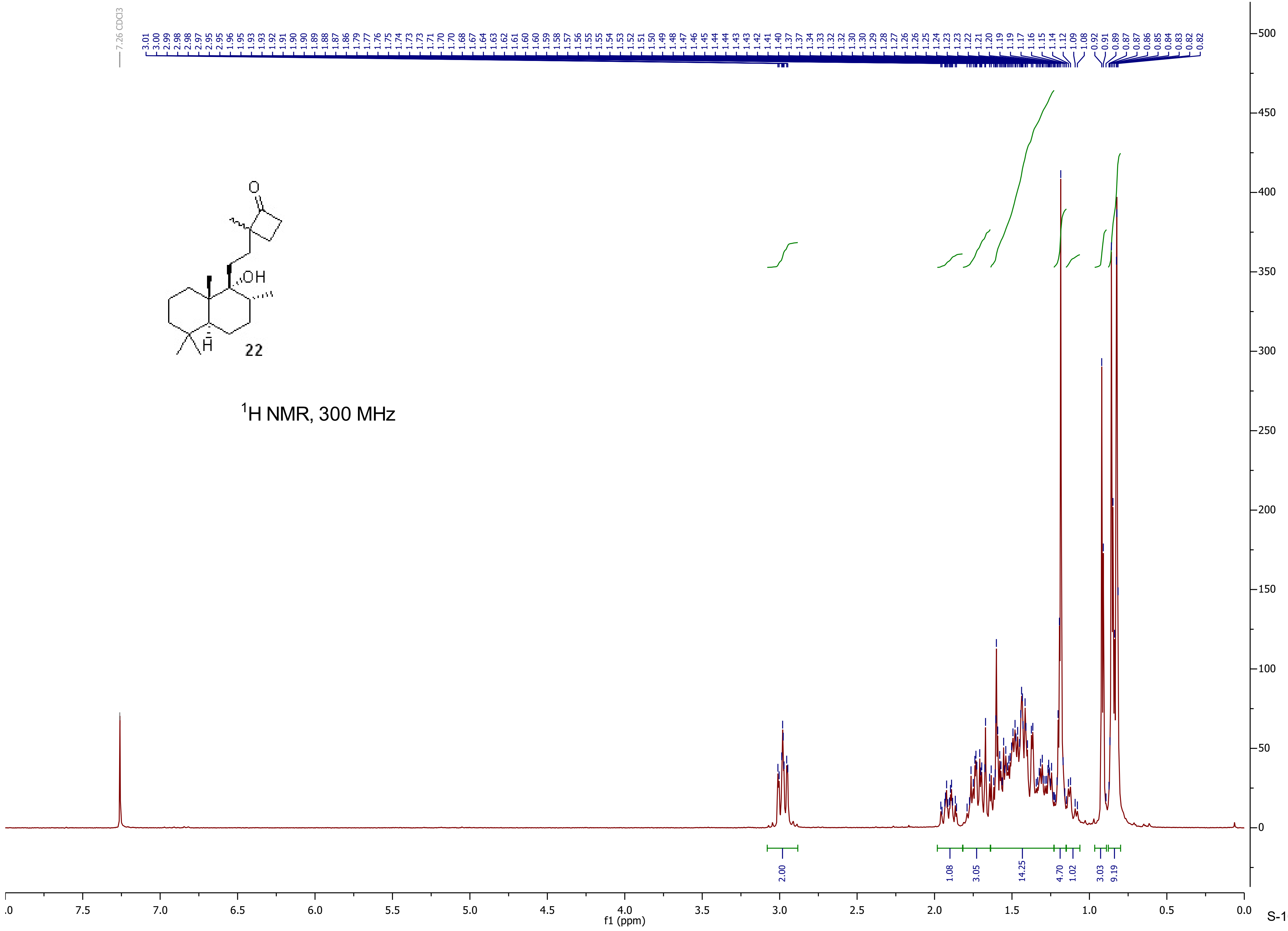
7.26 CDCl3
3.01 3.00 2.99 2.98 2.97 2.95 2.95 1.96 1.95 1.93 1.93 1.92 1.91 1.90 1.90 1.89 1.88 1.87 1.86 1.79 1.77 1.76 1.75 1.74 1.73 1.73 1.71 1.70 1.70 1.68 1.67 1.64 1.63 1.62 1.61 1.60 1.60 1.59 1.58 1.57 1.56 1.55 1.55 1.54 1.53 1.52 1.51 1.50 1.49 1.48 1.47 1.46 1.45 1.44 1.44 1.43 1.43 1.42 1.41 1.40 1.37 1.37 1.34 1.33 1.32 1.32 1.30 1.30 1.29 1.28 1.27 1.26 1.26 1.25 1.24 1.23 1.23 1.23 1.22 1.21 1.20 1.19 1.19 1.17 1.16 1.15 1.14 1.12 1.09 1.08 0.92 0.91 0.89 0.87 0.86 0.85 0.84 0.83 0.82 0.82
OH
22
1H NMR, 300 MHz
2.00
1.08
3.05
14.25
4.70
1.02
3.03
9.19
f1 (ppm)

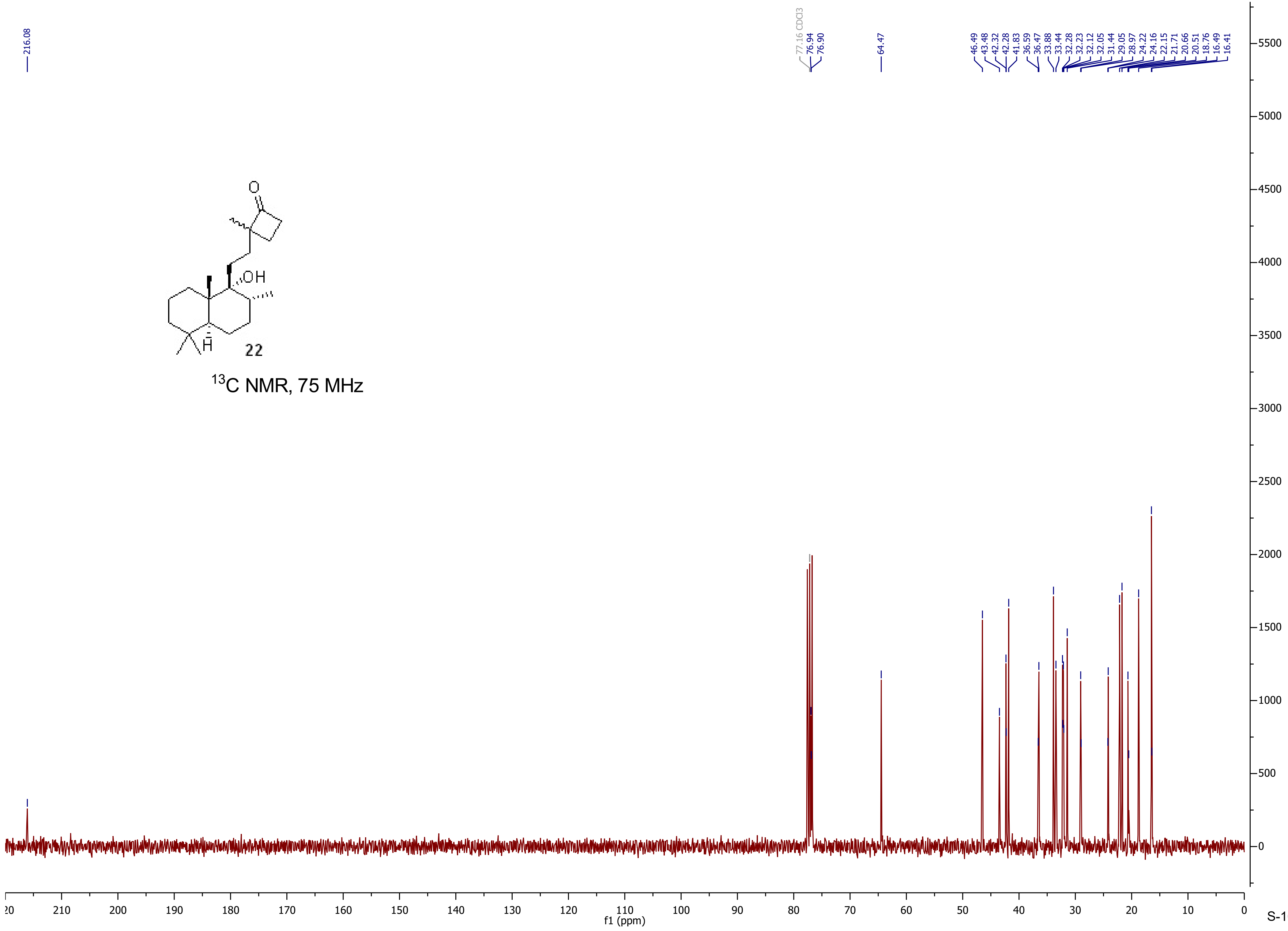

216.08
77.16 CDCl3
76.94
76.90
64.47
46.49
43.48
42.32
42.28
41.83
36.59
36.47
33.88
33.44
32.28
32.23
32.12
32.05
31.44
29.05
28.97
24.22
24.16
22.15
21.71
20.66
20.51
18.76
16.49
16.41
OH
H
22
13C NMR, 75 MHz
f1 (ppm)

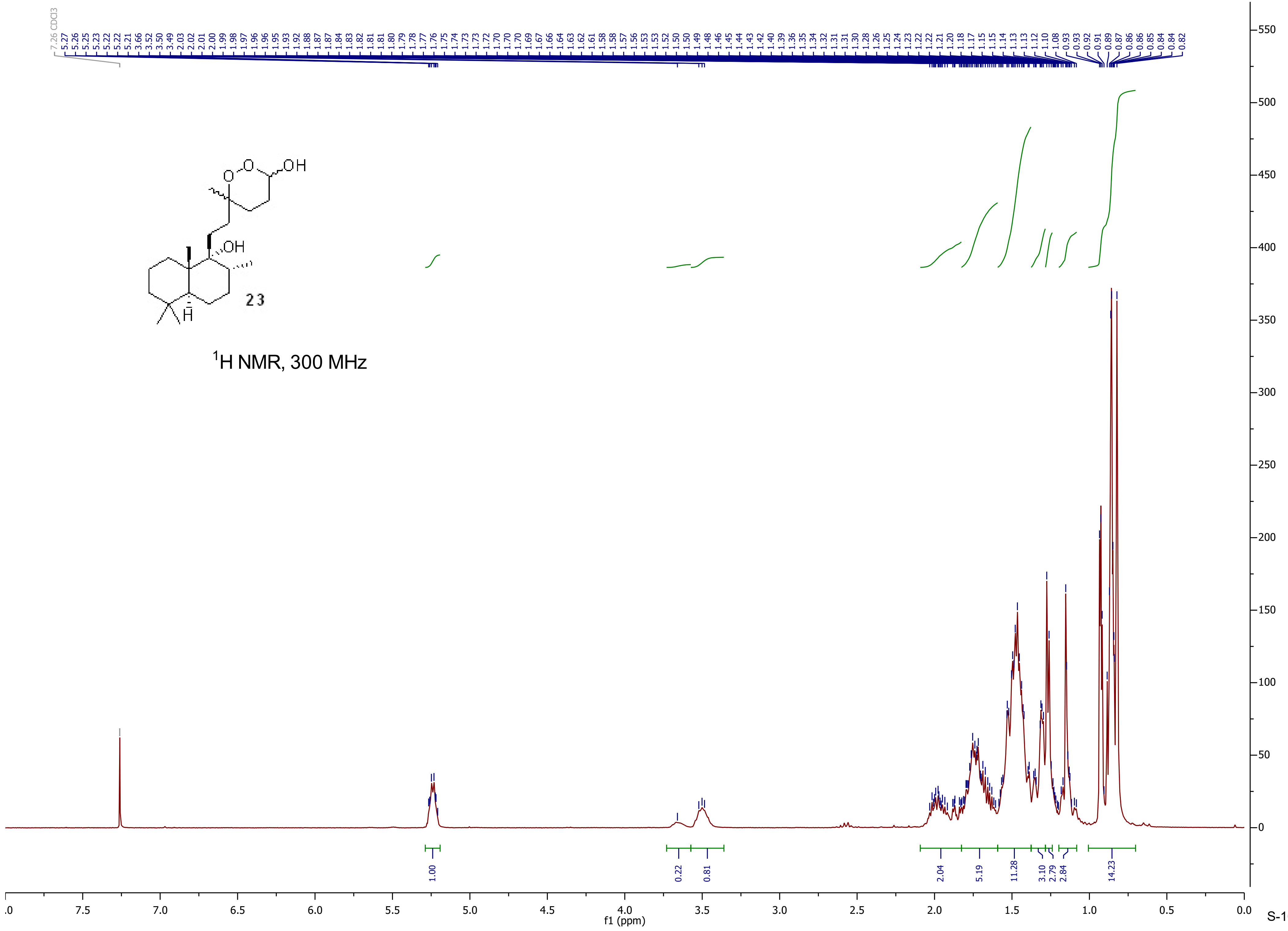

7.26 CDCl3
5.27 5.26 5.25 5.23 5.22 5.22 5.21 3.66 3.52 3.50 3.49 2.03 2.02 2.01 2.00 1.99 1.98 1.97 1.96 1.96 1.95 1.93 1.92 1.88 1.87 1.87 1.84 1.83 1.82 1.81 1.81 1.80 1.79 1.78 1.77 1.76 1.75 1.74 1.73 1.73 1.72 1.70 1.70 1.69 1.67 1.66 1.64 1.63 1.62 1.61 1.58 1.58 1.57 1.56 1.53 1.53 1.52 1.50 1.50 1.49 1.48 1.46 1.45 1.44 1.43 1.42 1.40 1.39 1.36 1.35 1.34 1.32 1.31 1.31 1.30 1.28 1.26 1.25 1.24 1.23 1.22 1.22 1.21 1.20 1.18 1.17 1.15 1.15 1.14 1.13 1.13 1.12 1.10 1.08 0.93 0.93 0.92 0.91 0.89 0.87 0.86 0.85 0.84 0.84 0.82
OH
O
O
OH
H
23
1H NMR, 300 MHz
1.00
0.22
0.81
2.04
5.19
11.28
3.10
2.79
2.84
14.23
f1 (ppm)

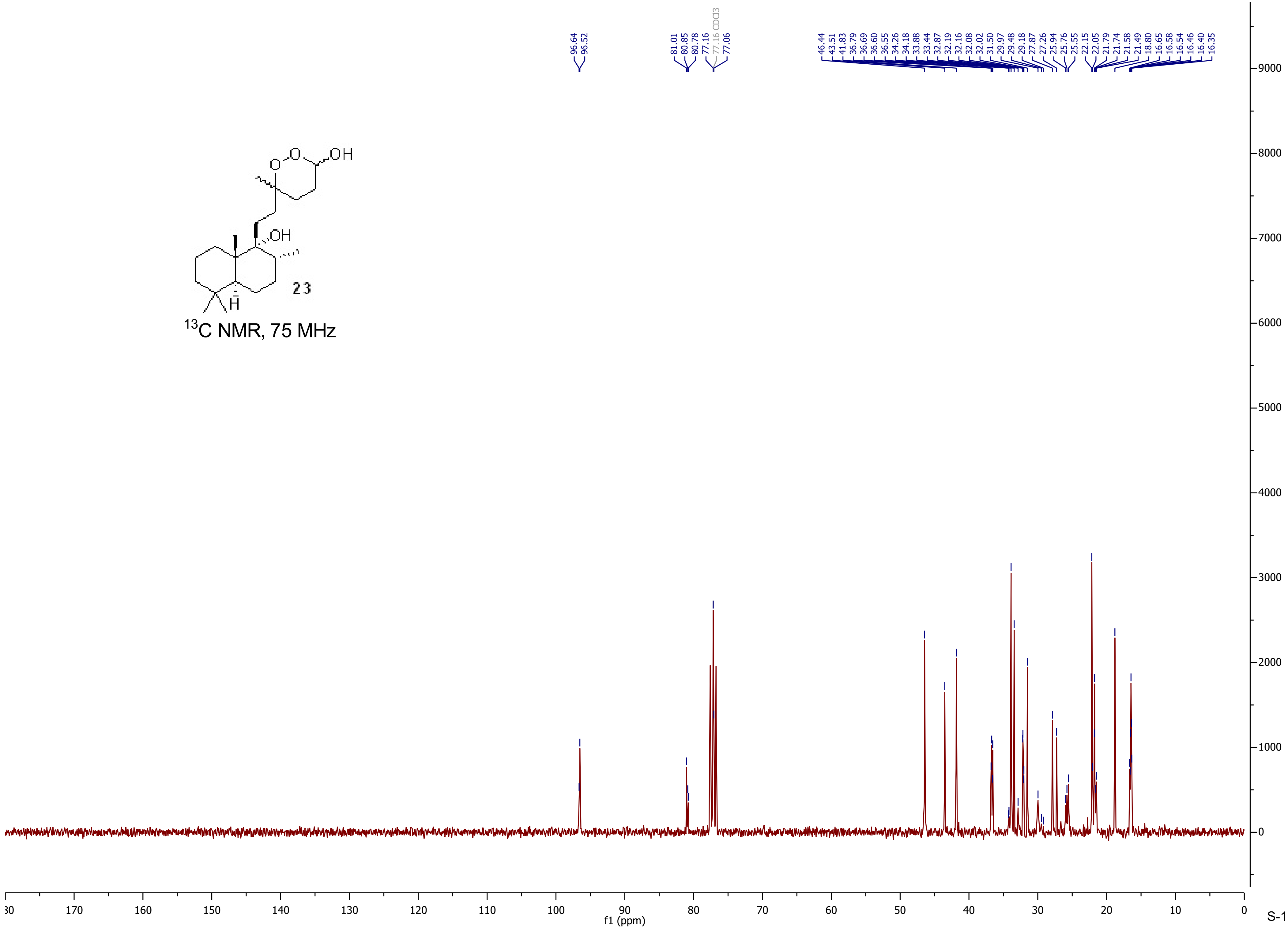

96.64
96.52
81.01
80.85
80.78
77.16
77.16 CDCl3
77.06
46.44
43.51
41.83
36.79
36.69
36.60
36.55
34.26
34.18
33.88
33.44
32.87
32.19
32.16
32.08
32.02
31.50
29.97
29.48
29.18
27.87
27.26
25.94
25.76
25.55
22.15
22.05
21.79
21.74
21.58
21.49
18.80
16.65
16.58
16.54
16.46
16.40
16.35
OH
O
O
OH
H
23
^{13}C NMR, 75 MHz
f1 (ppm)

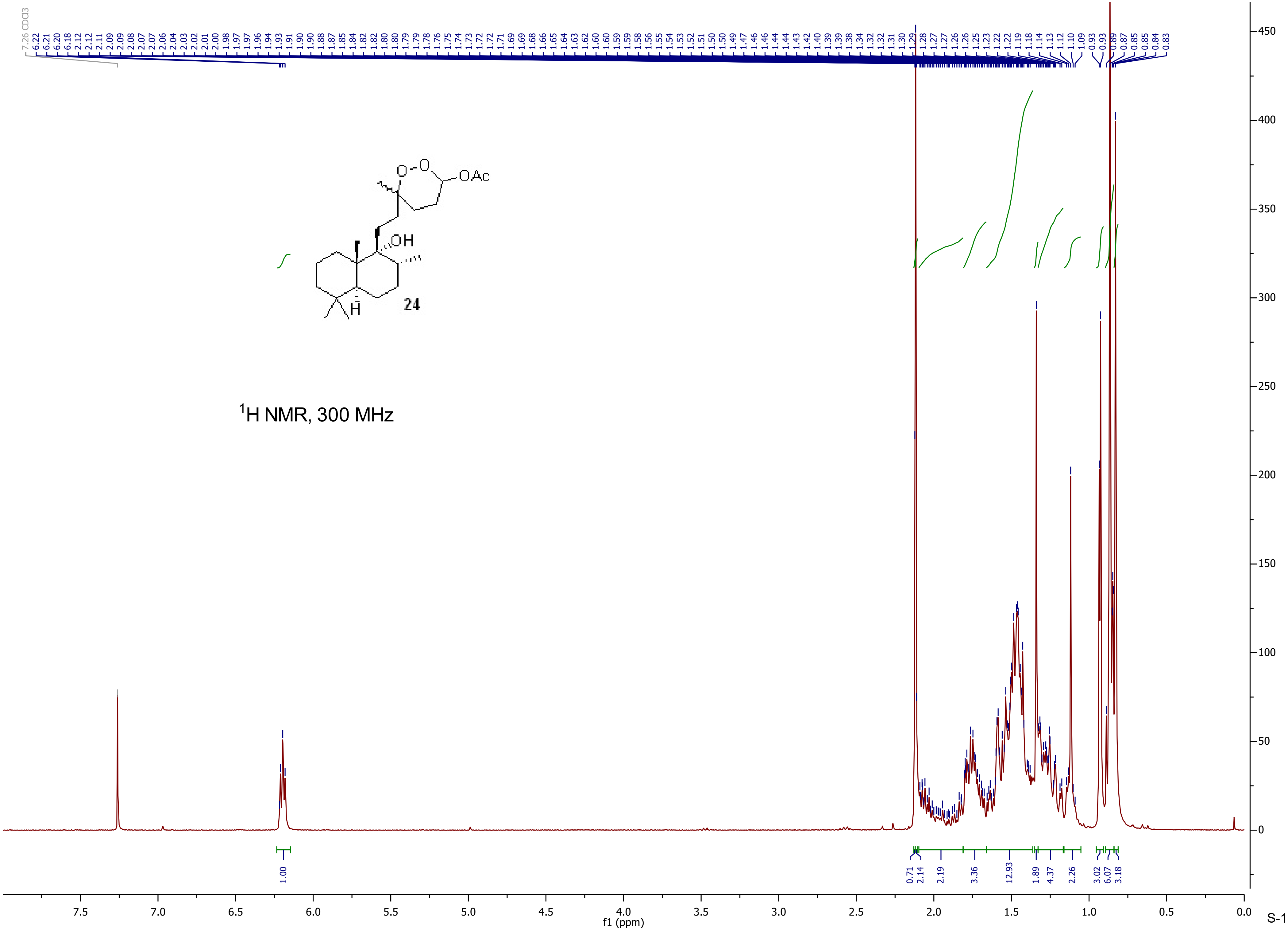

OAc
OH
24
1H NMR, 300 MHz
f1 (ppm)

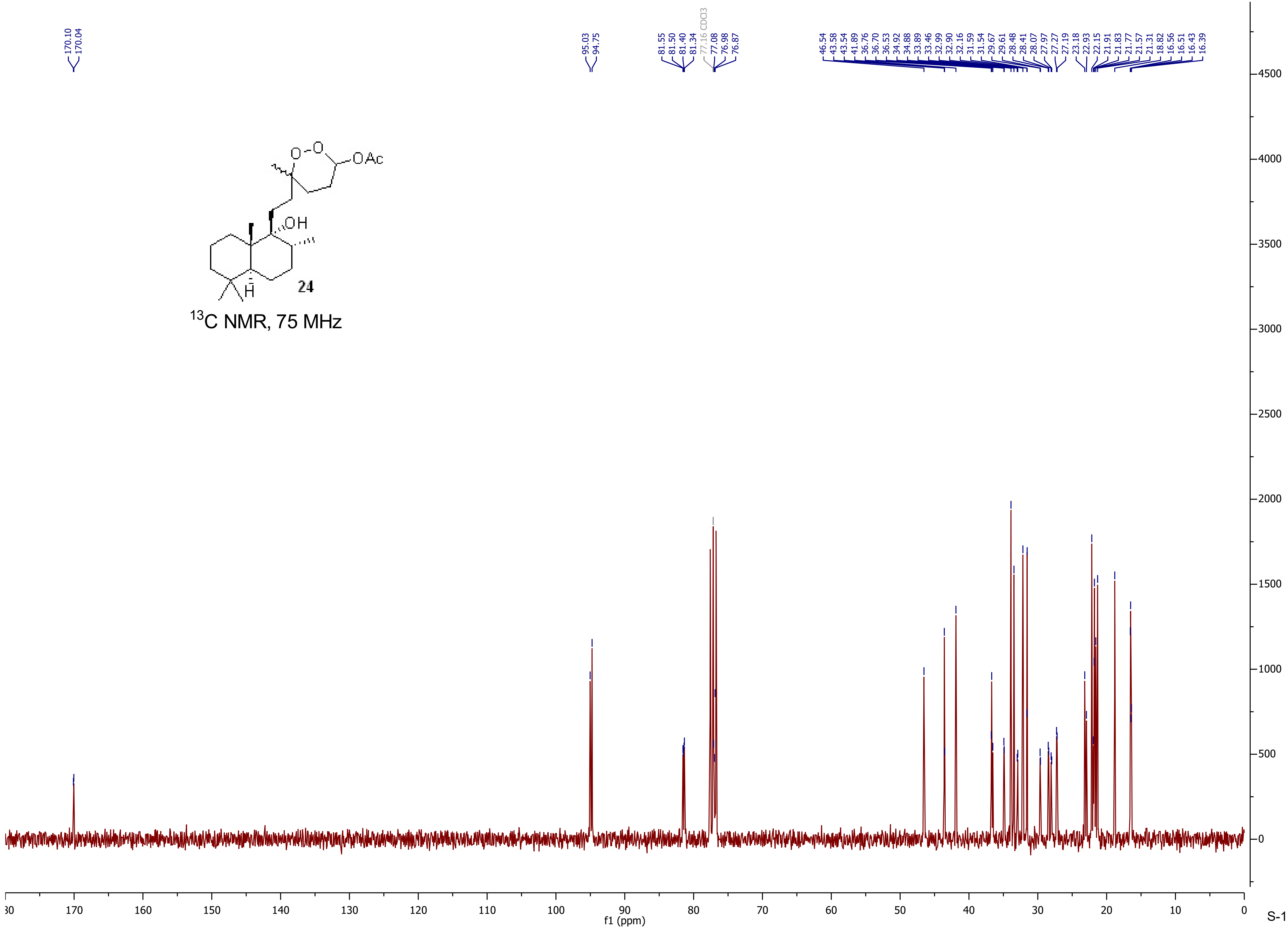
170.10
170.04
95.03
94.75
81.55
81.50
81.40
81.34
77.16 CDCl3
77.08
76.98
76.87
46.54
43.58
43.54
41.89
36.76
36.70
36.53
34.92
34.88
33.89
33.46
32.99
32.90
32.16
31.59
31.54
29.67
29.61
28.48
28.41
28.07
27.97
27.27
27.19
23.18
22.93
22.15
21.91
21.83
21.77
21.57
21.31
18.82
16.56
16.51
16.43
16.39
O-O
OAc
OH
H
24
13C NMR, 75 MHz
f1 (ppm)

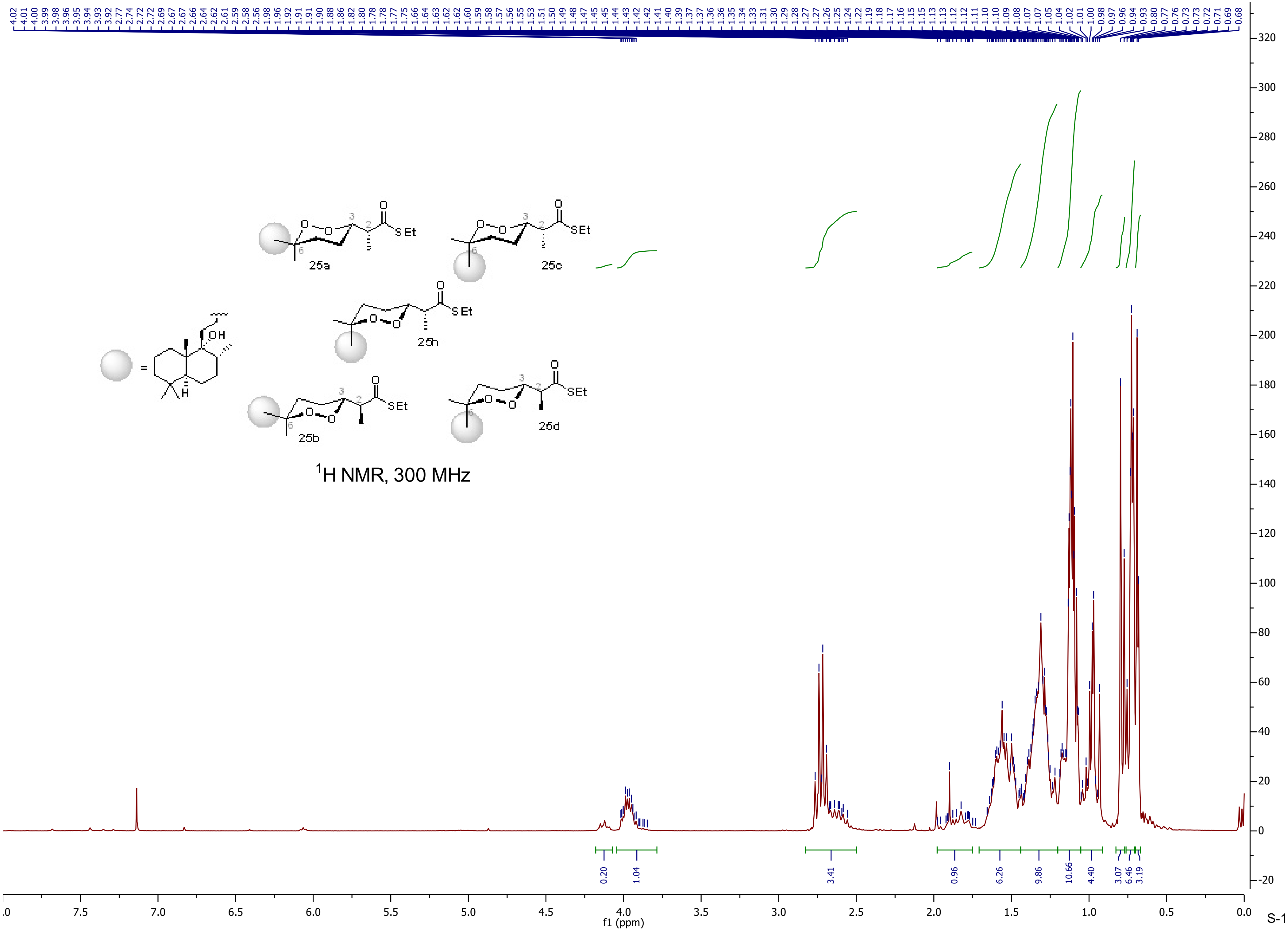
25a
25c
25h
25b
25d
SEt
OH
H
^{1}H NMR, 300 MHz
f1 (ppm)
0.20
1.04
3.41
0.96
6.26
9.86
10.66
4.40
3.07
6.46
3.19

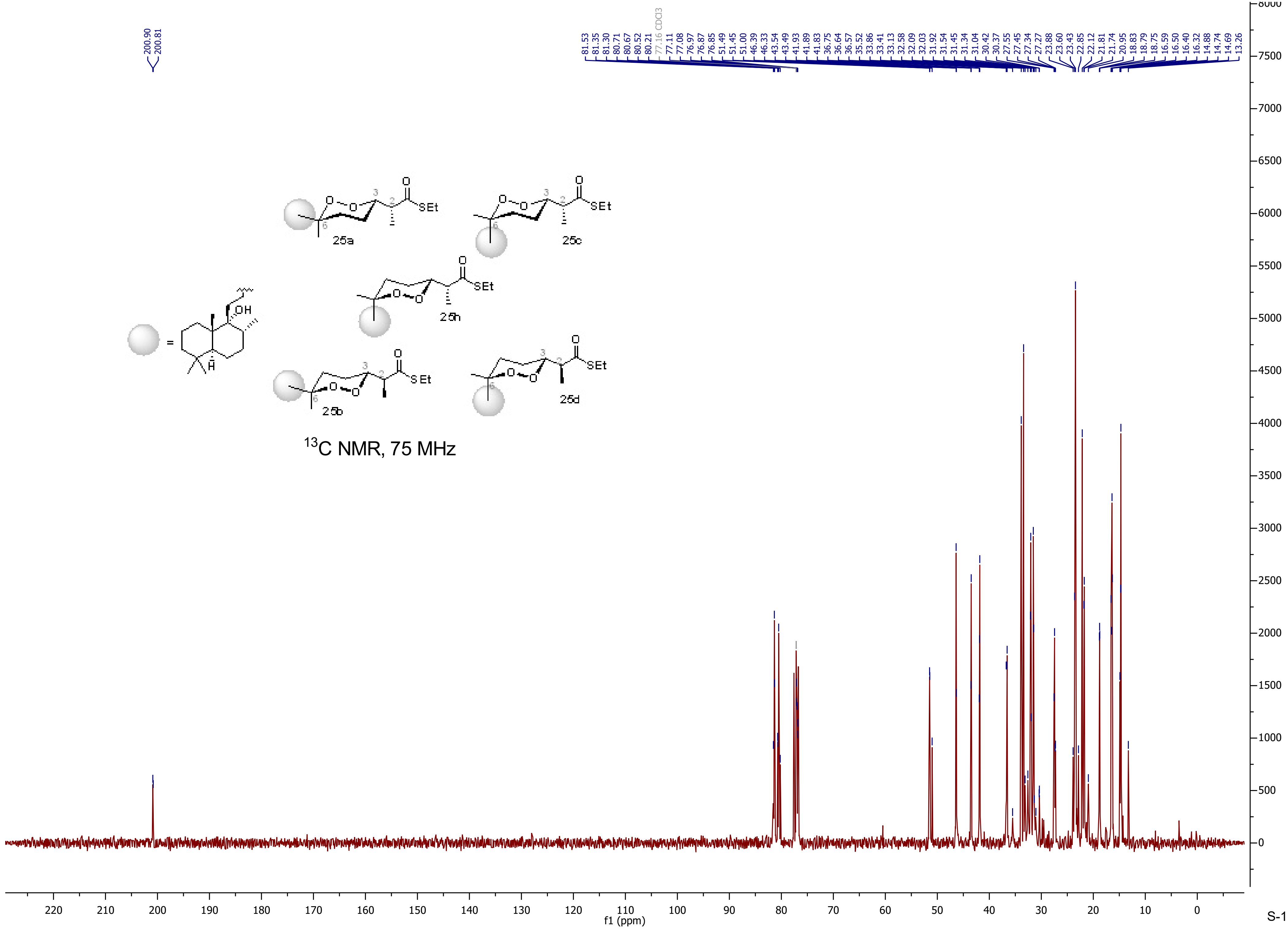
200.90
200.81
81.53
81.35
81.30
80.71
80.67
80.52
80.21
77.16 CDCl3
77.11
77.08
76.97
76.87
76.85
51.49
51.45
51.00
46.39
46.33
43.54
43.49
41.93
41.89
41.83
36.75
36.64
36.57
35.52
33.86
33.41
33.13
32.58
32.09
32.03
31.92
31.54
31.45
31.34
31.04
30.42
30.37
27.55
27.45
27.34
27.27
23.88
23.60
23.43
22.85
22.12
21.81
21.74
20.95
18.83
18.79
18.75
16.59
16.50
16.40
16.32
14.88
14.74
14.69
13.26
25a
25c
25h
25b
25d
SEt
OH
^{13}C NMR, 75 MHz
f1 (ppm)

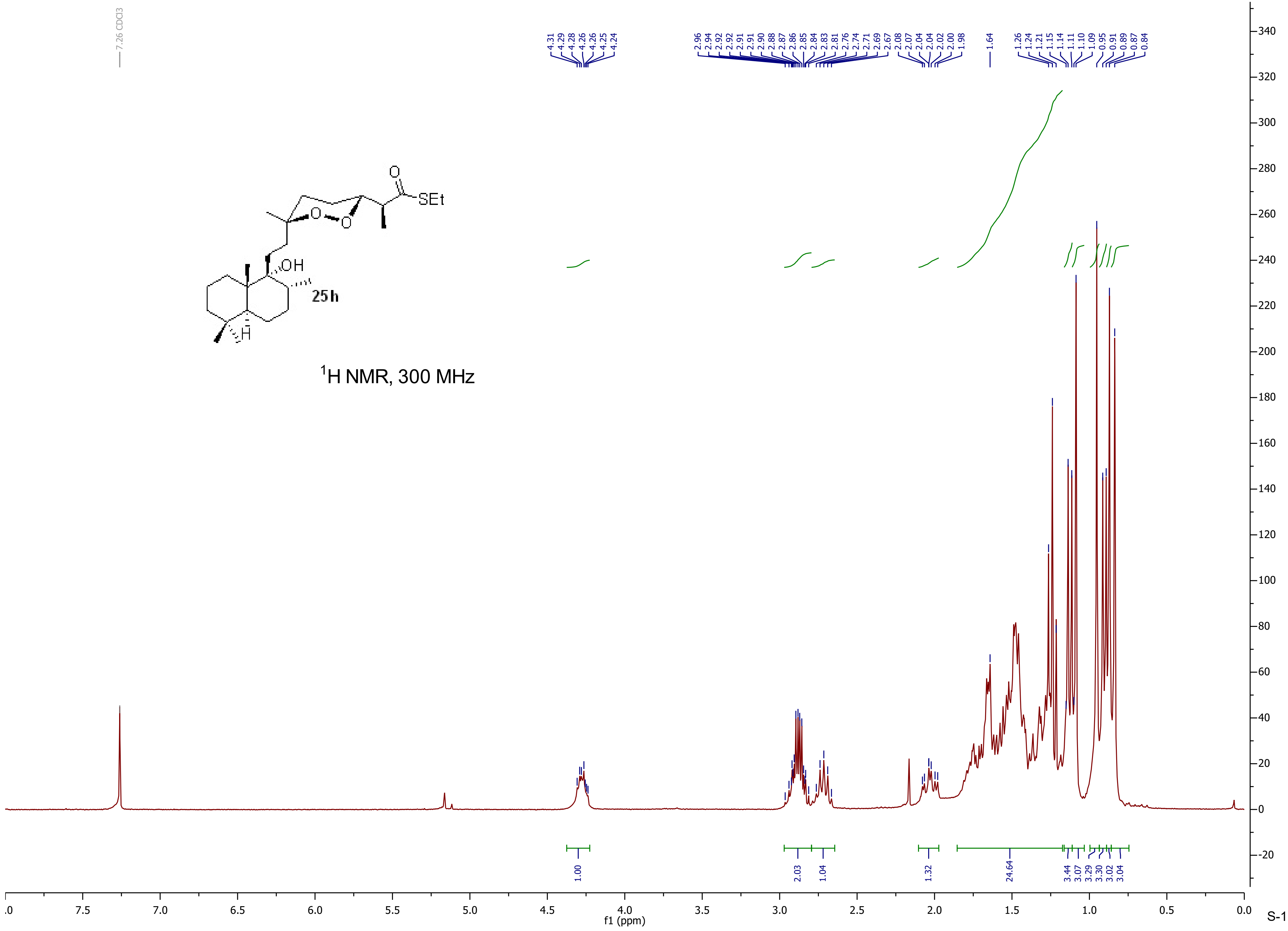

7.26 CDCl3
4.31 4.29 4.28 4.26 4.26 4.25 4.24
2.96 2.94 2.92 2.91 2.91 2.90 2.88 2.87 2.86 2.85 2.84 2.83 2.81 2.76 2.74 2.71 2.69 2.67 2.08 2.07 2.04 2.04 2.02 2.00 1.98
1.64
1.26 1.24 1.21 1.15 1.14 1.11 1.10 1.09 0.95 0.91 0.89 0.87 0.84
O
SEt
O
O
OH
H
25h
1H NMR, 300 MHz
1.00
2.03
1.04
1.32
24.64
3.44 3.07 3.29 3.30 3.02 3.04
f1 (ppm)

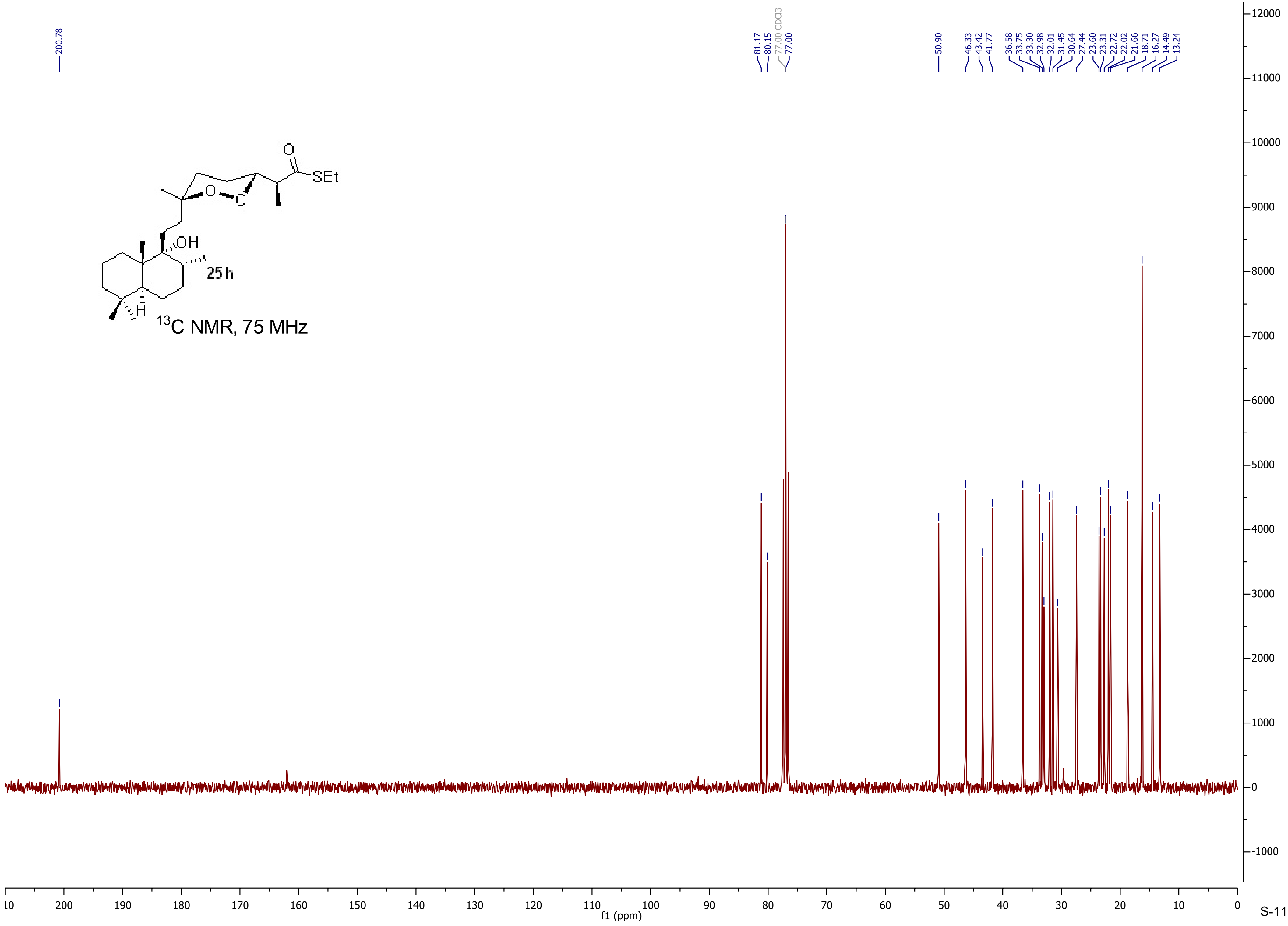
200.78
81.17
80.15
77.00 CDCl3
77.00
50.90
46.33
43.42
41.77
36.58
33.75
33.30
32.98
32.01
31.45
30.64
27.44
23.60
23.31
22.72
22.02
21.66
18.71
16.27
14.49
13.24
O
SEt
O
O
OH
H
25h
^{13}C NMR, 75 MHz
f1 (ppm)

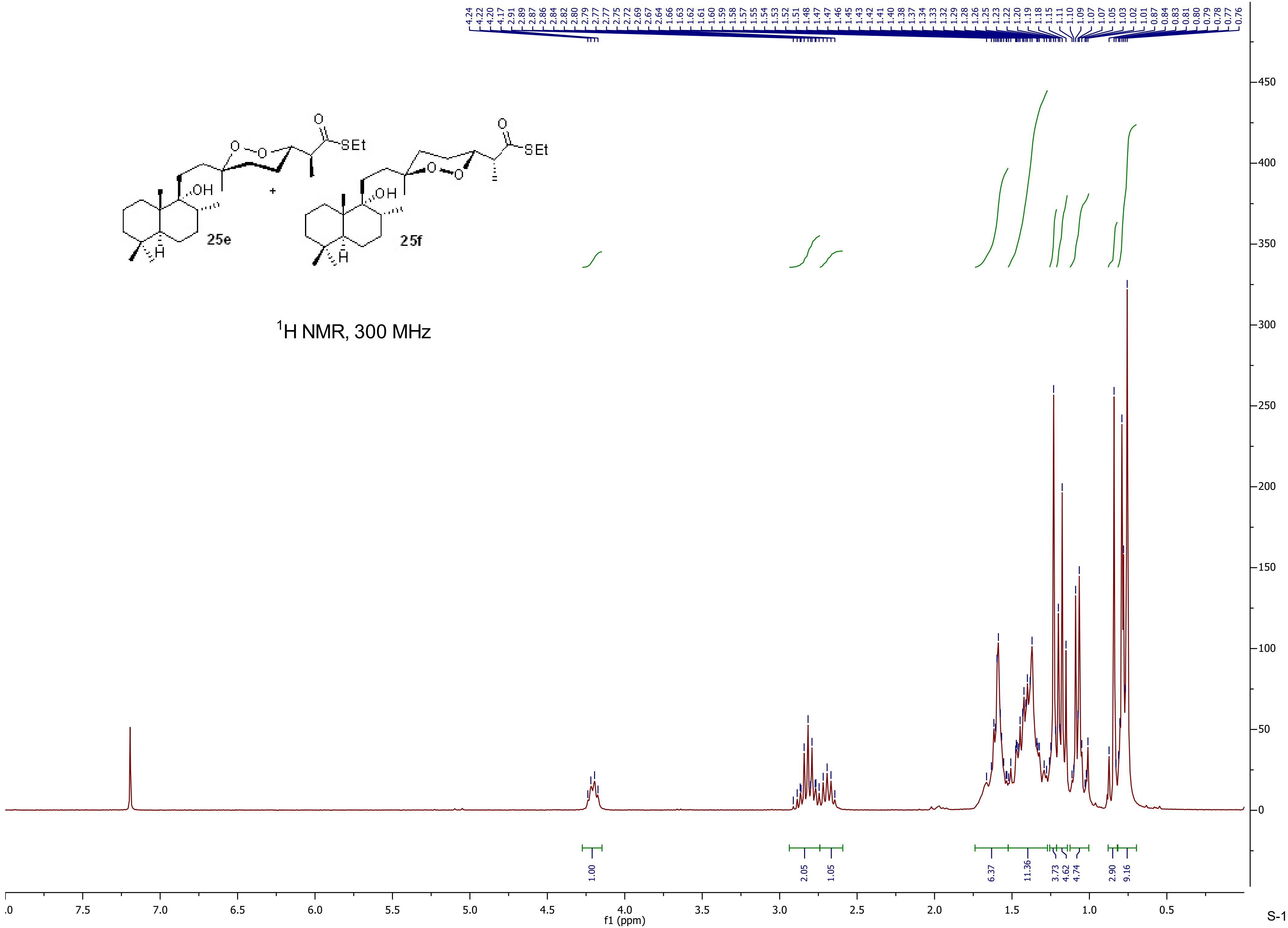

25e
25f
SEt
OH
^{1}H NMR, 300 MHz
4.24 4.22 4.20 4.17 2.91 2.89 2.87 2.86 2.84 2.82 2.80 2.79 2.77 2.75 2.72 2.69 2.67 2.64 1.66 1.63 1.62 1.61 1.60 1.59 1.58 1.57 1.55 1.54 1.53 1.52 1.51 1.48 1.47 1.47 1.46 1.45 1.43 1.42 1.41 1.40 1.38 1.37 1.34 1.33 1.32 1.29 1.28 1.26 1.25 1.23 1.22 1.20 1.19 1.18 1.15 1.11 1.10 1.09 1.07 1.07 1.05 1.03 1.02 1.01 0.87 0.84 0.83 0.81 0.80 0.79 0.78 0.77 0.76
1.00 2.05 1.05 6.37 11.36 3.73 4.62 4.74 2.90 9.16
f1 (ppm)

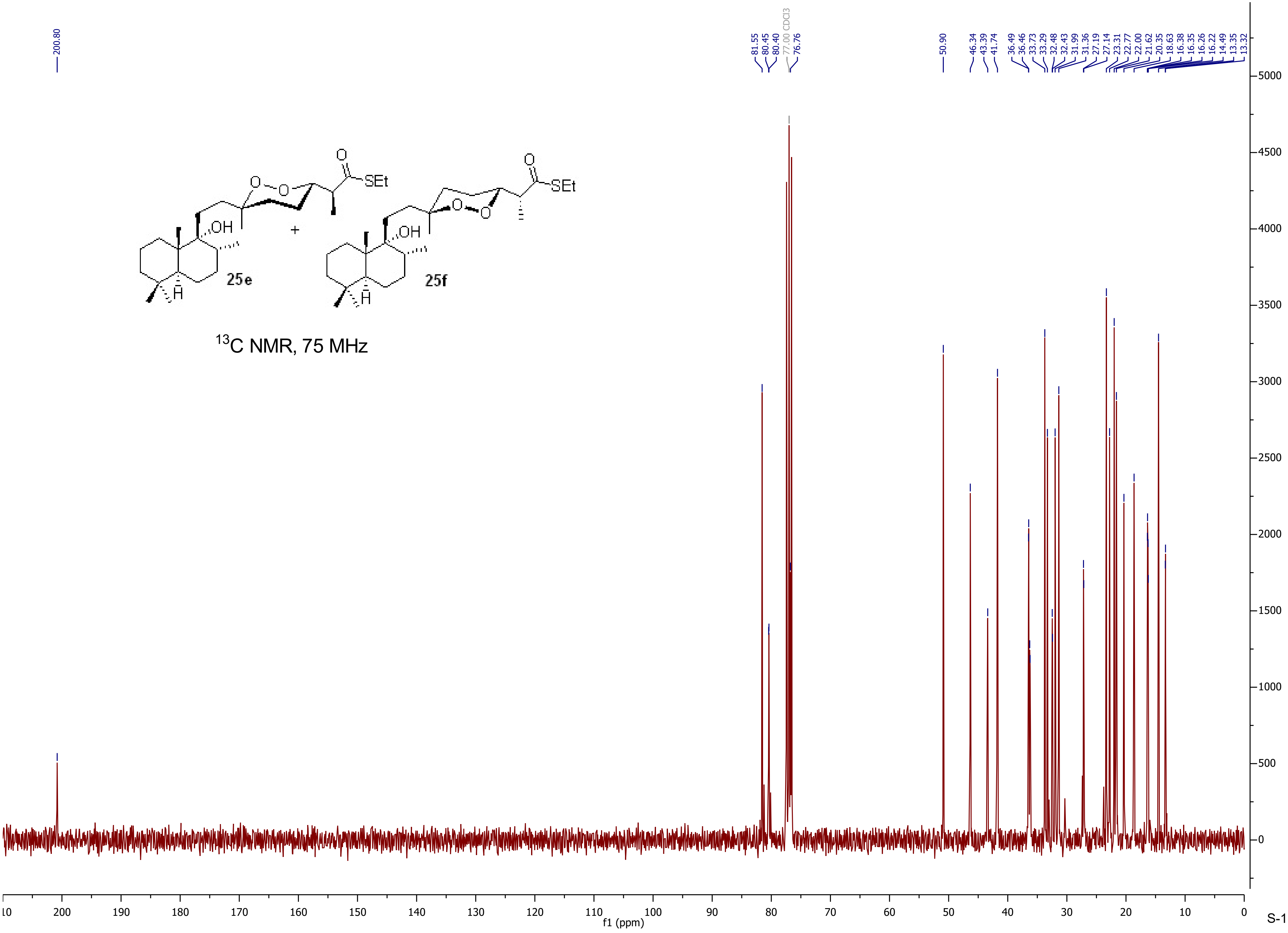
200.80
81.55
80.45
80.40
77.00 CDCl3
76.76
50.90
46.34
43.39
41.74
36.49
36.46
33.73
33.29
32.48
32.43
31.99
31.36
27.19
27.14
23.31
22.77
22.00
21.62
20.35
18.63
16.38
16.35
16.26
16.22
14.49
13.35
13.32
O
SEt
O
O
OH
+
25e
H
25f
^{13}C NMR, 75 MHz
f1 (ppm)

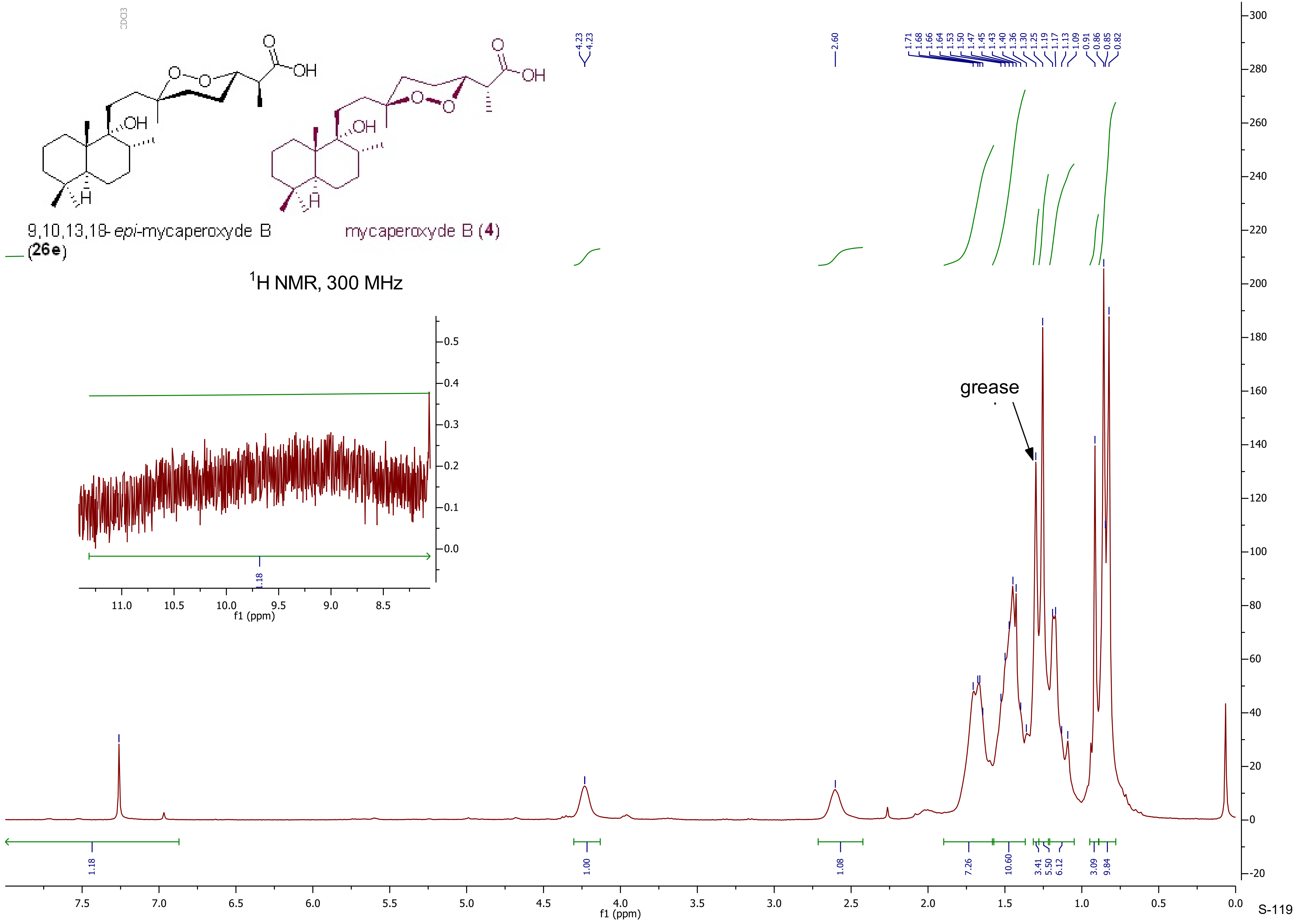
9,10,13,18-epi-mycaperoxyde B
(26e)
mycaperoxyde B (4)
1H NMR, 300 MHz
grease
CDCl3
f1 (ppm)

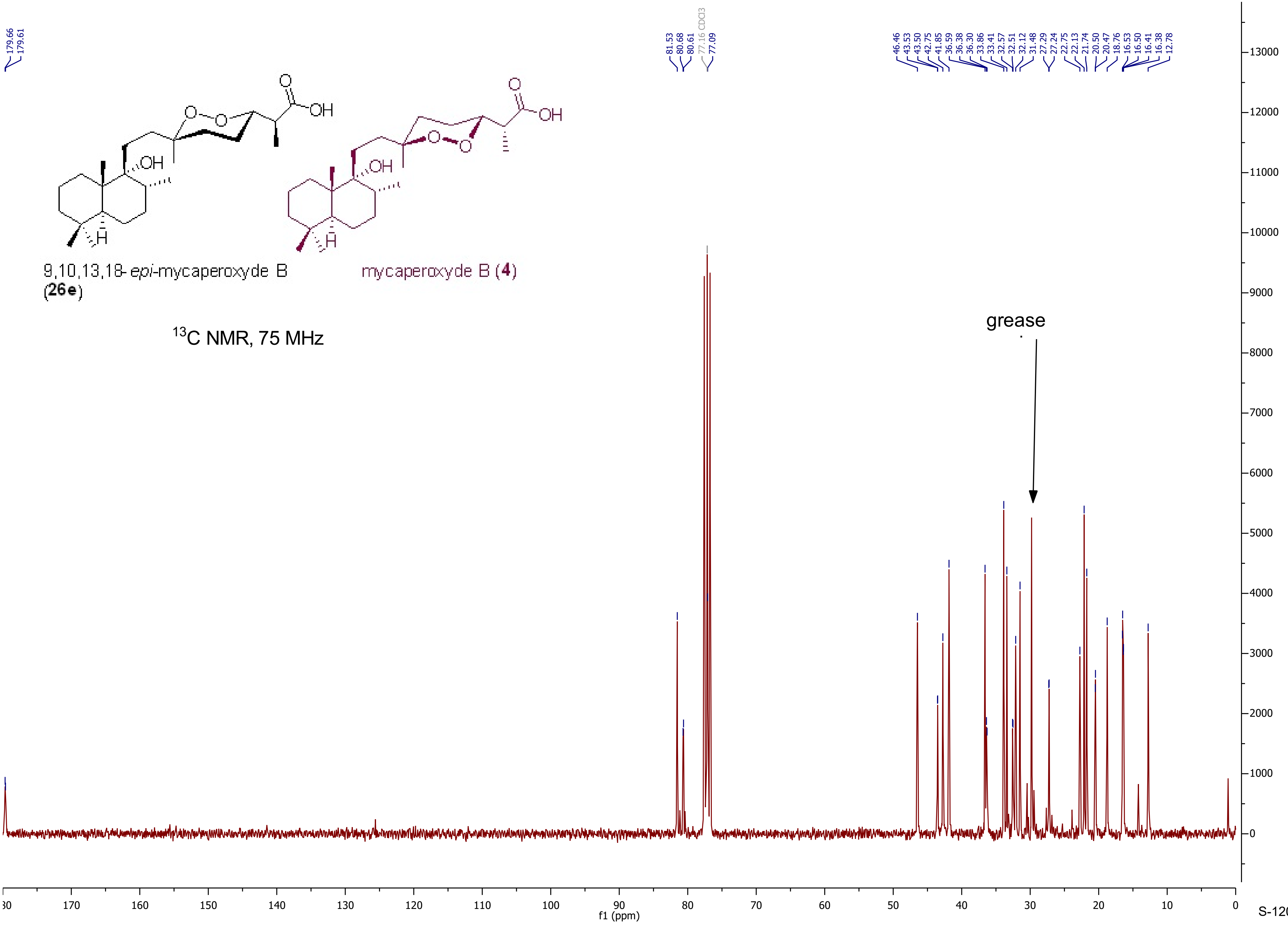
179.66
179.61
81.53
80.68
80.61
77.16 CDCl3
77.09
46.46
43.53
43.50
42.75
41.85
36.59
36.38
36.30
33.86
33.41
32.57
32.51
32.12
31.48
27.29
27.24
22.75
22.13
21.74
20.50
20.47
18.76
16.53
16.50
16.41
16.38
12.78
9,10,13,18-*epi*-mycaperoxyde B (**26e**)
mycaperoxyde B (**4**)
^{13}C NMR, 75 MHz
grease
f1 (ppm)

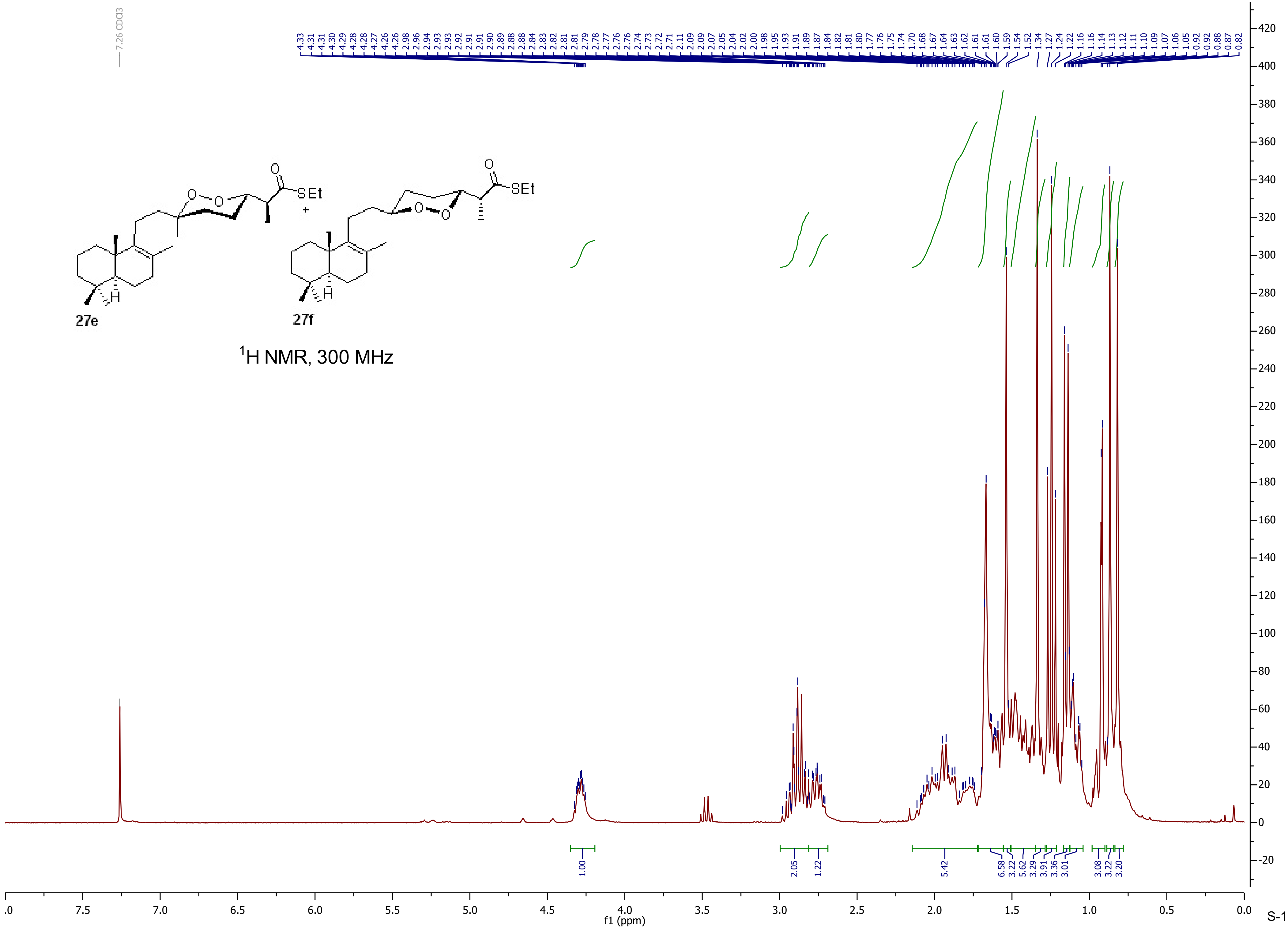
7.26 CDCl3
SEt
27e
27f
1H NMR, 300 MHz
f1 (ppm)

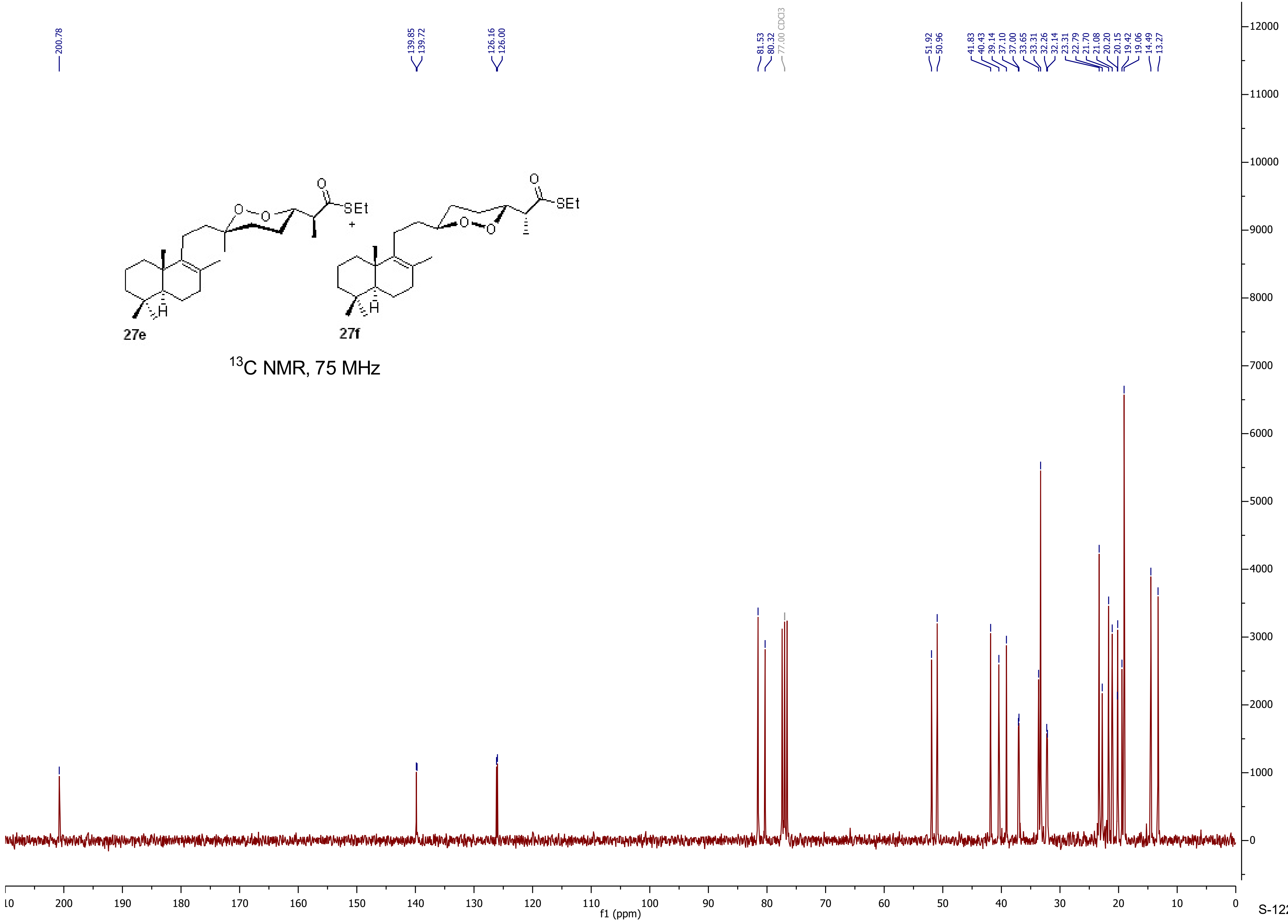
200.78
139.85
139.72
126.16
126.00
81.53
80.32
77.00 CDCl3
51.92
50.96
41.83
40.43
39.14
37.10
37.00
33.65
33.31
32.26
32.14
23.31
22.79
21.70
21.08
20.20
20.15
19.42
19.06
14.49
13.27
O
SEt
+
27e
27f
13C NMR, 75 MHz
f1 (ppm)

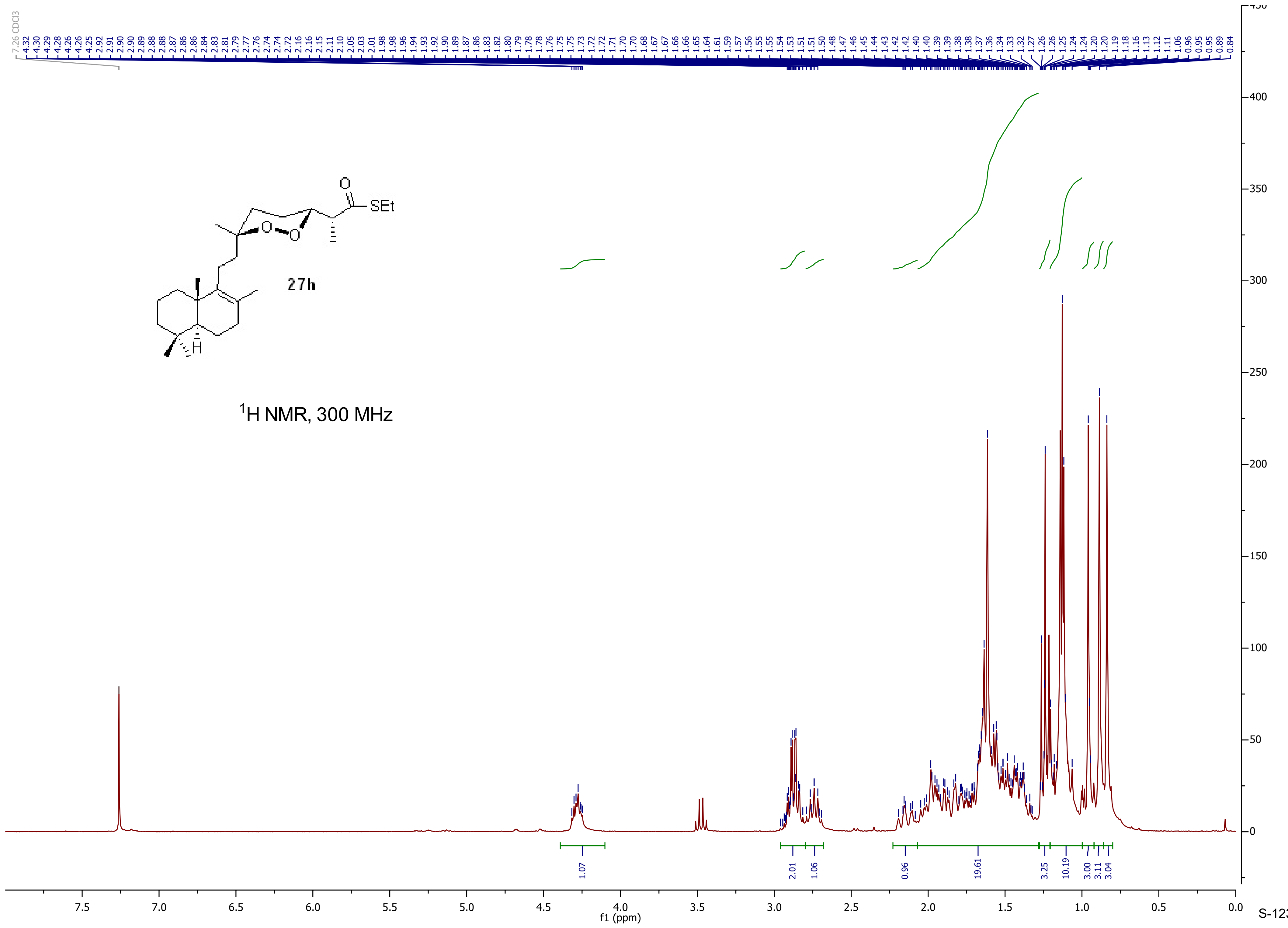
7.26 CDCl3
4.32 4.30 4.29 4.28 4.26 4.26 4.25 2.92 2.91 2.90 2.90 2.89 2.88 2.88 2.87 2.86 2.86 2.84 2.83 2.81 2.79 2.77 2.76 2.74 2.74 2.72 2.16 2.16 2.15 2.11 2.10 2.05 2.03 2.01 1.98 1.98 1.96 1.94 1.93 1.92 1.90 1.89 1.87 1.86 1.83 1.82 1.80 1.79 1.78 1.78 1.76 1.75 1.75 1.73 1.72 1.72 1.71 1.70 1.70 1.68 1.67 1.67 1.66 1.66 1.65 1.64 1.61 1.59 1.57 1.56 1.55 1.55 1.54 1.53 1.51 1.51 1.50 1.48 1.47 1.46 1.45 1.44 1.43 1.42 1.42 1.40 1.40 1.39 1.39 1.38 1.38 1.37 1.36 1.34 1.33 1.32 1.27 1.26 1.26 1.25 1.24 1.24 1.20 1.20 1.19 1.18 1.16 1.13 1.12 1.11 1.06 0.96 0.95 0.89 0.84
O
SEt
O
O
H
27h
1H NMR, 300 MHz
1.07
2.01
1.06
0.96
19.61
3.25
10.19
3.00
3.11
3.04
7.5 7.0 6.5 6.0 5.5 5.0 4.5 4.0 3.5 3.0 2.5 2.0 1.5 1.0 0.5 0.0
f1 (ppm)
400 350 300 250 200 150 100 50 0

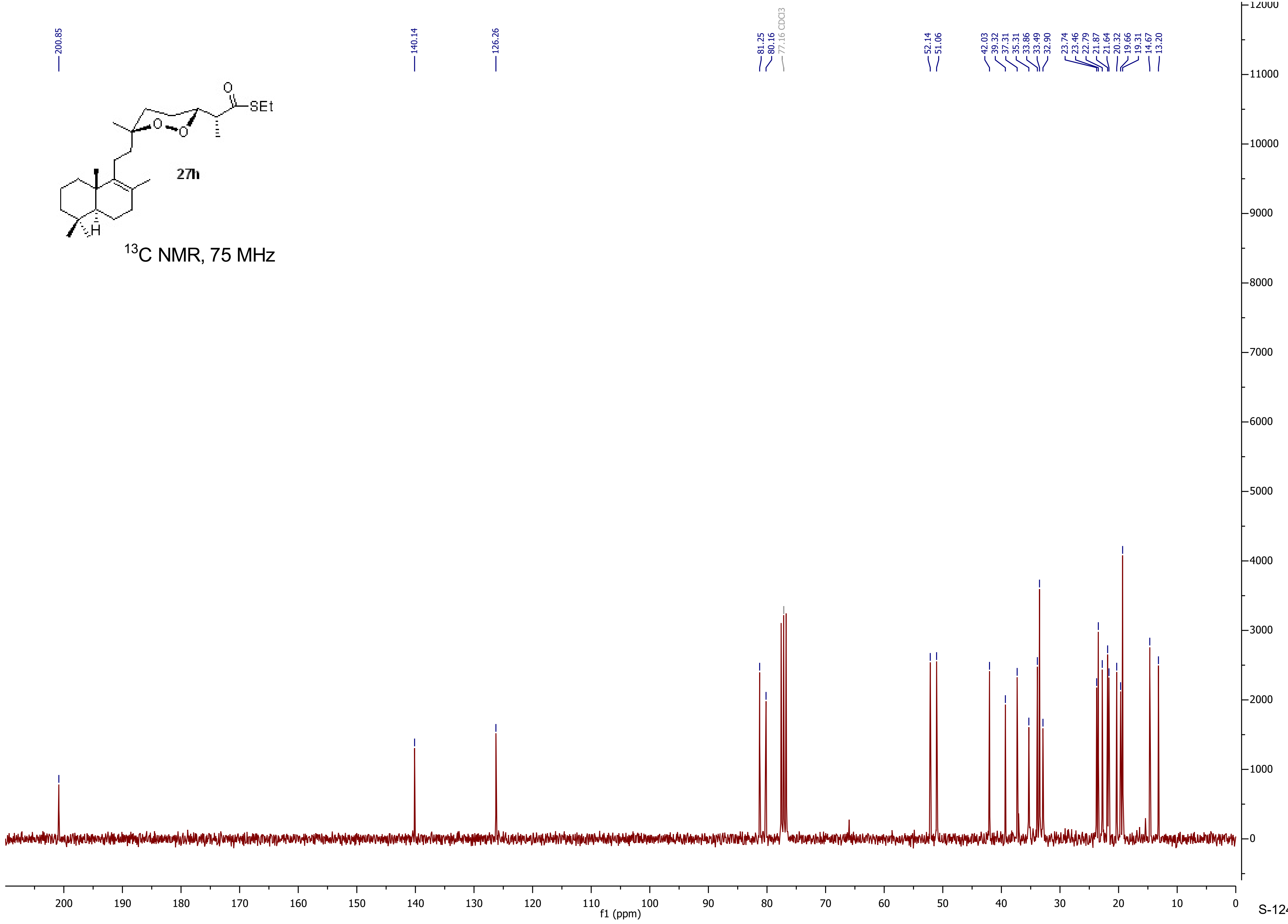

27h
13C NMR, 75 MHz
SEt
200.85
140.14
126.26
81.25
80.16
77.16 CDCl3
52.14
51.06
42.03
39.32
37.31
35.31
33.86
33.49
32.90
23.74
23.46
22.79
21.87
21.64
20.32
19.66
19.31
14.67
13.20
f1 (ppm)

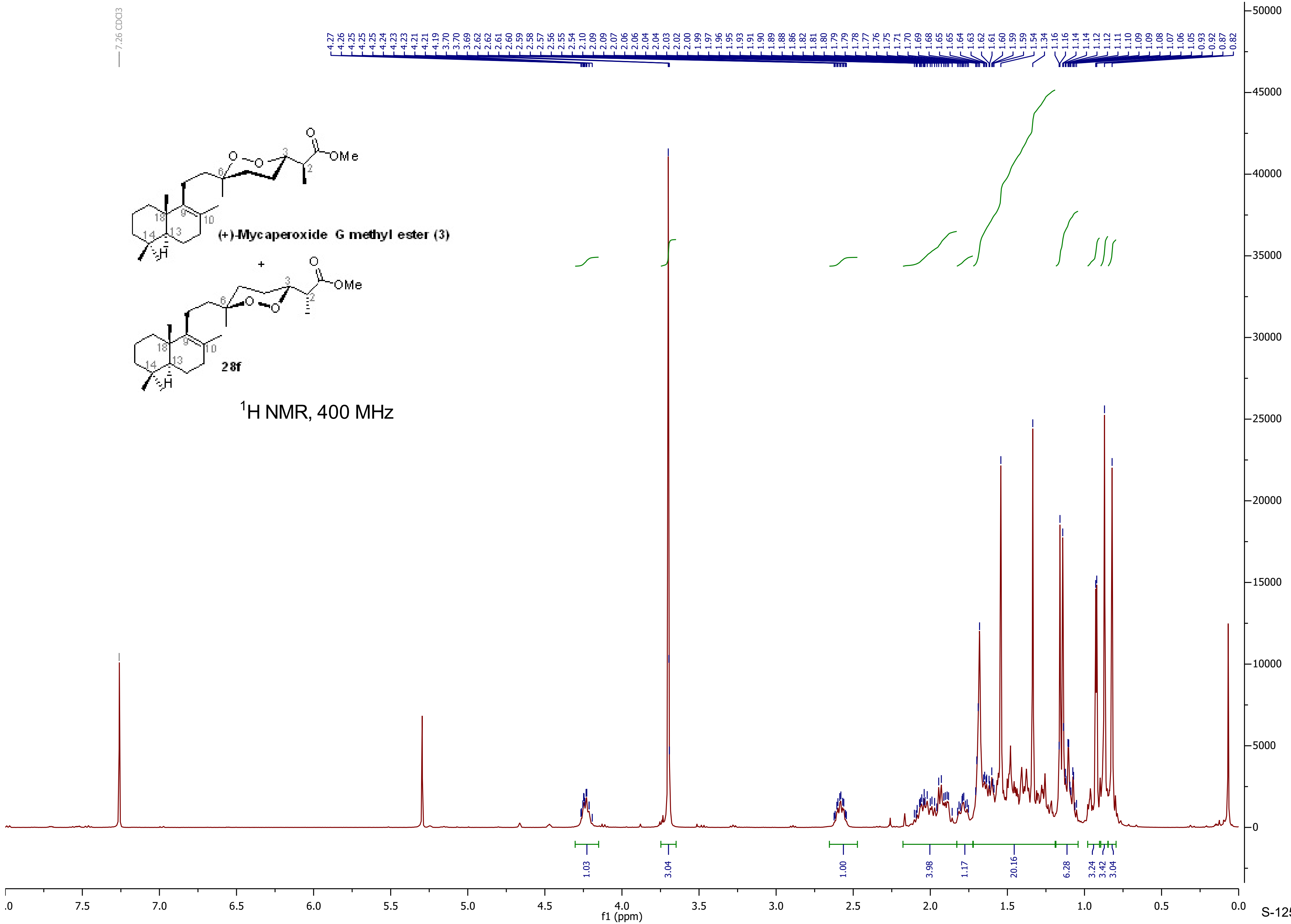

(+)-Mycaperoxide G methyl ester (3)
28f
¹H NMR, 400 MHz
f1 (ppm)

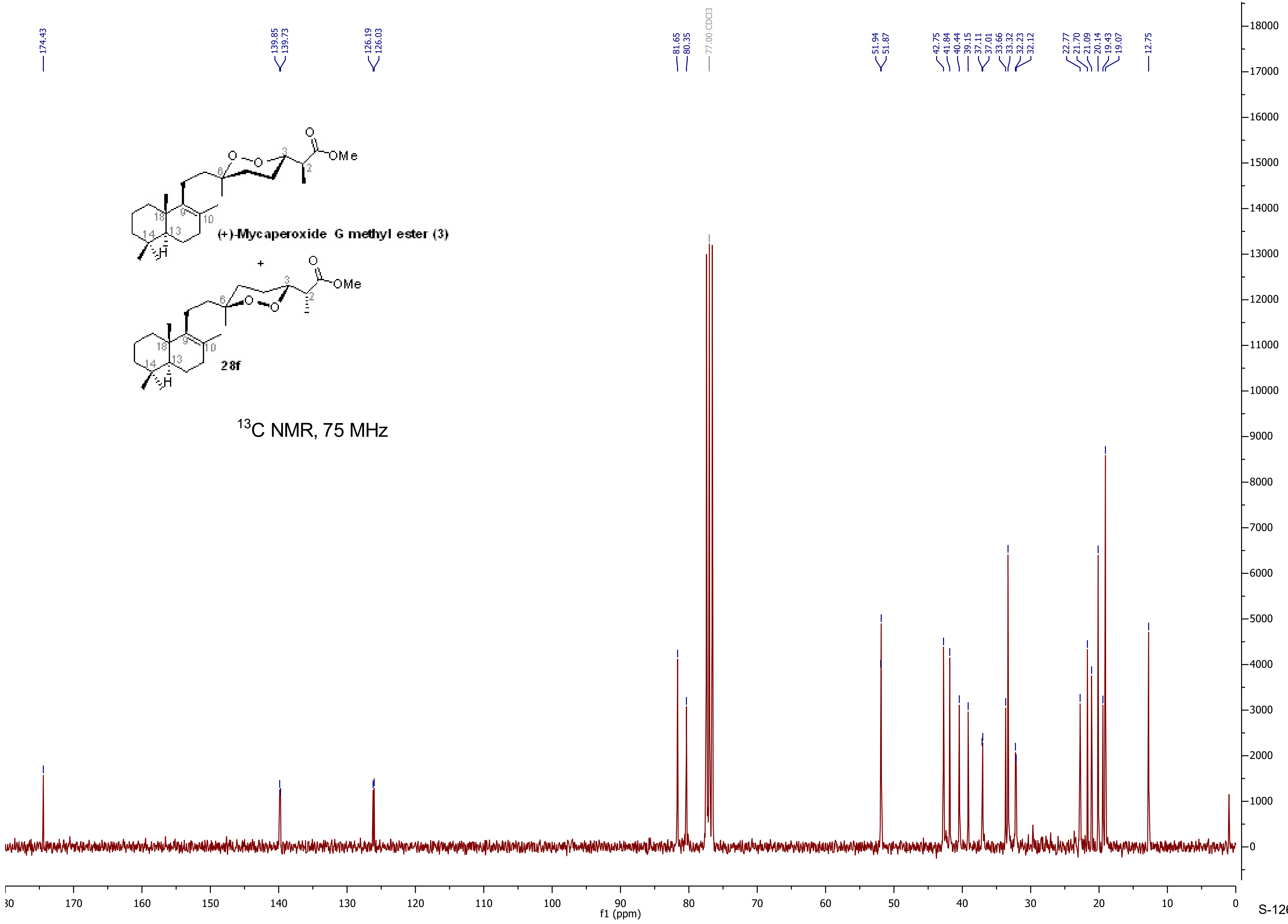

174.43
139.85
139.73
126.19
126.03
81.65
80.35
77.00 CDCl3
51.94
51.87
42.75
41.84
40.44
39.15
37.11
37.01
33.66
33.32
32.23
32.12
22.77
21.70
21.09
20.14
19.43
19.07
12.75
(+)-Mycaperoxide G methyl ester (3)
+
28f
OMe
^{13}C NMR, 75 MHz
f1 (ppm)
